# In situ formation of adaptive electronic skin in 2 seconds enabled by metal coordination

**DOI:** 10.1038/s41467-026-73303-w

**Published:** 2026-05-16

**Authors:** Xiaojuan Wang, Xiaosen Pan, Junzhi Jiang, Wanlong Song, Xiaoqi Zhou, Xu Lin, Jingye Zhao, Zhengjian Zhang, Zhenxing Liu, Xiaojun Ma, Hongbin Liu, Meng Gao

**Affiliations:** 1 State Key Laboratory of Biobased Fiber Manufacturing Technology, Tianjin University of Science and Technology, Tianjin, China; 2 Institute of Applied Physics and Materials Engineering, University of Macau, Macao, China; 3 School of Biotechnology, Tianjin University of Science and Technology, Tianjin, China

**Keywords:** Self-assembly, Electronic devices

## Abstract

Epidermal electronics, which are flexible and conformable electronic systems designed to interact seamlessly with human skin, hold great promise for healthcare monitoring and personal electronics. However, traditional fabrication methods face challenges of reliance on non-sustainable materials, intricate and time-consuming processes, and material softness-induced fragile transfer to target substrates. Inspired by the “milk skin” phenomenon, we developed a rapid dipping-dipping molecular assembly method to fabricate cellulose-based bio-skin in situ within seconds, exhibiting ultra-thin, highly conformal, shape-customizable, degradable, and low-impedance performances. This technique immerses substrates sequentially into carboxymethyl cellulose (CMC) and Cu(II) solutions, leveraging strong metal-coordination interactions. Membrane formation efficiency, influenced by the oxidation and coordination characteristics of metal ions, follows the order: Cu(II) > Fe(II) > Ca(II). CMC-Ag(I)/CMC-Cu(II) form stable membranes, whereas CMC-Fe(II) forms fragmented structures, and CMC-Mg(II)/CMC-Ca(II) remain in solution. This adaptable method can also be extended to other biomacromolecules like methylcellulose and carboxymethyl chitosan, broadening applications. The bio-skin enables real-time monitoring of electrocardiograms (ECG), electrooculograms (EOG), electroencephalograms (EEG), and electromyograms (EMG), showcasing its potential for wearable, biocompatible electronics in healthcare.

## Abstract (web summary)

Electronic skins are promising for wearable electronics, though existing fabrication methods have unfavorable processes and mechanical properties. Here the authors utilize a dipping-dipping molecular assembly to fabricate an E-skin in seconds.

## Introduction

Electrically conductive membranes enable advanced epidermal electronics by providing flexibility and high electrical conductivity, with wide applications in medical and health monitoring, human-machine interaction, athletic performance monitoring, and personalised medicine^1–4^. The high-performance realisation of epidermal electronic devices critically depends largely on conformal interfaces with biological tissues^5^, high-resolution accuracy of target signal, and favourable biocompatibility^6–8^. However, its further development remains limited by the following challenges: (i) Multi-step and time-consuming fabrication procedure^9,10^; (ii) Delicate interface design requirements such as adhesion modulation and ultra-thin membrane fabrication for matching dynamically deforming biological tissues^11,12^; (iii) Limited self-standing property that hinders intact transfer onto target surface without damage^13–15^; (iv) Conventional petroleum-based materials commonly used facing inherent issues with environmental friendliness and biocompatibility^16,17^. Therefore, there is an urgent need for a simple and time-efficient fabrication strategy to develop biomaterial-based electronic membranes that conform to living tissues, enabling accurate and high-fidelity physiological monitoring. Besides, if the electronic membrane can be formed in situ on demand, the membrane could better maintain its integrity, since no additional transfer to the target tissue or skin is required^18^.

**Fig. 1:**
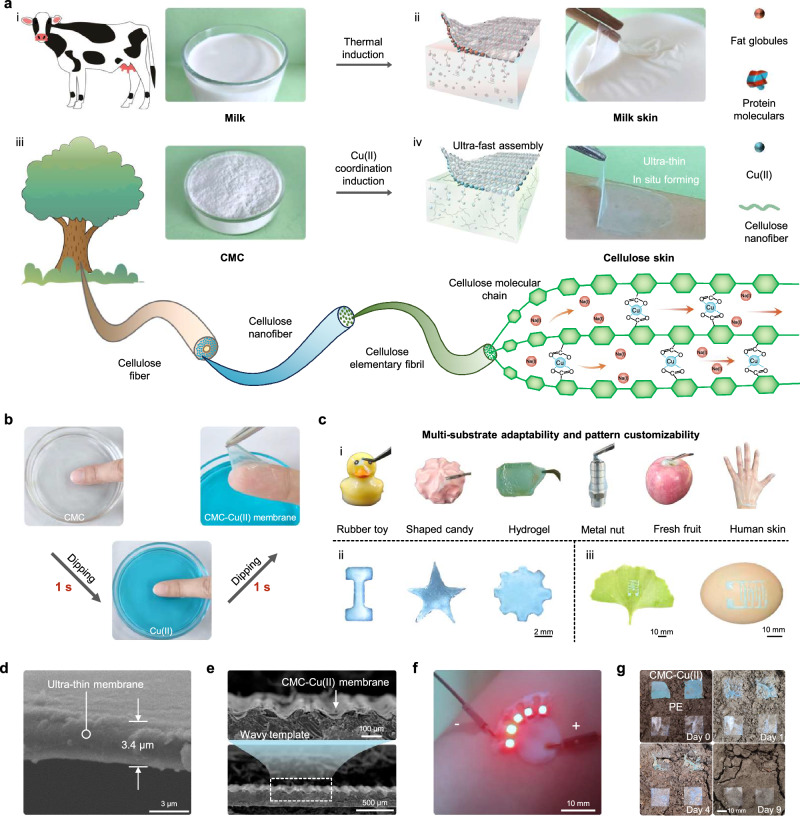
Bioinspired fabrication and characterization of CMC-Cu(II) membrane via metal ion-driven assembly strategy, mimicking the ‘milk skin effect’. **a** Schematic illustration of the formation of milk skin and cellulose skin. **i** Photograph of milk source (cow) and a cup of milk. **ii** Thermal-induced molecular assembly during milk skin formation and a digital image of milk skin. **iii** Multilevel structure of cellulose and a photograph of carboxymethylcellulose (CMC) powder. CMC, as a typical cellulose-derived macromolecule, is obtained from wood cellulose fiber. **iv** The molecular assembly promoted by strong coordination of Cu(II) ions and CMC molecules contributes to the rapid generation of CMC-Cu(II) ultra-thin conductive bio-membrane. The icons on the right denote fat globules (brown spheres), protein molecules (red-blue structures), Cu(II) ions (light blue spheres), and cellulose nanofibers (green wavy lines), respectively. **b** Digital photographs of CMC-Cu(II) membrane with Cu(II) coordination fabricated by simple dipping-dipping process from CMC and Cu(II) solutions. **c** The adaptable, highly conformal CMC-Cu(II) membrane assembled in situ on irregular surfaces of multi-substrates (**i**). Digital photographs of CMC-Cu(II) patterns (**ii**) on diverse 2D and 3D surfaces (**iii**). **d** Cross-sectional SEM image of ultra-thin CMC-Cu(II) membrane. Data are representative of 3 independent experiments, with similar results obtained in each replicate. **e** Cross-sectional SEM images of highly conformal CMC-Cu(II) membrane attached on polyvinyl chloride (PVC) wavy mold. Data are representative of three independent experiments, with similar results obtained in each replicate. **f** Digital image of CMC-Cu(II) patterned microelectronic circuit on human skin and its ability to illuminate LED lights. The “+“ and “-“ signs indicate the positive and negative electrodes of the LED circuit. **g** The degradation photographs of CMC-Cu(II) membranes and polyethylene (PE) films.

As one of the most exciting frontiers in materials science, molecular self-assembly emerges as a promising candidate for realising convenient and reliable production of bio-membranes due to its tunability and flexibility. It is a spontaneous process of aggregating from disorder to order, driven by non-covalent interactions such as hydrogen bonding, van der Waals forces, electrostatic interactions, and π-π stacking, without external intervention^19,20^. Self-assembly is widespread in nature, exemplified by the formation of the DNA double helix and the milk-skin effect upon heating^21,22^. Inspired by these natural processes, precise control of intermolecular interactions enables the design and construction of materials with tailored functions and structures, providing a versatile platform for developing membrane materials across multiple scales^23–25^. A notable example of thermally induced molecular self-assembly is the formation of milk skin (Fig. 1ai, ii). Heat unfolds the tertiary structure of coiled proteins, leading to the self-assembly of these molecules into an ordered milk-lamellar membrane. This process is driven by the synergistic effects of hydrophobic interactions, hydrogen bonding, and fat globules (Fig. 1aii)^22^. Leveraging this natural phenomenon, the rapid fabrication of on-demand, conformal bioconductive membranes through molecular self-assembly presents a promising strategy.

Here, we present a convenient and time-efficient strategy for the synthesis of biodegradable electronic bio-membranes through strong metal coordination interactions^26–28^, enabling ultra-fast, in situ, and adaptive assembly of biomacromolecules. By sequentially dipping the target substrate, regardless of its structure, first in carboxymethylcellulose (CMC) and then in a Cu(II) solution, an intact, robust bioconductive skin (CMC-Cu(II)) can be formed instantly within 2 s (Fig. 1aiii, iv). Hereafter, notations such as CMC‑Cu(II), CMC‑Fe(II), etc. refer to the complexes formed between CMC and the corresponding metal ions. Research demonstrates that the capacity for instant in situ membrane formation correlates with the oxidation state and coordination characteristics of the metal ions, following the trend: Cu(II) > Fe(II) > Ca(II). Specifically, at the same experimental conditions, CMC-Ag(I) and CMC-Cu(II) form well-defined, stable membranes, while CMC-Fe(II) exhibits a fragmented morphology, indicating incomplete formation, and CMC-Mg(II) and CMC-Ca(II) remain in the solution state without significant changes. This trend also applies to other biomacromolecules, such as methylcellulose (MC) and carboxymethyl chitosan (CMCH), further extending the method's applicability beyond CMC. The resulting CMC-based bio-membranes can serve as epidermal electronics for real-time physiological signal monitoring, including electrocardiogram (ECG), electrooculogram (EOG), electroencephalogram (EEG), and electromyography (EMG), demonstrating their potential for wearable, biocompatible electronics in healthcare applications.

## Results

### Synthesis and characterization of CMC-Cu(II) membrane

The ultra-thin conductive bio-molecular skin (CMC-Cu(II) membrane) can be achieved via a facile dipping-dipping coordination assembly process, which only needs the target item or surface to be sequentially immersed in CMC solutions and Cu(II) salt solutions (e.g., copper chloride or copper sulfate) (Fig. 1b, Supplementary Fig. 1 and Supplementary Movie 1-CMC-Cu(II) fabrication by dipping-dipping process). The entire contact-and-assembly process takes 2 s, with each dipping step lasting 1 s. In this recipe, CMC, a derivative of cellulose that is the most abundant biopolymer on Earth, is naturally biofriendly and enables for biodegradability of bioelectronic skin^29^. The Cu(II) ion is the key ingredient that ensures strong coordination and a rapid assembly process, which will be discussed in detail in the later section.

This simple dipping-dipping process enables on-demand in situ formation of adaptive, shape-conforming conductive bio-skin on materials with arbitrary surfaces. To demonstrate its conformal performance, we constructed a CMC-Cu(II) surface on varied materials, including a rubber model, a shaped candy, a polyacrylamide hydrogel, a metal nut, a fresh fruit (apple), and human palm skin (Fig.1ci). The CMC-Cu(II) membranes adhere seamlessly to these diverse surfaces, perfectly conforming to their shapes. Guided by the flexibility in the coordination assembly process, ultra-thin CMC-Cu(II) membranes can be achieved by controlling the concentration of CMC and Cu(II) (Supplementary Fig. 2). The cross-sectional scanning electron microscopy (SEM) image shows a 3.4 μm-thick CMC-Cu(II) membrane (Fig. 1d) fabricated from a 1 wt% CMC solution and a 0.5 M Cu(II) solution. The thin feature of the CMC-Cu(II) membrane further enhances its ability to conform to and match various interfaces^30,31^. It is noteworthy that the thickness of the CMC-Cu(II) membrane can be tuned by varying the Cu(II) concentration. As clearly illustrated in the SEM cross-sectional images presented in Supplementary Fig. 3, the membrane thickness exhibits a positive correlation with rising Cu(II) concentration. The SEM demonstrates that the CMC-Cu(II) membrane on the wavy polyvinyl chloride (PVC) template closely matches its microscopic contours (Fig. 1e and Supplementary Fig. 4), confirming that the proposed molecular assembly process fabricates ultra-thin CMC-Cu(II) membranes with favourable conformal adhesion, which is beneficial for the development of various applications.

The CMC-Cu(II) membrane exhibits good conductivity, enabling its effective use as a conductive pathway or epidermal electrode. In the CMC-Cu(II) membrane, coordination bonds between CMC and Cu(II) promote molecular aggregation while simultaneously forming ion channels that facilitate ion transport. In the typical commercially available CMC structure, the hydrogen atom on the hydroxyl group (–OH) at the C6 position of certain cellulose molecules are substituted with carboxymethyl groups (–CH₂COOH or –CH₂COONa). Most of these substituents terminate as sodium carboxylates (–COONa), in which the sodium ions (Na(I)) are ionically bonded to the carboxylate anions (–COO⁻). The incorporation of Cu(II) through coordination displaces Na(I) ions from their original sites, rendering them free and mobile within the system. Furthermore, water molecules in the membrane system are partially chelated with Cu(II), while some of the remaining free water molecules, acting as strong Lewis bases, form a solvation layer around Na(I) ions. These solvated Na(I) ions and unbound water molecules reside within the open ion-transport channels of the CMC-Cu(II) network, enabling efficient, directional ion mobility. This enhanced ionic mobility significantly improves the conductivity of the hydrated CMC-Cu(II) membrane (Supplementary Fig. 5)^32^.

Moreover, the facile dipping-dipping coordination assembly process allows CMC-Cu(II) membranes to be easily fabricated into custom-shaped patterns (Fig. 1cii) using a mask-forming approach (Supplementary Fig. 6). Unlike other conventional ways, this patterned conductive bio-skin can be formed in situ on various 2D and 3D surfaces directly (Fig. 1ciii), which eliminates the need for transfer and ensures conformal contact. Harnessing this characteristic, CMC-Cu(II) patterned microelectronic circuits are successfully integrated on human skin (Fig. 1f), providing a straightforward and effective approach for in situ manufacturing of flexible electronics. The preparation of CMC-Cu(II) membrane with defined size and shape on the skin via the mask-forming method is illustrated in Supplementary Fig. 7. Furthermore, a 90° peeling test reveals an average adhesion strength of 6.05 N/m for the CMC-Cu(II) membrane (Supplementary Fig. 8), which, together with the membrane’s conformability and ultra-thinness, ensures stable skin contact and signal reliability during electrophysiological monitoring.

Owing to the inherent biodegradability of CMC, the biodegradation of CMC-Cu(II) membrane in natural environments was investigated. Both CMC-Cu(II) membranes and polyethene (PE) films were placed on the soil surface. After 9 days, the CMC-Cu(II) membranes are visually degraded, whereas the polyethene (PE) films remain their original shape (Fig. 1g and Supplementary Fig. 9). These findings suggest rapid biodegradation of CMC-Cu(II) membranes in natural environments.

### Coordination interactions in CMC-Cu(II) membrane by molecular assembly

**Fig. 2:**
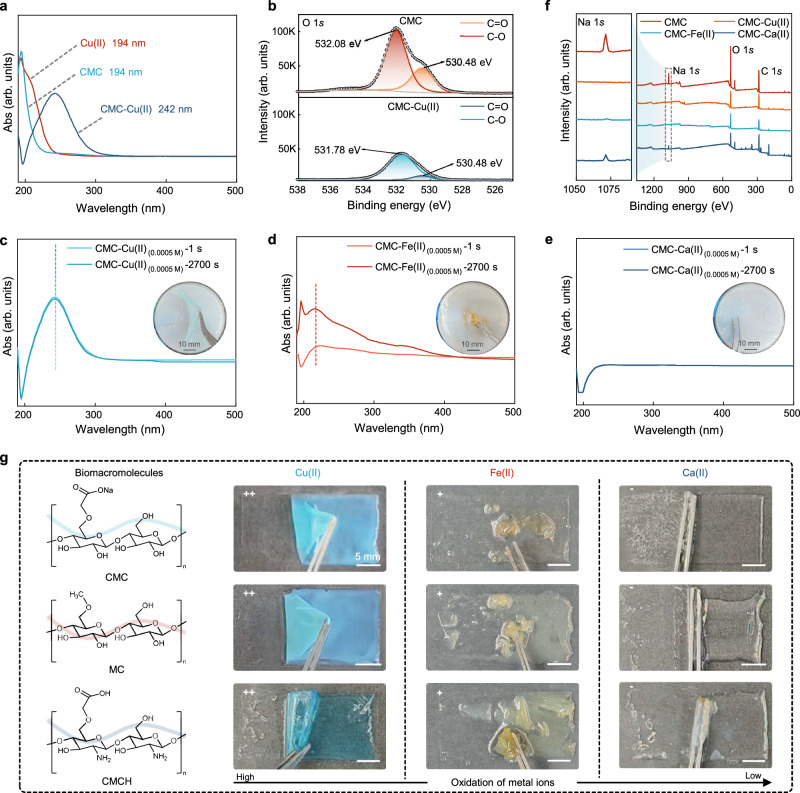
Mechanism validation and development of coordination interactions endowing molecular assembly to form membrane. **a** UV absorption spectra of CMC, Cu(II), and CMC-Cu(II), illustrating the coordination interaction between CMC and Cu(II). **b** The O 1 *s* fitted curves of CMC and CMC-Cu(II) membranes, demonstrating a significant reduction in the C-O and C = O functional groups in the CMC-Cu(II) membrane, providing strong evidence for the robust coordination between Cu(II) and CMC. UV spectra and membrane photographs of **c** CMC-Cu(II), **d** CMC-Fe(II), and **e** CMC-Ca(II) with different reaction time. **f** XPS survey spectra of Na element in CMC, CMC-Cu(II), CMC-Fe(II) and CMC-Ca(II). **g** Extending this membrane formation strategy and rule of metal ion-induced molecular assembly to other biomacromolecules such as MC and CMCH. “**++**” indicates that a complete and free-standing membrane can be formed; +” denotes a membrane that shows fragmented morphology and incomplete peeling; “–” indicates no membrane formation.

The Cu(II) ions, which possess strong coordination ability^33,34^, rapidly assemble CMC macromolecules by forming planar square coordinations with adjacent CMC chains, resulting in the instantaneous CMC-Cu(II) membrane formation. Upon contact, Cu(II) initially forms a coordinated bond with the oxygen atom of C6 carboxymethyl substituent on the CMC molecule, and thereafter experiences a ligand-to-metal charge transfer. Simultaneously, the partially filled *d* orbitals undergo energy level splitting, leading to *d-d* electron transitions. These changes result in ultraviolet-visible (UV-vis) absorption peaks at 242 nm, which are attributed to CMC-Cu(II) and are distinct from those of CMC (194 nm) and Cu(II) (194 nm). This indicates the formation of a separate material state, CMC-Cu(II) (Fig. 2a). The strong coordination between Cu(II) and CMC is further supported by the pronounced reduction in the intensities of C-O and C = O peaks in the O 1 *s* and C 1 *s* regions of the X-ray photoelectron spectroscopy (XPS) spectra for the CMC-Cu(II) membrane (Fig. 2b and Supplementary Fig. 10). Additionally, Fourier transform infrared (FTIR) spectra also confirm this through sharpened -OH peaks and shifted C-O stretching vibrations (Supplementary Fig. 11).

To further validate that the ultra-fast formation of CMC-Cu(II) membrane results from the strong coordination capacity of Cu(II), a series of common metal ions with varying oxidation states, including Ca(II), Mg(II), Mn(II), Zn(II), Fe(II), and Ag(I), were investigated for their molecular assembly capabilities in terms of membrane formation. It has been found that the membrane-forming abilities are influenced by their oxidation properties and coordination characteristics. For instance, at the same concentration, Ag(I) forms complete and stable membranes with CMC, Fe(II) creates incomplete membrane, and Zn(II), Mn(II), Mg(II), and Ca(II) remain in solution state (Supplementary Fig. 12). Among them, representative metal ions, Fe(II) and Ca(II), with distinct oxidation and coordination characteristics, were chosen for further investigation to evaluate their molecular assembly abilities, obtaining materials named CMC-Fe(II) and CMC-Ca(II). At the same concentration, compared to CMC-Cu(II), which shows well-formed complete membrane peeling (Fig. 2c inset and Supplementary Movie 1-CMC-Cu(II) fabrication by dipping-dipping process), CMC-Fe(II) displays fragmented morphology and incomplete peeling (Fig. 2d inset and Supplementary Movie 1-CMC-Fe(II) fabrication by dipping-dipping process) with an additional UV absorption peak (Supplementary Fig. 13a); while CMC-Ca(II) remains in solution form without any discernible macroscopic changes (Fig. 2e inset and Supplementary Movie 1-CMC-Ca(II) fabrication by dipping-dipping process) and exhibits a negative UV absorption peak (Supplementary Fig. 13b). This phenomenon may be attributed to the weak coordination between Ca(II) and CMC, leading to the formation of microscopic aggregates or invisible turbidity. When the size of these aggregates approaches or exceeds the wavelength of incident light, significant light scattering occurs. This scattered light may compensate for, or even surpass, the attenuation of direct transmitted light caused by both scattering and absorption, resulting in the observation of a negative absorption peak. A Ca(II) solution with the same concentration (0.5 M) as that used for Cu(II) was prepared and confirmed to be fully dissolved. Using the identical dipping-dipping method and a 2 wt% CMC solution, CMC-Ca(II) samples were fabricated. After 20 cycles, slight turbidity began to appear in the CMC solution; after 200 cycles, the turbidity became more pronounced (Supplementary Fig. 14). This progressive increase in turbidity is attributed to the cumulative residual CMC in the Ca(II) solution, which enhances the weak coordination interaction between Ca(II) and CMC over repeated operations. These results provide preliminary evidence supporting the assembly ability order Cu(II) > Fe(II) > Ca(II), which is also in accordance with the oxidizing ability order of these ions (i.e., Cu(II) has the strongest tendency to be reduced). In addition, the UV peak position and absorbance of CMC-Cu(II) remain unchanged with different reaction time (Fig. 2c). In contrast, the absorbance of the CMC-Fe(II) peak increases with prolonged reaction time, accompanied by a slight redshift, suggesting that Fe(II) forms a weak complex with CMC and requiring more time to complete coordination. (Fig. 2d, Supplementary Fig. 15). Meanwhile, CMC-Ca(II) displays no UV absorption peak (Fig. 2e). These findings further demonstrate that Cu(II) possesses a more stable coordination interaction and molecular assembly capacity, as expected. Furthermore, Ag(I), with a higher oxidation potential than Cu(II), forms an ultra-thin freestanding CMC-Ag(I) membrane and exhibits an additional UV absorption peak (Supplementary Fig. 16). These results strongly support the aforementioned trend.

We previously indicated that Na(I) substituents in the CMC molecule can be occupied by coordination positions of other metal ions with higher oxidation states. In Fig. 2f, the intensity of Na elements increases as follows: CMC-Cu(II) < CMC-Fe(II) < CMC-Ca(II) < CMC, according to their ability to replace Na(I). Notably, no Na peaks are observed in CMC-Cu(II). These results confirm the consistent molecular assembly ability of these three metal ions based on their coordination energies.

More attractive, we experimentally discovered that other biomacromolecules, such as MC and CMCH, also demonstrate molecular assembly behavior triggered by metal ion coordination (see relevant UV spectra in Supplementary Fig. 17). Moreover, the membrane-forming behavior of these biomacromolecules with three metal ions (Biomacromolecule-metal ions) aligns with the CMC (Fig. 2g). Similarly, Ag(I) forms membranes (CMC-Ag(I), MC-Ag(I), and CMCH-Ag(I)) with performance comparable to Cu(II) (Supplementary Fig. 18). This expanded ultra-fast molecular assembly strategy will facilitate the reliable generation of bio-conductive membranes from various sustainable biomass resources.

### Mechanism characterization and simulation of molecular assembly

**Fig. 3:**
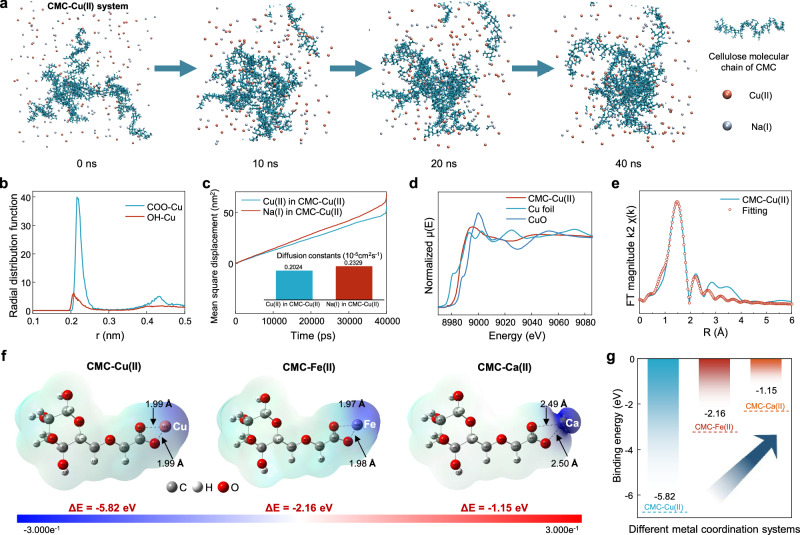
Mechanism characterization and simulation of molecular assembly induced by coordination interactions. **a** Snapshots of the Cu(II)-induced assembly of CMC chains at 0, 10, 20, and 40 ns simulated using the GROMACS package. The teal chains represent the cellulose molecular chains of CMC; the reddish-brown spheres denote Cu(II) ions, and the light gray spheres denote Na(I) ions. **b** Radial distribution function of Cu(II) around CMC-Cu(II) system. **c** Mean square displacement of Cu(II) and Na(I) in CMC-Cu(II) system. **d** Cu K-edge XANES spectra of the CMC-Cu(II), CuO, and Cu foil; spectra of CuO and Cu foil standards are shown for comparison purposes. **e** R-space EXAFS spectrum of the CMC-Cu(II) and the corresponding fitting curve. FT, Fourier transform. **f** The coordination structures and electrostatic views of CMC-Cu(II), CMC-Fe(II), and CMC-Ca(II) obtained by DFT simulation. Color scale ranges from −0.300 (red) to 0.300 (blue) in atomic units (a.u.). **g** The binding energies of CMC-Cu(II), CMC-Fe(II), and CMC-Ca(II) obtained by DFT calculation.

To investigate the influence of Cu(II)’s strong coordination on molecular chain dynamics and ion transport pathways at the molecular level, molecular dynamics (MD) simulations were conducted to analyse the structural evolution and ionic transport behaviour of molecular chains in the CMC-Cu(II) system (Supplementary Table 1). As shown in Fig. 3a, the simulation snapshot results reveal that the CMC molecules initially dispersed in an aqueous environment and rapidly aggregated within 40 ns under the strong coordination of Cu(II), thereby completing molecular assembly and forming a solid-state thin membrane. Furthermore, the interactions between the carboxylate (-COO^−^) and hydroxyl (-OH) groups in the CMC molecular chain and Cu(II) were quantitatively analysed using the radial distribution function (RDF). The results indicate a stronger preference of Cu(II) for coordination with -COO^−^ groups than with -OH groups, as evidenced by the higher peak intensity in the RDF (Fig. 3b). This suggests that CMC molecule assembly is primarily driven by the coordination between Cu(II) and -COO^−^ groups along the polymer chain. Moreover, the migration behaviour of Na(I) and Cu(II) along the CMC chain was evaluated by calculating their diffusion coefficients. The calculated diffusion coefficients are 0.20 × 10^−5 ^cm^2^·s^−1^ for Cu(II) and 0.23 × 10^−5 ^cm^2^·s^−1^ for Na(I) (Fig. 3c inset), confirming the faster diffusion of Na(I). Additionally, the simulated mean square displacements of Cu(II) and Na(I) in the CMC-Cu(II) system are 55.40 nm² and 68.36 nm², respectively (Fig. 3c), indicating higher mobility of Na(I). This supports the effectiveness of the ionic conduction mechanism in the CMC-Cu(II) system. Collectively, these MD simulations validate the feasibility of the proposed strategy, which utilises strong Cu(II) coordination to induce ultra-fast molecular assembly and form ultra-thin conductive bio-membranes. This approach offers significant practical advantages for on-demand applications in fields such as biomedicine and wearable electronics.

To further elucidate the detailed structural characteristics and transient assembly mechanism of metal ion coordination membranes, X-ray absorption spectroscopy (XAS) was employed to analyse the Cu valence and coordination state in the formed CMC-Cu(II) membrane complex. Furthermore, density functional theory (DFT) calculations were conducted to determine the most stable coordination structures. The Cu K-edge X-ray absorption near-edge spectrum (XANES; Fig. 3d) indicates that the electronic state of Cu ions in CMC-Cu(II) is close to that of CuO, showing characteristic absorption peaks of Cu(II). This confirms that Cu in CMC-Cu(II) remains in the +2 state without any chemical state alteration. Fitting of the XANES and extended X-ray absorption fine structure (EXAFS; Fig. 3e and Supplementary Fig. 19) spectra reveals two Cu-O distances of 2.14 ± 0.01 Å and 2.00 ± 0.01 Å (Supplementary Table 2). These distances correspond to the coordination of Cu(II) with the two oxygen atoms of the carboxyl groups in CMC. Additionally, the coordination numbers of copper in CMC-Cu(II) are 2.2 ± 0.6 and 1.8 ± 0.7, respectively (Supplementary Table 2), giving a total coordination number of approximately 4. This suggests that the carboxyl groups of CMC form a coordination structure resembling a square-planar geometry with Cu(II) via a bidentate coordination mode.

To support and display the coordination structures and corresponding binding energies, three systems exhibiting different membrane-forming behaviors—CMC-Cu(II), CMC-Fe(II), and CMC-Ca(II)—were selected as representative examples. A single glucose unit substituted with a carboxymethyl group was employed as a model to simulate the theoretical optimal structures using DFT, with consideration of the solvent effect. Fig. 3f illustrates the coordination structure of the complexes. The Cu(II) ion forms a bidentate coordination with the two oxygen atoms of a single carboxyl group on the CMC chain (based on the result of Fig. 3b). The calculated lengths of the two Cu-O coordination bonds are both 1.99 Å, which are in good agreement with the experimental fitting results (~2.14 ± 0.01 Å and ~2.00 ± 0.01 Å). It is speculated that in the actual reaction, the Cu(II) would form bidentate coordination with two carboxyl groups from two different molecular chains, ultimately adopting a typical tetra-coordinated configuration close to a square planar geometry. This is highly consistent with the structure inferred from EXAFS analysis. In the simulated system containing a single glucose unit, Fe(II) and Ca(II) display coordination configurations similar to those of Cu(II), yet the bond lengths are distinct. The calculated Fe-O bond lengths are 1.97 Å and 1.98 Å respectively, while the calculated Ca-O bond length are 2.49 Å and 2.50 Å respectively. All these values are consistent with the conventional theoretical values.

The binding energy calculation further quantifies the strength of the coordination interaction from a thermodynamic perspective (Fig. 3g, Supplementary Table 3). The calculated binding energies of the three systems, CMC-Cu(II), CMC-Fe(II), and CMC-Ca(II), are −5.82 eV, −2.16 eV, and −1.15 eV, respectively. All these values are negative, indicating that the formation of these complexes is energetically favourable, suggesting a spontaneous tendency. Moreover, the absolute values of the binding energies follow the order: CMC-Cu(II) > CMC-Fe(II) > CMC-Ca(II). For negative binding energies, a larger absolute value indicates a more stable combination complex. Thus, CMC-Cu(II) forms the most stable coordination complex, followed by CMC-Fe(II), while CMC-Ca(II) forms the least stable one. This is consistent with the previous experimental observations and characterization results.

### High-fidelity physiological monitoring by CMC-Cu(II) membranes as epidermal electrodes

**Fig. 4:**
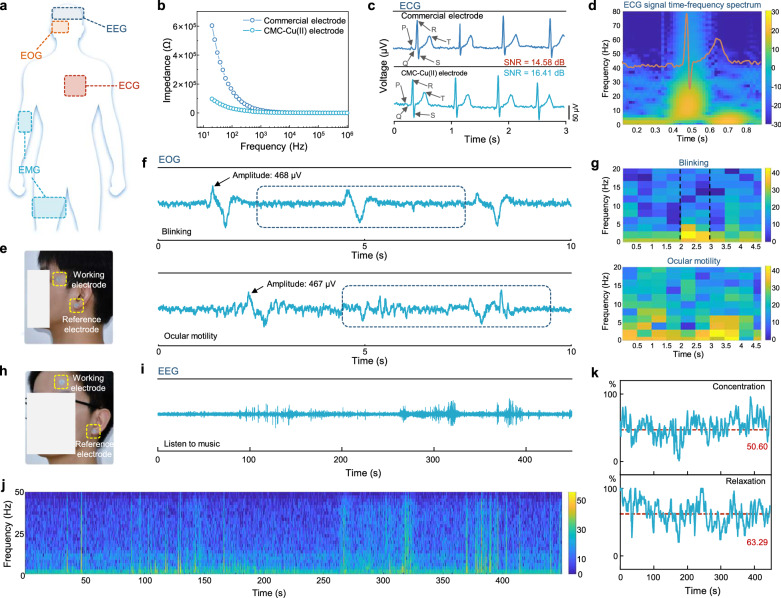
High-fidelity monitoring of ECG, EOG, and EEG by the CMC-Cu(II) epidermal electrode. **a** Schematic diagram of ECG, EMG, EOG, and EEG monitoring. **b** Skin-contact impedance values of CMC-Cu(II) and commercial electrodes within 20 Hz to 1000 kHz. **c** The periodic ECG signals and SNR of CMC-Cu(II) and commercial electrodes. **d** Time-frequency spectrum of a single cycle of ECG signal. **e** Schematic illustration of electrode placement during EOG collection. **f**, **g** Periodic EOG signals captured by the CMC-Cu(II) electrodes displaying movements involving blinking and ocular motility of the subject (**f**) and corresponding time-frequency spectrum of a single cycle of EOG signal (**g**). **h** Schematic indication of electrode placement during EEG collection. **i**–**k** EEG signals (**i**), corresponding time-frequency spectrum (**j**), and coefficients of concentration and relaxation (**k**) recorded by CMC-Cu(II) electrodes during the subject’s cognitive engagement in music listening. Color scale (blue to yellow) indicates the spectral intensity from low to high for all ECG, EOG and EEG time-frequency representations.

Physiological monitoring is crucial for continuously tracking key biological signals, such as ECG, EOG, EEG, and EMG, to assess health and enable early disease detection (Fig. 4a). For accurate monitoring, sensors must maintain conformal contact with the skin, deforming synchronously during use. Based on the stress-strain curve of the CMC-Cu(II) membrane (Supplementary Fig. 20), the fracture elongation is determined to be 98.66%, with a corresponding fracture energy (a key indicator of toughness) of 2.4 × 10⁶ J·m⁻³. These results demonstrate that the membrane possesses adequate flexibility and ductility to serve as a skin-contact electrode under dynamic strain conditions during physiological signal monitoring. Additionally, electrodes must exhibit favourable biocompatibility to avoid adverse reactions like erythema and oedema. Therefore, biocompatibility tests of the CMC-Cu(II) membrane were conducted. After 24 h culture of human umbilical vein endothelial cells (HUVECs), both the blank control and the CMC-Cu(II) membrane group exhibited good cell compatibility (Supplementary Fig. 21). These findings suggest that the CMC-Cu(II) electrode employed in this study poses negligible cytotoxic risk, supporting its safe application in wearable bioelectronics requiring prolonged skin contact. With its ultra-fast and facile in situ preparation, thin and conformal structure, favourable biocompatibility, and satisfactory ionic conductivity, the CMC-Cu(II) membrane represents an ideal epidermal electrode.

One crucial criterion for acquiring high-quality physiological signals is establishing a stable, low-impedance interface between electrodes and skin^35^. The CMC-Cu(II) electrodes demonstrate lower impedance than commercial Ag/AgCl electrodes (Fig. 4b and Supplementary Fig. 22). Specifically, compared with the corresponding commercial electrodes (6.392 × 10^5^ Ω), the impedance of the CMC-Cu(II) electrodes (9.998 × 10^4^ Ω) exhibits a notable reduction of approximately 84% at 20 Hz. Furthermore, the capacitance of CMC-Cu(II) (48.59 nF) is substantially higher than that of the commercial electrode (13.17 nF), while its resistance (173.23 Ω) is considerably lower than that of the commercial counterpart (683 Ω). Maintaining high interfacial capacitance and low resistance at the electrode-skin interface is crucial for minimizing bioelectrical signal attenuation and enhancing the overall signal-to-noise ratio (SNR)^18^. The enhanced electrical property of CMC-Cu(II) relative to commercial electrodes is attributed to its high ionic conductivity and conformal contact with the skin. Furthermore, practical applications of physiological signal monitoring frequently involve dynamic environments, such as mechanical stretching, compression, and exposure to sweat. To evaluate the functional stability of CMC-Cu(II) under such dynamic conditions, its electrical performance (impedance) was measured following repeated mechanical deformation, sweat exposure, and prolonged wear (24 h) (Supplementary Fig. 23). After stretching and compression (Supplementary Fig. 23a, insets i-iv), the impedance shows negligible changes, indicating that the membrane retained its electrical conductivity after mechanical deformation (Supplementary Fig. 23a, b). Upon exposure to sweat, an increase in capacitance and a decrease in resistance (Supplementary Fig. 23d) are observed at 20 Hz, indicating a reduction in impedance (Supplementary Fig. 23c). This behavior is likely attributed to the conductive nature of sweat and the presence of trace ions, such as Na(I), which enhance ionic mobility within the system. After 24 h of wear, a slight increase in impedance is observed. Specifically, impedance, capacitance, and resistance of the CMC-Cu(II) electrode were systematically measured every 3 h under the test condition. The impedance values at 20 Hz for the samples at 0 h, 3 h, 6 h, 9 h, 12 h, and 24 h are recorded as 9.75 × 10^4^ Ω, 9.98 × 10^4^ Ω, 9.99 × 10^4^ Ω, 1.38 × 10^5^ Ω, 1.36 × 10^5^ Ω, and 2.21 × 10^5^ Ω, respectively (Supplementary Fig. 23e, f). The impedance of the CMC-Cu(II) electrode exhibits a slight increase within the first 6 h, followed by a more substantial rise after 12 h and an even more pronounced increase by 24 h; however, it remains lower than that of the commercial electrode. This indicates that the physiological signal monitoring performance of the CMC-Cu(II) electrode decreases slightly within 6 h, shows partial attenuation within 12 h, but remains adequate for reliable conventional physiological monitoring. This is likely due to water loss from the membrane in environmental conditions.

Subsequently, circular CMC-Cu(II) electrodes (15 mm diameter) were applied in a classical three-lead ECG setup based on the “Einthoven triangle” theory. From the collected ECG signals (Fig. 4c), both commercial and CMC-Cu(II) electrodes exhibit clear and characteristic PQRST waveforms. The P wave corresponds to atrial depolarisation, representing the electrical activation preceding atrial contraction; the QRS complex reflects ventricular depolarisation, which drives cardiac pumping; and the T wave indicates ventricular repolarisation, marking the recovery of the ventricles to their resting state. These waveform components serve as fundamental parameters in the clinical assessment of cardiac rhythm and function. Compared with the commercial electrodes, the CMC-Cu(II) electrodes not only capture ECG signals with identical waveforms but also achieve a higher SNR (16.41 vs. 14.58 dB). Further time-frequency analysis^36^ enables precise interpretation of ECG signals by revealing the frequency distribution characteristics of different waveforms. The P wave is typically concentrated in the 0.5–10 Hz range, exhibiting a brief low-frequency energy peak; the QRS complex spans a broader frequency spectrum from 10 to 40 Hz, reflecting high-frequency components associated with ventricular depolarization; the T wave generally falls within the same 0.5–10 Hz range as the P wave but demonstrates higher energy density. By employing the short-time Fourier transform, time-frequency analysis simultaneously captures signal details in both temporal and spectral domains. This approach facilitates understanding of the electrophysiological behavior of a normal heart and supports the detection of abnormalities such as arrhythmias, while also providing a reliable foundation for ECG signal denoising and feature extraction. A complete cycle of the ECG signal acquired by CMC-Cu(II) is extracted, and its corresponding time-frequency spectrum (Fig. 4d) is obtained via the Fourier transform. In the resulting spectrogram, the horizontal axis represents time, reflecting the temporal evolution of the ECG signal; the vertical axis denotes frequency, indicating the distribution of signal energy across different frequency bands. The color bar reflects the signal power or energy: colors approaching deep yellow indicate higher energy levels at specific time-frequency points. This time-frequency spectrum demonstrates that the ECG signal acquired by the CMC-Cu(II) electrode is highly consistent with the characteristic features of a typical electrocardiogram, confirming its reliable performance in physiological signal detection.

The application of the CMC-Cu(II) electrode as a horizontal unipolar lead for EOG monitorin is demonstrated in Fig. 4e. A single CMC-Cu(II) electrode placed at the outer corner of the eye captures periodic EOG signals, reflecting blinking and ocular motility^37^. For blinking, the CMC-Cu(II) electrode captures distinct voltage patterns for each blink event, with localized amplification illustrating precise signal characteristics (Fig. 4f top). The time-frequency spectrum derived from the Fourier transform of the EOG signal reveals detailed signal characteristics, thereby further substantiating its stability and accuracy (Fig. 4g top). Similarly, during ocular motility tests, the CMC-Cu(II) records the persistent pattern of EOG signals corresponding to eye movements, which is distinctly difference from the intermittent signal associated with blinking. Localized amplification and time-frequency spectrum analysis further reveal detailed signal patterns (Fig. 4f bottom and Fig. 4g bottom). These results align with the patterns observed in the subjects’ blinking and ocular motility behavior recorded using commercial electrodes (Supplementary Fig. 24). Based on the signal amplitude, the peak monitored by the CMC-Cu(II) electrode is slightly higher than that of the commercial electrode, demonstrating its capability to capture high-fidelity ocular signals.

Unipolar lead EEG collection offers a simple and efficient approach for analyzing the electrical activity of specific brain regions. EEG signals can be categorised into distinct frequency band, each corresponding to particular physiological and cognitive states. The δ wave (0.5–4 Hz) is predominantly observed during deep sleep and reflects the brain’s restorative and recovery processes. The θ wave (4–8 Hz) is associated with light sleep, relaxation, or meditative states and is often enhanced during certain cognitive processing and memory-related tasks. The α wave (8–13 Hz) is most prominent during a relaxed, awake state with eyes closed, indicating cortical inhibition and relaxation; it diminishes significantly upon eye opening or when attention is actively engaged. The β wave (13–30 Hz) is linked to active thinking, concentration, anxiety, and arousal, reflecting the brain’s engagement in information processing and task execution. The γ wave (>30 Hz) is closely associated with higher-order cognitive functions, including learning, perception, and multisensory integration, and becomes particularly evident in tasks requiring rapid coordination across distributed brain regions. Analysing these frequency bands enables the characterization of dynamic brain activity across various cognitive states and behavioural tasks, offering critical insights for neuroscience research and practical applications such as brain-computer interfaces. A CMC-Cu(II) electrode attached to a volunteer’s forehead (Fig. 4h) enables detection of prefrontal cortex activity by measuring potential differences between the recording and reference electrodes. The prefrontal cortex is associated with decision-making, attention, executive function, and other cognitive processes.

When the subjects listen to music, brain activity elicited by real-time stimuli from musical elements—such as sound, lyrics, and melody—is captured as electrical signal by the CMC-Cu(II) electrode (Fig. 4i). These acquired signals are subsequently transformed into time-frequency spectra via Fourier transformation (Fig. 4j). The resulting energy density distribution (represented by color intensity) across frequency bands offers a detailed representation of the subjects’ cognitive processes during each time interval. In contrast, EEG signals recorded by CMC-Cu(II) during the participants’ mathematical calculation tasks (Supplementary Fig. 25a) and their corresponding time-frequency spectra (Supplementary Fig. 25b) exhibit distinct patterns of frequency band energy distribution compared to those observed during music listening. This difference confirms that the CMC-Cu(II) electrode effectively captures the brain’s real-time neural responses to varying external stimuli, thereby reflecting the dynamic interplay between the brain and its environment. Additionally, the EEG signal collection and analysis system provides concentration and relaxation metrics across different tasks, which also show significant variation between tasks. Music listening activity shows averag concentration and relaxation coefficients of 50.60 and 63.29, respectively (range 0–100) (Fig. 4k), while mathematical calculation yields 57.60 for concentration and 44.87 for relaxation (Supplementary Fig. 25c), which aligns with the expected trend. A 20-second localized result further illustrates the stability and precision of CMC-Cu(II) recordings (Supplementary Fig. 26a and 25d), with the time-frequency spectrum displaying well-defined frequency bands (Supplementary Fig. 26b and 25e)^38,39^. These results demonstrate the accuracy and practical application potential of CMC-Cu(II) as an electrophysiological sensor.

**Fig. 5:**
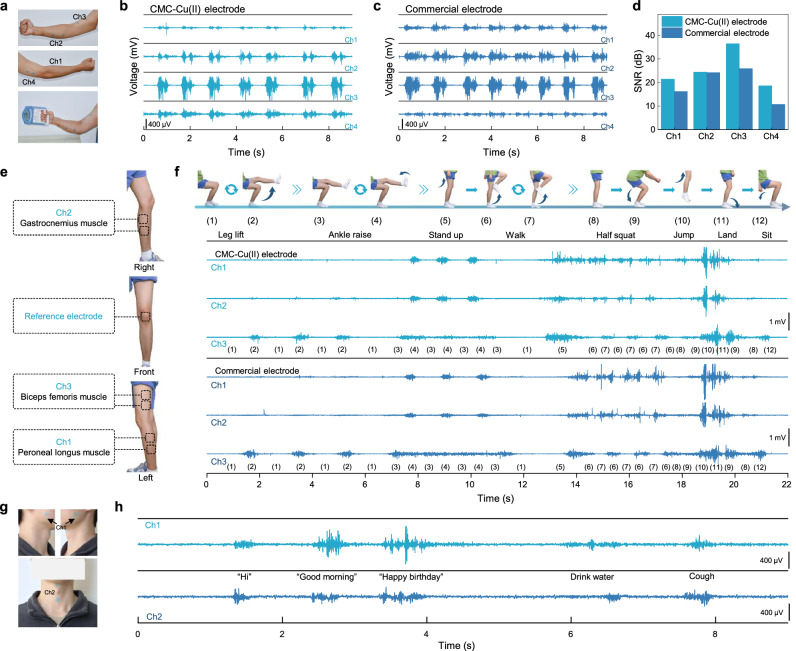
High-fidelity EMG monitoring of multi-channel and fine-scale movements by the CMC-Cu(II) epidermal electrode. **a** Schematic illustration of electrode placement on different arm muscles during EMG collection. **b-c** The four-channel periodic EMG signals of CMC-Cu(II) (**b**) and commercial electrodes (**c**) when the subject uses the grip dynamometer. **d** SNR comparisons of EMG signals collected by CMC-Cu(II) and commercial electrodes in each channel. **e** Schematic illustration of electrode placement on different leg muscles during EMG collection. **f** A series of leg movements during the acquisition of leg EMG signals (top); EMG signals corresponding to the series of leg movements of the subject captured by the CMC-Cu(II) and commercial electrodes (bottom). **g** Schematic illustration of electrodes placed on both buccal masseters and throat to collect micro-signals. **h** EMG signals when the subject speaks some common words and makes other oral movements recorded by CMC-Cu(II) and commercial electrodes.

Further, we employed CMC-Cu(II) for multi-channel and fine-scale myoelectrophysiological monitoring. Pairs of circular CMC-Cu(II) electrodes, corresponding to four lead channels, were fabricated in situ on the extensor carpi radialis longus, extensor carpi ulnaris, biceps brachii, and triceps brachii muscles, respectively (Fig. 5a), with commercial electrodes as controls. During grip dynamometer tests, different muscle groups exhibit varying contractions for the same gripping action due to differences in force application, leading to distinct EMG signal amplitudes and pattern. CMC-Cu(II) collects EMG signals that clearly distinguish muscle contractions across channels (Fig. 5b), similar to commercial electrodes (Fig. 5c). This demonstrates the ability of CMC-Cu(II) to precisely detect varying degrees of muscle activity. The SNR of CMC-Cu(II) signals (21.39 dB, 24.36 dB, 36.55 dB, and 18.63 dB) surpasses that of commercial electrodes (16.18 dB, 24.27 dB, 25.96 dB, and 10.72 dB) at the corresponding site (Fig. 5d), demonstrating that CMC-Cu(II) exhibits better performance in acquiring high-quality EMG signals compared to commercial electrode. Similar outcomes are observed during fist clenches (Supplementary Fig. 27).

To monitor EMG signals of human legs, pairs of CMC-Cu(II) electrodes were positioned on the gastrocnemius, peroneal longus, and biceps femoris muscles, corresponding to three lead channels (Fig. 5e), with commercial electrodes as controls. During various leg activities, such as leg lift, ankle raise, standing, walking, half-squatting, jumping, landing, and sitting (Fig. 5f top), the CMC-Cu(II) electrodes record physiological signals consistent with those from commercial electrodes. Specifically, distinct waveforms and amplitudes in Fig. 5f bottom reveal varying muscle contraction levels during different movements. The precise identification of different muscle contractions and accurate transmission of electrical signals by the CMC-Cu(II) electrodes are confirmed again during human leg movements.

For recognizing and transmitting electrophysiological signals during extensive motion, we further employed the CMC-Cu(II) electrodes for sensing micro-signals. Two CMC-Cu(II) electrodes were positioned on the masseter muscle of both cheeks and the throat (Fig. 5g), enabling the recording of mouth-related movements. Notably, when phrases such as ‘Hi’, ‘Good morning’, and ‘Happy birthday’ are spoken, the CMC-Cu(II) electrodes record high-fidelity EMG signals matching both the durations and intensities of muscle contractions. Similarly, actions such as coughing or drinking water produce consistent high-quality signals. Moreover, for distinct mouth-related activities, the two channels exhibit varying EMG signals (Fig. 5h). These findings demonstrate the ability of CMC-Cu(II) to collect and transmit electrophysiological signals with high precision, highlighting its potential as an epidermal electrode for monitoring human electrophysiological activity in practical applications.

## Discussion

In summary, inspired by the milk skin effect and the principle of molecular self-assembly, we have proposed a straightforward and time-efficient strategy for rapid fabrication of ultra-thin electronic bio-membranes via robust metal coordination, achieving in situ and conformal formation of bio-skin. By employing a dipping-dipping process, first in a CMC solution and subsequently in a Cu(II) solution, a well-formed conductive biological skin can be instantaneously formed on the surface of any target substrate within seconds. This bioelectronic skin demonstrates favorable interfacial conformal contact, degradability, and low impedance. The capacity and efficiency of membrane formation, which rely on metal ion coordination ability, follow the order: Cu(II) > Fe(II) > Ca(II). Under the same experimental conditions, CMC-Cu(II) and CMC-Ag(I) form complete and stable membranes, while CMC-Fe(II) shows incomplete formation, and CMC-Ca(II) and CMC-Mg(II) remain unchanged in solution. This trend is also evident in other biological macromolecules, such as MC and CMCH, further highlighting the generalization of the approach. The merits of this membrane include: (1) It is made from sustainable cellulose, the most abundant natural material on Earth, and is biodegradable within 9 days post-use; (2) The formation process is straightforward and time-efficient, requiring only 2 seconds; (3) The bio-skin’s ultra-thin thickness (3.4 μm) ensures highly conformal contact with any substrate and achieves low impedance, with a 84% reduction compared to commercial electrodes at 20 Hz; (4) It is formed directly on the target substrate, eliminating the need for additional transfers of the soft membrane. These attributes make the bio-skin a promising candidate for epidermal applications. Its performance has been demonstrated for diverse electrophysiological signal monitoring, showcasing its potential for developing sustainable bioelectronic across various applications. It should be noted that, while the CMC-Cu(II) membrane shows favorable in vitro biocompatibility and conformal properties for epidermal applications, the 24 h cytotoxicity results only confirm safety within conventional monitoring durations. Future work, including skin irritation and sensitisation assessments (e.g., in accordance with ISO 10993 standards), is needed to evaluate long-term safety. Hence, this work provides a preliminary validation, with full regulatory assessment beyond its scope.

## Methods

### Materials

Carboxymethylcellulose (CMC) with a viscosity range of 1000–1400 mPa·s was purchased from Shanghai Aladdin Biochemical Technology Co., Ltd. The substitution degrees of the carboxymethyl groups are 16% for the protonated form (-COOH) and 64% for the sodium salt form (-COONa), giving a total substitution degree of 80%. Copper chloride (CuCl_2_, AR, 99%) was purchased from Tianjin Damao Chemical Reagent Factory. Ferrous chloride tetrahydrate (FeCl_2_·4H_2_O, AR, 98%), calcium chloride (CaCl_2_, AR, 96%), methylcellulose (MC, 450 mPa·s), and carboxymethyl chitosan (CMCH) were obtained from Shanghai Macklin Biochemical Co., Ltd. Silver nitrate (AgNO_3_, AR) was acquired from Xilong Scientific Co., Ltd. The HUVECs were obtained from Haling Biotechnology Co., Ltd. (Shanghai, China).

### Assembly of CMC-Cu(II) membrane

The CMC-Cu(II) membrane was synthesized through a sequential dipping-dipping process using CMC and CuCl_2_ solutions. Specifically, a thin layer of CMC solution was initially dipped or coated onto the target substrate surface, followed by dipping into a CuCl_2_ solution after 1 s to facilitate the instantaneous coordination assembly of the CMC-Cu(II) membrane. Unless otherwise specified, the concentrations used for all CMC-Cu(II) membranes in this study were 2 wt% for the CMC solution and 0.5 M for the CuCl_2_ solution.

### Preparation of patterned CMC-Cu(II) membranes and microcircuits

The patterned CMC-Cu(II) membranes were prepared using a laser-cut polyamide mask to define the desired pattern on the substrate. The mask was first adhered to the target surface. The CMC solution was then applied onto the substrate through the patterned openings of the mask. After removing the mask, a CuCl₂ solution was either dropped or coated onto the surface to form patterned CMC-Cu(II) membranes. The CMC-Cu(II) microcircuit was fabricated using the same mask-forming method according to the designed circuit pattern. Subsequently, an LED lamp was positioned at the corresponding location on the circuit. Upon powering on, the complete CMC-Cu(II) microcircuit functioned properly.

### Preparation of CMC-Fe(II), CMC-Ca(II), CMC-Ag(I) and other biomacromolecule-metal ions

Solutions of MC and CMCH with a concentration of 2 wt% were prepared, along with solutions of FeCl_2_, CaCl_2_, and AgNO_3_ at a concentration of 0.5 M. A thin layer of CMC solution was applied onto the desired surface, followed by subsequent coating with FeCl_2_ and CaCl_2_ solutions covering the CMC layer to form CMC-Fe(II) and CMC-Ca(II). The same dipping-dipping procedure was employed to obtain MC-Cu(II), MC-Fe(II), MC-Ca(II), MC-Ag(I), CMCH-Cu(II), CMCH-Fe(II), CMCH-Ca(II), and CMCH-Ag(I).

### Characterization

The thickness of CMC-Cu(II) membranes was characterised by cross-sectional SEM using a Hitachi SU-1510 SEM. To assess the conformal properties, a CMC-Cu(II) membrane was attached onto a wavy PVC mould and then subjected to embrittlement in liquid nitrogen. A 90° peeling test and stress-strain curves were measured on the electronic universal testing machine (Instron Corporation 3369). Samples containing 0.5 wt% of CMC, MC, and CMCH were mixed with metal salt solutions (CuCl_2_, FeCl_2_, and CaCl_2_) at various concentrations. The resulting mixtures were characterised using a JOSVOK UV-5600P UV-VIS spectrophotometer with a test range of 190–500 nm. The absorption behaviour of samples in the UV-vis region was analysed by the characteristic absorption peak positions and intensities. The chemical state of the CMC-Cu(II) membrane was analysed using XPS equipment (Thermo Scientific K-Alpha, USA). Fourier transform infrared (FTIR) spectra of CMC and CMC-Cu(II) membranes were obtained using the Nicolet iS5 spectrometer (Thermo Scientific, USA), covering wavenumbers ranging from 4000–400 cm^−1^. The inductance-capacitance-resistance (LCR) meter (TH2838H, Tonghui) with a frequency range of 20 Hz–2 MHz was employed to measure the intrinsic impedance of both the CMC-Cu(II) skin-contact electrode and the commercial electrode.

Cu K-edge analysis of CMC-Cu(II) membrane was performed with Si(111) crystal monochromators at the BL11B beamline at the Shanghai Synchrotron Radiation Facility (SSRF, Shanghai, China). Before the analysis at the beamline, samples were pressed into thin sheets with a diameter of 1 cm and sealed using Kapton tape film. The spectra of X-ray absorption fine structure (XAFS) were recorded at room temperature using a 4-channel Silicon Drift Detector (SDD) Bruker 5040. K-edge extended X-ray absorption fine structure (EXAFS) spectra were recorded in transmission mode. Negligible changes in the line shape and peak position of Cu K-edge XANES spectra were observed between two scans taken for a specific sample. The XAFS spectra of these standard samples (Cu foil and CuO) were recorded in transmission mode. The spectra were processed and analyzed by the software codes Athena and Artemis.

### Biodegradability test

CMC-Cu(II) membranes and PE films were cut into 2 × 2 cm squares. The films were placed on natural soil surface, and photos were taken once a day. The normalized undegraded area was calculated as the average membrane area from two parallel samples.

### Biocompatibility test

HUVECs were seeded in Dulbecco’s modified Eagle’s medium supplemented with 10% fetal bovine serum, 100 units/mL penicillin, and 100 μg/mL streptomycin. The cells were maintained under either blank control conditions or in the presence of a CMC-Cu(II) membrane (5 mm × 5 mm) in a humidified incubator at 37 °C with 95% humidity and 5% CO₂. After 24 h, the cells were gently washed twice with phosphate-buffered saline and then stained with Calcein AM and propidium iodide for 30 min at 37 °C. Following staining, samples were immediately visualized using a confocal laser scanning microscope (FV3000, Olympus, Japan)^40^.

### Molecular dynamics simulation

A molecular dynamics simulation was conducted to investigate the ultra-fast molecular assembly behaviour driven by Cu(II) coordination force of CMC-Cu(II) system in an aqueous environment. Specifically, quantum chemistry calculations were first performed to optimize molecular geometries of CMC using the Gaussian 16 package at B3LYP/6-311 + G(d) level of theory. The atomistic force field parameters for all ions and molecules are described by the OPLS-AA, and water was taken from the SPCE model. In the CMC-Cu(II) simulation system, 10 CMC, 100 CuCl_2_, 130 Na(I), and 11112 water molecules were randomly inserted into a simulation box with x = 7.2 nm, y = 7.2 nm, and z = 7.2 nm.

Atomistic simulations were performed using the GROMACS package with cubic periodic boundary conditions. The equations for the motion of all atoms were integrated using a classic Verlet leapfrog integration algorithm with a time step of 2.0 fs. A cutoff radius of 1.4 nm was set for short-range van der Waals interactions and real-space electrostatic interactions. The particle-mesh Ewald (PME) summation method with an interpolation order of 4 and a Fourier grid spacing of 0.12 nm was employed to handle long range electrostatic interactions in reciprocal space. In all the three directions, periodic boundary conditions were imposed. Leapfrog algorithm was used to integrate the Newtonian equation of motion. The MD simulation was processed in an NPT ensemble and the simulation time was 40 ns. In NPT simulations, the pressure was maintained at 1 bar by the Berendsen barostat in an isotropic manner, and the temperature was maintained by the V-rescale thermostat at 298.15 K.

### Density functional theory calculations

CMC-Cu(II), CMC-Fe(II), and CMC-Ca(II) models, each consisting of one carboxymethyl-substituted glucose unit and one metal ion, were calculated using DFT to gain the corresponding coordination structure and binding energy. The binding energy is defined as $\triangle {E}_{X}={E}_{1{\mbox{glucose}}-M}-{E}_{1{\mbox{glucose}}}-{E}_{M}$, where *E*_1glucose_ represents the energy of one glucose unit substituted with carboxymethyl (1glucose) and *E*_*M*_ denotes the energy of corresponding metal ions (Cu(II), Fe(II), or Ca(II)). *E*_1glucose_-_*M*_ refers to the energy of the coordinated system (1glucose-Cu(II), 1glucose-Fe(II), and 1glucose-Ca(II)), and the “*X*” in *ΔE*_*X*_ corresponds to CMC-Cu(II), CMC-Fe(II), and CMC-Ca(II).

DFT calculations were carried out using the Gaussian 16 W, employing the B3LYP functional, the basic set 6–311 + G*, and the LANL2DZ as effective core potential for geometric optimisation, energy calculation, and frequency analysis. Additionally, a continuous solvation model SMD was used to simulate solvent effects on the molecules.

### Electrophysiological signal monitoring

For ECG monitoring, following the “Einthoven triangle” theory, two circular CMC-Cu(II) epidermal electrodes with a diameter of 15 mm were applied on the subject’s left and right wrists respectively. Additionally, a reference electrode was placed on the subject’s left ankle. The arrangement of commercial electrodes followed the same configuration. ECG signals were recorded using an ECG sensor equipped with an integrated ADS1292R signal acquisition converter, STM32F103C8T6 microcontroller, and Bluetooth transmission device.

The EOG signal was recorded using a horizontal unipolar lead. A CMC-Cu(II) epidermal electrode, with a diameter of 15 mm, was attached to the outer corner of the subject’s left eye. The reference electrode was positioned on the left earlobe, as depicted in Fig. 4e. The signals generated during blinking and turning eyeball movements were captured and recorded by a signal recording system equipped with an integrated bandpass filter (Neurosky TGAM EEG03, Jiangsu, China) connected to the electrodes.

EEG signals were also logged using a unipolar lead configuration. A CMC-Cu(II) epidermal electrode (F4), with a diameter of 15 mm, was placed on the subject’s clean forehead, while the reference electrode was positioned on the earlobe following the 10–20 international electrode placement system. The detailed arrangement of electrodes was illustrated in Fig. 4h. All electrodes were connected to a signal recording system equipped with an integrated bandpass filter (Neurosky TGAM EEG03, Jiangsu, China). EEG signals were detected and transmitted during mathematical calculation and music listening activities.

For EMG signal monitoring, a six-channel system including a signal monitoring microcontroller (ZTEMG-1100 PCB, Zhituo Intelligent Technology Co., Ltd., Qingdao, China), a signal input terminal (CH-50RB), and an oscilloscope (Handyscope Model HS4, TiePie engineering, China) was utilized, and all electrodes were connected to the system. Each channel consisted of a pair of working electrodes. CMC-Cu(II) working electrode pairs were formed in the extensor carpi radialis longus, extensor carpi ulnaris, biceps brachii, and triceps brachii (Fig. 5a), corresponding to four channels for capturing EMG signals from the arm. The reference electrode was positioned at the elbow joint. Subjects performed grip dynamometer exercises or made a fist while their resulting signals were recorded by the sensor. For collecting EMG signals from the legs, CMC-Cu(II) electrodes (Fig. 5e) were in situ prepared at three lead channels on the gastrocnemius, peroneus longus, and biceps femoris muscles respectively; meanwhile, a reference electrode was placed on the patella. A series of leg activities including leg lift, ankle raise, standing, walking, half-squatting, jumping, landing, and sitting were performed by subjects to elicit corresponding signals which were identified and transmitted through connected electrodes and sensors accordingly. Additionally, EMG signals generated by mouth movements of subjects were captured by sensors with two CMC-Cu(II) electrodes placed on the left and right masseter muscles and throat, respectively, with a reference electrode positioned on the earlobe (Fig. 5g). All commercial electrodes underwent acquisition following identical procedures.

Written informed consent was obtained from all volunteers participating in this study.

### Statistics and reproducibility

All experiments were repeated independently with similar results for at least three times.

### Reporting summary

Further information on research design is available in the Nature Portfolio Reporting Summary linked to this article.

## Supplementary information

Supplementary Information

Description of Additional Supplementary Files

Supplementary Movie 1

Reporting Summary

Transparent Peer Review file

## Source data

Source Data

## Data Availability

The data generated in this study are provided in the main article, Supplementary Information and Source data file. All data are available from the corresponding author upon request. Source data are provided with this paper.
