# Supplementary material for: In situ formation of adaptive electronic skin in 2 seconds enabled by metal coordination
Source: Nat Commun. 2026 May 16;17:6496. doi: 10.1038/s41467-026-73303-w (PMC13377055; doi:10.1038/s41467-026-73303-w)
Supplement: Supplementary file 1 — Supplementary Information [file 41467_2026_73303_MOESM1_ESM.pdf]

Supplementary Information

In Situ Formation of Adaptive Electronic Skin in 2 Seconds Enabled by Metal Coordination

Xiaojuan Wang,[1,2] Xiaosen Pan,[1] Junzhi Jiang,[1] Wanlong Song,[1] Xiaoqi Zhou,[1] Xu Lin,[1] Jingye Zhao,[3] Zhengjian Zhang, [1] Zhenxing Liu, [3] Xiaojun Ma,[1] Hongbin Liu,[1] Meng Gao[1,2*]

[1] State Key Laboratory of Biobased Fiber Manufacturing Technology, Tianjin University of Science and Technology, Tianjin 300457, China

[2] Institute of Applied Physics and Materials Engineering, University of Macau, Macao, SAR 999078, China

[3] School of Biotechnology, Tianjin University of Science and Technology, Tianjin 300457, China

Correspondence to: Meng Gao, E-mail: gaomeng91@gmail.com

This supplementary information file includes:

- Supplementary Figures 1-27
- Supplementary Tables 1-3
- Supplementary References 1

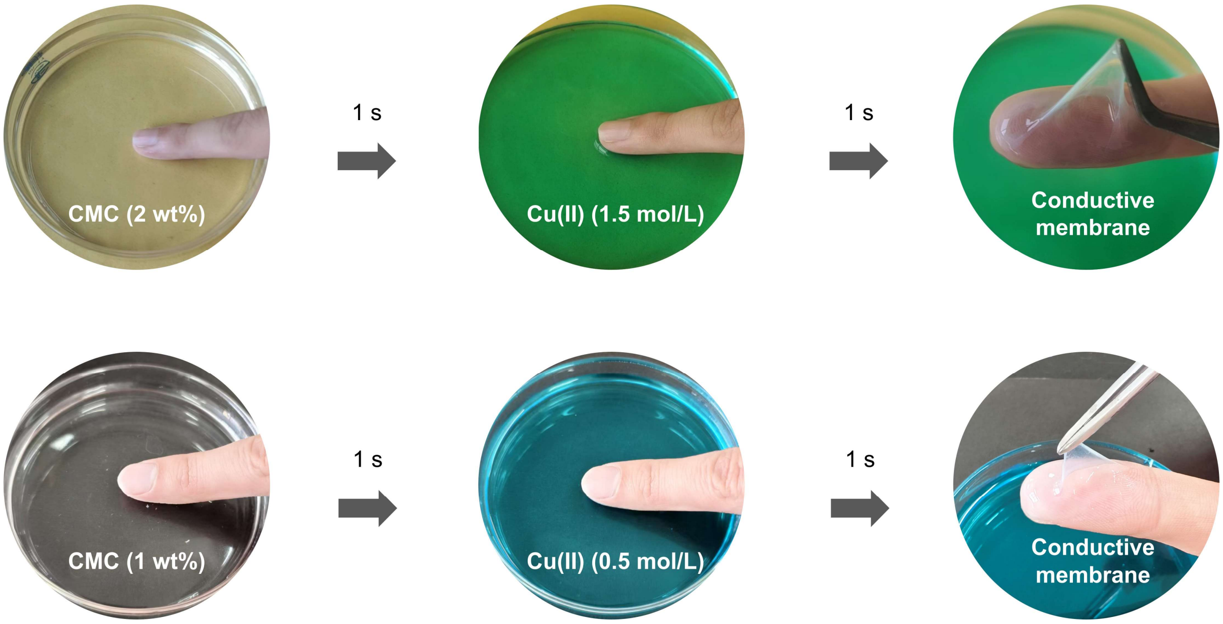

Supplementary Fig. 1. CMC-Cu(II) membranes formed at various concentrations. Digital photographs of CMC-Cu(II) membranes fabricated by the dipping-dipping coordination assembly process from CMC and Cu(II) solutions with different concentrations.

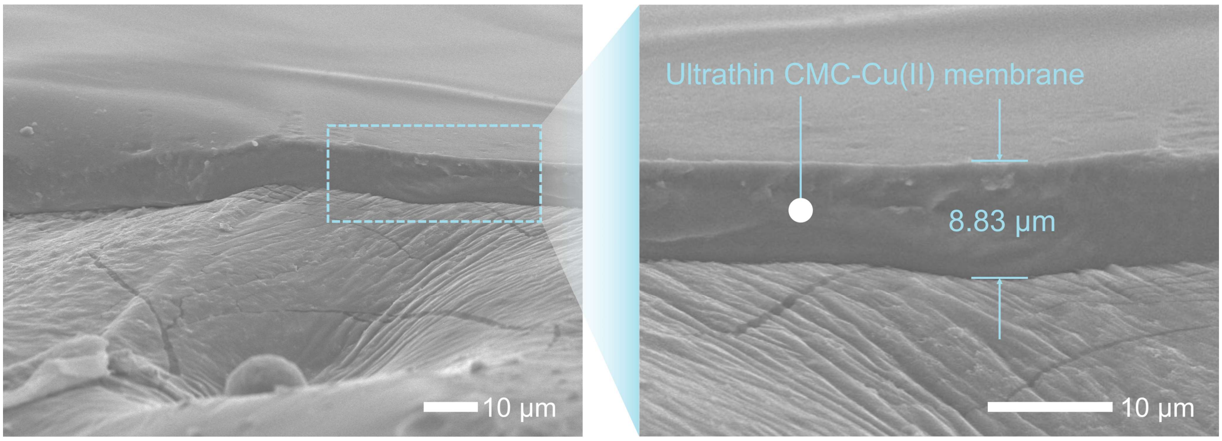

Supplementary Fig. 2. Ultrathin CMC-Cu(II) membrane. Cross-sectional SEM image of ultra-thin CMC-Cu(II) membrane. Scale bar, 10 μm.

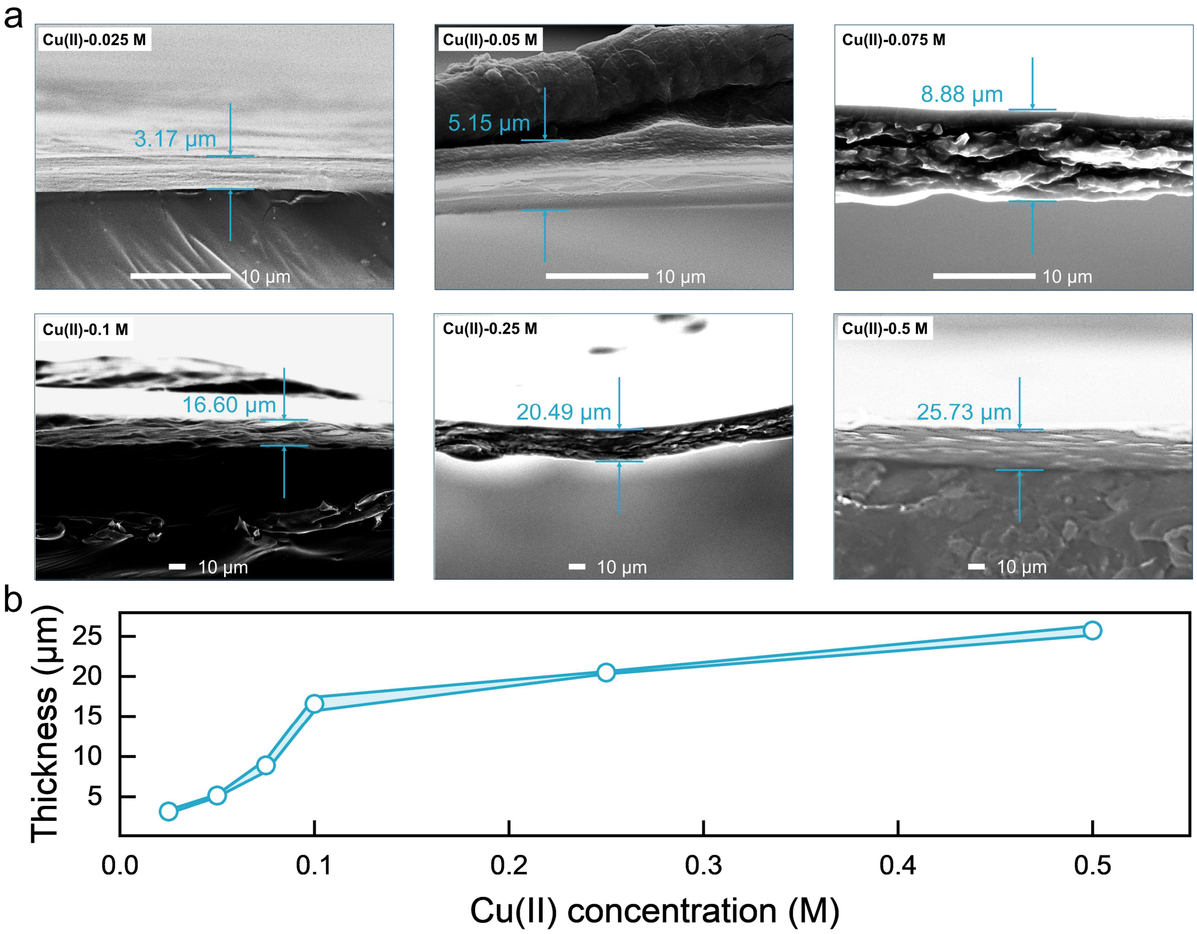

Supplementary Fig. 3. Control over the thickness of CMC-Cu(II) membranes. a Cross-sectional SEM images of CMC-Cu(II) membranes fabricated using varying concentrations of Cu(II). Scale bar, 10 μm. **b** A positive correlation between the thickness of the CMC-Cu(II) membrane and the concentration of Cu(II).

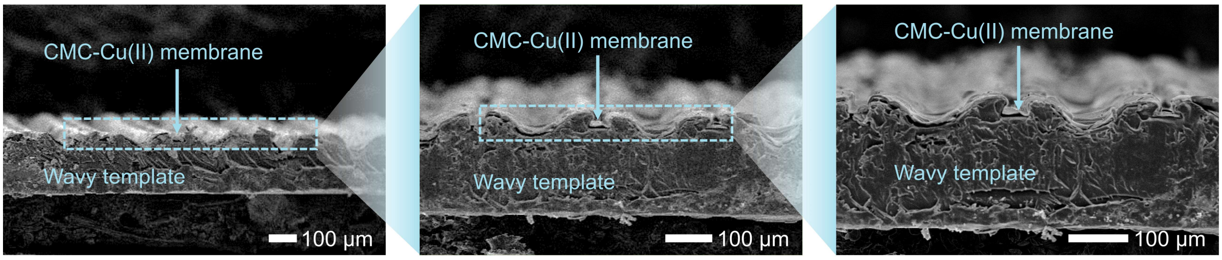

Supplementary Fig. 4. Conformal CMC-Cu(II) membrane. Cross-sectional SEM images of highly conformal CMC-Cu(II) membrane attached on polyvinyl chloride (PVC) wavy mold. Scale bar, 100 μm.

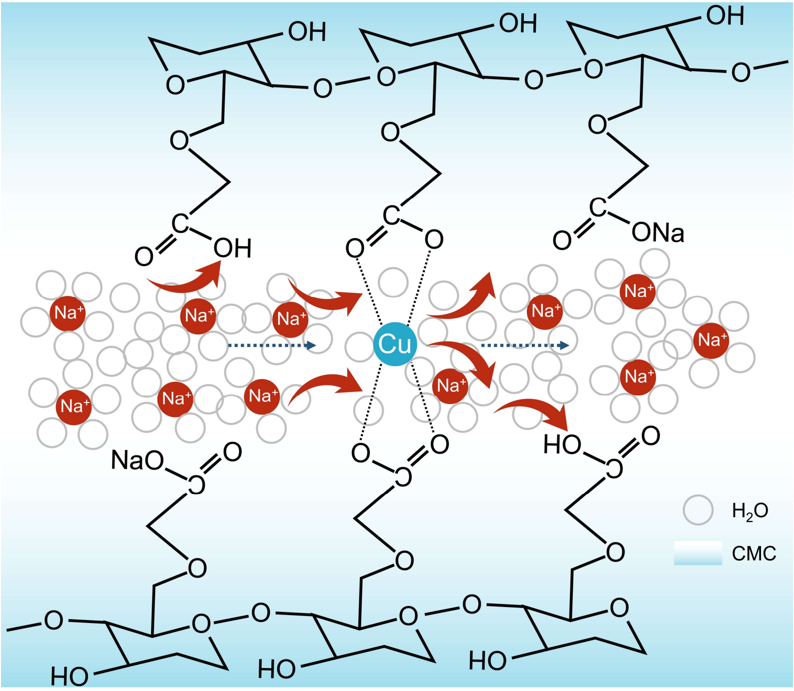

Supplementary Fig. 5. Conductivity mechanism of CMC-Cu(II) membrane. Schematic illustration of the assembly of CMC molecules into support channels induced by Cu(II) coordination bonds in CMC-Cu(II) membrane to facilitate ion transport and consequently enhance electrical conductivity. The blue dashed arrow indicates that Na(I) ions migrate together with water molecule clusters, and the red arrows indicate Na(I) and free water molecule transport via polar group sites (especially Cu(II))[1].

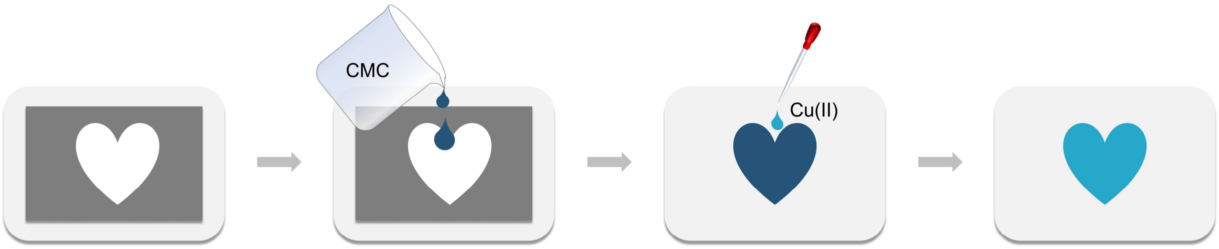

Supplementary Fig. 6. Mask-forming approach for patterned CMC-Cu(II). Schematic illustration of the process: attach mask to target surface; apply CMC solution through mask openings; remove mask; apply Cu(II) solution to form patterned CMC-Cu(II) membranes in seconds.

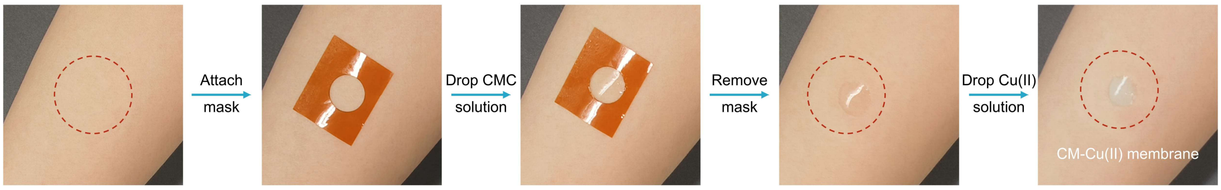

Supplementary Fig. 7. Mask-forming approach for patterned CMC-Cu(II) on skin. Schematic illustration of the steps: attach mask to skin; apply CMC through mask openings; remove mask; apply Cu(II) to form patterned CMC-Cu(II) membrane directly on skin.

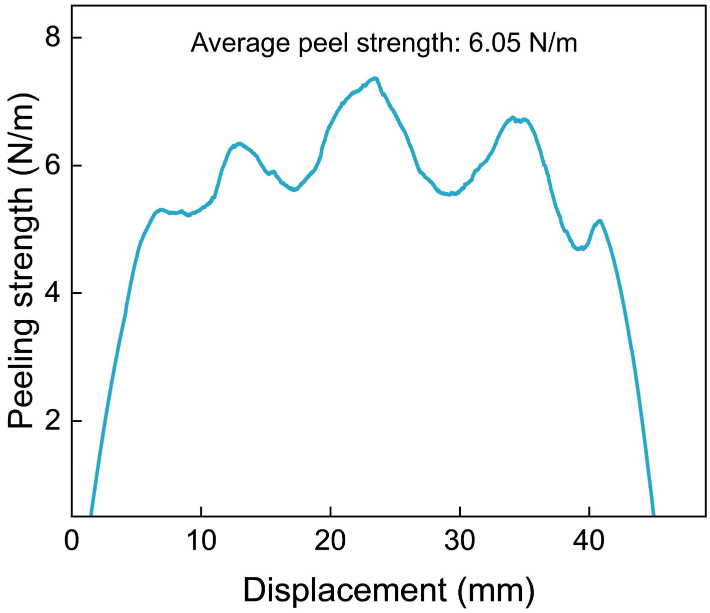

Supplementary Fig. 8. Adhesion of CMC-Cu(II) membrane. Peeling strength of the CMC-Cu(II) membrane detached from pigskin by 90° peeling test.

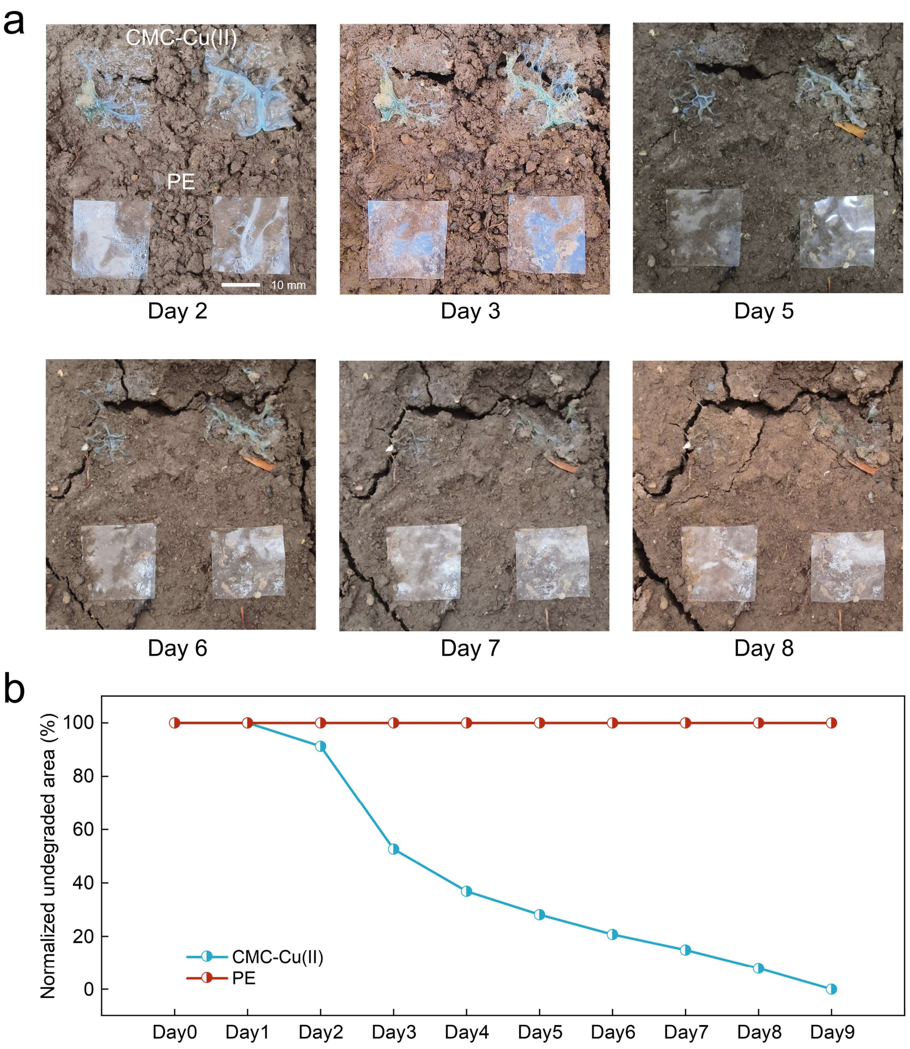

Supplementary Fig. 9. Degradation test for CMC-Cu(II) membranes and polyethylene (PE) films. a The degradation photographs of CMC-Cu(II) membranes and PE films in day 2, 3, 5, 6, 7, and 8. Scale bar, 10 mm. **b** Normalized visible undegraded area data of CMC-Cu (II) membranes and PE films over a 9-day period.

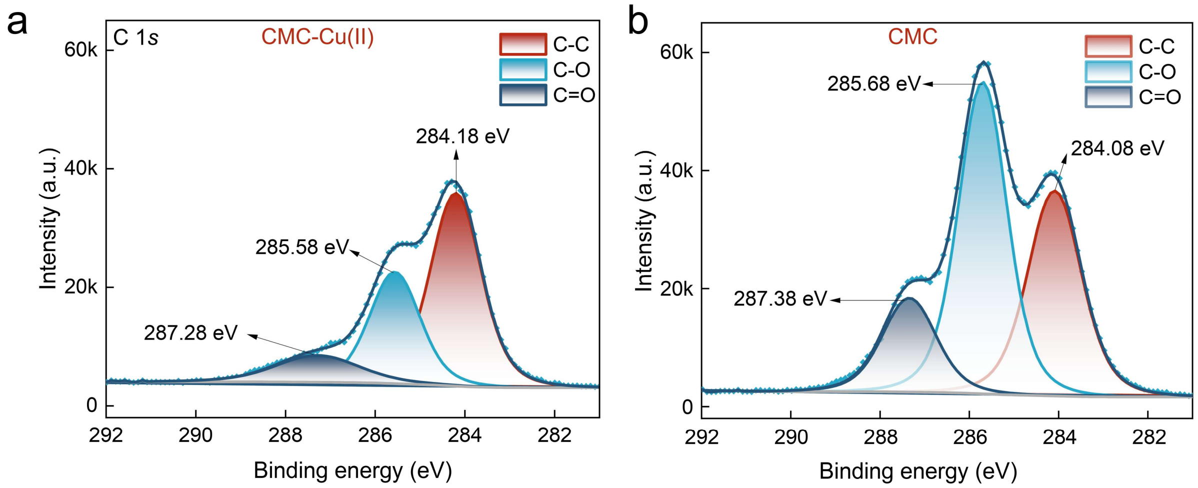

Supplementary Fig. 10. XPS C 1s spectra of CMC and CMC-Cu(II) membranes. The C 1*s* fitted curves of CMC-Cu (**a**) and CMC (II) (**b**) membranes, demonstrating a significant reduction in the C-O and C=O functional groups in the CMC-Cu(II) membrane, providing strong evidence for the robust coordination between Cu(II) and CMC.

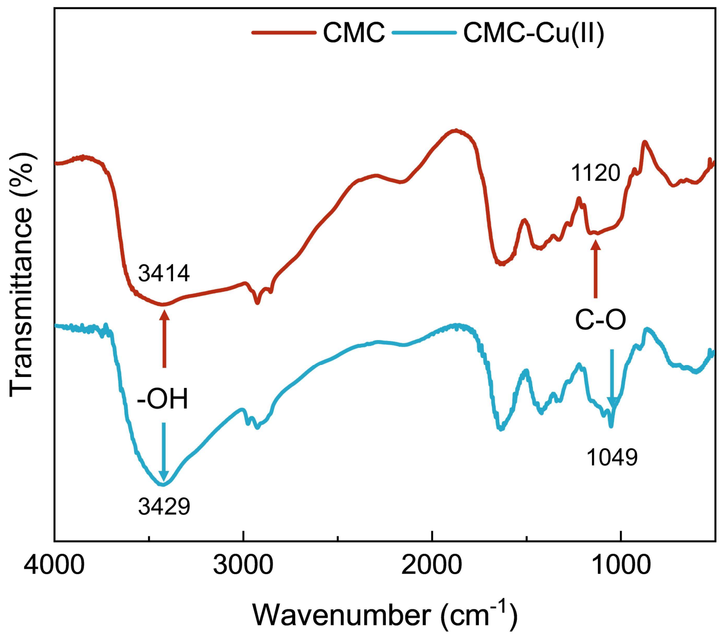

Supplementary Fig. 11. FTIR spectra of CMC and CMC-Cu(II) membranes. Compared with CMC spectrum, the sharpening of characteristic-OH peaks and the shift towards lower wave numbers in the stretching vibration peaks of C-O observed in CMC-Cu(II) spectrum provide support for the strong coordination between Cu(II) and CMC.

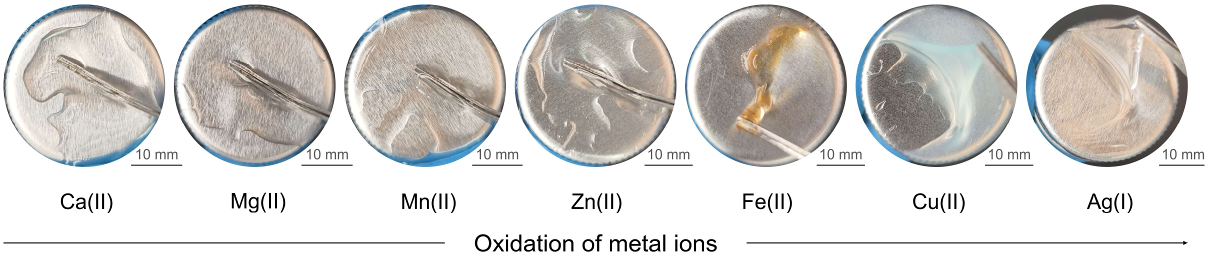

Supplementary Fig. 12. Membrane formation behavior of various metal ions. The membrane formation behavior of a series of common metal ions with varying oxidation states, including Ca(II), Mg(II), Mn(II), Zn(II), Fe(II), Cu(II) and Ag(I). Scale bar, 10 mm.

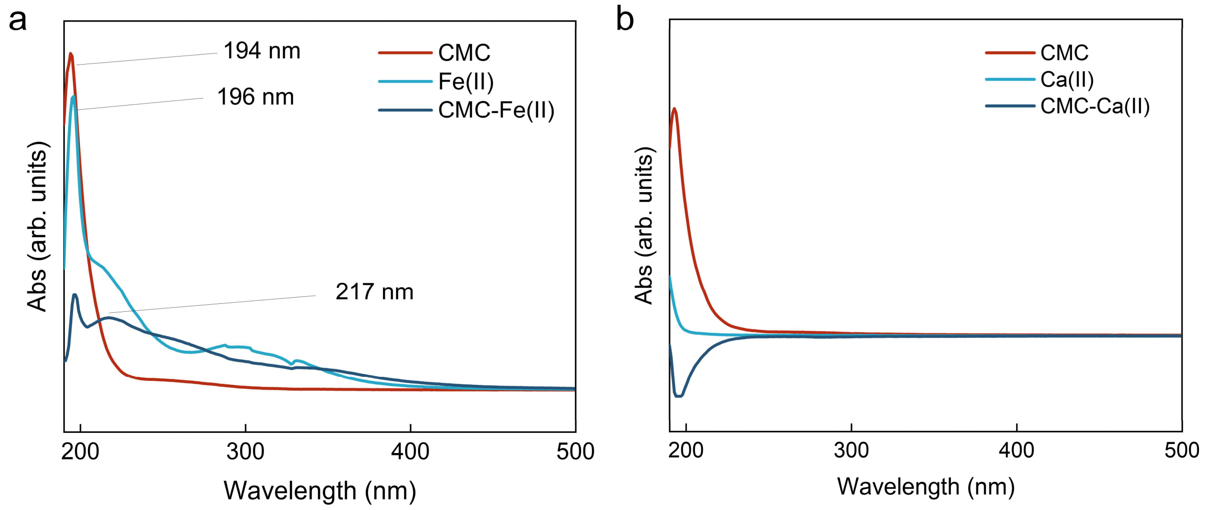

Supplementary Fig. 13. The UV absorption spectra of CMC with other metal ions. a The UV absorption spectra of CMC, Fe(II), and CMC-Fe(II), inducing a new UV absorption peak of CMC-Fe(II) in a comparison with CMC and Fe(II). **b** UV absorption curves of CMC, Ca(II), and CMC-Ca(II), exhibiting a negative UV absorption peak in a comparison with CMC and Ca(II).

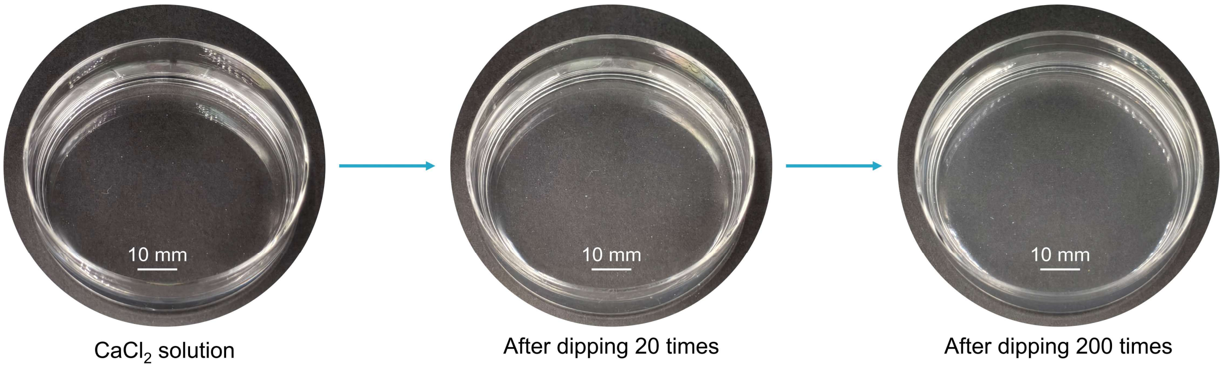

Supplementary Fig. 14. CMC-Ca(II) formation behavior over dipping-dipping cycles. Digital photos of Ca(II) solution after 20 and 200 cycles of the dipping-dipping process with 2 wt% CMC. Slight turbidity appears at 20 cycles and increases at 200 cycles, due to accumulated residual CMC enhancing weak Ca(II)-CMC coordination. No membrane forms.

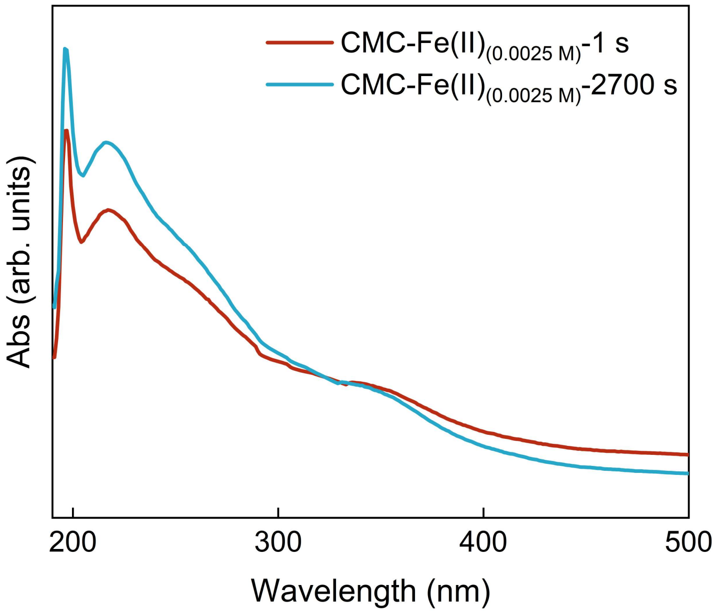

Supplementary Fig. 15. Time-dependent UV absorbance of CMC-Fe(II). The UV curves of CMC-Fe(II) with different reaction time. The absorbance of the CMC-Fe(II) peak increases with prolonged reaction time.

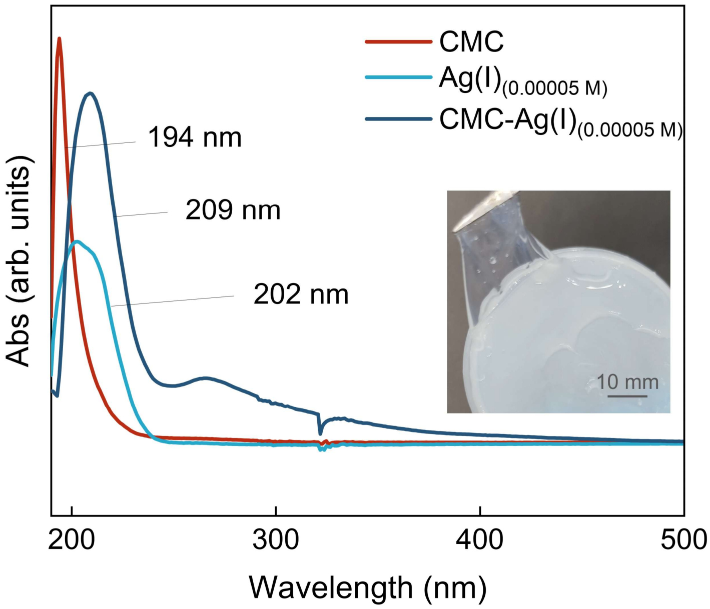

Supplementary Fig. 16. UV spectra and membrane photograph of CMC-Ag(I) coordination. The UV absorption spectra of CMC, Ag(I), and CMC-Ag(I), as well as membrane photograph of CMC-Ag(I) (inset, Scale bar, 10 mm**.**), illustrating the coordination interaction between CMC and Ag(I).

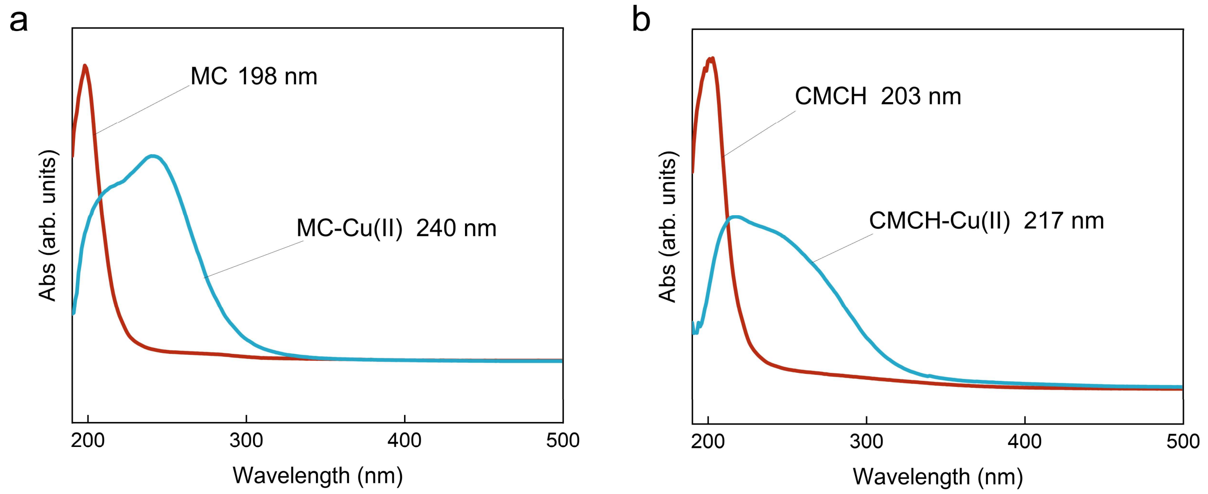

Supplementary Fig. 17. The UV absorption spectra of other biomacromolecule-metal ions. a The UV absorption spectra of MC and MC-Cu(II), inducing a new UV absorption peak of MC-Cu(II) compared with MC. **b** UV absorption curves of CMCH and CMCH-Cu(II), also exhibiting a new UV absorption peak of CMCH-Cu(II) compared with CMCH.

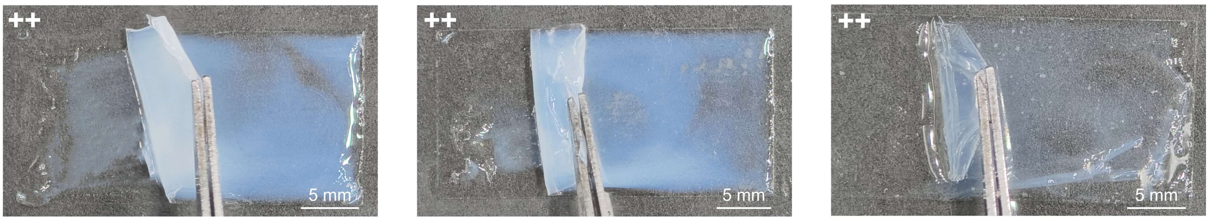

CMC-Ag(I) MC-Ag(I) CMCH-Ag(I)

Supplementary Fig. 18. Ag(I)-coordinated bio-membranes. Digital photographs of CMC-Ag(I), MC-Ag(I), and CMCH-Ag(I) membranes formed by Ag(I) coordination. "++" indicates that a complete and free-standing membrane can be formed via the dipping-dipping process. Scale bar, 5 mm**.**

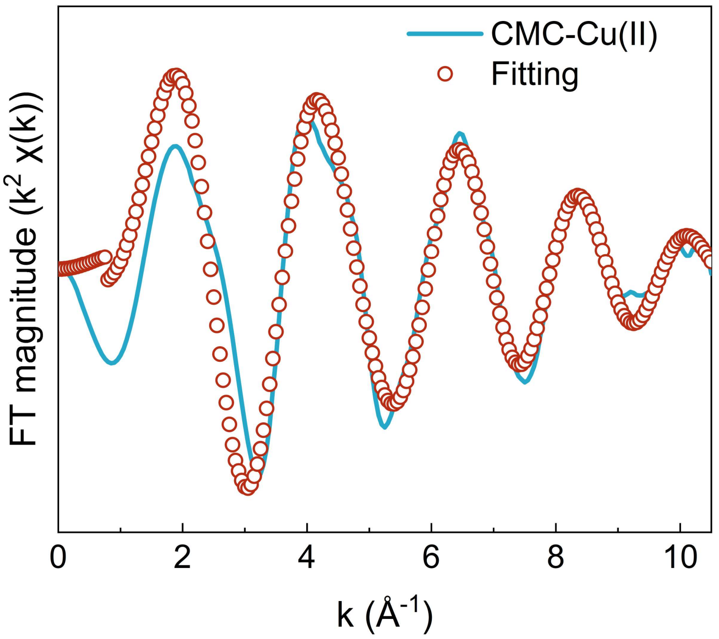

Supplementary Fig. 19. EXAFS fitting of CMC-Cu(II) in k-space. K-space EXAFS spectrum of CMC-Cu(II) (blue) and the corresponding best-fit curve (red). The fitting yields Cu–O bond distances and coordination numbers consistent with a bidentate coordination mode between Cu(II) and carboxyl groups. FT, Fourier transform.

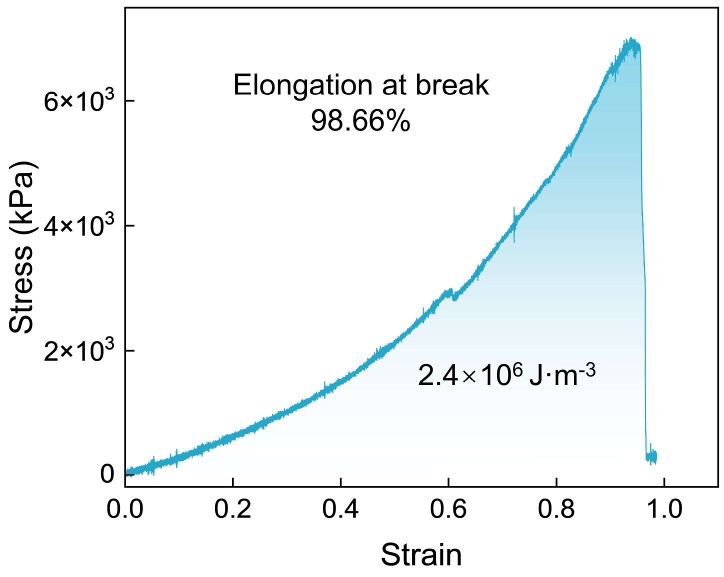

Supplementary Fig. 20. Mechanical properties of CMC-Cu(II) membrane. Stress-strain curve displaying the mechanical strength of CMC-Cu(II) membrane, along with the fracture elongation rate and fracture energy calculated from this curve.

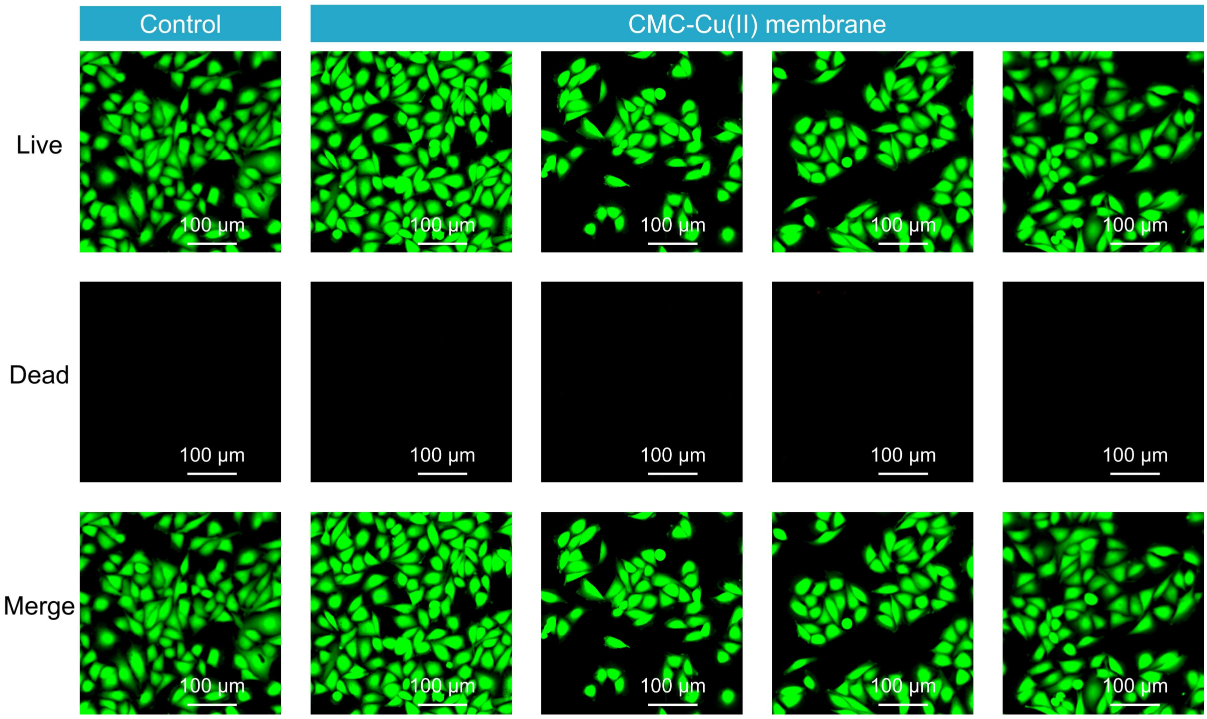

Supplementary Fig. 21. Biocompatibility evaluation of CMC-Cu(II) membrane. Representative fluorescence staining images showing live (green) and dead (red) HUVECs cells cultured without (Control) and with CMC-Cu(II) membrane (four parallel experiments). Scale bar, 100 μm

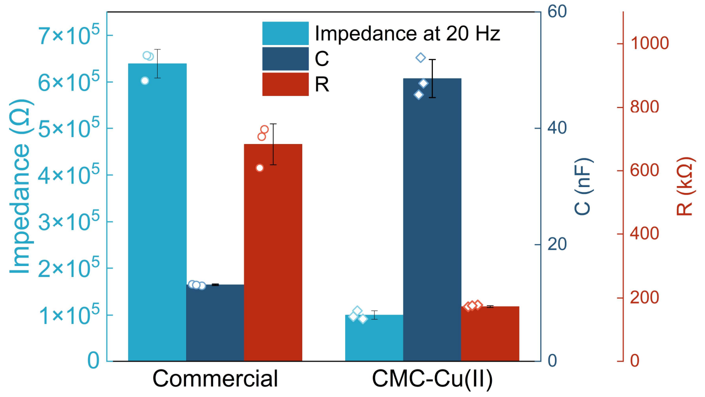

Supplementary Fig. 22. Electrode-skin interface electrical properties. Skin-contact impedance values at 20 Hz, capacitance, and resistance of CMC-Cu(II) and commercial electrodes. Data are presented as mean values ± SD with individual data points overlaid, n = 3. Circles: commercial electrodes; diamonds: CMC-Cu(II) electrodes. Blue: impedance; dark blue: capacitance (C); red: resistance (R).

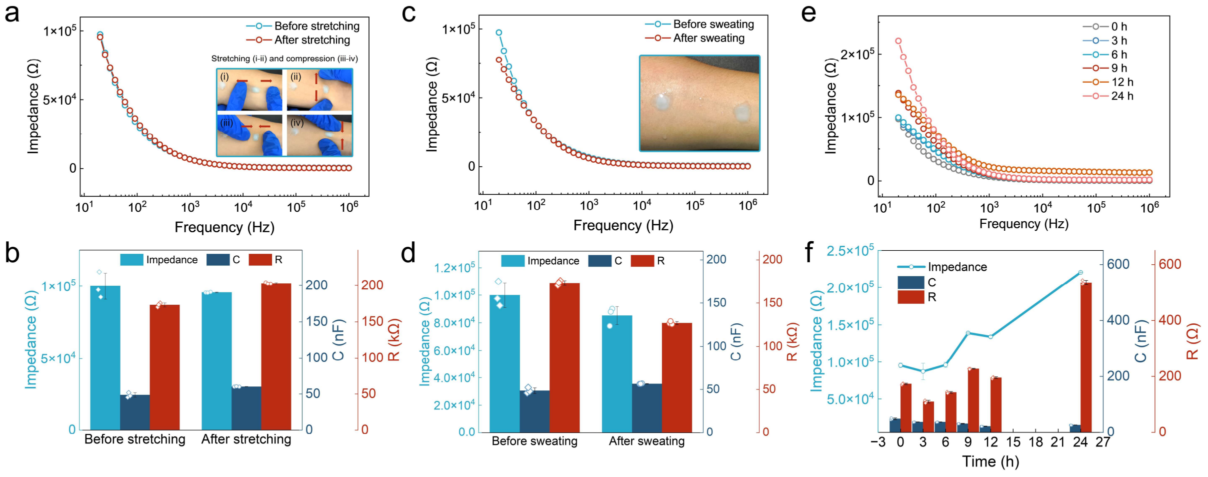

Supplementary Fig. 23. The electrical performances of CMC-Cu(II) membrane under dynamic conditions. a Skin-contact impedance values within 20 Hz to 1000 kHz of CMC-Cu(II) electrode before and after mechanical operation (stretching-compression). **b** Skin-contact impedance value at 20 Hz, capacitance, and resistance of CMC-Cu(II) electrodes before and after mechanical operation (stretching-compression). Data are presented as mean values ± SD with individual data points overlaid, n = 3. Diamonds: before stretching; circles: after stretching. Blue: impedance; dark blue: capacitance (C); red: resistance (R). **c** Skin-contact impedance values within 20 Hz to 1000 kHz of CMC-Cu(II) electrode before and after sweating. **d** Skin-contact impedance value at 20 Hz, capacitance, and resistance of CMC-Cu(II) electrodes before and after sweating. Data are presented as mean values ± SD with individual data points overlaid, n = 3. Diamonds: before sweating; circles: after sweating. Blue: impedance; dark blue: capacitance (C); red: resistance (R). **e** Skin-contact impedance values within 20 Hz to 1000 kHz of CMC-Cu(II) electrode within 24 h. **f** Skin-contact impedance value at 20 Hz, capacitance, and resistance of CMC-Cu(II) electrodes within 24 h. Data are presented as mean values ± SD with individual data points overlaid, n = 3. Dark blue circles: capacitance (C); Red diamonds: resistance (R).

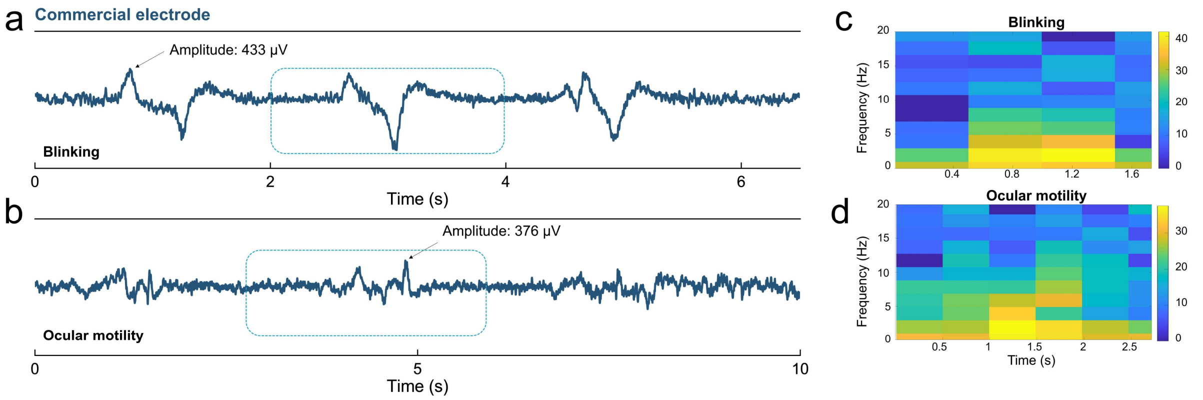

Supplementary Fig. 24. EOG performance of commercial electrodes. a-d Periodic EOG signals captured by the commercial electrodes displaying movements involving blinking (**a**) and ocular motility (**b**) of the subject and corresponding time-frequency spectrum of a single cycle of EOG signal (**c and d**). Color scale (blue to yellow) in indicates the spectral intensity from low to high for **c** and **d** time-frequency representations.

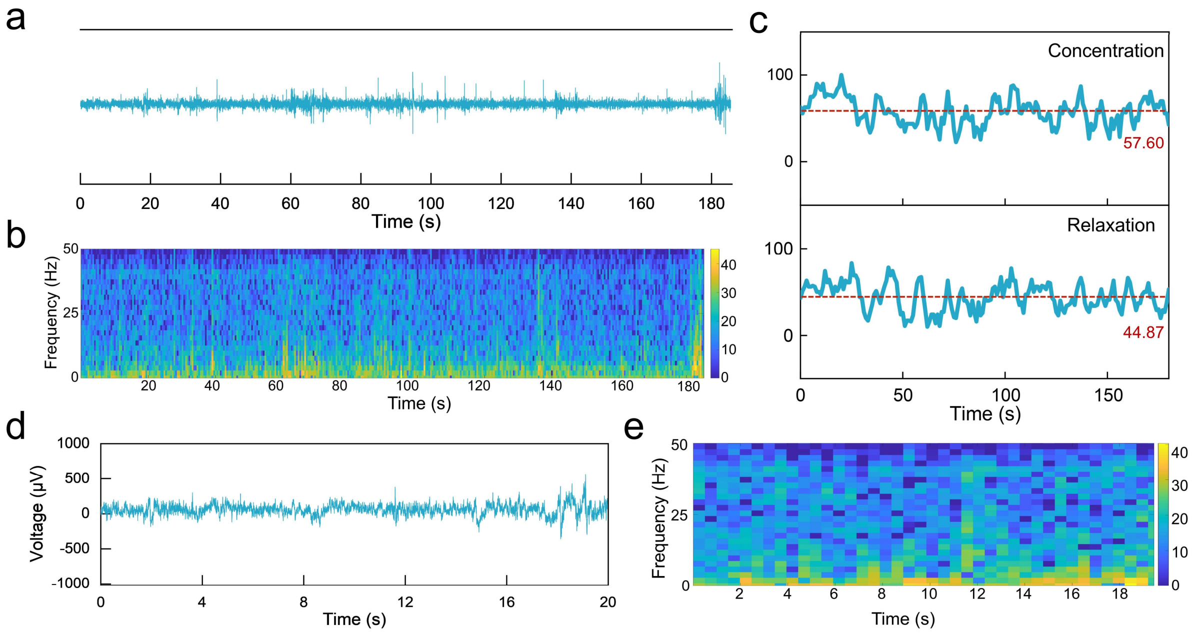

Supplementary Fig. 25. EEG monitoring during mathematical calculation by CMC-Cu(II) electrodes. a to **c** EEG signals (**a**), corresponding time-frequency spectrum (**b**), and coefficients of concentration and relaxation (**c**) during the subject's cognitive engagement in mathematical calculation. **d** to **e** Localized EEG signals over a 20-second interval (d) and the corresponding time-frequency spectrum. Color scale (blue to yellow) in indicates the spectral intensity from low to high for **b** and **e** time-frequency representations.

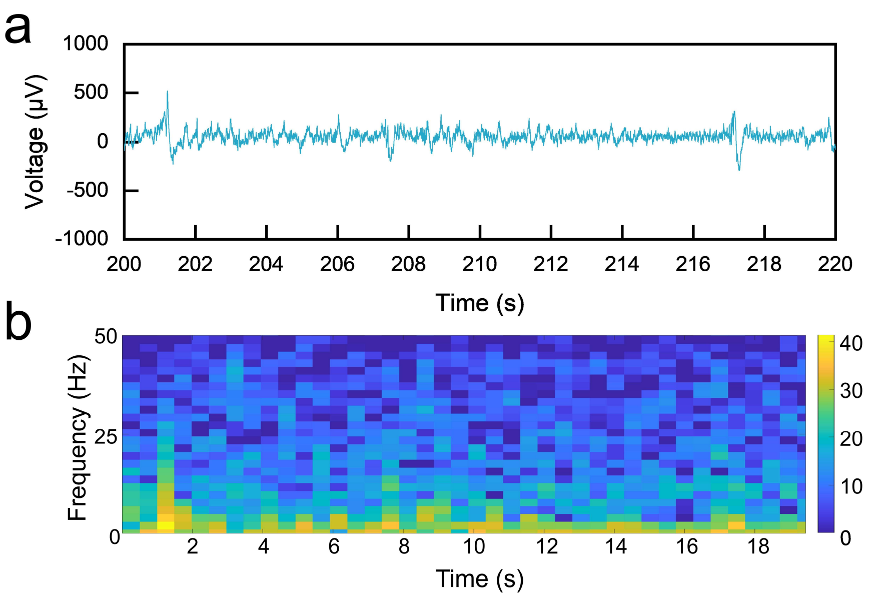

Supplementary Fig. 26. EEG monitoring during music listening by CMC-Cu(II) electrodes. **a** to **b** Localized EEG signals over a 20-second interval (**a**) and the corresponding time-frequency spectrum during the subject's cognitive engagement in music listening. Color scale (blue to yellow) indicates the spectral intensity from low to high.

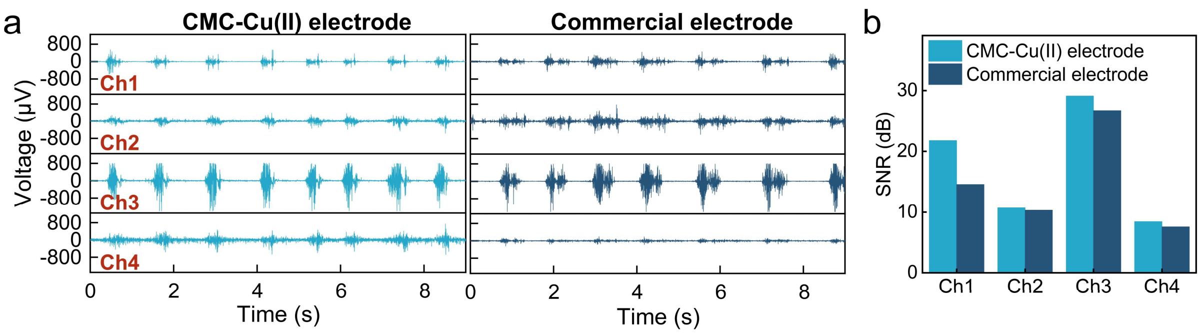

Supplementary Fig. 27. Electrophysiological monitoring of arm muscles with CMC-Cu(II) electrodes. a The periodic EMG signals in four-channel of CMC-Cu(II) and commercial electrodes when the subject makes a fist. **b** SNR comparisons of EMG signals collected by CMC-Cu(II) and commercial electrodes in each channel.

Supplementary Table 1 | Initial setup of the CMC-Cu(II) simulation system.

System	CMC chains	Cu(II)	Na(I)	Water molecules
CMC-Cu(II)	10	100	130	11112

Supplementary Table 2 | EXAFS coordination environment analysis of CMC-Cu(II).

Sample	Scattering Pair	CN	R (Å)	σ^2	ΔE_0 (eV)
CMC-Cu(II)	Cu-O	2.2±0.6	2.14±0.01	2.4±0.3	9.0±0.1
	Cu-O	1.8±0.7	2.00±0.01		

CN: coordination number, R: bond distance, σ^2: Debye-Waller factors, and ΔE_0: energy shift.

Supplementary Table 3 | The simulation and calculation for binding energies for the CMC-Cu(II), CMC-Fe(II) and CMC-Ca(II) model systems.

Molds	System	Energy (eV)	Binding energy (eV)
CMC-Cu(II)	One carboxymethyl substituted glucose units (1glucose)	-24895.49466	**-5.82**
	Cu(II)	-5322.81394872	
	1glucose-Cu(II)	-30224.12871413	
CMC-Fe(II)	1glucose	-24895.49466	**-2.16**
	Fe(II)	-3347.0237917	
	1glucose- Fe(II)	-28244.67916817	
CMC-Ca(II)	1glucose	-24895.49466	**-1.15**
	Ca(II)	-991.60084928	
	1glucose-Ca(II)	-25888.2472815	

Supplementary References

1. Wu, M., *et al.* A high-performance hydroxide exchange membrane enabled by Cu^{2+}-crosslinked chitosan. *Nat. Nanotechnol.* **17**, 629-636 (2022).
