# Supplementary material for: In situ formation of adaptive electronic skin in 2 seconds enabled by metal coordination
Source: Nat Commun. 2026 May 16;17:6496. doi: 10.1038/s41467-026-73303-w (PMC13377055; doi:10.1038/s41467-026-73303-w)
Supplement: Supplementary file 2 — Description of Additional Supplementary Files [file 41467_2026_73303_MOESM2_ESM.pdf]

Description of Additional Supplementary Files

File name: Supplementary Movie 1
Description: CMC-Cu(II), CMC-Fe(II), and CMC-Ca(II) fabrication by dipping-dipping process.
