# Supplementary material for: In situ formation of adaptive electronic skin in 2 seconds enabled by metal coordination
Source: Nat Commun. 2026 May 16;17:6496. doi: 10.1038/s41467-026-73303-w (PMC13377055; doi:10.1038/s41467-026-73303-w)
Supplement: Supplementary file 4 — Reporting Summary [file 41467_2026_73303_MOESM4_ESM.pdf]

nature portfolio

Corresponding author(s): Meng Gao

Last updated by author(s): May 2, 2026

Reporting Summary

Nature Portfolio wishes to improve the reproducibility of the work that we publish. This form provides structure for consistency and transparency in reporting. For further information on Nature Portfolio policies, see our Editorial Policies and the Editorial Policy Checklist.

Statistics

For all statistical analyses, confirm that the following items are present in the figure legend, table legend, main text, or Methods section.

n/a	Confirmed	
☐	☒	The exact sample size (*n*) for each experimental group/condition, given as a discrete number and unit of measurement
☐	☒	A statement on whether measurements were taken from distinct samples or whether the same sample was measured repeatedly
☐	☒	The statistical test(s) used AND whether they are one- or two-sided<br>*Only common tests should be described solely by name; describe more complex techniques in the Methods section.*
☒	☐	A description of all covariates tested
☒	☐	A description of any assumptions or corrections, such as tests of normality and adjustment for multiple comparisons
☐	☒	A full description of the statistical parameters including central tendency (e.g. means) or other basic estimates (e.g. regression coefficient) AND variation (e.g. standard deviation) or associated estimates of uncertainty (e.g. confidence intervals)
☒	☐	For null hypothesis testing, the test statistic (e.g. *F*, *t*, *r*) with confidence intervals, effect sizes, degrees of freedom and *P* value noted<br>*Give P values as exact values whenever suitable.*
☒	☐	For Bayesian analysis, information on the choice of priors and Markov chain Monte Carlo settings
☒	☐	For hierarchical and complex designs, identification of the appropriate level for tests and full reporting of outcomes
☒	☐	Estimates of effect sizes (e.g. Cohen's *d*, Pearson's *r*), indicating how they were calculated

Our web collection on statistics for biologists contains articles on many of the points above.

Software and code

Policy information about availability of computer code

Data collection

Membrane thickness: Hitachi SU 1510 scanning electron microscope (SEM)
Conformal properties: Qualitative assessment by attaching the membrane onto a wavy PVC mold followed by embrittlement in liquid nitrogen (Hitachi SU 1510 SEM)
Mechanical tests (90° peeling test, stress strain curves): Instron Corporation 3369 electronic universal testing machine
UV vis absorption spectra: JOSVOK UV 5600P UV vis spectrophotometer
Chemical state (XPS): Thermo Scientific K Alpha XPS equipment
FTIR spectra: Nicolet iS5 spectrometer (Thermo Scientific, USA)
Electrical impedance (LCR): TH2838H inductance capacitance resistance (LCR) meter (Tonghui)
X ray absorption fine structure (XAFS, EXAFS, XANES): BL11B beamline at Shanghai Synchrotron Radiation Facility (SSRF) with Si(111) crystal monochromators; 4 channel Silicon Drift Detector (SDD) Bruker 5040; transmission mode
Cell culture and treatment: Humidified incubator (37 °C, 95% humidity, 5% CO_2)
Cell staining: Calcein AM and propidium iodide
Cell imaging: Confocal laser scanning microscope (Olympus FV3000, Japan)
Quantum chemistry geometry optimization: Gaussian 16 package (B3LYP/6 311+G(d) level of theory)
Molecular dynamics simulation: GROMACS package (OPLS AA force field, SPC/E water model, cubic periodic boundary conditions, Verlet leapfrog integrator with 2.0 fs time step, cutoff 1.4 nm for short range interactions, particle mesh Ewald (PME) for long range electrostatics, NPT ensemble with Berendsen barostat at 1 bar and V rescale thermostat at 298.15 K, 40 ns simulation time)
DFT calculations (geometry optimization and energy calculation): Gaussian 16W software with B3LYP functional, 6 311+G* basis set, LANL2DZ effective core potential, and SMD continuum solvation model
ECG signals: ECG sensor with integrated ADS1292R signal acquisition converter, STM32F103C8T6 microcontroller, and Bluetooth transmission

device.
EOG and signals: Signal recording system with integrated bandpass filter (Neurosky TGAM EEG03, Jiangsu, China).
EEG signals: Same Neurosky TGAM EEG03 system.
EMG signals (arm): Six channel system including ZTEMG 1100 PCB (Zhituo Intelligent Technology Co., Ltd.), CH 50RB signal input terminal, and Handyscope Model HS4 oscilloscope (TiePie engineering, China).

Data analysis

XPS: Casa software.
XAFS data analysis: Athena and Artemis software.
DFT calculations: GaussianView 6.0 software
Others: Origin software

For manuscripts utilizing custom algorithms or software that are central to the research but not yet described in published literature, software must be made available to editors and reviewers. We strongly encourage code deposition in a community repository (e.g. GitHub). See the Nature Portfolio guidelines for submitting code & software for further information.

Data

Policy information about availability of data

All manuscripts must include a data availability statement. This statement should provide the following information, where applicable:

- Accession codes, unique identifiers, or web links for publicly available datasets
- A description of any restrictions on data availability
- For clinical datasets or third party data, please ensure that the statement adheres to our policy

The data generated in this study are provided in the main article, Supplementary Information and Source data file. All data are available from the corresponding author upon request. Source data are provided with this paper.

Research involving human participants, their data, or biological material

Policy information about studies with human participants or human data. See also policy information about sex, gender (identity/presentation), and sexual orientation and race, ethnicity and racism.

Reporting on sex and gender

In this work, the authors themselves participated in physiological signal collection experiments after providing informed consent. Sex and gender were not considered as variables in the study design. No sex- or gender-based analyses were performed, and relevant disaggregated data were not collected.

Reporting on race, ethnicity, or other socially relevant groupings

No socially constructed or socially relevant categorization variables were used in this study.

Population characteristics

No covariate-relevant population characteristics were assessed or reported in this study.

Recruitment

Participants were the authors of the present work. No self-selection bias was involved. Minor physiological differences do not compromise the key conclusions of the study.

Ethics oversight

N/A. The human experiments in this study only involved non-invasive physiological signal acquisition for the demonstration of device performance, and did not involve any health, safety or clinical outcome assessments.

Note that full information on the approval of the study protocol must also be provided in the manuscript.

Field-specific reporting

Please select the one below that is the best fit for your research. If you are not sure, read the appropriate sections before making your selection.

[x] Life sciences   [ ] Behavioural & social sciences   [ ] Ecological, evolutionary & environmental sciences

For a reference copy of the document with all sections, see nature.com/documents/nr-reporting-summary-flat.pdf

Life sciences study design

All studies must disclose on these points even when the disclosure is negative.

Sample size

No statistical methods were used to predetermine sample size. The sample size of 3 healthy human subjects was chosen according to common practices in non-invasive human physiological signal research. Data including ECG, EOG, EEG and EMG were collected from these 3 participants. This sample size is sufficient to obtain stable, representative and repeatable physiological signals, ensuring the reliability and reproducibility of the experimental results.

Data exclusions

Some signal segments contaminated by unexpected movement, vibration or instrumental artifacts were excluded from the analyses. These exclusions were based on pre-established criteria to remove abnormal data, ensuring the reliability of subsequent processing and interpretation of ECG, EOG, EEG and EMG signals.

Replication

Physiological signals including ECG, EOG, EEG and EMG were independently replicated three times per experiment for each of the three human subjects. ECG, EOG and EMG showed highly consistent and reproducible patterns. EEG signals exhibited expected minor variations across trials and individuals due to inherent physiological fluctuations and brain state differences, but the overall signal characteristics and key

	features remained reproducible. All replications were successful in validating the reliability of the experimental findings.al patterns were obtained across trials and subjects. These results confirm the good reproducibility of the experimental findings.
Randomization	This study involved the acquisition of physiological signals from healthy human participants without allocation into experimental groups. Therefore, randomization was not applicable. All recruited subjects were assessed under the same experimental conditions to ensure uniform data quality.
Blinding	This study only involves non-invasive collection and analysis of ECG, EOG, EEG and EMG signals from human participants, with no grouping of subjects or intervention treatments. Therefore, blinding of investigators during data collection and analysis was not applicable and not necessary for this study.

Reporting for specific materials, systems and methods

We require information from authors about some types of materials, experimental systems and methods used in many studies. Here, indicate whether each material, system or method listed is relevant to your study. If you are not sure if a list item applies to your research, read the appropriate section before selecting a response.

Materials & experimental systems

n/a	Involved in the study
☒	☐ Antibodies
☐	☒ Eukaryotic cell lines
☒	☐ Palaeontology and archaeology
☒	☐ Animals and other organisms
☒	☐ Clinical data
☒	☐ Dual use research of concern
☒	☐ Plants

Methods

n/a	Involved in the study
☒	☐ ChIP-seq
☒	☐ Flow cytometry
☒	☐ MRI-based neuroimaging

Eukaryotic cell lines

Policy information about cell lines and Sex and Gender in Research

Cell line source(s)	The human umbilical vein endothelial cells (HUVEC) were obtained from Haling Biotechnology Co., Ltd. (Shanghai, China).
Authentication	The HUVECs were commercially obtained and were used without further authentication. No cell line authentication procedures were performed.
Mycoplasma contamination	The cell lines were not tested for mycoplasma contamination.
Commonly misidentified lines (See ICLAC register)	N/A. HUVECs are not listed on the ICLAC register of commonly misidentified cell lines.

Plants

Seed stocks	N/A
Novel plant genotypes	N/A
Authentication	N/A
