# Supplementary material for: In situ formation of adaptive electronic skin in 2 seconds enabled by metal coordination
Source: Nat Commun. 2026 May 16;17:6496. doi: 10.1038/s41467-026-73303-w (PMC13377055; doi:10.1038/s41467-026-73303-w)
Supplement: Supplementary file 5 — Transparent Peer Review file [file 41467_2026_73303_MOESM5_ESM.pdf]

nature portfolio

Peer Review File

In Situ Formation of Adaptive Electronic Skin in 2 Seconds Enabled by Metal Coordination

Corresponding Author: Professor Meng Gao

This file contains all reviewer reports in order by version, followed by all author rebuttals in order by version.

Version 0:

Reviewer comments:

Reviewer #1

(Remarks to the Author)
The manuscript presents the development of an ionically conductive hydrogel film for e-skin. While the authors emphasize the novelty of their “milk-skin” inspired “dipping-dipping” molecular assembly method, it is important to note that CMC-based metal coordination with metal ions has been previously explored. The rapid membrane formation is interesting, but it lacks novelty, as this knowledge is already established. Additionally, the manuscript provides limited quantification and vague explanations of the underlying mechanisms. The demonstrated applications, such as ECG and EMG, may not be entirely suitable for the membranes. Consequently, I believe the manuscript is currently weak and may not be appropriate for publication in Nature Communications. Below are specific comments for improvement:
1. The authors may not understand the concept of electronic skin (e-skin). A basic electrode used for EEG measurements does not qualify as e-skin. E-skin typically refers to flexible materials that mimic the properties of human skin, enabling functionalities such as tactile sensing and stretchability. It is designed to provide a more comprehensive interface for various applications beyond simple electrical measurements. I recommend reviewing the definition and characteristics of e-skin to ensure that the manuscript accurately reflects this concept.
2. How do the authors control the thickness of the film? Thickness is crucial, as it directly influences the film's functionality. For instance, Figure 1d shows a thin 3.4 μm membrane, while other films appear significantly thicker, perhaps around hundreds of μm (e.g., Figures 1f, S7, and S8).
3. The assertion of skin compatibility is overly simplistic. Figure S20 presents a misleading experiment. The red tint observed upon removing the "commercial electrode" results from adhesive removal, not an allergic reaction to the material. Furthermore, comparing materials with different cross-sectional areas is not fair. I observed some rash on the CMC-Cu electrode after application and removal. This experiment lacks rigor and should not lead to claims of “biocompatibility” based solely on photographs. A comprehensive long-term study is needed.
4. The manuscript does not present the mechanical and electrical properties of the films. This information is critical for evaluating their performance.
5. Can the membrane retain water? If not, how will performance (impedance, EEG, ECG) change over time? In my experience, CMC-metal gels tend to lose water rapidly in ambient environments.
6. Additionally, in Figure S9, what happens to the material's functionality when submerged over time? I believe both conductivity and structural integrity will vary with submersion duration. Please provide quantification.
7. In Figure S4, the claim that CMC molecules in support channels induced by Cu(II) coordination bonds enhance ion transport lacks clarity. A more detailed explanation is needed.
8. The explanation regarding Figure S14—suggesting that a negative UV absorption peak in CMC-Ca(II) is due to weak electrostatic attractions or complexation—seems questionable. Did the authors observe this phenomenon visually? Was the calcium fully dissolved in the solution?
9. The caption for Figure S15 appears to be incorrect, as there are no graphs for CMC-Ca(II) over different time intervals.
10. Is there any mechanical characterization of MC and CMCH with Cu(II) (or other ions)? The manuscript lacks explanation for these materials.
11. As a reader not specialized in physiological monitoring, I find Figures 4, 5, S21, and S22 unclear. There is no explanation of the significance of the peaks and time-frequency spectrum. Specifically, in Figure S21, what do the authors mean by “EEG monitoring during mathematical calculation by CMC-Cu(II) electrodes”?
12. How is the gel applied to the skin? What is the adhesion force? Is there a risk of slippage? Will the gel dry over time? How practical is this as a device?
13. Can the CMC-Cu(II) electrode sustain measurements over extended periods? Physiological monitoring, such as EEG

and EMG, typically requires at least 60 minutes. How stable is the material during operation over an hour?
14. Was the comparison of impedance in Figure 4b fair?

Reviewer #2

(Remarks to the Author)
1. How does the performance of the CMC-Cu(II) bio-skin quantitatively compare to state-of-the-art electronic skins in terms of conductivity, biocompatibility, and fabrication scalability? A comparison with benchmarks would highlight the novelty and advantages of the proposed method.

2. Given the use of Cu(II) ions, what is the environmental fate of the degraded CMC-Cu(II) membrane, particularly regarding potential Cu(II) ion release into soil or water? Additionally, were authors able to conduct cytotoxicity or long-term skin contact studies to ensure biocompatibility, especially considering the potential toxicity of copper ions?

3. The manuscript claims the CMC-Cu(II) membrane remains stable in aqueous environments like sweat for 30 days. Can authors show experimental evidence that support this claim? How does the membrane's performance (e.g., conductivity, adhesion) hold up under dynamic conditions such as mechanical stretching, sweating, or prolonged wear?

4. While the "dipping-dipping" process is rapid and simple, how feasible is it for large-scale manufacturing or clinical applications? What challenges are there in achieving uniform membrane thickness or patterning complex circuits over large or irregular surfaces? How might these affect practical deployment?

5.Authors note that Ag(I), despite its +1 oxidation state, forms stable membranes comparable to Cu(II). What is the chemical or structural basis for this unexpected performance? Is it due to differences in coordination geometry, binding energy, or other factors? How does this impact the generalizability of the metal coordination strategy?

Reviewer #3

(Remarks to the Author)
This paper proposes metal-coordination bond assisted rapid-curable, in-situ forming ultrathin hydrogel for bio-electronic skin, showing its applications of monitoring various biopotential signals. Metal coordination bond-assisted rapid-curing of hydrogel allows in-situ formation of ultrathin conductive hydrogel on curved surface with any shape desired. Cu(II), Ag(I) metal ion coordination bond provides superior curing speed of hydrogel compared with other metal ions, and its mechanism is verified by experiments and density functional theory simulation. Various biopotential signals (EOG, EEG, ECG, EMG) were monitored with conductive bioelectrode and showed superior contact impedance and SNR compared to commercial Ag/AgCl bioelectrode, owing to its high conformability. In fact, curing of cellulose-based material by Cu(II) ion is already reported in previous literature (Nature 598, 590–596 (2021), Nat. Nanotechnol. 18, 168–176 (2023), Nat. Nanotechnol. 17, 629–636 (2022))), which were cited as references in the manuscript. So, the approach of utilizing metal coordination bond with Cu(II) ion and cellulose is not novel by itself. The novelty of this manuscript lies in rapid curing approach. However, the manuscript does not provide sufficient data to support this result. The density functional theory simulation and X-ray absorption spectroscopy analysis is not sufficient to explain "speed" of curing by metal ion coordination bond. They only show that CMC-Cu(II) and CMC-Ag(I) will be cured to form hydrogel by metal-ion coordination bond, which is already shown in previous literature. More analysis and discussion must be done to provide a mechanism to explain faster of hydrogel curing by Cu(II) and Ag(I) ions. In addition, there are several missing points in this manuscript, which are crucial for bioelectrode applications. (No electrical and mechanical properties characterization of hydrogel; Adhesion characteristics of hydrogel; no details of the method for in-situ fabrication of hydrogel on skin like face and neck; no EOG/EEG performance comparison with Ag/AgCl electrodes) Therefore, based on the amount of novelty and lack of supporting data, this manuscript is not suitable to be published in Nature Communications.

Version 1:

Reviewer comments:

Reviewer #2

(Remarks to the Author)
There are several grammatical errors and awkward phrases which should be corrected for better readability.

Reviewer #3

(Remarks to the Author)
Authors revised the manuscript according to the reviewer's comments. Therefore, the reviewer thinks that the paper is good for publication in Nat. Comm.

Reviewer #4

(Remarks to the Author)
[Editor's Note: This reviewer was invited to evaluate the response to Reviewer #1's evaluation]

Overall, the revision is clearly improved. The authors have substantially strengthened the manuscript in quantification, added mechanistic support, and clarified several technical points raised by Reviewer #1. In particular, the response is materially stronger on thickness control, mechanical/electrical characterization, and mechanistic explanation of the Cu(II)-CMC assembly process.

However, Reviewer #1's concerns are not entirely resolved.

The concerns regarding novelty have only been partially addressed. The authors have now clarified that the claimed novelty lies not in CMC-metal coordination itself, but in the ultrafast, in situ, transfer-free formation of a conformal membrane directly from solution on the target substrate. This distinction is clearer than before. However, the response mainly differentiates the work from a relatively narrow subset of prior CMC-based studies, rather than fully establishing sufficient novelty against the broader literature on rapid or in situ membrane/hydrogel formation. On this point, I consider the response improved but not fully convincing.

The concern regarding limited quantification and vague mechanistic explanation has been addressed substantially better. The authors have added UV spectroscopy, XANES/EXAFS, molecular dynamics simulations, and DFT analysis, and the revised results provide a more quantitative explanation for the rapid coordination of Cu(II) with CMC and film formation. I consider this part of the problem has been largely resolved.

The concern regarding suitability for epidermal applications is only partially resolved. The authors have repositioned the system as an epidermal electronic membrane and provide additional performance arguments for electrode use. However, the skin-compatibility issue remains weaker than the current claim language. The added 24 h HUVEC live/dead assay supports low short-term cytotoxicity, but it does not directly resolve Reviewer #1's original concern about skin rash and true epidermal-use safety; the authors also explicitly state that no additional skin-irritation data were provided. Thus, this issue is improved, but not fully resolved.

In summary, the authors have made a serious effort and have resolved many of Reviewer #1's technical concerns. However, the two core issues upon which Reviewer #1's negative evaluation was based—novelty and skin compatibility/epidermal suitability—remain partially unresolved. Therefore, my overall assessment is that the response letter is substantial and represents a significant improvement to the manuscript, but it does not fully address all the issues initially raised by Reviewer #1.

Open Access This Peer Review File is licensed under a Creative Commons Attribution 4.0 International License, which permits use, sharing, adaptation, distribution and reproduction in any medium or format, as long as you give appropriate credit to the original author(s) and the source, provide a link to the Creative Commons license, and indicate if changes were made.
In cases where reviewers are anonymous, credit should be given to 'Anonymous Referee' and the source.
The images or other third party material in this Peer Review File are included in the article’s Creative Commons license, unless indicated otherwise in a credit line to the material. If material is not included in the article’s Creative Commons license and your intended use is not permitted by statutory regulation or exceeds the permitted use, you will need to obtain

permission directly from the copyright holder.
To view a copy of this license, visit https://creativecommons.org/licenses/by/4.0/

Itemized list of response to the reviewers' remarks

(Black: Reviewers' remarks; Blue type: Our response)

Reviewer #1 (Remarks to the Author):

The manuscript presents the development of an ionically conductive hydrogel film for e-skin. While the authors emphasize the novelty of their "milk-skin" inspired "dipping-dipping" molecular assembly method, it is important to note that CMC-based metal coordination with metal ions has been previously explored. The rapid membrane formation is interesting, but it lacks novelty, as this knowledge is already established. Additionally, the manuscript provides limited quantification and vague explanations of the underlying mechanisms. The demonstrated applications, such as ECG and EMG, may not be entirely suitable for the membranes. Consequently, I believe the manuscript is currently weak and may not be appropriate for publication in Nature Communications. Below are specific comments for improvement:

Author reply: We thank Reviewer #1 for the constructive feedback and thoughtful evaluation of our manuscript. We highly value your comments. The reviewer has put forward insightful points in three crucial areas:

(i) concerns regarding the novelty of our work, particularly related to previously reported studies on CMC-based metal coordination with metal ions;

(ii) the importance of quantification and clear explanations elucidating the underlying mechanism;

(iii) the suitability for use in epidermal electronic applications for ECG, EMG, and so on.

Below, we address these issues point-by-point, starting with a clarification of our work's novelty and how it differs from the cited literature.

1. Clarifying the Novelty of Our Work in Comparison to Prior Studies

We sincerely appreciate your insightful suggestion. You raised the concern that CMC-based metal coordination with metal ions might undermine the significance of our work's novelty. We therefore elaborate from two perspectives: (i) the differences between our work and the reported literature; and (ii) the novelty of our work.

i. The differences between our work and the reported literature

The coordination interaction between CMC and metal ions such as Cu(II) has been reported in prior studies. However, our approach differs fundamentally. Although the coordination interaction between CMC and metal ions such as Cu(II) has been reported in prior studies, this interaction is only recognized as a strong physical interaction for mechanical/functional improvement or Cu(II) adsorption and removal from water (**Figure R1a**). Additionally, the reported studies commonly use formed gel or membrane to interact with Cu(II) and thereby impart functionality (**Figure R1a**), whereas our work uses a biomass-based solution that interacts with Cu(II) to form a conformal membrane first, which is then used as an epidermal electrode (**Figure R1b**).

That is, none of those works developed a skin-membrane strategy that enables ultrafast (<2 s), in situ membrane fabrication on targeted substrate—an approach that is fundamentally different in terms of materials design, processing methodology, and functional application, with features that also perfectly match the requirements for epidermal electronics.

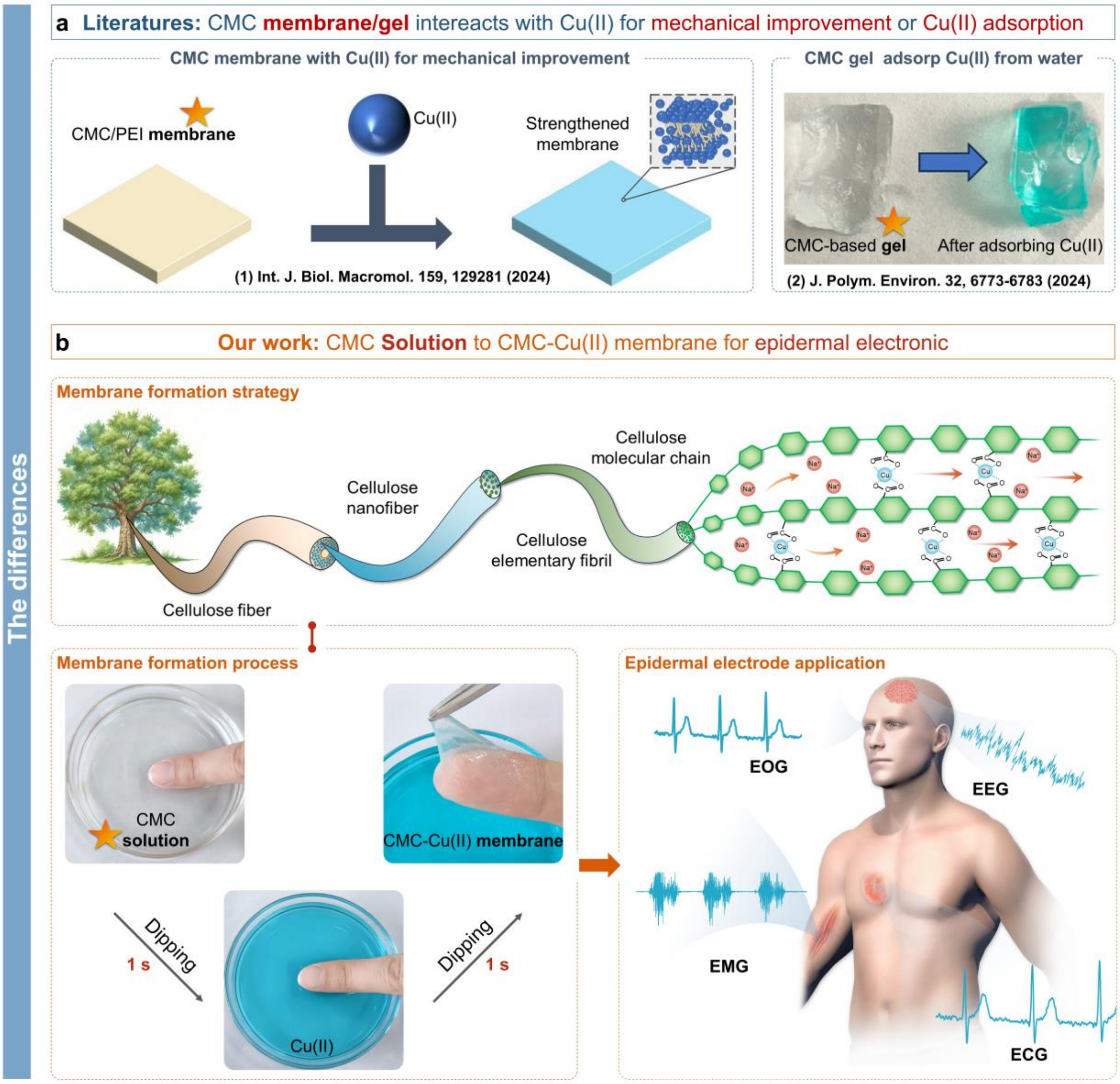

Figure R1. The differences between reported literatures about CMC-based metal coordination and our work a The reported literatures use CMC-based membranes/gel to interact with Cu(II) and thereby impart functionality.[1,2] **b** Our work uses CMC solution interacting with Cu(II) to form a conformal membrane for epidermal electrode.

ii. The novelty of our work

Before providing specific responses, it is worth noting that, for epidermal electrodes, impedance is the primary electrical parameter that reflects their ability to acquire high-quality bioelectric signals from the skin surface. A low electrode-skin impedance indicates superior physiological signal detection and conduction capabilities. Typically, the electrode-skin interface is modeled as a parallel circuit (LCR circuit) consisting of leakage resistance and capacitance (**Figure R2**), which illustrates the measurement setup for the electrode-skin impedance in dual-electrode recording configurations. For each recording electrode, the

electrode-skin impedance can be derived according to the following equation:

$$Z(\omega) = R_s + \frac{R_d/jC_d\omega}{R_d+1/jC_d\omega} = R_s + \frac{R_d}{1+jC_dR_d\omega}, \quad \omega=2\pi f \qquad (1)$$

Where the resistor R_d represents the charge transfer impedance of the electrode-skin interface, while the capacitance C_d stands for the electric double layer between the electrode and skin. R_s is related to the skin surface conditions and underlying tissues, and R_{tissue} is the resistance of the dermis, subcutaneous tissue layers, and deeper tissues. Thus, maintaining high interfacial capacitance and low resistance at the electrode-skin interface (Eq.1) is crucial for minimizing bioelectrical signal attenuation and enhancing the overall signal-to-noise ratio (SNR)[3]. This theoretical framework explains the low electrode–skin impedance observed with our ultrathin, conformal CMC-Cu(II) membrane, confirming its suitability for epidermal signal acquisition.

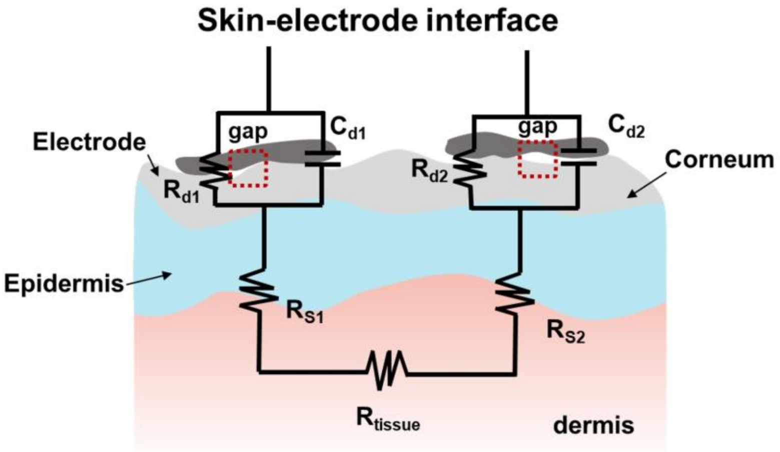

Figure R2. Simplified LCR circuit model of the electrode-skin interface in epidermal electronic applications.

Based on the above comparison with the previous literature, it can be concluded that our CMC–Cu(II) approach constitutes a unique membrane-forming strategy, and the novelty of our work includes (**Figure R3**): (i) developing an ultra-fast (≈2 s), highly convenient in-situ membrane formation strategy, whose mechanism has been systematically investigated and has guided us to expand the method to other systems; (ii) the achieved membrane exhibits various advantageous features, including ultrathin thickness (~3.4 μm), conformality, patternability, a transfer-free process (no additional post-transfer step), conductivity, low impedance, biocompatibility, and biodegradability, which fit perfectly for epidermal electrodes for physiological monitoring. Detailed explanations are as follows.

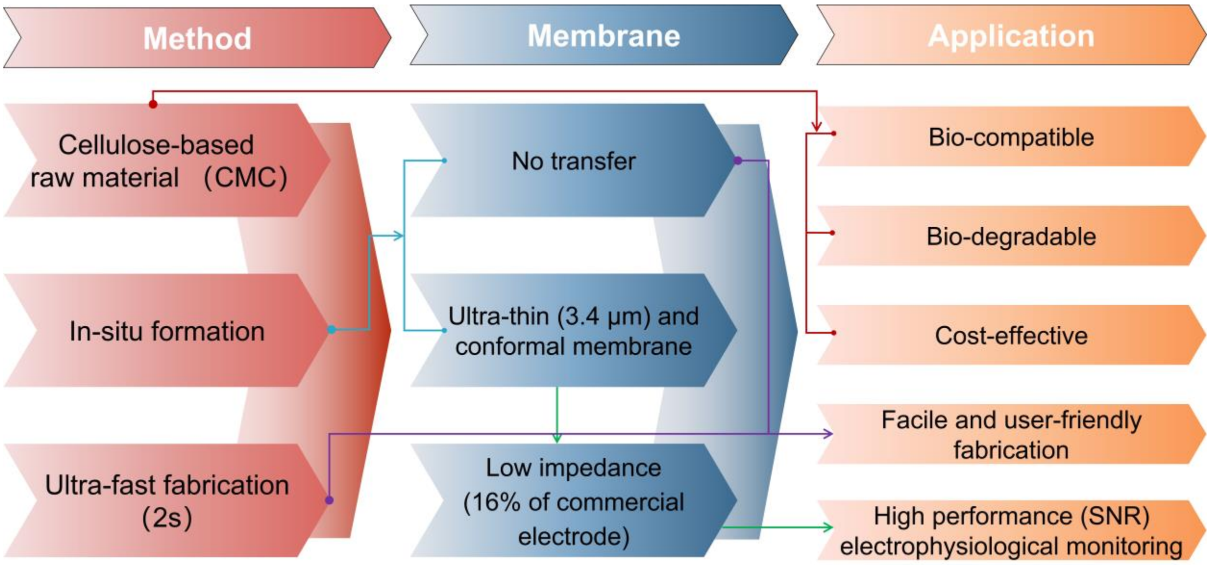

Figure R3. Illustration of the CMC-Cu(II) advantages for epidermal electronics from method to membrane and epidermal electrode applications.

- **Unique membrane formation technology.** The key novelty of this study is a facile "dipping–dipping" molecular-assembly method using the CMC-Cu(II) system that enables an ultra-fast liquid-to-solid transformation. Unlike traditional routes of casting → drying/curing → transfer, which are complicated, our approach directly forms the bio-membrane in situ by sequentially dipping the target surface into CMC solution and then Cu(II), which is highly convenient and time-saving. The rapid solidification is driven by strong coordination and accelerated interfacial assembly, elucidated by experiments (UV–vis, XANES, EXAFS) and simulations (MDS—captures nanosecond interfacial assembly kinetics; DFT—quantifies coordination structures and binding energies consistent with XANES/EXAFS). We further generalize the strategy to other metal ions (Ca(II), Fe(II), Ag(I)) and other biomacromolecules (Methylcellulose, MC; carboxymethyl chitosan, CMCH), establishing design rules that relate coordination energetics and interfacial assembly dynamics to membrane-forming efficacy, thereby delivering a transfer-free, substrate-agnostic, seconds-scale route to ultrathin, conformal membranes with tunable performance for bio-integrated electronics (**Figure R3**).
- **Multiple advantageous features that perfectly suit epidermal electrodes.** The method—and the resulting membrane—offer a suite of advantages that meet the core requirements of epidermal electrodes (**Figure R3 and R4**). From the materials perspective, the membrane is ultrathin and conformal, enabling low interfacial impedance and a high signal-to-noise ratio for high-performance bioelectrical measurements—its most critical attribute. In addition, being derived from a cellulose derivative, the membrane is biocompatible and biodegradable, making it cost-effective, skin-benign, and environmentally friendly. From the manufacturing perspective, the process is ultra-fast and convenient; the in-situ formation strategy eliminates transfer steps that could damage fragile membranes, and its inherent patternability broadens application scenarios. Collectively, these features make the membrane an excellent fit for high-fidelity acquisition of physiological signals (ECG, EOG, EMG, EEG).

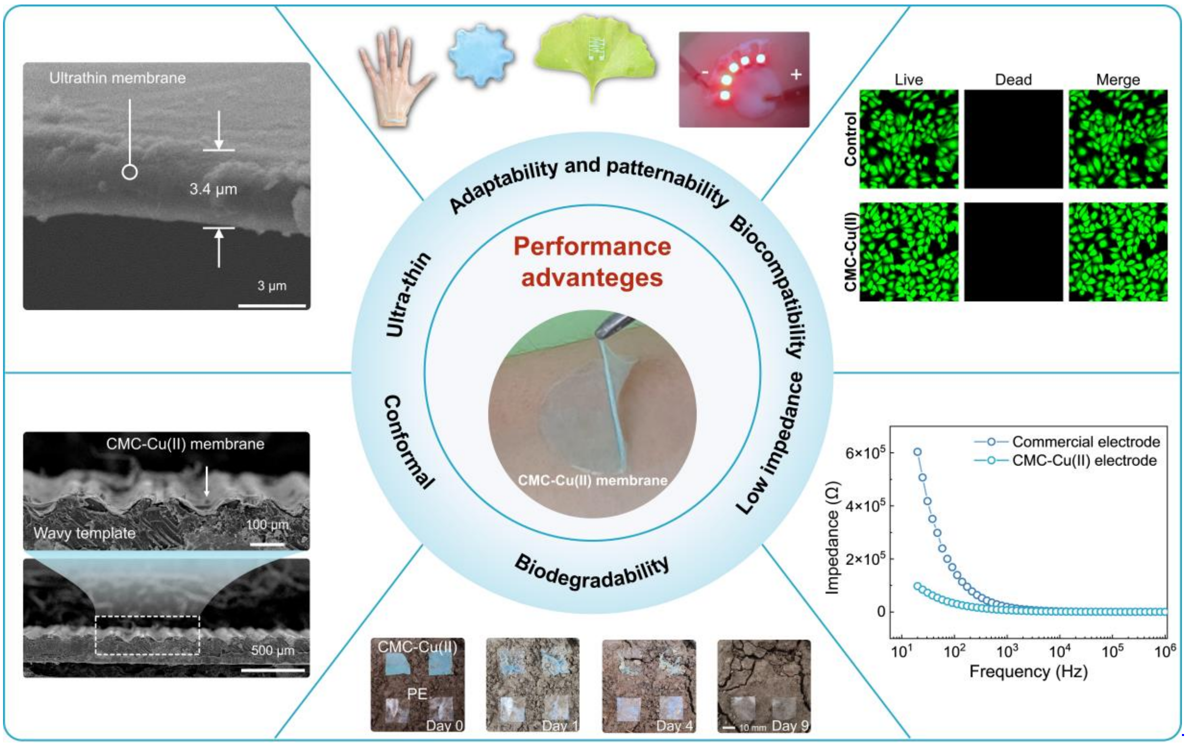

Figure R4. The combination of attributes about functional performance of CMC-Cu(II) membrane involving ultrathin, conformal, adaptive, patternable, degradable, biocompatible and low impedance characteristics.

In conclusion, this work presents a novel nature-inspired "dipping-dipping" assembly method and systematically validates its scientific foundation and practical significance through systemic evaluations of performance, underlying mechanisms, and application potential. Furthermore, the approach demonstrates broad universality and scalability. We believe this work possesses advancement in the field of "controlled preparation methods for flexible electronic materials," offering clear innovation and translational potential.

References:

1. Shan Z, Huang J, Huang Y, Zhou Y, Li Y. Copper ions reinforced flexible carboxymethylcellulose/polyethyleneimine composite films with enhanced mechanical properties, UV-shielding performance, thermal stability, solvent resistance, and antibacterial activity. *Int. J. Biol. Macromol.* **259**, 129281 (2024).
2. Liu X, Xie Z, Zhu C. Preparation of Zirconium Phosphate/Carboxymethyl Cellulose Composite Hydrogel for Cu^{2+} Removal. *J. Polym. Environ.* **32**, 6773-6783 (2024).
3. Tang H, *et al.* In Situ Forming Epidermal Bioelectronics for Daily Monitoring and Comprehensive Exercise. *ACS Nano* **16**, 17931-17947 (2022).

2. The mechanism investigation of the fast CMC-Cu(II) coordination

Thank you for your valuable suggestions. The quantitative and clear explanation you have provided regarding the membrane-forming mechanism of the CMC-Cu(II) is of utmost importance. We fully agree with this view. The mechanism of the CMC-Cu(II) membrane encompasses not only the liquid-to-solid transformation (molecular assembly) of CMC-Cu(II),

but more crucially, the underlying mechanism of the "speed" of this transformation. "Rapid molecular assembly" facilitates the formation of a uniform and well-structured "thin" membrane and enhances the processing convenience and applicability of this membrane in epidermal electronic application scenarios. Therefore, to systematically investigate the underlying mechanisms involved in the membrane formation and membrane-forming speed of CMC-Cu(II), in the revised version:

(1) We supplemented molecular dynamics simulations (MDS) to directly demonstrate the ultra-fast molecular assembly speed (40 ns) of CMC-Cu(II).

(2) The MDS-derived data and existing synchrotron X-ray absorption spectroscopy (XAS) have disclosed the detailed actual coordination information within CMC-Cu(II).

(3) The UV spectra results indicate that the characteristic peaks of CMC-Cu(II) remain unchanged over time, which corroborates the ultra-fast coordination between Cu(II) and CMC.

(4) We re-conducted systematic density functional theory (DFT) simulation and calculation of binding energies, which reveals that the higher binding energy of CMC-Cu(II) is the fundamental underlying mechanism for its extremely rapid membrane-formation speed, from a thermodynamic perspective.

By cross-validating the simulation and calculation results with the UV spectra and XAS analysis results, we systematically revealed the mechanism of CMC-Cu(II) coordination interaction, especially its ultrafast “speed” of interface liquid-solid transformation.

Specifically:

- **MDS:** As shown in **Figure R5a**, the simulation snapshot results reveal that, the CMC molecules initially dispersed in the aqueous environment and rapidly aggregated within 40 ns under the strong coordination forces of Cu(II), thereby completing molecular assembly and forming a solid-state thin membrane. This result directly illustrates the ultra-fast assembly “speed” of CMC under the coordination of Cu(II).
- **MDS and XAFS:** As shown in **Figure R5b**, quantitative analysis of the interaction between carboxyl (-COO) and hydroxyl (-OH) groups in the CMC chain and copper (II) ions was performed using radial distribution function data from MDS. The results showed a stronger coordination tendency between Cu(II) and carboxyl groups, indicated by higher peak intensity in the distribution function, suggesting that CMC assembly is primarily driven by Cu(II)-carboxyl coordination rather than Cu(II)-hydroxyl coordination.

  Copper K-edge XANES results show that the electronic state of Cu in CMC-Cu(II) closely resembles that of CuO. The presence of a characteristic Cu(II) absorption peak confirms copper is in the +2 oxidation state without further chemical changes. EXAFS fitting analyses reveal that Cu(II) forms two oxygen-coordinated types with CMC, with coordination numbers of 2.2 ± 0.6 and 1.8 ± 0.7 (**Supplementary Table 2**), summing to approximately 4.

  The results of MDS and XAFS suggest that Cu(II) forms a local coordination structure closed to the square planar with carboxyl groups of CMC by bidentate coordination mode.
- **UV-spectra:** As shown in **Figure R5c**, Firstly, when compared with the UV characteristic peaks of CMC and Cu(II), a new UV absorption peak emerged in CMC -Cu(II), which demonstrated the formation of a "new substance", specifically the

CMC-Cu(II) complex. Furthermore, above the mixtures of the three metal ions (Cu(II), Fe(II), and Ca(II)) with CMC, the UV absorption peak of CMC-Cu(II) remains completely unchanged over time, indicating Cu(II) and CMC forming a complete coordination bond immediately upon contact. The peak shape and position of CMC-Fe(II) have changed significantly over time, demonstrating Fe(II) consistently reacts with CMC, yet not at a rapid speed. In CMC-Ca(II), since its coordination effect is too weak and no typical UV absorption peak is observed, it shows a different behavior. This result further corroborates the ultra-fast coordination speed between Cu(II) and CMC.

- **DFT:** As shown in **Figure R5d**, the structures of CMC-Cu(II), CMC-Fe(II), and CMC-Ca(II) were optimized, and their binding energies were calculated. The results indicate that the binding energies of these three compounds are -5.82 eV, -2.16 eV, and -1.15 eV, respectively. A negative binding energy suggests that the coordination reactions of the three systems occur spontaneously. The larger absolute value of the binding energy from CMC - Cu(II) further demonstrates that Cu(II) possesses a stronger coordination ability, which is the underlying reason for the rapidity of the reaction.

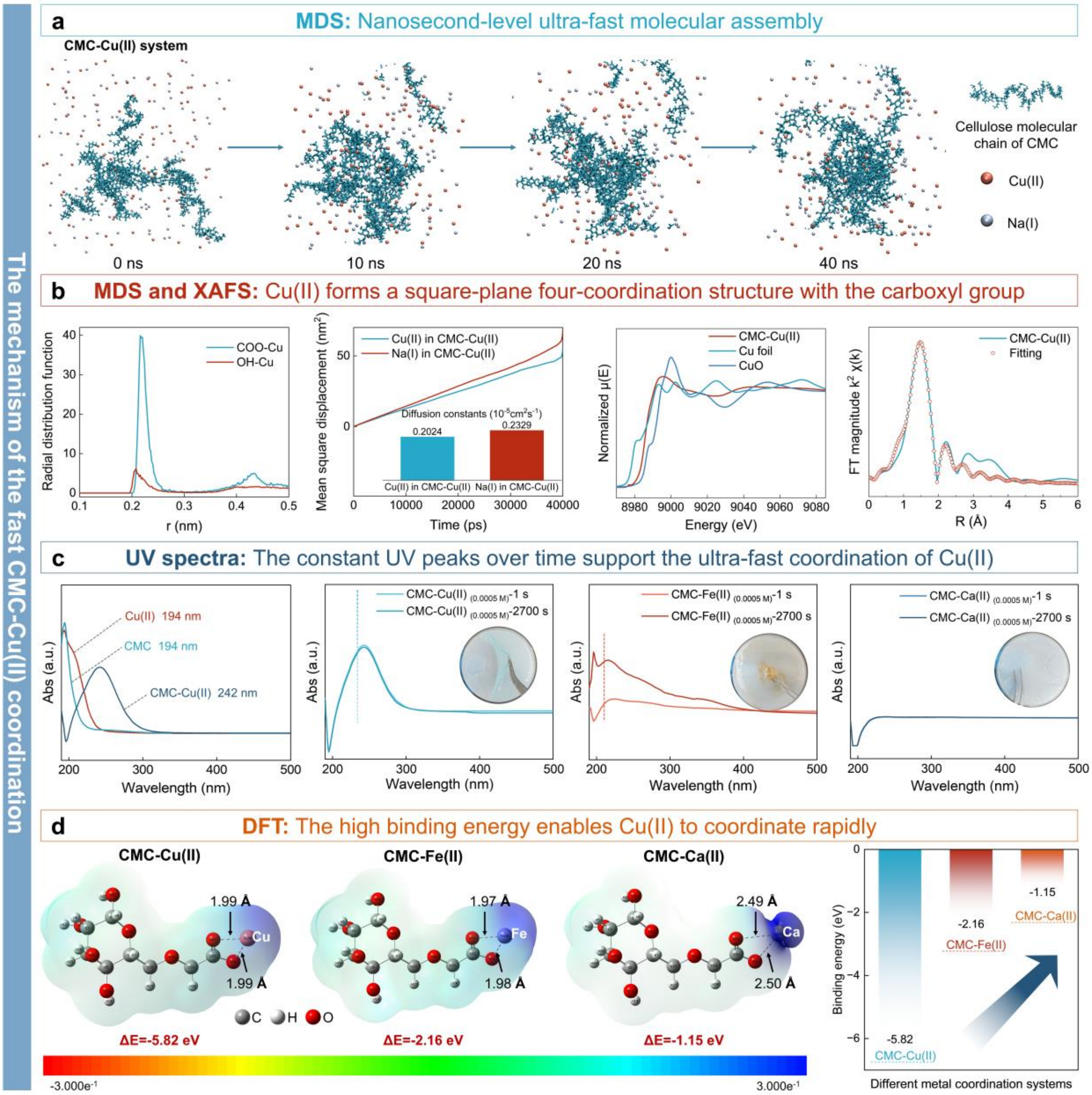

Figure R5. The mechanism characterization and simulation of the fast CMC-Cu(II) coordination. a The snapshot of the Cu(II)-induced CMC molecular assembly within 40 ns obtained by the MDS, clearly demonstrates the ultra-fast coordination of CMC-Cu(II). **b** The radial distribution function of Cu (II), and mean square displacement of Cu(II) and Na(I) around CMC-Cu(II) system from MDS, combined with EXAFS and XANES data from XAFS, show that Cu(II) coordinates with two carboxyl oxygen atoms in CMC to form a nearly square four-coordinate structure. **c** The UV spectra of the three metal ions (Cu(II), Fe(II), and Ca(II)) after mixing with CMC demonstrate that only CMC-Cu(II) displays a characteristic peak that remains unchanged over time, which indicates the rapid coordination of Cu(II) with CMC. **d** The static electron density maps of the coordination structures of CMC-Cu(II), CMC- Fe(II), and CMC-Ca(II), calculated by DFT, along with their corresponding binding energies, show that the absolute value of the binding energy of CMC - Cu(II) is the highest.

Supplementary Table 2 | EXAFS coordination environment analysis of CMC-Cu(II).

Sample	Scattering Pair	CN	R (Å)	σ^2	ΔE_0 (eV)
CMC-Cu(II)	Cu-O	2.2±0.6	2.14±0.01	2.4±0.3	9.0±0.1
	Cu-O	1.8±0.7	2.00±0.01		

CN: coordination number, R: bond distance, σ^2: Debye-Waller factors, and ΔE_0: energy shift.

In the revised manuscript, **Figure R5a** has been substituted with **Fig. 3a**; the two left- most figures in **Figure R5b** (The Radial distribution function and the Average orientation shift) have been supplemented as **Fig. 3b and Fig. 3c**; and **Figure R5d** (The re-run DFT calculation) has been revised to **Fig. 3f**.

Updates to the revised manuscript: According to Reviewer #3's comments, we added the above results in the revised manuscript on Pages 12-14, 29.

"Mechanism characterization and simulation of molecular assembly

*To investigate the influence of Cu(II)'s strong coordination on molecular chain dynamics and ion transport pathways at the molecular level, molecular dynamics simulations were conducted to analyze the structural evolution and ionic transport behavior of the molecular chains in the CMC-Cu system (**Supplementary Table 1**). As shown in **Fig. 3a**, the simulation snapshot results reveal that, the CMC molecules initially dispersed in the aqueous environment and rapidly aggregated within 40 ns under the strong coordination forces of Cu(II), thereby completing molecular assembly and forming a solid-state thin membrane. Furthermore, the interactions between carboxyl (-COO) and hydroxyl (-OH) groups in the CMC molecular chain and Cu(II) are quantitatively analyzed using radial distribution functions. The results indicate a stronger preference of Cu(II) for coordination with -COO groups compared to -OH groups, as evidenced by the higher peak intensity in the distribution function (**Fig. 3b**). This suggests that CMC molecule assembly is primarily driven by the coordination between Cu(II) and -COO groups along the polymer chain. Moreover, the migration behavior of Na(I) and Cu(II) along the CMC chain direction was evaluated by calculating their diffusion coefficients. The calculated diffusion coefficients are 0.20 × 10^{-5} $cm^2 \cdot s^{-1}$ for Cu(II) and 0.23 × 10^{-5} $cm^2 \cdot s^{-1}$ for Na(I) (**Fig. 3c inset**), confirming faster diffusion of Na(I). Additionally, the simulated mean square displacements of Cu(II) and Na(I) in the CMC-Cu(II) system are 55.40 nm^2 and 68.36 nm^2, respectively (**Fig. 3c**), indicating that Na(I) exhibits a higher displacement rate. This supports the effectiveness of the ionic conduction mechanism in the CMC-Cu(II) system. Collectively, the MDS validate the feasibility of the proposed strategy that utilizing strong Cu(II) coordination to induce ultra-fast molecular assembly for the formation of ultrathin conductive bio-membranes, which offers significant practical advantages for on-demand applications in fields such as biomedicine and wearable electronics.*

To further reveal and elucidate the detailed structure characteristics and transient

assembly mechanism of metal ion coordination membranes, X-ray absorption spectroscopy (XAS) is employed to analyze the Cu valence and coordination state in formed CMC-Cu(II) membrane complex. Furthermore, density functional theory (DFT) calculations are conducted to determine the most stable coordination structures. The coordinated state of copper ions in the CMC-Cu(II) membrane is verified by XAS. The results of the Cu K-edge X-ray absorption near-edge spectrum (XANES; ***Fig. 3d****) indicate that the electronic state of the Cu ions in CMC-Cu(II) is closer to the CuO, showing characteristic absorption peaks of Cu(II), which supports that the Cu in CMC-Cu(II) is in a +2 state without other chemical changes. Fitting of the XANES and extended X-ray absorption fine structure (EXAFS;* ***Fig. 3e and Supplementary Fig. 19****) spectra reveals two Cu-O distances of 2.14 ± 0.01 Å and 2.00 ± 0.01 Å (****Supplementary Table 2****). These distances can be attributed to the coordination of Cu(II) with the single and double bonds of oxygen atoms in the carboxyl groups of CMC. Additionally, we observe that the coordination numbers of copper in CMC-Cu(II) are 2.2 ± 0.6 and 1.8 ± 0.7, respectively (****Supplementary Table 2****), indicating a total coordination number of approximately 4. This suggests that the carboxyl groups of CMC form a coordination structure closed to the square planar with Cu(II) by bidentate coordination mode.*

To support and display the coordination structures and corresponding binding energy, CMC-Cu(II), CMC-Fe(II), and CMC-Ca(II), which exhibit different membrane-forming behaviors, are selected as representative examples. One glucose unit substituted with a carboxymethyl group is employed as model to simulate their theoretical optimal structures using DFT theory, with consideration of the solvent effect. ***Fig. 3f*** *illustrates the coordination structure of the complexes. The Cu(II) ion forms a bidentate coordination with the two oxygen atoms of a single carboxyl group on the CMC chain (based on the result of* ***Fig. 3b****). The calculated coordination bond lengths of C=O and C-O are both 1.99 Å, which is in good agreement with the experimental fitting results (~2.14 ± 0.01 Å and ~2.00 ± 0.01 Å). It is speculated that in the actual reaction, the Cu(II) ion will form a bidentate coordination with the carboxyl group on another molecular chain, ultimately forming a typical tetra-coordinated configuration that approximates a regular tetrahedron. This is highly consistent with the structure inferred from EXAFS analysis. In the simulated system containing a single glucose unit, Fe(II) and Ca(II) display coordination configurations similar to that of Cu(II), yet the lengths of the coordination bonds are distinct. The calculated bond lengths of Fe=O and Fe-O are 1.97 Å and 1.98 Å respectively, whereas the bond lengths of Ca=O and Ca-O are 2.49 Å and 2.50 Å respectively. All these values are in line with the conventional theoretical values.*

*The binding energy calculation further quantifies the strength of the coordination interaction at the thermodynamic level (****Fig. 3g****,* ***Supplementary Table 3****). The calculated binding energies of the three systems, CMC-Cu(II), CMC-Fe(II), and CMC-Ca(II), are -5.82 eV, -2.16 eV, and -1.15 eV respectively. All these values are negative, indicating that all three reactions are spontaneous. Moreover, the absolute values of the binding energies of the three systems followed the order: CMC-Cu(II) > CMC-Fe(II) > CMC-Ca(II). When the binding energy is negative, a larger absolute value indicates a more stable combination of substances. The binding energy results indicate that among the three systems, CMC-Cu(II) forms the most stable coordination complex, followed by CMC-*

Fe(II), and CMC-Ca(II) forms the least stable one. This is consistent with the previous experimental phenomena and characterization results"

"Characterization

Molecular dynamics simulation

A molecular dynamics simulation was conducted to investigate the ultra-fast molecular assembly behavior driven by Cu(II) coordination force of CMC-Cu system at aqueous environment. Specifically, quantum chemistry calculations were first performed to optimize molecular geometries of CMC using the Gaussian 16 package at B3LYP/6-311+G(d) level of theory. The atomistic force field parameters for all ions and molecules are described by the OPLS-AA and water was taken from the SPCE model. In the CMC-Cu(II) simulation system, 10 CMC, 100 $CuCl_2$, 130 Na(I), and 11112 water molecules were randomly inserted into a simulation box with x = 7.2 nm, y = 7.2 nm, and z =7.2 nm.

Atomistic simulations were performed using the GROMACS package with cubic periodic boundary conditions. The equations for the motion of all atoms were integrated using a classic Verlet leapfrog integration algorithm with a time step of 2.0 fs. A cutoff radius of 1.4 nm was set for short-range van der Waals interactions and real-space electrostatic interactions. The particle-mesh Ewald (PME) summation method with an interpolation order of 4 and a Fourier grid spacing of 0.12 nm was employed to handle long range electrostatic interactions in reciprocal space. In all the three directions, periodic boundary conditions were imposed. Leapfrog algorithm was used to integrate the Newtonian equation of motion. The MD simulation was processed in an NPT ensemble and the simulation time is 40 ns. In NPT simulations, the pressure was maintained at 1 bar by the Berendsen barostat in an isotropic manner, and the temperature was maintained by the V-rescale thermostat at 298.15 K."

3. Clarifying the suitability of CMC-Cu(II) for use in epidermal electronic applications

In response to the reviewer's concerns regarding the applicability to physiological signal applications such as ECG and EMG, we have clarified the key requirements for epidermal electronics and highlighted how the CMC-Cu(II) membrane meets these criteria through its multiple favorable properties, as detailed below.

i. Requirements for Epidermal Electronics

Epidermal electronics aim to establish a seamless interface with human skin to enable high-quality biomedical signal monitoring, demonstrating significant potential in health monitoring, clinical diagnostics, and human-machine interaction. To fulfill these roles, the materials used must satisfy several critical criteria:

- **Strong conformal adhesion** to reduce motion artifacts and contact noise;
- **Low interfacial impedance** for accurate signal acquisition;
- **Simple, rapid, and cost-effective fabrication methods** suitable for scalable production;
- **Biocompatibility and safety** for prolonged skin contact;
- **Environmental degradability** to minimize long-term ecological impact.

ii. CMC-Cu(II) Membrane Meets Key Criteria for Epidermal Electronics

In response to Reviewer #1's concerns regarding the applicability of our material for physiological signal monitoring, we have systematically clarified how the CMC-Cu(II) membrane addresses the above requirements, as summarized below and demonstrated in **Figure R6**.

- **Conformality & Low Impedance**

  Among all the factors, conformability is of vital importance for achieving high-quality signal acquisition of epidermal electronics. According to the simplified LCR circuit model of the electrode-skin interface and the impedance calculation equation (Eq.1), imperfect electrode-skin contact introduces significant contact impedance, leading to severe attenuation of bioelectrical signals and increased noise. Rigid or poorly conformal electrodes typically establish only limited point contacts, resulting in persistently high contact impedance. In contrast, a tight conformal fit maximizes the effective contact area, substantially reducing contact impedance and thereby improving signal fidelity and monitoring accuracy. Therefore, achieving excellent conformal contact remains one of the core challenges in the design of epidermal electronics.

  The ultrathin and flexibility nature of the membrane enables excellent conformal adhesion. Our "dipping-dipping" in-situ molecular assembly strategy effectively achieves these two critical features. It allows for rapid in-situ membrane formation on biological surfaces of arbitrary geometry within 2 seconds, completely eliminating alignment issues and mechanical damage associated with conventional "preparation-transfer" processes (**Figure R6b**). Simultaneously, the resulting micrometer-scale ultrathin layer (~3.4 μm, **Figure R6a**) can deform synchronously with skin surface movements and folds like a "second skin," preventing delamination. This seamless integration (**Figure R6a)** is the key factor responsible for the low interfacial impedance observed. Notably, the CMC-Cu(II) membrane exhibits significantly lower impedance compared to commercial Ag/AgCl gel electrodes across the frequency spectrum (**Figure R6d**), underscoring its superior signal acquisition performance.

- **Rapid, Transfer-free, and Cost-Effective Fabrication**

  The fabrication process is a major bottleneck for the scalability of soft bioelectronic systems. Our proposed "dipping–dipping" approach allows for in-situ membrane formation within 2 seconds on diverse substrates, including soft rubber, hydrogel, metal nuts, fresh fruit, and biological skin (**Figure R6b-i**). This method eliminates the complex transfer steps typically required for ultrathin membranes and avoids alignment issues or membrane damage during handling. Moreover, the process supports high pattern customizability (**Figure R6b-ii** and **iii**), enabling freeform patterning without photolithography or masks, which is vital for adapting to individualized anatomy or device layouts. The materials used—carboxymethyl cellulose and copper salt—are inexpensive and commercially available, making the approach economically viable for large-scale or disposable healthcare applications.

- **Biocompatibility & Safety**

  Biocompatibility is a non-negotiable requirement for long-term skin-contact devices. The CMC-Cu(II) membrane leverages carboxymethyl cellulose, a well-known biocompatible and FDA-approved material, as the structural matrix. The Cu(II) ions are stably

coordinated within the membrane and showed negligible release under physiological conditions, ensuring long-term safety. To validate safety, we conducted in vitro cytotoxicity assays using human umbilical vein endothelial cells (HUVECs), showing 100% cell viability after 24-hour exposure (**Figure R6e**), with no significant cell death observed in live/dead staining.

- **Environmental Degradability**
  Sustainable and degradable electronics are critical to reducing long-term environmental burden. The CMC-Cu(II) membrane is composed entirely of biodegradable materials, as shown in the soil degradation experiment (**Figure R6f**). Within 9 days, the membrane partially decomposed in moist soil, while traditional plastic films (PE) remained unchanged. Such environmental responsiveness ensures that spent electrodes will not contribute to microplastic pollution. This is particularly advantageous for single-use diagnostics or community-scale health monitoring systems where large volumes of bioelectronic waste may be generated.

The CMC-Cu(II) ultrathin membrane system systematically fulfills all the essential criteria for epidermal electronics, offering a well-balanced combination of conformal adhesion, low impedance, biocompatibility, scalability, and environmental sustainability. These integrated performance attributes make it a highly competitive and promising platform for wearable biomedical applications. We appreciate the reviewers' constructive suggestions, which have helped us refine our discussion. In addition to epidermal electronics, the material's rapid in-situ processability, flexibility, and ionic conductivity suggest broader potential in flexible sensors, intelligent packaging, and biointerfaces. Future work will focus on expanding and tailoring these applications.

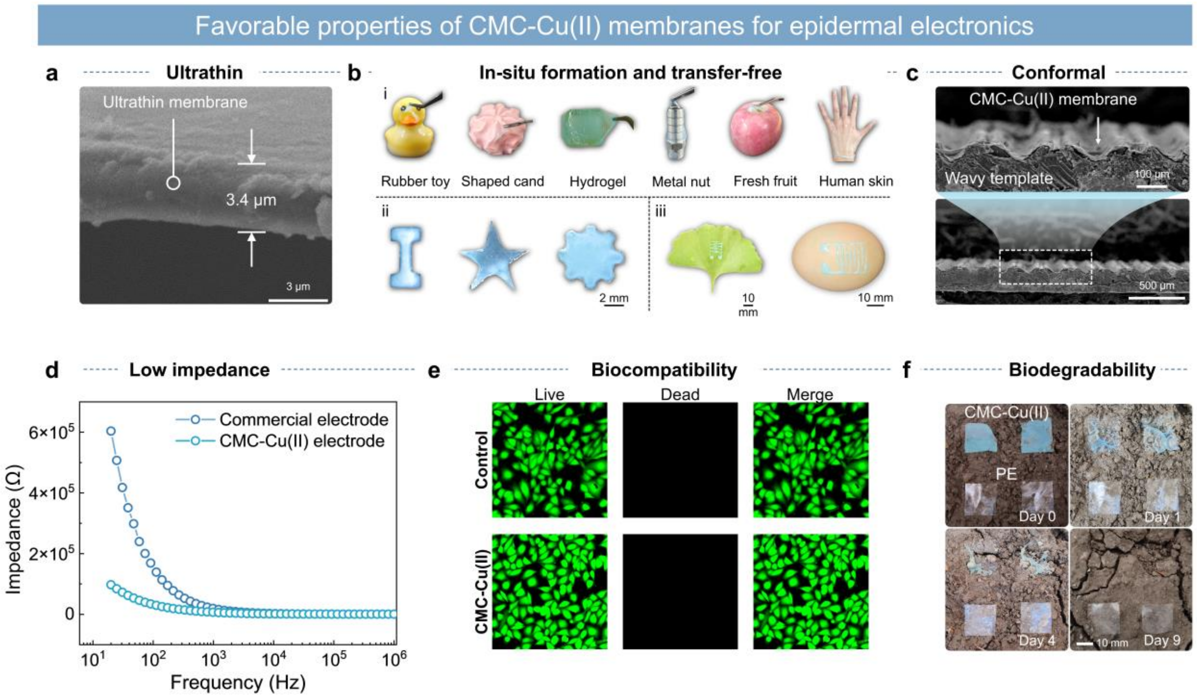

Figure R6. The favorable functional and performance characteristics of CMC-Cu(II) membrane which is well-suited for application as epidermal electrode. a Cross-sectional SEM image of ultrathin CMC-Cu(II) membrane. **b** The adaptable, highly conformal CMC-

Cu(II) membrane assembled in-situ on irregular surfaces of multi-substrates (i). Digital photographs of CMC-Cu(II) patterns (ii) on diverse 2D and 3D surfaces (iii) without additional transfer steps. **c** Cross-sectional SEM images of highly conformal CMC-Cu(II) membrane attached on PVC wavy mold. **d** Low impedance values of CMC-Cu(II) compared to commercial electrodes. **e** Biocompatibility of CMC-Cu(II) demonstrated by fluorescent imaging-based vitro cytotoxicity test. **f** Biodegradable characteristic of CMC-Cu(II) membrane.

Furthermore, we have carefully considered each of the reviewer #1's comments and addressed them as follows.

1. The authors may not understand the concept of electronic skin (e-skin). A basic electrode used for EEG measurements does not qualify as e-skin. E-skin typically refers to flexible materials that mimic the properties of human skin, enabling functionalities such as tactile sensing and stretchability. It is designed to provide a more comprehensive interface for various applications beyond simple electrical measurements. I recommend reviewing the definition and characteristics of e-skin to ensure that the manuscript accurately reflects this concept.

Author reply: Thanks very much for your careful review and valuable suggestions. We fully appreciate your concerns regarding the definition of "electronic skin (E-skin)." Traditionally, electronic skin has primarily focused on mimicking the multimodal sensory capabilities of human skin, such as tactile sensation, pressure detection, temperature responsiveness, and stretchability.

To gain a deeper understanding of the concept of electronic skin, we conducted a comprehensive review and synthesis of relevant literature. Some recent authoritative reviews and experimental studies have consistently categorized "E-skin," "epidermal electronics," and "on-skin electronics" as flexible device systems capable of conformal adhesion to the skin. These systems are widely applied in both bionic tactile sensing and continuous monitoring of physiological signals, including electrocardiography (ECG), electromyography (EMG), and electroencephalography (EEG). That is, in the medical and health field, flexible electrode-based physiological monitoring systems are thus recognized as forms of E-skin, as they exhibit essential characteristics such as mechanical flexibility, stretchability, and intimate skin contact, while also enabling an enhanced human-machine interface (e.g., D. Kim, et al, Science. 2011, 333(6044), 838-843;[4] C. H. Xu, et al, Matter. 2020, 2(6), 1414-1445;[5] J. C. Yang, et al. Adv. Mater. 2019, 31, 1904765;[6]).

In this paper, our work is based on flexible and adhesive electronic membranes designed for high-quality physiological signal acquisition. To avoid ambiguity, we have explicitly defined the research focus of this study as "epidermal electronic membrane" in the revised manuscript. We believe that this revision effectively clarifies the terminology usage and resolves potential ambiguities. We sincerely thank the reviewer for the constructive critique, which has significantly improved the alignment of our literature review and strengthened the conceptual clarity of our work.

Updates to the revised manuscript: According to Reviewer #1's comments, we revised the positioning of the CMC-Cu(II) electronic membrane in the Abstract section in the revised manuscript on Page 1.

> *"Epidermal electronics, which are flexible and conformable electronic systems designed to interact seamlessly with the human skin, hold great promise for healthcare monitoring and personal electronics. However, traditional fabrication methods face challenges of reliance on non-sustainable materials, intricate and time-consuming processes, and material softness-induced fragile transfer to target substrates. Inspired by the "milk skin" phenomenon, we developed a rapid "dipping-dipping" molecular assembly method to in-situ fabricate cellulose-based bio-skin within seconds, exhibiting ultrathin, high conformal, shape-customizable, degradable, and low impedance performances."*

References:

4. Kim D-H, et al. Epidermal Electronics. Science 333, 838-843 (2011).
5. Xu C, Yang Y, Gao W. Skin-Interfaced Sensors in Digital Medicine: from Materials to Applications. *Matter* **2**, 1414-1445 (2020).
6. Yang JC, Mun J, Kwon SY, Park S, Bao Z, Park S. Electronic Skin: Recent Progress and Future Prospects for Skin-Attachable Devices for Health Monitoring, Robotics, and Prosthetics. *Adv. Mater.* **31**, 1904765 (2019).

2. How do the authors control the thickness of the film? Thickness is crucial, as it directly influences the film's functionality. For instance, Figure 1d shows a thin 3.4 µm membrane, while other films appear significantly thicker, perhaps around hundreds of µm (e.g., Figures 1f, S7, and S8).

Author reply: As the reviewer pointed out, membrane thickness is indeed a critical parameter influencing its performance. In response, we have incorporated additional experimental data in the revised supplementary materials (**Supplementary Fig. 3**) to demonstrate that membrane thickness can be controlled by tuning the Cu(II) concentration during preparation. This is clearly illustrated in the newly added SEM cross-sectional images (**Supplementary Fig. 3**), which show a consistent increase in membrane thickness with increasing Cu(II) concentration.

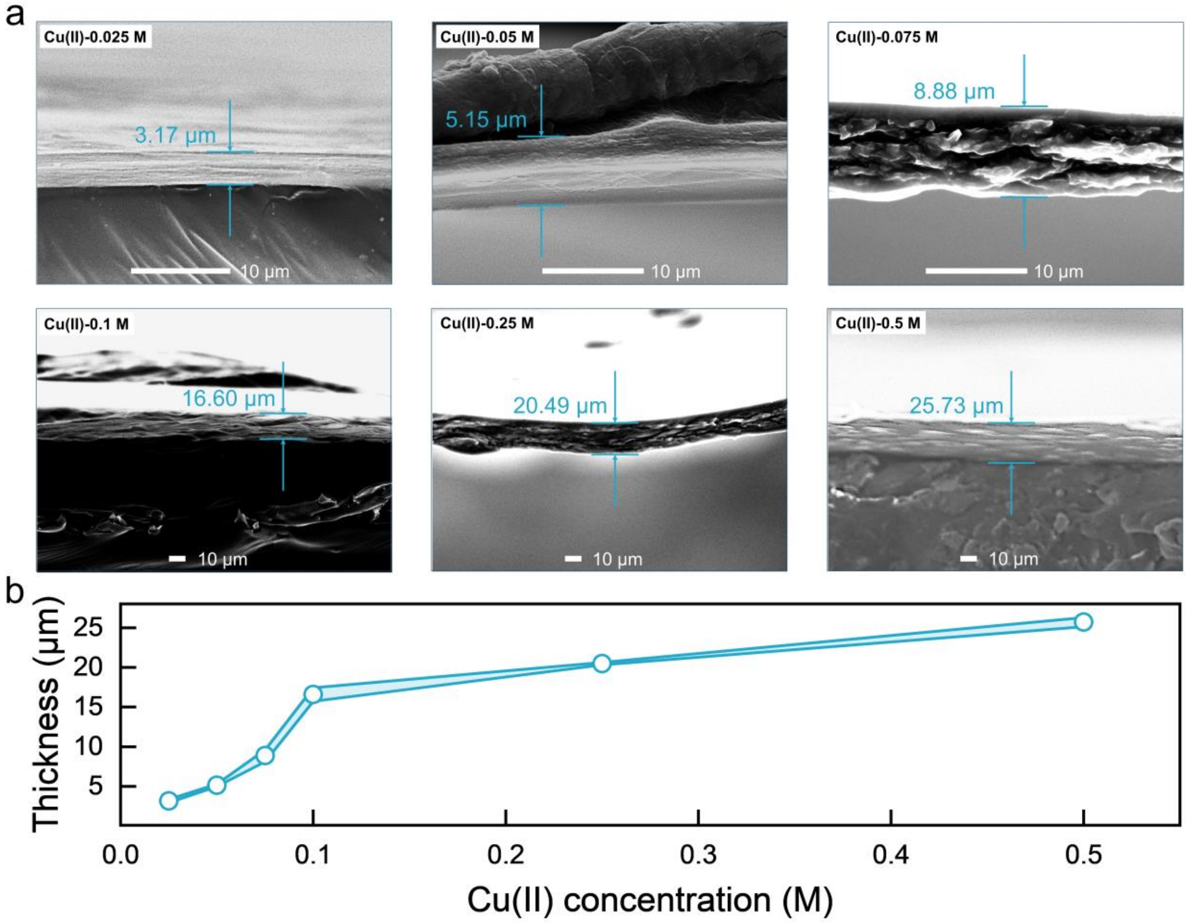

Supplementary Fig. 3. Control over the thickness of CMC-Cu(II) membranes. a Cross-sectional SEM images of CMC-Cu(II) membranes fabricated using varying concentrations of Cu(II). **b** A positive correlation between the thickness of the CMC-Cu(II) membrane and the concentration of Cu(II).

Regarding the thicker membranes presented in **Figure 1f, S7, and S8** in the original manuscript, we would like to clarify that these were intentionally selected to better visualize the overall morphology. Ultrathin membranes (only several micrometers thick) pose challenges in clearly resolving structural features in imaging, as evident from **Figure R7**.

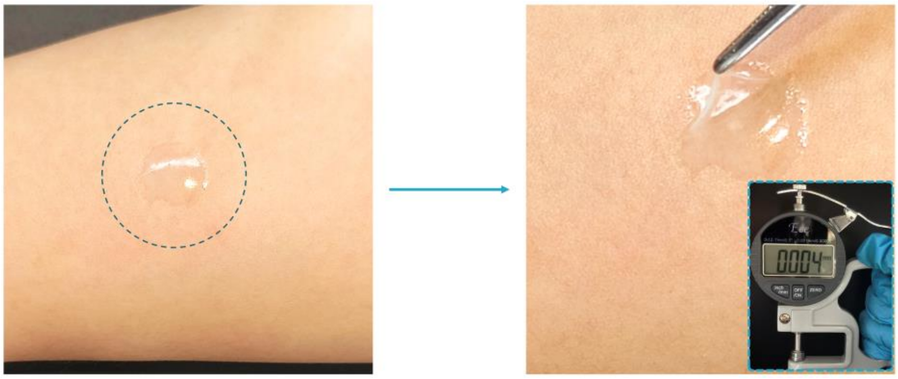

Figure R7. Digital images depicting the morphological characteristic and thickness measurement (insert) of the ultrathin CMC-Cu(II) membrane.

Updates to the revised manuscript: According to Reviewer #1's comments, we added the above results about controlling the thickness of CMC-Cu membrane on Page 6.

*"The cross-sectional scanning electron microscopy (SEM) image reveals a 3.4-µm-thick CMC-Cu (II) membrane (**Fig. 1d**) fabricated from a 1 wt% CMC solution and 0.5 M Cu(II) solution. The "thin" feature of the CMC-Cu(II) membrane further enhances its ability to conform to and match various interfaces[27, 28]. It is noteworthy that the thickness of the CMC-Cu(II) membrane can be tuned by varying the Cu(II) concentration. As clearly illustrated in the SEM cross-sectional images presented in **Supplementary Fig. 3**, the membrane thickness exhibits a positive correlation with rising Cu(II) concentration."*

3. The assertion of skin compatibility is overly simplistic. Figure S20 presents a misleading experiment. The red tint observed upon removing the "commercial electrode" results from adhesive removal, not an allergic reaction to the material. Furthermore, comparing materials with different cross-sectional areas is not fair. I observed some rash on the CMC-Cu electrode after application and removal. This experiment lacks rigor and should not lead to claims of "biocompatibility" based solely on photographs. A comprehensive long-term study is needed.

Author reply: Thank you for your critical and constructive comments. Your feedback is highly valuable in guiding us toward a more accurate and comprehensive evaluation of the material's biocompatibility. To address your concern, we have added in vitro cytotoxicity test to more rigorously validate the biocompatibility of the CMC-Cu(II) membrane.

In vitro cytotoxicity test showing biocompatibility of CMC-Cu(II)

To provide a more reliable assessment, we have performed in vitro cell culture experiments. In four parallel experiments, human umbilical vein endothelial cells (HUVECs) were co-cultured with the CMC-Cu(II) membrane for 24 hours. The results of fluorescence staining images from live/dead cell assays (see the revised **Supplementary Fig. 21**) demonstrate that the CMC-Cu membranes exhibit negligible cytotoxicity, thereby illustrating the good biocompatibility of the CMC-Cu(II) electrode. Detailed experimental processes and results are described in the Methods and Results sections of the revised manuscript. This experiment was carried out by the Key Laboratory of Biomedical Information Engineering of Ministry of Education, School of Life Science and Technology, Xi'an Jiaotong University.

To be noted, additional skin irritation data regarding skin contact is not provided here since that the editor requested not to perform skin irritation tests because this would make the study a clinical trial.

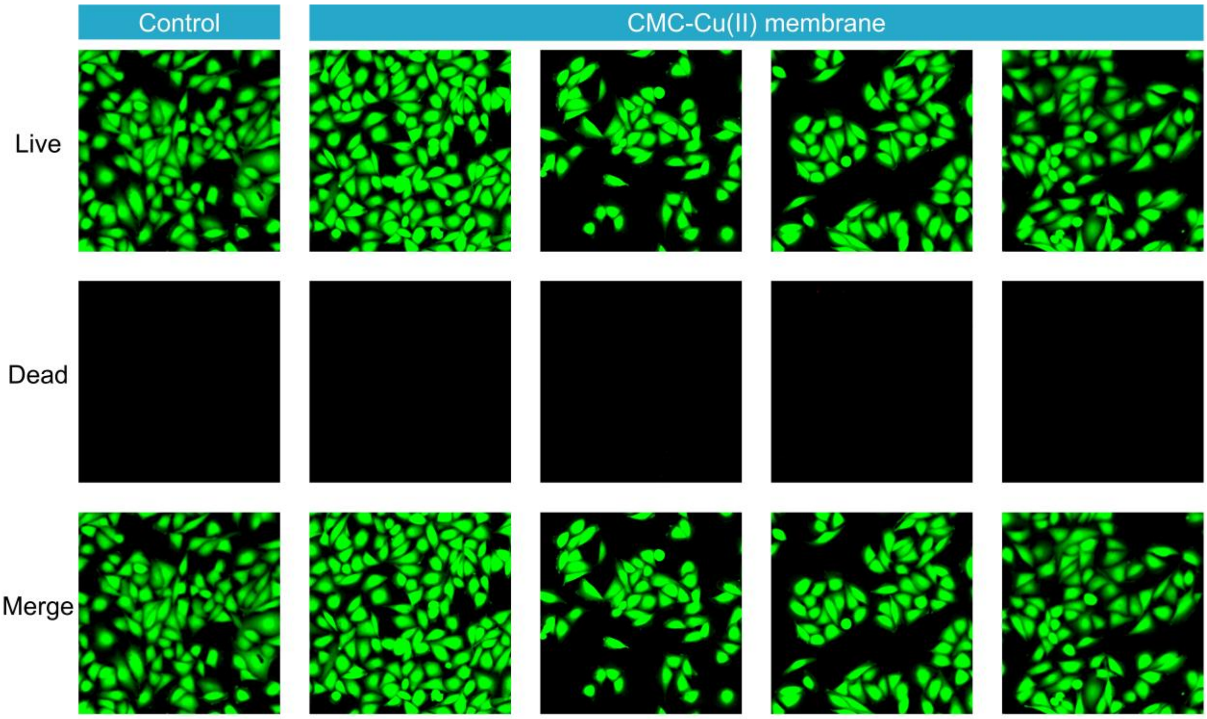

Supplementary Fig. 21. Biocompatibility evaluation of CMC-Cu(II) membrane. Representative fluorescence staining images showing live (green) and dead (red) HUVECs cells cultured without (Control) and with CMC-Cu(II) membrane (four parallel experiments).

Updates to the revised manuscript: According to Reviewer #1's comments, we have added the detailed experimental procedures about vitro cell culture experiment of CMC-Cu membrane in the revised manuscript on Page 28.

> *"**Biocompatibility test***
>
> *Human umbilical vein endothelial cells (HUVECs) were seeded in Dulbecco's modified Eagle's medium supplemented with 10% fetal bovine serum, 100 units/mL penicillin, and 100 µg/mL streptomycin. The cells were maintained under either blank control conditions or in the presence of a CMC-Cu(II) membrane (5 mm × 5 mm) in a humidified ncubator at 37°C with 95% humidity and 5% CO_2. After 24 h, the cells were gently washed twice with phosphate-buffered saline and then stained with Calcein AM and propidium iodide for 30 min at 37 °C. Following staining, samples were immediately visualized using a confocal laser scanning microscope[37]."*

Updates to the revised manuscript: We added the results about the biocompatibility test of CMC-Cu membrane in the revised manuscript on Page 16.

> *"Therefore, biocompatibility tests of the CMC-Cu(II) membrane were conducted. After culturing HUVECs for 24 hours, both the blank control group and the CMC-Cu(II) membrane exhibited excellent cellular compatibility (**Supplementary Fig. 21**). These findings suggest that the CMC-Cu(II) electrode employed in this study poses negligible cytotoxic risk, supporting its safe application in wearable bioelectronics requiring prolonged skin contact."*

4. The manuscript does not present the mechanical and electrical properties of the films. This information is critical for evaluating their performance.

Author reply: Thank you very much for highlighting this critical issue. We fully agree that evaluating both the mechanical and electrical properties of CMC-Cu(II) membrane is essential for a comprehensive performance assessment. In response to your suggestion, we have incorporated the following updates about mechanical and electrical properties of CMC-Cu(II) membrane in the revised manuscript:

- **Mechanical property:** We have added the tensile stress-strain curve of CMC-Cu(II) membrane and calculated an elongation at break (98.66%) and the fracture energy (2.4×10^6 J·m^{-3}) which reflects the material's toughness. The results demonstrate that the membrane exhibits sufficient flexibility and extensibility, making it suitable for skin-contact electronic devices under dynamic strain (**Supplementary Fig. 20**).

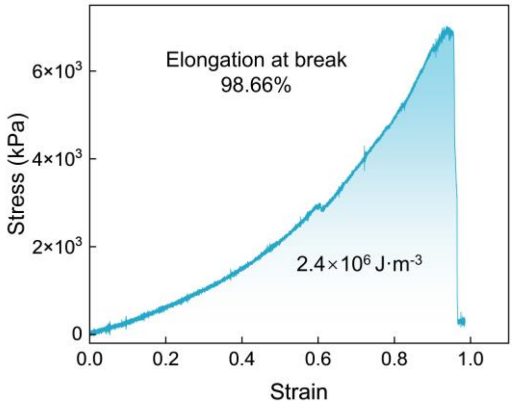

Supplementary Fig. 20. Stress-strain curve displaying the mechanical strength of CMC-Cu(II) membrane, along with the fracture elongation rate and fracture energy calculated from this curve.

Updates to the revised manuscript: According to Reviewer #1's comments, we added the above results and corresponding test method in the revised manuscript on Pages 16 and 27.

> *"For accurate monitoring, sensors must maintain conformal contact with the skin, deforming synchronously during use. Based on the stress-strain curve of the CMC-Cu(II) membrane, the fracture elongation is determined to be 98.66%, with a corresponding fracture energy (toughness) of 2.4×10^6 J·m^{-3} (**Supplementary Fig. 20**), demonstrating that the membrane possesses adequate flexibility and ductility for use as a skin-contact electrode in dynamic strain environments during physiological signal monitoring."*
>
> *"Characterization*
> *The thickness of CMC-Cu(II) membranes was characterized using Hitachi SU-1510 SEM to obtain cross-sectional characterization data. To assess the conformal properties, a CMC-Cu(II) membrane was attached onto a wavy PVC mold and subjected to embrittle in liquid nitrogen. 90° peeling test and stress-strain curves were measured on the electronic universal testing machine (Instron Corporation -3369)."*

- **Electrical property:** Based on the aforementioned simplified LCR circuit model of the electrode-skin interface (**Figure R2**) and the impedance calculation equation (Eq 1), impedance is demonstrated as the core parameter for evaluating the electrical performance of epidermal electrodes. To further evaluate the electrical property of CMC-Cu(II), we conducted a systematic re-evaluation. In addition to the existing impedance data, we supplemented measurements of the membrane's capacitance and resistance and compared these parameters with those of a commercial electrode. The CMC-Cu(II) electrode has the same surface area as the conductive hydrogel electrode employed in the commercial product. The impedance of CMC-Cu(II) was significantly lower than that of the commercial Ag/AgCl electrode (**Figure R8a**). At 20 Hz, the impedance of CMC-Cu(II) (9.75×10^4 Ω) was approximately one order of magnitude lower than that of the commercial electrode (6.04×10^5 Ω). Furthermore, the capacitance of CMC-Cu(II) (48.59 nF) was substantially higher than that of the commercial electrode (13.17 nF), while its resistance (173.23 Ω) was considerably lower than that of the commercial counterpart (683 Ω) (**Figure R8b**). These results collectively demonstrate that CMC-Cu(II) outperforms the commercial electrode as an epidermal electrode in terms of electrical performance, that is, the capabilities of physiological signal monitoring and acquisition.

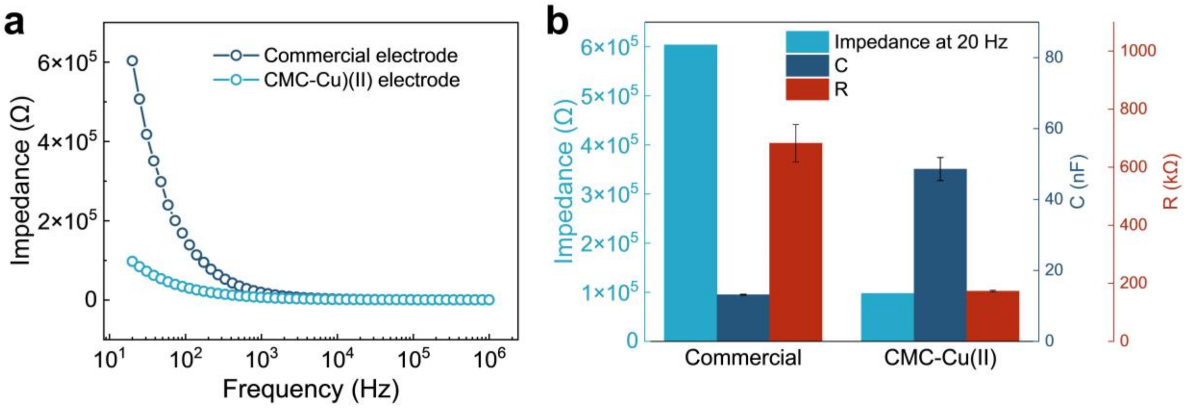

Figure R8. Electrical property of CMC-Cu(II) membrane as epidermal electrode. a Skin-contact impedance values of CMC-Cu(II) and commercial electrodes within 20 Hz to 1000 kHz. **b** Skin-contact impedance values at 20 Hz, capacitance, and resistance of CMC-Cu(II) and commercial electrodes.

The mechanical (**Supplementary Fig. 20**) and electrical performance (**Figure R9a and b**) data, along with corresponding analyses, have been incorporated into the revised manuscript and the supplementary information (**Figure R9a has been revised as Fig. 4b, and Figure R9b has been added as Supplementary Fig. 22.**). We believe these enhancements effectively address the reviewers' concerns and contribute to a more comprehensive evaluation of the fabricated membrane's performance.

Updates to the revised manuscript: According to Reviewer #1's comments, we added the above results in the revised manuscript on Page 16-17.

"The CMC-Cu(II) electrodes demonstrate lower impedance than commercial Ag/AgCl electrodes (Fig. 4b and Supplementary Fig. 22). Specifically, compared with the

corresponding commercial electrodes (6.04×10^5 Ω), the impedance of the CMC-Cu(II) electrodes (9.75×10^4 Ω) exhibits a notable reduction by approximately 84% at 20 Hz. Furthermore, the capacitance of CMC-Cu(II) (48.59 nF) is substantially higher than that of the commercial electrode (13.17 nF), while its resistance (173.23 Ω) is considerably lower than that of the commercial counterpart (683 Ω). Maintaining high interfacial capacitance and low resistance at the electrode-skin interface is crucial for minimizing bioelectrical signal attenuation and enhancing the overall signal-to-noise ratio (SNR)[15]. The superior electrical property of CMC-Cu(II) relative to commercial electrodes is attributed to its high ionic conductivity and conformal contact with the skin."

5. Can the membrane retain water? If not, how will performance (impedance, EEG, ECG) change over time? In my experience, CMC-metal gels tend to lose water rapidly in ambient environments.

Author reply: As pointed out by Reviewer #1, membrane dehydration significantly influences the performance of the CMC-Cu(II) membrane when used as an epidermal electrode. In our measurements, the electrodes were tested under sealed conditions, which substantially limited water evaporation compared to open-air environments and thereby maintained much better performance during the recording period. For further improvement of long-term hydration stability, incorporation of high-boiling-point solvents such as ethylene glycol (EG) could be considered, as discussed later.

To evaluate the membrane's hydration-dependent performance, we first established a standard test configuration, illustrated in **Figure R9a and b**. In this setup, the commercial electrode's conductive hydrogel was replaced with a CMC-Cu(II) membrane of equivalent area to ensure comparability. For clarity, this testing configuration (**Figure R9a**) has been included in **Supplementary Fig. 7** of the supplementary materials, and the corresponding descriptions have been added to the main manuscript. To examine dehydration behavior, water loss was monitored under both ambient (open) and testing (sealed) environments. The results indicate that while the CMC-Cu(II) membrane fully dehydrates within 3 hours when exposed to ambient air (**Figure R9c (top)**), it retains sufficient hydration for over 12 hours under operational testing conditions (**Figure R9c (bottom)**), demonstrating improved hydration stability in practical applications.

Updates to the revised manuscript: According to Reviewer #1's comments, we added the information of **Figure R9a** (**Supplementary Fig. 7**) in the revised manuscript on Page 7.

*"Additionally, the preparation of CMC-Cu(II) membrane with defined size and shape on the skin via the mask-forming method is illustrated in **Supplementary Fig. 7**."*

Additionally, under the test conditions, a series of electrical property measurements—impedance, capacitance, and resistance—were systematically conducted on the CMC-Cu(II) electrode every 3 hours. The impedance values at 20 Hz for the samples at 0 h, 3 h, 6 h, 9 h, 12 h, and 24 h were recorded as 9.75×10^4 Ω, 9.98×10^4 Ω, 9.99×10^4 Ω, 1.38×10^5 Ω, 1.36×10^5 Ω, and 2.21×10^5 Ω, respectively (**Figure R9d and e**). Both capacitance and resistance exhibited

consistent and expected trends in accordance with Eq. 1 (**Figure R9e**). The impedance of the CMC-Cu(II) electrode exhibited a slight increase within the first 6 hours, followed by a more substantial rise after 12 hours, and a more pronounced rise by 24 hours; however, it remained lower than that of the commercial electrode. This indicates that the physiological signal monitoring performance of the CMC-Cu(II) electrode experiences a very slight decrease within 6 hours, followed by a partial attenuation within 12 hours, and remains fully adequate for reliable, conventional physiological monitoring.

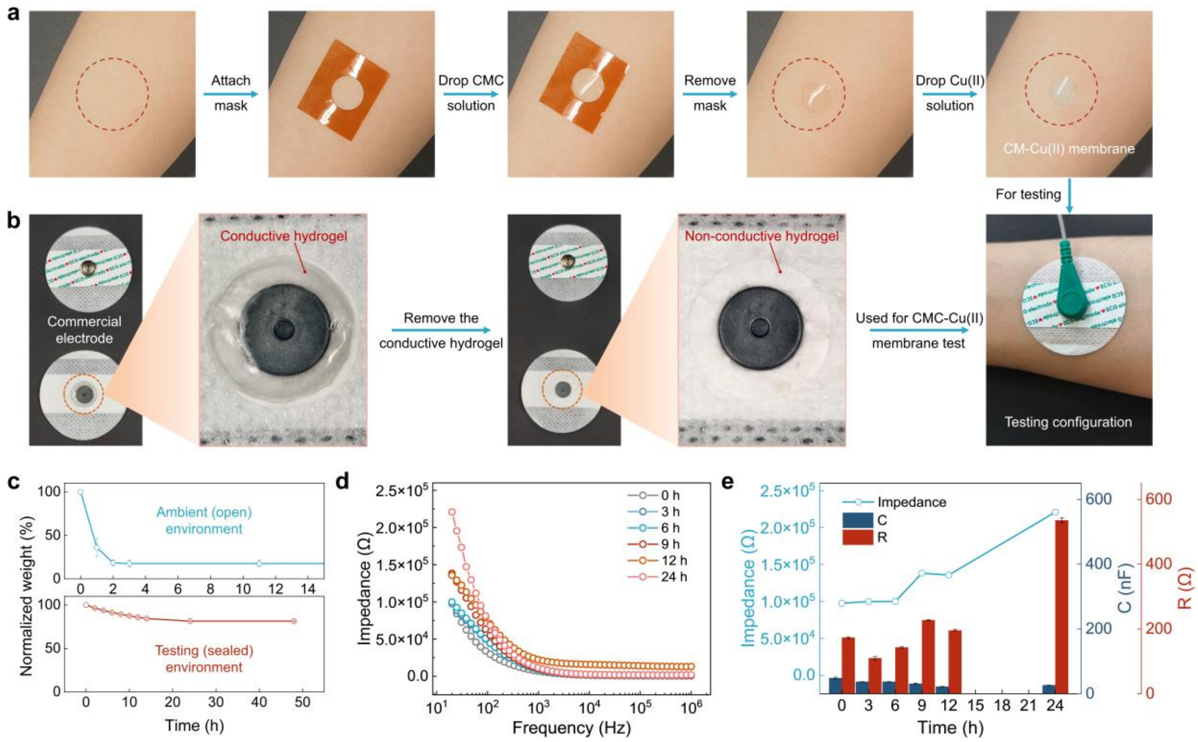

Figure R9. Schematic illustration of the physiological signal monitoring capability test for the CMC-Cu(II) membrane and its performance stability over time. **a** The formation of the CMC-Cu(II) membrane on the skin using the mask-forming method. **b** The test configuration of the CMC-Cu(II) membrane on the skin. **c** Normalized water loss data of the CMC-Cu(II) membrane under ambient and test environments. **d** Skin-contact impedance values within 20 Hz to 1000 kHz of CMC-Cu(II) electrode vary with time. **e** Skin-contact impedance value at 20 Hz, capacitance, and resistance of CMC-Cu(II) electrodes vary with time.

To address the issue of water loss in the CMC-Cu(II) membrane, we further propose replacing water with ethylene glycol (EG), a solvent with a higher boiling point, to enable the long-term and continuous operation of the CMC-Cu(II) electrode. As shown in **Figure R10a**, a conformal conductive CMC-Cu(II) membrane can also be fabricated via the dipping-dipping method using ethylene glycol (EG) as the solvent. The weight change experiment demonstrated that the CMC-Cu(II) membrane prepared with EG exhibited negligible mass variation, showing no significant loss and even a slight increase, over 24 hours (**Figure R10b**). This minimal change may be attributed to moisture absorption from the ambient environment, which is positive for its electrical performance. As anticipated, the CMC-Cu(II) membrane formulated with EG achieved an impedance value comparable to that of the aqueous-based counterpart. Moreover,

its impedance remained stable over the entire 24-hour period without measurable degradation. At 20 Hz, the impedance values at 0 h, 3 h, 6 h, 9 h, 12 h, and 24 h were recorded as 9.53×10^4 Ω, 9.53×10^4 Ω, 9.58×10^4 Ω, 9.68×10^5 Ω, 9.63×10^5 Ω, and 9.57×10^5 Ω, respectively. Both capacitance and resistance values maintained consistent stability throughout the testing period (**Figure R10c and d)**. These results confirm the effectiveness and feasibility of employing EG as an alternative solvent in this system.

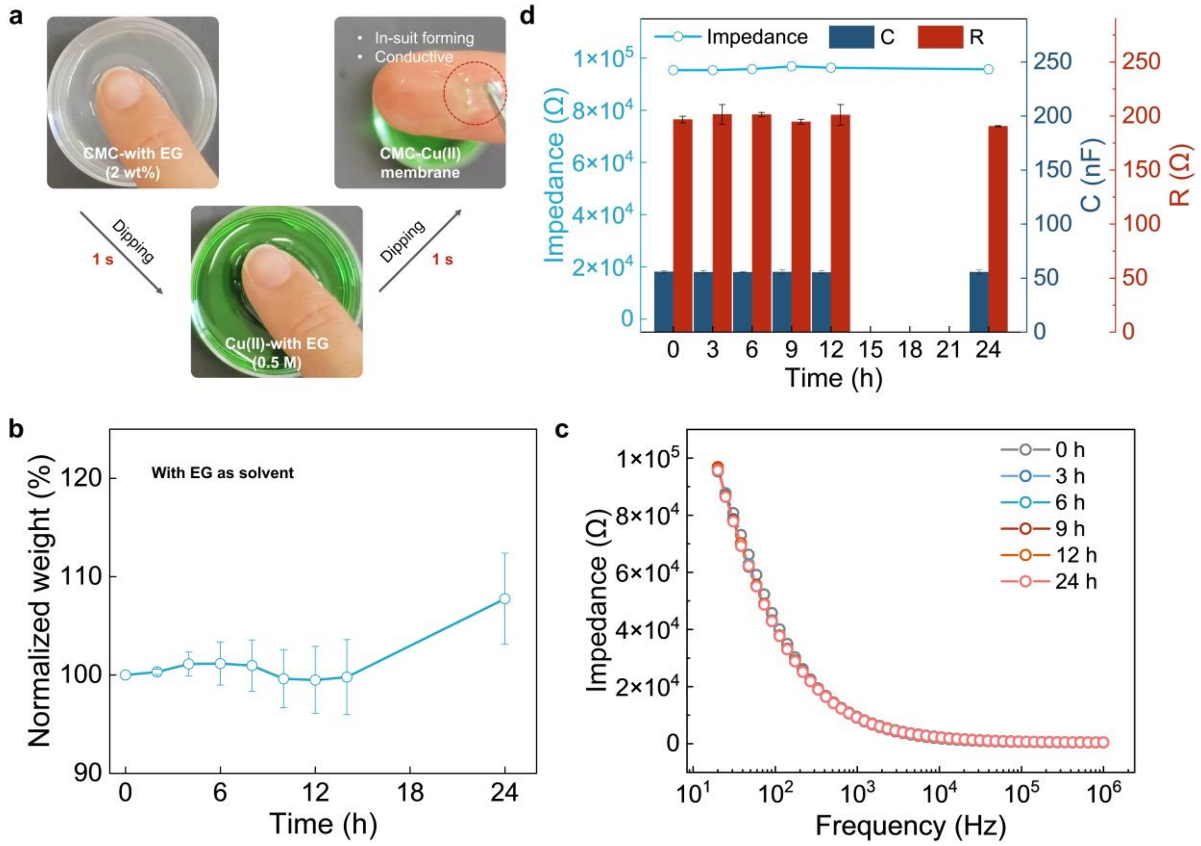

Figure R10. Preparation and electrical performance of CMC-Cu(II) membranes using ethylene glycol (EG) replacing water as the solvent. a Digital photographs of CMC-Cu(II) membrane with EG as solvent fabricated by simple "dipping-dipping" process. **b** Normalized weight loss data of the CMC-Cu(II) membrane with EG as solvent under testing environments. **c** Skin-contact impedance values within 20 Hz to 1000 kHz of CMC-Cu(II) electrode with EG as solvent vary with time. **d** Skin-contact impedance values at 20 Hz, capacitance, and resistance of CMC-Cu(II) electrode with EG as solvent vary with time.

Based on the above electrical behavior, the physiological signal monitoring performance of CMC-Cu(II) electrodes with water and EG as solvents was evaluated for electrocardiogram (ECG) signal acquisition after a 3-hour period. The ECG signal with the highest amplitude indicates that the CMC-Cu(II) electrode prepared with water as solvent exhibits slight signal attenuation after 3 hours, yet retains its functional integrity. Upon replenishing the electrode with a drop of water to this electrode, the signal amplitude is partially restored. In contrast, the CMC-Cu(II) electrode fabricated with EG as the solvent maintains a stable and consistent signal amplitude throughout the 3-hour duration. This superior stability is further corroborated by the SNR result (**Figure R11)**.

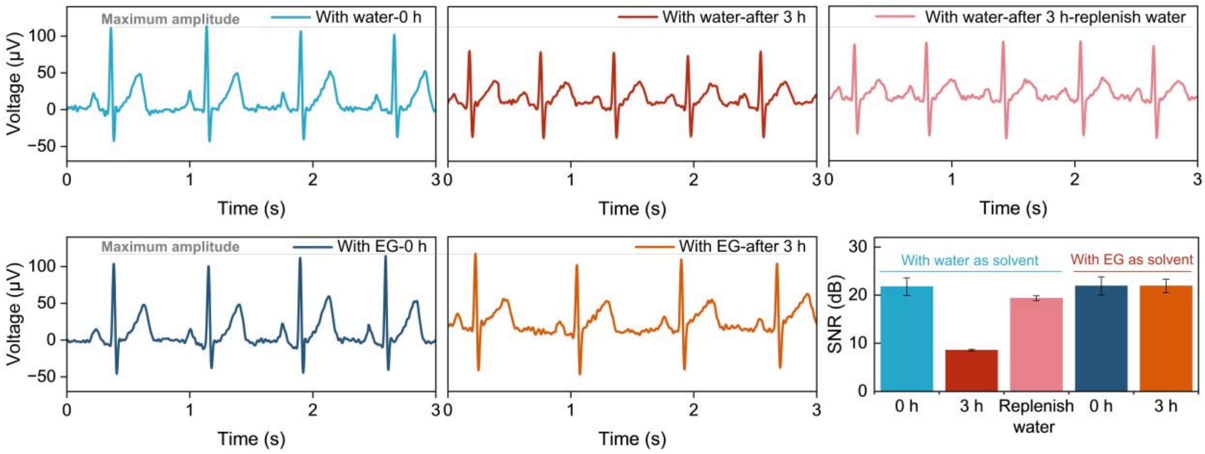

Figure R11. The periodic ECG signals and SNR of CMC-Cu(II) with water and EG as solvents acquired at 0 h and after 3 h. Note: "With water-after 3 h-replenish water" is a process in which a drop of water is reintroduced onto the CMC-Cu membrane after 3 h, followed by continued signal recording.

To support the above conclusion of ECG monitoring, the electromyographic (EMG) monitoring performance of CMC-Cu(II) electrodes using water and EG as solvents was evaluated after 3 hours. Two experimental electrodes were placed on each of the four muscle groups on the subjects' arms: brachioradialis, flexor carpi radialis, biceps brachii, and triceps brachii, corresponding to Ch1, Ch2, Ch3, and Ch4, respectively. A reference electrode was positioned at the elbow region, an area devoid of significant muscular activity. Muscle electrical signals were recorded during two distinct sets of badminton-playing movements. For the CMC-Cu(II) electrode with water as the solvent, signal noise increased after 3 hours; however, distinct EMG signals corresponding to different muscles and actions remained detectable. In contrast, the CMC-Cu(II) electrode using EG as the solvent exhibited negligible signal variation over the same period and maintained high-fidelity signal acquisition throughout. These EMG findings, consistent with the previously observed ECG monitoring results, collectively demonstrate the effectiveness of our proposed solution in addressing the dehydration issue associated with CMC-Cu(II) membrane (**Figure R12)**.

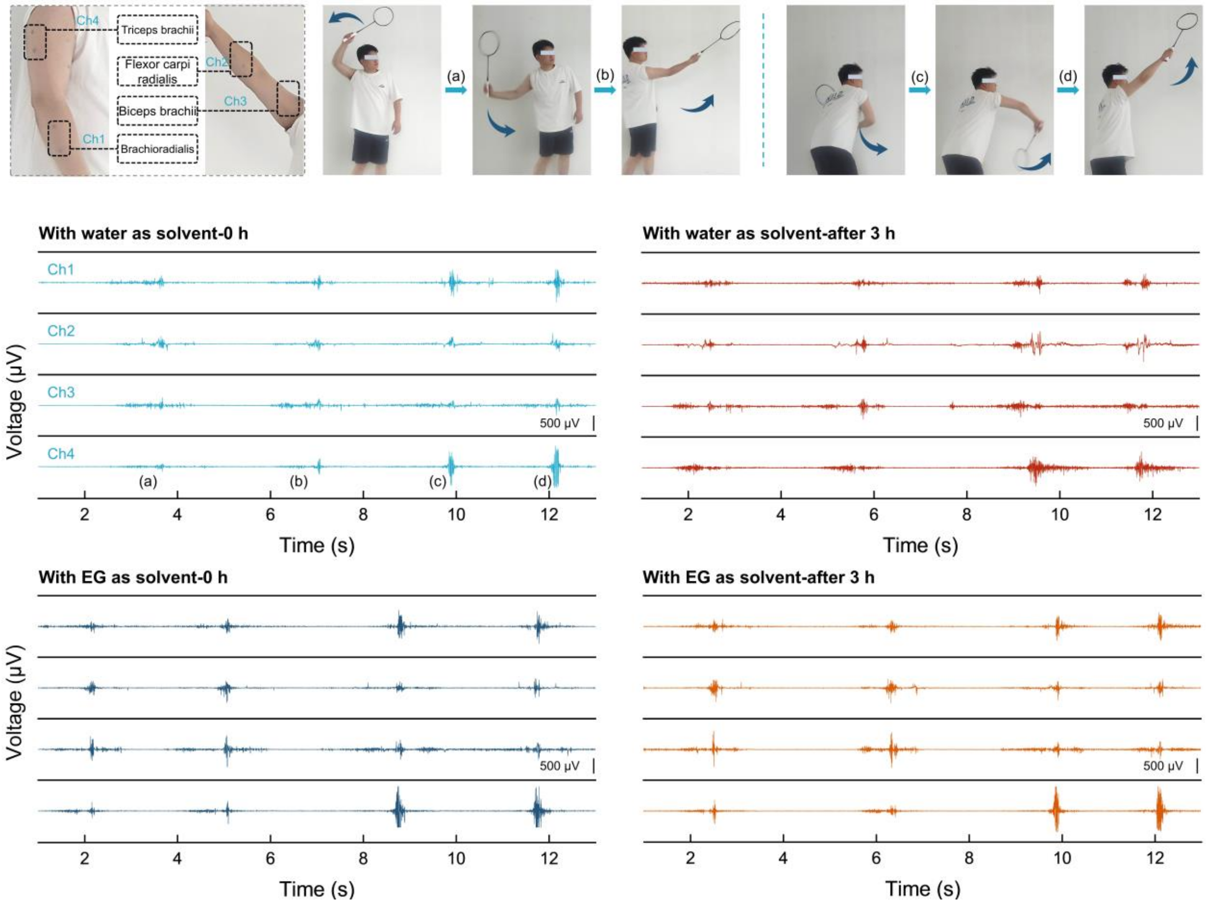

Figure R12. Schematic illustration of electrode placement on different arm muscles during EMG collection and a series of arm movements of badminton-playing during the acquisition of arm EMG signals (top); EMG signals corresponding to the series of arm movements of the subject captured by the CMC-Cu(II) with water (middle) and EG (bottom) as solvent at 0 h and after 3 h.

6. Additionally, in Figure S9, what happens to the material's functionality when submerged over time? I believe both conductivity and structural integrity will vary with submersion duration. Please provide quantification.

Author reply: The structural integrity and electrical properties of the CMC-Cu(II) membrane in aqueous environment were quantitatively characterized over time. Digital images and normalized membrane area measurements revealed a gradual contraction of the membrane, with the membrane area decreasing to 45.19% of its initial value after 30 days (**Figure R13a**). Since the structural formation of the CMC-Cu(II) membrane relies on the strong coordination interaction between CMC and Cu(II), to further verify its structural integrity, UV spectroscopy was employed to analyze both aqueous Cu(II) solutions and the soaking water collected after immersing the CMC-Cu(II) membrane. The results reveal a distinct absorption peak at 194 nm in the UV spectrum of Cu(II), whereas no such absorption peak is observed in the soaking water even after 30 days of immersion (**Figure R13b**), that is, there is no release of Cu(II). This indicates that, under aqueous conditions, the CMC-Cu(II) membrane undergoes apparent morphological contraction but no collapse of the internal structure.

Electrical performance analysis showed that the impedance values at 20 Hz remained stable during the first 24 hours, measured as 9.75×10^4 Ω, 9.99×10^4 Ω, 9.64×10^4 Ω, 9.96×10^4 Ω, 9.68×10^4 Ω, and 9.97×10^4 Ω at 0 h, 3 h, 6 h, 9 h, 12 h, and 24 h, respectively, indicating minimal change within the first day. Over the subsequent week, the impedance gradually increased. At 3 d, 5 d, and 7 d, the impedance values at 20 Hz were recorded as 1.04×10^5 Ω, 1.16×10^5 Ω, and 1.29×10^5 Ω, respectively, demonstrating only slight functional degradation (**Figure R13c and d**).

In the original manuscript, the aqueous stability tests were conducted to demonstrate the strong coordination between CMC and Cu(II), showing that the membrane could maintain its integrity even under prolonged immersion. In practical use, however, the electrode is prepared on demand and used immediately as a disposable device, without scenarios requiring long-term immersion in water. Therefore, although the electrode exhibits good performance in aqueous environments, we have decided to remove the corresponding figure and related analyses from the revised manuscript and supplementary materials to avoid potential misinterpretation of the data.

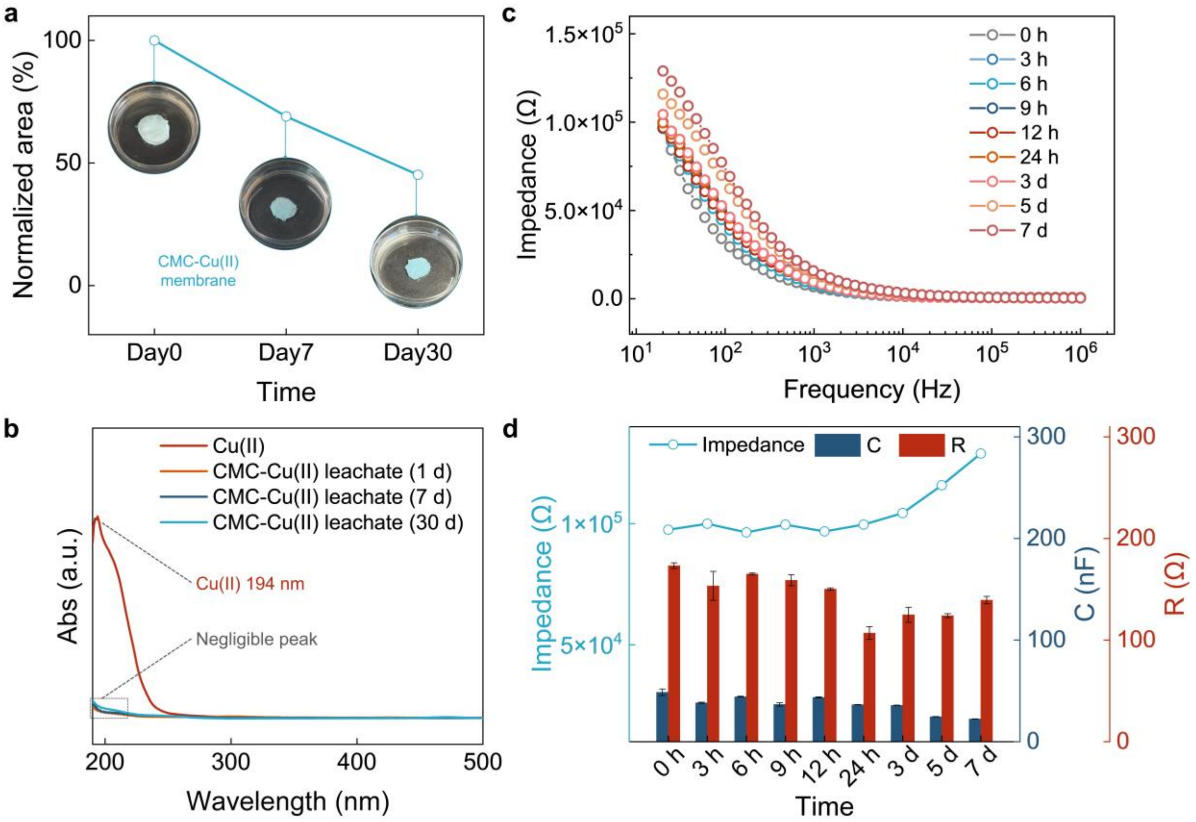

Figure R13. The apparent morphology and electrical properties of the CMC-Cu(II) membrane in aqueous environment over time. a The apparent morphology and normalized membrane area of CMC-Cu(II) membrane in aqueous environment within 30 days. **b** UV absorption spectra of Cu(II) and soaking water collected after immersing the CMC-Cu(II) membrane at day 1, day 7 and day 30. **c** Skin-contact impedance values within 20 Hz to 1000 kHz of CMC-Cu(II) membrane in aqueous environment over a period of 7 days. **d** Skin-contact impedance values at 20 Hz, capacitance, and resistance of CMC-Cu(II) membrane within 7

days.

7. In Figure S4, the claim that CMC molecules in support channels induced by Cu(II) coordination bonds enhance ion transport lacks clarity. A more detailed explanation is needed.

Author reply: In response to Reviewer #1's suggestion, we have revised and expanded the explanation of the conductive mechanism underlying the enhanced ion transport via the support channels formed by Cu(II) coordination bonds with CMC, thereby improving its clarity and comprehensibility. Furthermore, we have updated the corresponding mechanism figure (**Supplementary Fig. 5**) accordingly. For a comprehensive discussion, please refer to the updates to the revised manuscript.

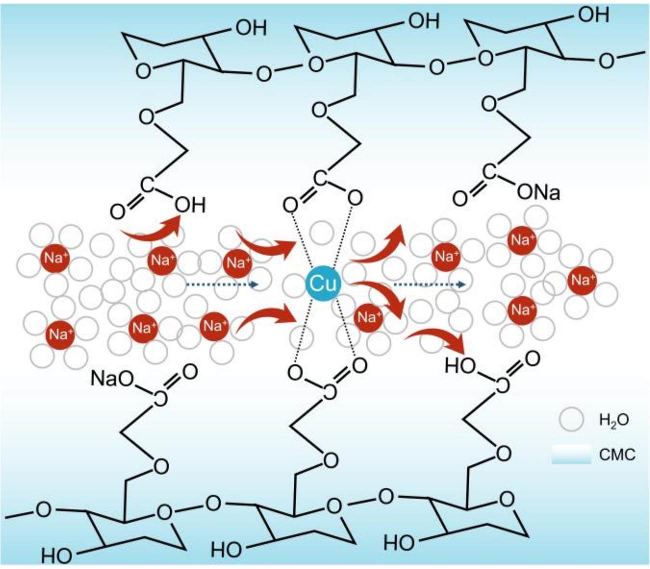

Supplementary Fig. 5. Schematic illustration of the assembly of CMC molecules into support channels induced by Cu(II) coordination bonds in CMC-Cu(II) membrane to facilitate ion transport and consequently enhance electrical conductivity. The blue dashed arrow indicates that Na(I) ions migrate together with water molecule clusters, and the red arrows indicate Na(I) and free water molecule transport via polar group sites (especially Cu(II))[1].

Updates to the revised manuscript: According to Reviewer #1's comments, we revised and expanded the explanation of the conductive mechanism in the revised manuscript on Page 6-7.

> *"The CMC-Cu(II) membrane exhibits excellent conductivity, enabling its effective use as a conductive pathway or epidermal electrode. In the CMC-Cu(II) membrane, coordination bonds between CMC and Cu(II) promote molecular aggregation while simultaneously forming ion channels that facilitate ion transport. In typical CMC structure, hydrogen ions at the carboxymethyl group on the C6 position are partially substituted by Na(I) ions. The incorporation of Cu(II) through coordination displaces Na(I) ions from their original sites, rendering them free and mobile within the system. Furthermore, water molecules in the membrane system are partially chelated with Cu(II), while some of the remaining free water molecules, acting as strong Lewis bases, form a solvation layer around Na(I) ions. These solvated Na(I) and unbound water molecules reside within the open ion transport channels of the CMC-Cu(II) network, allowing for*

*efficient and directional ion mobility. This enhanced ionic mobility significantly improves the conductivity of the hydrated CMC-Cu(II) membrane (**Supplementary Fig. 5**)[29].”*

8. The explanation regarding Figure S14—suggesting that a negative UV absorption peak in CMC-Ca(II) is due to weak electrostatic attractions or complexation—seems questionable. Did the authors observe this phenomenon visually? Was the calcium fully dissolved in the solution?

Author reply: To investigate whether the observed negative ultraviolet absorption peak in CMC-Ca(II) arises from the formation of microscopic aggregates or invisible precipitates due to weak coordination interactions between Ca(II) and CMC, we conducted a controlled experiment. A Ca(II) solution with the same concentration (0.5 M) as that used for Cu(II) was prepared and ensured to be fully dissolved. Using the identical “dipping-dipping” method and 2 wt% CMC solution, CMC-Ca(II) samples were fabricated. After 20 cycles, slight turbidity began to appear in the CMC solution; following 200 cycles, the turbidity became more pronounced (**Supplementary Fig. 14**). This progressive increase in turbidity is attributed to the cumulative residual CMC in the Ca(II) solution, which enhances the weak coordination interaction between Ca(II) and CMC over repeated operations. These observations support the explanation provided in the manuscript regarding the origin of the negative UV absorption feature. To enhance clarity, the relevant discussion has been revised accordingly in the updated manuscript.

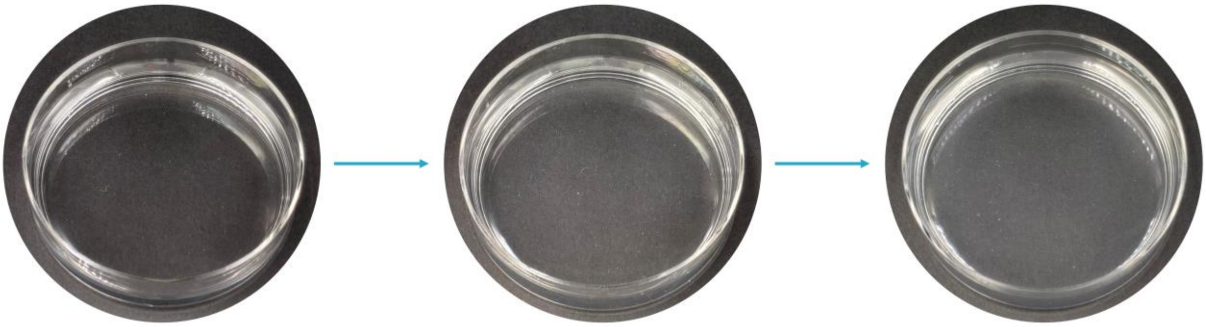

Supplementary Fig. 14. Digital photos of Ca(II) solution after 20 and 200 cycles of the “dipping-dipping” process.

Updates to the revised manuscript: We revised the explanation of the negative UV absorption peak of CMC-Ca(II) in the revised manuscript on Page 8-9.

“This phenomenon may be attributed to the weak coordination between Ca(II) and CMC, leading to the formation of microscopic aggregates or invisible turbidity. When the size of these aggregates approaches or exceeds the wavelength of incident light, significant light scattering occurs. This scattered light may compensate for, or even surpass, the attenuation of direct transmitted light caused by both scattering and absorption, resulting in the observation of a negative absorption peak. A Ca(II) solution with the same concentration (0.5 M) as that used for Cu(II) was prepared and ensured to be fully dissolved. Using the identical “dipping-dipping” method and 2 wt% CMC solution, CMC-Ca(II) samples were fabricated. After 20 cycles, slight turbidity began to appear in the

*CMC solution; following 200 cycles, the turbidity became more pronounced (**Supplementary Fig. 14**). This progressive increase in turbidity is attributed to the cumulative residual CMC in the Ca(II) solution, which enhances the weak coordination interaction between Ca(II) and CMC over repeated operations."*

9. The caption for Figure S15 appears to be incorrect, as there are no graphs for CMC-Ca(II) over different time intervals.

Author reply: Thank you for your thorough review, and we have accordingly revised the caption of **Supplementary Fig. 15** in revised manuscript as follows.

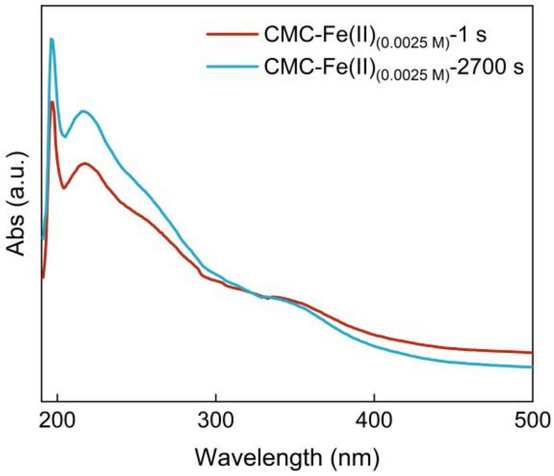

Supplementary Fig. 15. The UV curves of CMC-Fe(III) with different reaction time. The absorbance of the CMC-Fe(II) peak increases with prolonged reaction time.

10. Is there any mechanical characterization of MC and CMCH with Cu(II) (or other ions)? The manuscript lacks explanation for these materials.

Author reply: We sincerely thank Reviewer #1 for the insightful suggestion. In response, we have conducted tensile tests on MC-Cu(II) and CMCH-Cu(II) membranes, and the corresponding stress–strain curves are provided in **Figure R14**. The results show that the fracture elongation values for MC-Cu(II) and CMCH-Cu(II) are 69.75% and 23.71%, respectively, and their fracture energies are 2.82×10^5 J·m^{-3} and 8.51×10^3 J·m^{-3}. These results indicate that the mechanical properties of MC-Cu(II) are close to those of CMC-Cu(II), whereas CMCH-Cu(II) exhibits relatively different behavior. The relatively weak mechanical properties of the CMCH-Cu(II) membrane can be elucidated by its molecular structure characteristics. (i) The regularity of the main chain: the main chain of CMCH features the presence of amino or N-carboxymethyl groups at the C2 position. This presence causes significant steric hindrance, resulting in a reduction in the regularity of the main chain and a looser packing, thus inherently weakening its mechanical support. (ii) The uniformity of functional groups and coordination networks: CMCH contains carboxyl, hydroxyl, and amino (or their derivatives) concurrently. Different functional groups possess different coordination competitiveness for Cu(II), which readily leads to local non-uniformity of the coordination network and the formation of mechanical weak points.

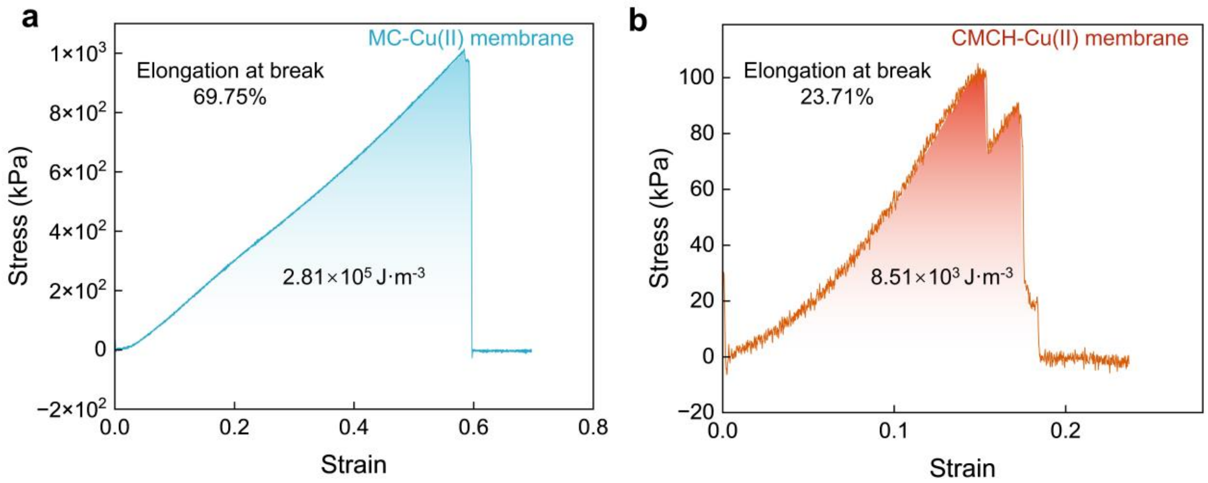

Figure. R14. Stress-strain curves of displaying the mechanical strength of MC-Cu(II) (**a**) and CMCH-Cu(II) (**b**) membranes, along with the fracture elongation rate and fracture energy calculated from these curves.

11. As a reader not specialized in physiological monitoring, I find Figures 4, 5, S21, and S22 unclear. There is no explanation of the significance of the peaks and time-frequency spectrum. Specifically, in Figure S21, what do the authors mean by "EEG monitoring during mathematical calculation by CMC-Cu(II) electrodes"?

Author reply: We recognize that readers without a background in physiological signal research may find **Figure 4, 5, S21, and S22** in the original manuscript lacking sufficient explanatory context. In response to your constructive feedback, we have implemented the following revisions in the revised manuscript:

- **Supplementary signal interpretation:** We have enhanced the main text and figure legends with detailed explanations of the physiological significance of key features in the signals. Specifically, for the ECG waveform, we now clearly label and describe the cardiac electrical events associated with the PQRST waveform (**Fig. 4c**). For the EEG spectra, we provide interpretations of major frequency bands (e.g., α and β waves) and discuss their modulation under specific task conditions. Furthermore, we have included a more comprehensive description of the time-frequency analysis method to improve accessibility. These additions enable non-specialist readers to grasp the essential signal characteristics more easily.

- **Clarification of expression:** The caption of **Supplementary Fig. S21** in the original manuscript has been revised from "EEG monitoring during mathematical calculation by CMC-Cu(II) electrodes" to "EEG signals recorded by CMC-Cu(II) during the participants' mathematical calculation tasks." This revision improves clarity and eliminates potential ambiguity.

These revisions are intended to enhance the clarity and interpretability of the results shown in

Fig. 4, 5, S21, and S25 for a broader, interdisciplinary audience.

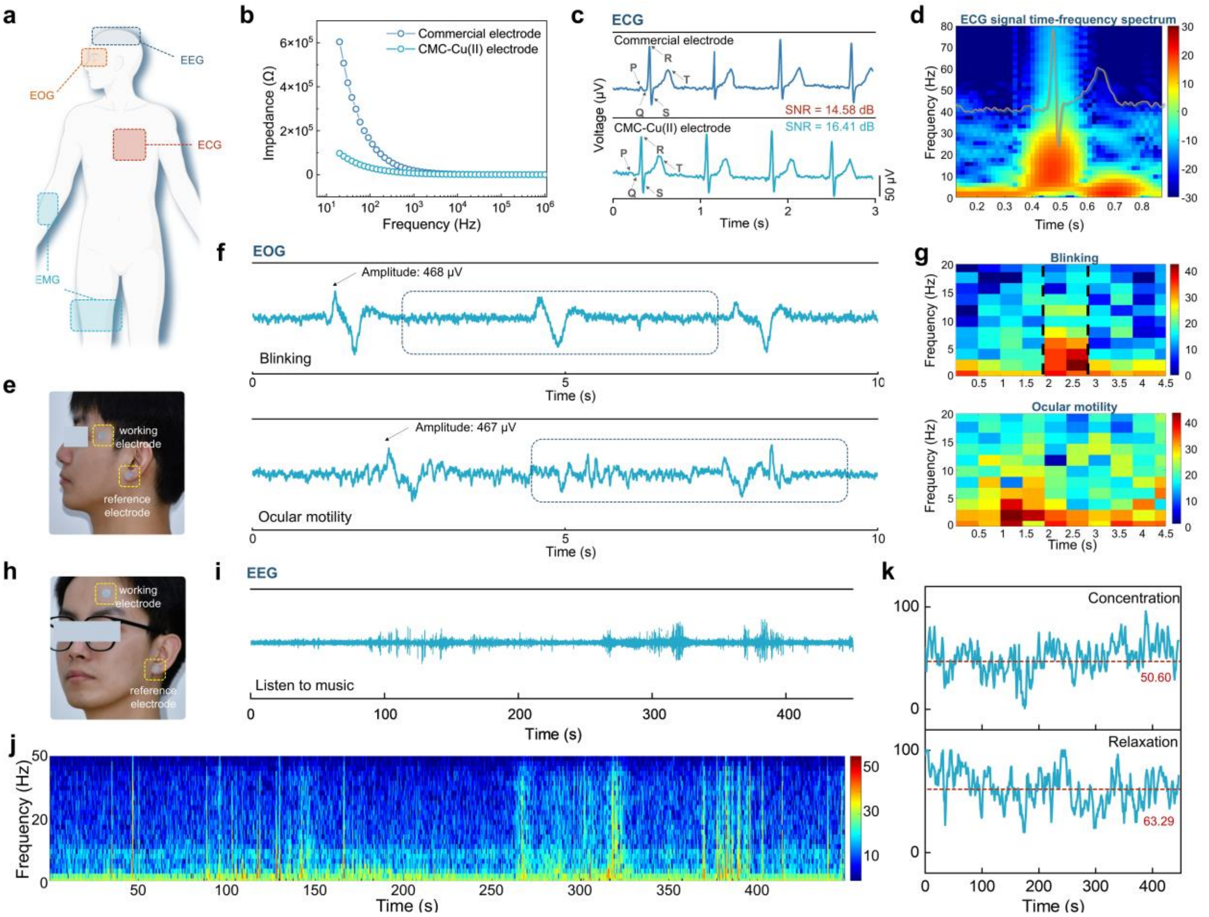

Fig. 4: High-fidelity monitoring of ECG, EOG, and EEG by the CMC-Cu(II) epidermal electrode. a Schematic diagram of ECG, EMG, EOG, and EEG monitoring. **b** Skin-contact impedance values of CMC-Cu(II) and commercial electrodes within 20 Hz to 1000 kHz. **c** The periodic ECG signals and SNR of CMC-Cu(II) and commercial electrodes. **d** Time-frequency spectrum of a single cycle of ECG signal. **e** Schematic illustration of electrode placement during EOG collection. **f and g** Periodic EOG signals captured by the CMC-Cu(II) electrodes displaying movements involving blinking and ocular motility of the subject (**f**) and corresponding time-frequency spectrum of a single cycle of EOG signal (**g**). **h** Schematic indication of electrode placement during EEG collection. **i** to **k** EEG signals (**i**), corresponding time-frequency spectrum (**j**), and coefficients of concentration and relaxation (**k**) recorded by CMC-Cu(II) electrodes during the subject's cognitive engagement in music listening.

Updates to the revised manuscript: According to Reviewer #1's comments, we revised and added above content about enhancing the clarity and interpretability of ECG, EOG, EEG, and EMG in the revised manuscript on Pages 17-23.

About ECG, Pages 17-18:

> *"Subsequently, circular CMC-Cu(II) electrodes (15 mm diameter) are applied in a classical three-lead ECG setup following the "Einthoven triangle" theory. From the collected ECG signals (**Fig. 4c**), both commercial and CMC-Cu(II) electrodes exhibit clear and characteristic PQRST waveforms. The P wave corresponds to atrial*

*depolarization, representing the electrical activation preceding atrial contraction; the QRS complex reflects ventricular depolarization, which initiates the primary electrical event driving cardiac pumping; and the T wave indicates ventricular repolarization, marking the recovery of the ventricles to their resting state. These waveform components serve as fundamental parameters in the clinical assessment of cardiac rhythm and function. When compared with the commercial electrodes, the CMC-Cu(II) electrodes not only capture ECG signals with identical waveforms but also achieve a higher SNR of 16.41 dB compared to 14.58 dB. Further time-frequency[33] analysis enables precise interpretation of ECG signals by revealing the frequency distribution characteristics of different waveforms. The P wave is typically concentrated in the 0.5–10 Hz range, exhibiting a brief low-frequency energy peak; the QRS complex spans a broader frequency spectrum from 10 to 40 Hz, reflecting high-frequency components associated with ventricular depolarization; the T wave generally falls within the same 0.5–10 Hz range as the P wave but demonstrates higher energy density. By employing short-time Fourier transform, time-frequency analysis simultaneously captures signal details in both temporal and spectral domains. This approach not only facilitates understanding of the electrophysiological behavior of a normal heart but also supports the detection of abnormalities such as arrhythmias, while providing a reliable foundation for ECG signal denoising and feature extraction. A complete cycle of the ECG signal acquired by CMC-Cu(II) is extracted, and its corresponding time-frequency spectrum (**Fig. 4d**) is obtained via the Fourier transform. In the resulting spectrogram, the horizontal axis represents time, reflecting the temporal evolution of the ECG signal; the vertical axis denotes frequency, indicating the distribution of signal energy across different frequency bands. The color bar reflects the signal power or energy: colors approaching deep red indicate higher energy levels at specific time-frequency points. This time-frequency spectrum demonstrates that the ECG signal acquired by the CMC-Cu(II) electrode exhibits strong consistency and accuracy with the characteristic features of a typical electrocardiogram, thereby indicating its reliable performance in physiological signal detection."*

About EOG, Page 19:

*"The application of the CMC-Cu (II) electrode in EOG monitoring as horizontal unipolar leads is demonstrated in **Fig. 4e**. Single CMC-Cu(II) electrodes at the outer corner of the eye capture periodic EOG signals, reflecting blinking and ocular motility[34]. For blinking, the CMC-Cu(II) electrode captures distinct voltage patterns for each blink event, with localized amplification illustrating precise signal characteristics (**Fig. 4f top**). The time-frequency spectrum derived from the Fourier transform of the EOG signal reveals detailed signal characteristics, thereby further substantiating its stability and accuracy (**Fig. 4g top**). Similarly, during ocular motility tests, the CMC-Cu(II) records persistent pattern of EOG signals corresponding to eye movements that displays distinct difference from the intermittent independent signal of blinking, with localized amplification and time-frequency spectrum analysis revealing detailed signal patterns (**Fig. 4f bottom and Fig. 4g bottom**). The above results align with the peak patterns observed in the subjects' blinking and ocular motility behaviors, which are recorded using commercial electrodes (**Supplementary Fig. 24**). Based on the amplitude of the acquired signals, the signal peak*

monitored by the CMC-Cu(II) electrode is slightly higher than that of the commercial electrode, demonstrating its capability to capture high-fidelity ocular signals."

About EEG, Pages 19-21:

*"Unipolar lead EEG collection offers a simple and efficient approach for analyzing electrical activity of specific brain regions. EEG signals can be categorized into distinct frequency bands based on their specific ranges, with each band corresponding to particular physiological and cognitive states. The δ wave (0.5–4 Hz) is predominantly observed during deep sleep and reflects the brain's restorative and recovery processes. The θ wave (4–8 Hz) is associated with light sleep, relaxation, or meditative states and is often enhanced during certain cognitive processing and memory-related tasks. The α wave (8–13 Hz) is most prominent during a relaxed, awake state with eyes closed, indicating cortical inhibition and relaxation; it diminishes significantly upon eye opening or when attention is actively engaged. The β wave (13–30 Hz) is linked to active thinking, concentration, anxiety, and arousal, reflecting the brain's engagement in information processing and task execution. The γ wave (>30 Hz) is closely associated with higher-order cognitive functions, including learning, perception, and multisensory integration, and becomes particularly evident in tasks requiring rapid coordination across distributed brain regions. Analyzing these frequency bands enables the characterization of dynamic brain activity across various cognitive states and behavioral tasks, offering critical insights for neuroscience research and practical applications such as brain-computer interfaces. A CMC-Cu(II) electrode attached to a volunteer's forehead, where it intimately associates with decision-making, attention, executive function, and other cognitive processes (**Fig. 4h**), enables detection of prefrontal cortex activity by measuring potential differences between the recording and reference electrodes.*

*When the subjects listen to music, brain activities elicited by real-time stimuli from musical elements—such as sound, lyrics, and melody—are converted into electrical signals and captured by the CMC-Cu(II) electrode (**Fig. 4i**). These acquired signals are subsequently transformed into time-frequency spectra via Fourier transformation (**Fig. 4j**). The resulting energy density distribution (represented by color intensity) across frequency bands offers a more detailed representation of the subjects' cognitive processes during each time interval. In contrast, EEG signals recorded by CMC-Cu(II) during the participants' mathematical calculation tasks (**Supplementary Fig. 25a**) and their corresponding time-frequency spectra (**Supplementary Fig. 25b**) exhibit distinct patterns of frequency band energy distribution compared to those observed during music listening. This difference confirms that the CMC-Cu(II) electrode effectively captures the brain's real-time neural responses to varying external stimuli, thereby reflecting the dynamic interplay between the brain and its environment. Additionally, the EEG signal collection and analysis system simultaneously provides concentration and relaxation metrics across different tasks, which also show significant variation. Music listening activity shows average coefficients of concentration and relaxation at 50.60 and 63.29, respectively (total range 0-100) (**Fig. 4k**), while mathematical calculation displays 57.60 for concentration and 44.87 for relaxation (**Supplementary Fig. 25c**), which aligned with the*

*general trend. A localized result over 20 seconds further illustrates the stability and precision of CMC-Cu(II) recordings (**Supplementary Fig. 26a and 25d**), with the time-frequency spectrum displaying well-defined frequency bands (**Supplementary Fig. 26b and 25e**)[35, 36]. These results demonstrate the accuracy and practical application potential of CMC-Cu(II) as an electrophysiological sensor."*

About EMG, Pages 22-23:

*"Further, we employ CMC-Cu(II) for multi-channel and finer-scale myoelectrophysiological monitoring. A pair of circular CMC-Cu(II) electrodes, corresponding to four lead channels, is fabricated in-situ in the extensor carpi radialis longus, extensor carpi ulnaris, biceps brachii, and triceps brachii muscles, respectively (**Fig. 5a**), with commercial electrodes as controls. During grip dynamometer tests, different muscle groups exhibit varying contractions during the same gripping action due to differences in force application, leading to distinct amplitudes and patterns of EMG signals. CMC-Cu (II) collects EMG signals that clearly distinguish muscle contractions across channels (**Fig. 5b**), similar to commercial electrodes (**Fig. 5c**), which demonstrates CMC-Cu(II)'s ability to precisely detect varying degrees of muscle activity. The SNR of CMC-Cu(II) signals (21.39 dB, 24.36 dB, 36.55 dB, and 18.63 dB) surpasses that of commercial electrodes (16.18 dB, 24.27 dB, 25.96 dB, and 10.72dB) at the corresponding site (**Fig. 5d**), demonstrating that CMC-Cu(II) exhibit exhibits superior performance in acquiring high-quality EMG signals compared to commercial electrode. Similar outcomes are observed during fist clenches (**Supplementary Fig. 27**)."*

*"For recognizing and transmitting electrophysiological signals during extensive motion, we further employ them for sensing microsignals. Two CMC-Cu(II) electrodes are positioned on the masseter muscle of both cheeks and throat (**Fig. 5g**), enabling to obtain mouth-related movements. Attractively, when phrases like 'Hi', 'Good morning', and 'Happy birthday' are spoken, the CMC-Cu(II) electrodes record high-fidelity EMG signals matching both the durations and intensity of muscle contractions."*

12. How is the gel applied to the skin? What is the adhesion force? Is there a risk of slippage? Will the gel dry over time? How practical is this as a device?

Author reply:

Q12-1. The CMC-Cu(II) membrane is formed via a two-step dipping strategy directly on the target substrate (e.g., skin). Specifically, the substrate is first immersed in an aqueous solution of carboxymethyl cellulose (CMC), allowing the polymer to adsorb onto the surface. It is then transferred into a Cu(II) ion solution, where rapid coordination between CMC and Cu(II) triggers in situ membrane formation. This process is rapid, mild, and compatible with biological surfaces.

If patterned structures are desired, a laser-cut mask with predefined geometries can be placed onto the substrate prior to the dipping steps. The mask selectively exposes regions of the substrate during CMC deposition, confining the polymer adsorption to desired areas. After removal of the mask, the Cu(II) solution is applied to initiate site-specific coordination, resulting in precise and mask-defined membrane formation (**Figure R15, Figure R15a** is

included as **Supplementary Fig. 6 in Supplementary Information**). This approach enables direct patterning of conductive hydrogel membranes on skin with excellent spatial fidelity and without the need for photolithography or complex transfer processes, as demonstrated in **Figure R15b (Supplementary Fig. 7)**.

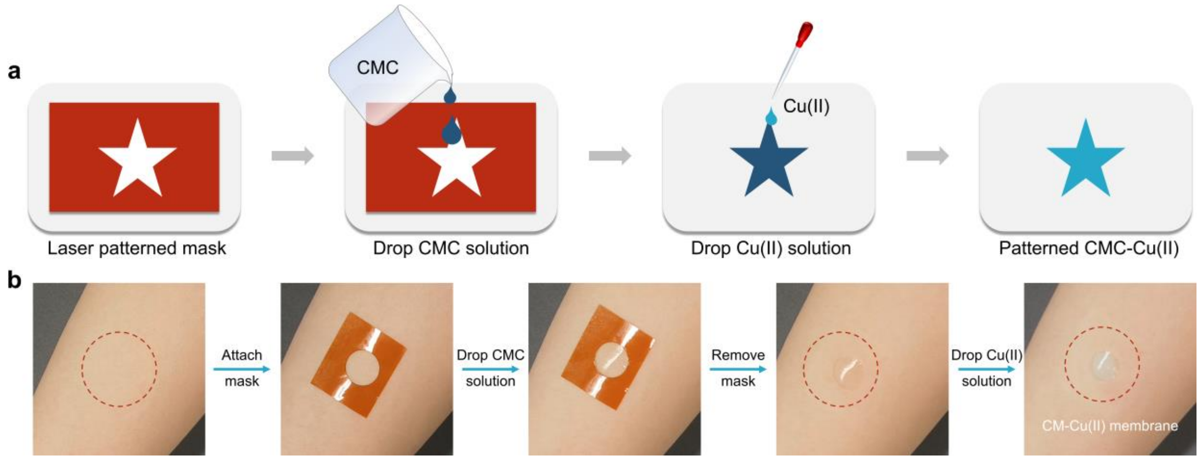

Figure R15. a Schematic representation of the fabrication process for patterned CMC-Cu(II) via mask-forming approach. **b** The formation of the CMC-Cu(II) membrane on the skin. **c** The test configuration of the CMC-Cu(II) membrane on the skin.

Updates to the revised manuscript: According to Reviewer #1's comments, we have added the above information in the revised manuscript on Page 7.

*"Additionally, the preparation of CMC-Cu(II) membrane with defined size and shape on the skin via the mask-forming method is illustrated in **Supplementary Fig.** 7."*

Q12-2 and -3. In response to the reviewer's inquiry concerning adhesion, we performed a 90° peeling test on the CMC-Cu(II) membrane using pigskin with a modulus comparable to that of human tissue as the substrate. The average adhesion strength was measured at 6.05 N/m (**Supplementary Fig. 8**). While moderate in magnitude, this adhesion strength is sufficient to maintain stable contact during routine skin movements, ensuring reliable performance when used as a skin-attached electronic device without causing discomfort or damage to the skin. Based on both the measured adhesion strength and visual observation, the CMC-Cu(II) membrane exhibits no tendency to slip during operation. This balanced adhesion aligns well with the requirements of epidermal electronics, which favor conformal, soft interfaces over rigid fixation. These characteristics, in combination with the membrane's conformability and ultrathinness, highlight its suitability for applications in electrophysiological signal monitoring. To provide a more comprehensive characterization of CMC-Cu(II), the adhesion strength data (**Figure R14c**) have been added to **Supplementary Fig. 8** in the supplementary materials, along with corresponding descriptions and test method incorporated into the revised manuscript.

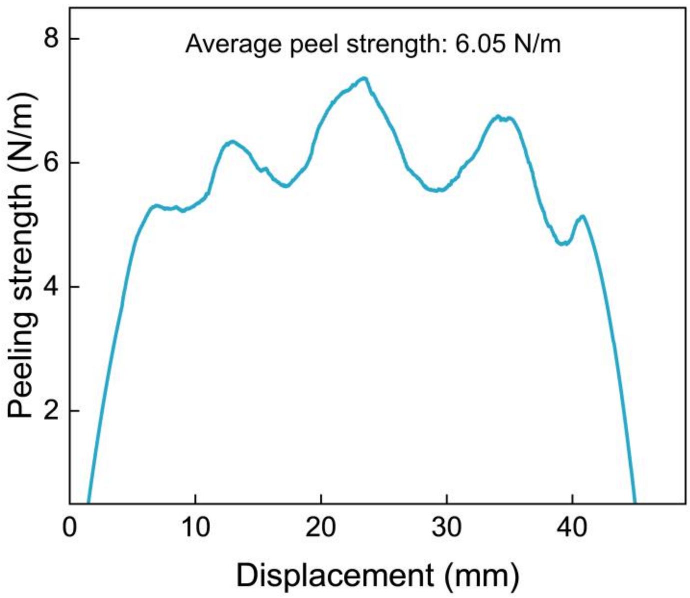

Supplementary Fig. 8. Peeling strength of the CMC-Cu(II) membrane detached from pigskin by 90° peeling test.

Updates to the revised manuscript: According to Reviewer #1's comments, we have added the above results and corresponding test method in the revised manuscript on Pages 7 and 27.

> *"Furthermore, a 90° peeling test revealed an average adhesion strength of 6.05 N/m for the CMC-Cu(II) membrane (**Supplementary Fig. 8**), which, together with the membrane's conformability and ultrathinness, ensures stable skin contact and signal reliability during electrophysiological monitoring."*

> *"Characterization*
> *The thickness of CMC-Cu(II) membranes was characterized using Hitachi SU-1510 SEM to obtain cross-sectional characterization data. To assess the conformal properties, a CMC-Cu(II) membrane was attached onto a wavy PVC mold and subjected to embrittle in liquid nitrogen. 90° peeling test and stress-strain curves were measured on the electronic universal testing machine (Instron Corporation -3369)."*

Q12-4. Water retention is indeed crucial for maintaining the performance of the CMC-Cu(II) gel membrane. This issue is addressed in detail in Reviewer #1, Comment 5 (see Page 21 of this response letter). Specifically, we compared water loss in ambient (open) and testing (sealed) environments, showing that the testing configuration significantly delayed dehydration, allowing sufficient hydration to be maintained for over 12 hours. To further enhance water retention, we incorporated ethylene glycol (EG) as a high-boiling-point humectant, which effectively improved hydration stability. In addition, the stability of electrophysiological signal acquisition was systematically evaluated using the encapsulated configuration, and reliable operation was confirmed. These findings, along with the relevant experimental configuration and performance validation, are presented in **Figures R9–R12** and discussed accordingly in the revised manuscript and supplementary materials.

Q12-5. Regarding the practicality of CMC-Cu(II) as a device, we have conducted a systematic evaluation and provided supplementary explanations from the perspective of cost, operational convenience, performance, and biocompatibility. We believe the CMC-Cu(II) membrane shows strong potential for real-world applications:

- **Cost and sustainability**: The membrane is fabricated from carboxymethyl cellulose (CMC), a low-cost, abundant, and biodegradable biopolymer. The entire device is assembled using minute amounts of Cu(II) solution without the need for specialized facilities or expensive equipment, highlighting its suitability for scalable and eco-friendly manufacturing.

- **Operational convenience**: The "dipping–dipping" strategy enables in-situ formation of the membrane within 2 seconds on diverse target surfaces, with no need for post-processing, transfer steps, or adhesive layers. This simple and rapid operation greatly improves usability in both clinical and consumer contexts.

- **Comparable performance**: The CMC-Cu(II) membrane exhibits excellent conformability (thickness ~3.4 μm), stable adhesion (6.05 N/m), and significantly reduced interfacial impedance—84% lower than that of commercial electrodes at 20 Hz. These features contribute to high-fidelity acquisition of physiological signals (ECG, EOG, EMG, EEG) across multiple anatomical sites and users, with consistent and reproducible performance (**Fig. 4, Fig. 5; Supplementary Figs. 24–27**).

- **Biocompatibility**: The cellulose-based membrane is inherently biocompatible and biodegradable. In vitro cell viability assays confirmed that the CMC-Cu(II) membrane maintains 100% viability after 24 hours (**Supplementary Fig. 21**), affirming its suitability for prolonged skin contact and biological interfacing.

We also acknowledge potential limitations and ongoing development directions:

- **Performance improvement**: Despite the advantages above, certain performance aspects, including environmental stability and conductivity, can be further optimized. Moderate water loss under ambient conditions is inevitable, but as detailed in Comment 5 (**Figures R9–R12**), testing in a sealed environment and incorporation of ethylene glycol (EG) significantly prolong hydration and signal stability, enabling applications such as Holter monitoring. Furthermore, although the conformality and ion-rich membrane ensure low impedance, introducing electron-conductive components could enable hybrid ionic–electronic conduction, enhancing signal fidelity and supporting advanced diagnostic functions.
- **Device integration**: Although this study focused on the membrane-level interface, we are actively exploring integration with soft circuit modules and wireless transmission systems to enable fully functional wearable healthcare devices in the future.

Therefore, we conclude that the CMC-Cu(II) device demonstrates high practicality and

significant application potential, making it a promising candidate for future deployment in clinical diagnostics or daily health monitoring settings.

13. Can the CMC-Cu(II) electrode sustain measurements over extended periods? Physiological monitoring, such as EEG and EMG, typically requires at least 60 minutes. How stable is the material during operation over an hour?

Author reply: We appreciate the reviewer's concern regarding the long-term stability of the CMC-Cu(II) electrode during physiological monitoring. To address this, we conducted systematic evaluations of dehydration behavior, electrical properties, and functional signal monitoring over time. Our results confirm that the membrane retains sufficient hydration under testing conditions and demonstrates reliable performance within and beyond 1 hour. Furthermore, this reliability can be further enhanced by incorporating ethylene glycol (EG) to reduce dehydration.

1. Stability in Aqueous Conditions

The water-based CMC-Cu(II) membrane maintains adequate hydration for over 12 hours in the sealed test configuration (**Figure R9c**), enabling extended monitoring durations. Impedance measurements at 20 Hz over 24 hours (**Figure R16a and b**) show only a slight increase in the first 6 hours, from 9.75×10^4 Ω to 9.99×10^4 Ω, with values gradually rising afterward. This indicates that the electrical properties remain stable within the initial 1-hour window, supporting its application for short- to mid-term monitoring tasks.

Physiological signal performance was evaluated through ECG and EMG tests at 0 h and after 3 h. As shown in **Figure R16c and d**, signal amplitude showed minor attenuation but maintained functional integrity. Even after 3 hours, muscle-specific EMG signals from four muscle groups (brachioradialis, flexor carpi radialis, biceps brachii, triceps brachii) remained distinguishable during dynamic arm movements. These results confirm functional stability within 1 hour of operation.

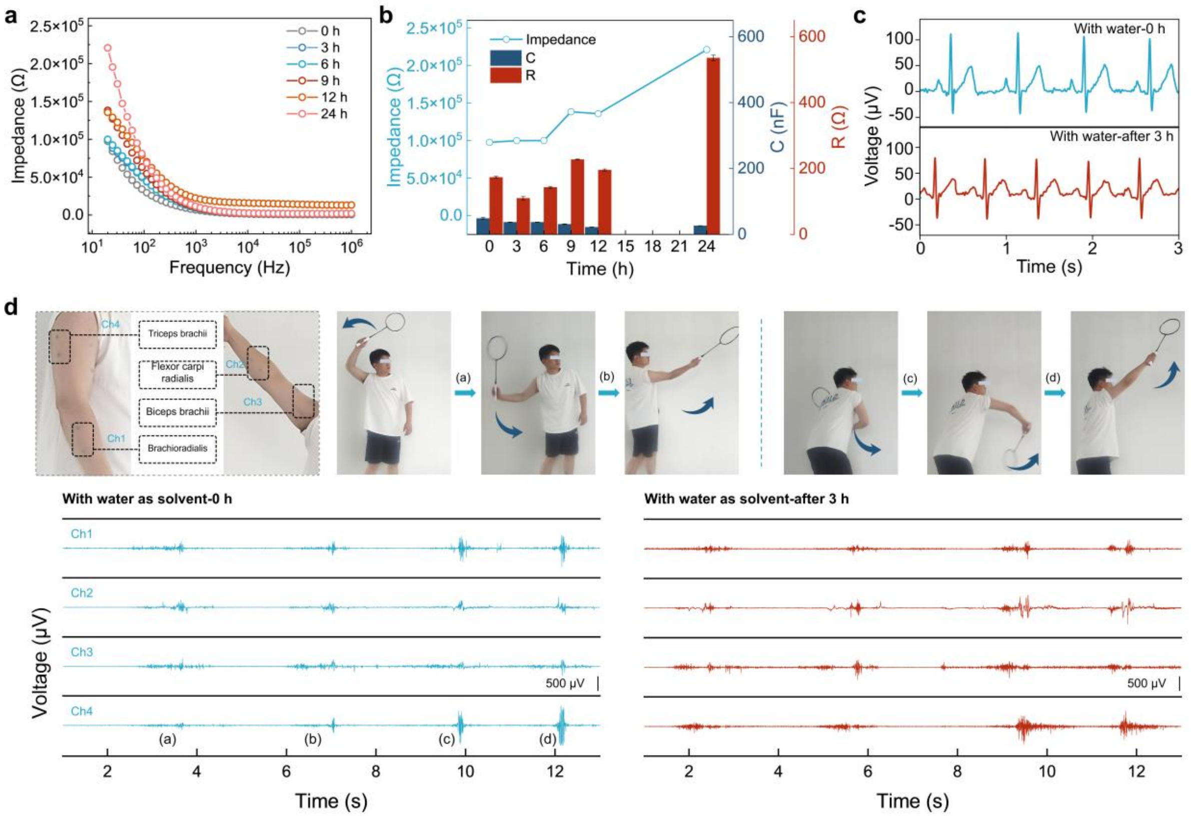

Figure R16. The stability of the electrical and physiological signal monitoring capability for the CMC-Cu(II) membrane over time. a Skin-contact impedance values within 20 Hz to 1000 kHz of CMC-Cu(II) electrode varies with time. **b** Skin-contact impedance value at 20 Hz, capacitance, and resistance of CMC-Cu(II) electrodes varies with time. **c** The periodic ECG signals of CMC-Cu(II) acquired at 0 h and after 3 h. **d** Schematic illustration of electrode placement on different arm muscles during EMG collection and a series of arm movements of badminton-playing during the acquisition of arm EMG signals (top); EMG signals corresponding to the series of arm movements of the subject captured by the CMC-Cu(II) at 0 h and after 3 h (bottom).

2. Stability with Ethylene Glycol (EG) as Solvent

To further enhance water retention and stability, ethylene glycol (EG) was used to prepare the CMC-Cu(II) membrane. As illustrated in **Figure R17**, both electrical impedance and electrophysiological signal outputs (ECG, EMG) remained highly stable across the full 24-hour period. Compared to the water-based membrane, signal noise was significantly reduced and long-term signal fidelity improved. Therefore, the EG-based CMC-Cu(II) membrane is especially promising for applications requiring extended monitoring, such as Holter testing.

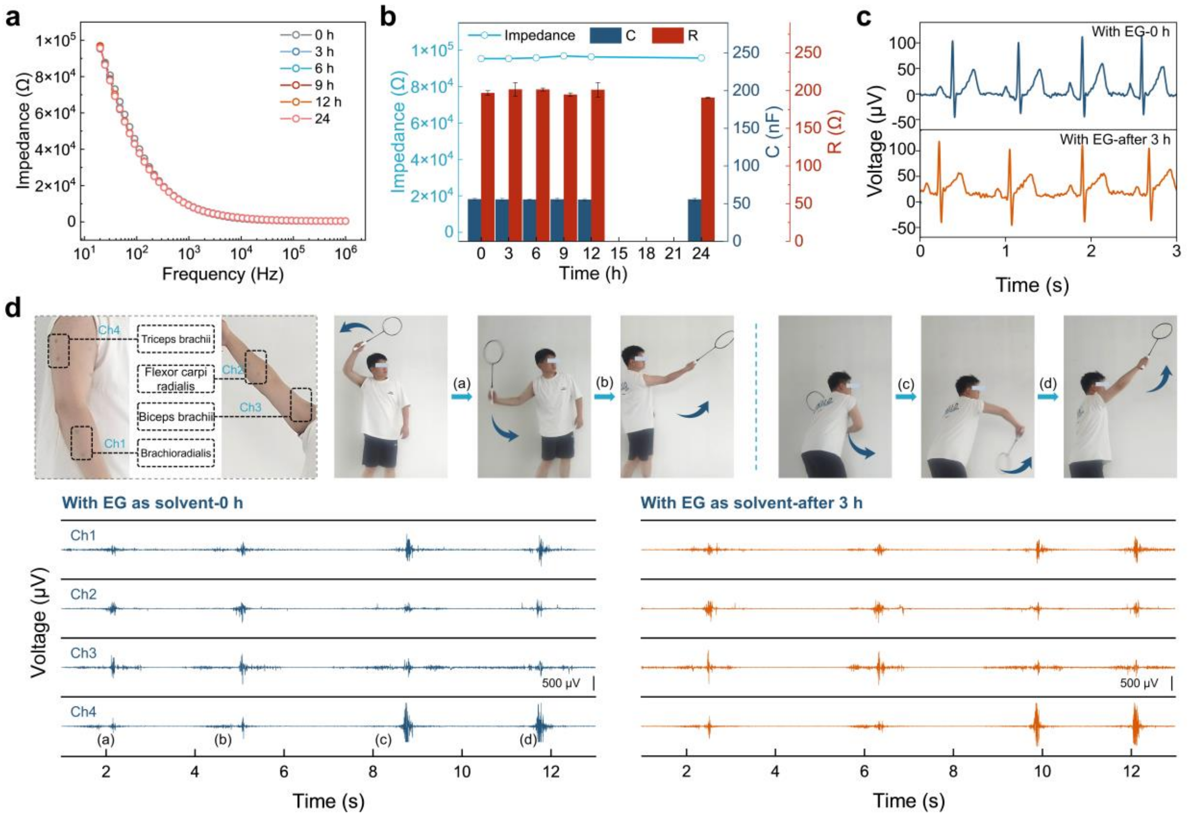

Figure R17. The stability of the electrical and physiological signal monitoring capability for the CMC-Cu(II) membrane with EG as solvent over time. a Skin-contact impedance values within 20 Hz to 1000 kHz of CMC-Cu(II) electrode with EG as solvent vary with time. **b** Skin-contact impedance value at 20 Hz, capacitance, and resistance of CMC-Cu(II) electrodes with EG as solvent vary with time. **c** The periodic ECG signals of CMC-Cu(II) acquired at 0 h and after 3 h. **d** Schematic illustration of electrode placement on different arm muscles during EMG collection and a series of arm movements of badminton-playing during the acquisition of arm EMG signals (top); EMG signals corresponding to the series of arm movements of the subject captured by the CMC-Cu(II) with EG as solvent at 0 h and after 3 h (bottom).

14. Was the comparison of impedance in Figure 4b fair?

Author reply: We sincerely thank the reviewer for raising this important question regarding the fairness of the impedance comparison shown in **Fig. 4b**. We would like to clarify the experimental design to ensure the comparison is indeed fair and reliable.

First, all measurements were performed under identical experimental conditions, including the same skin site on the same subject, the same environmental conditions (temperature and humidity), and the same measurement setup (impedance analyzer, frequency sweep range of 20 Hz–1000 kHz, and identical instrument parameters). These controls were applied to minimize any external factors that might affect the impedance results.

Second, the electrode-skin contact area was carefully kept consistent between the CMC-Cu(II)

electrodes and the commercial electrodes. This ensures that the impedance differences observed are not due to mismatches in electrode size or placement but rather reflect the intrinsic properties of the electrodes themselves.

Third, the chosen frequency range of 20 Hz–1000 kHz is widely used in electrode characterization and covers the physiologically relevant bands for bioelectrical signal acquisition. Thus, the impedance comparison across this range provides meaningful insight into the practical performance of both electrodes in real applications.

Taken together, we believe that the impedance comparison presented in **Fig. 4b** was conducted under fair and controlled conditions, and the results reliably reflect the differences in skin–electrode contact performance between the CMC-Cu(II) electrodes and the commercial electrodes.

Reviewer #2 (Remarks to the Author):

1. How does the performance of the CMC-Cu(II) bio-skin quantitatively compare to state-of-the-art electronic skins in terms of conductivity, biocompatibility, and fabrication scalability? A comparison with benchmarks would highlight the novelty and advantages of the proposed method.

Author reply: We thank the reviewer for this insightful comment. In response, we address this point in two parts: (i) clarifying the novelty of our work, and (ii) providing a quantitative comparison of the CMC–Cu(II) bio-skin with state-of-the-art electronic skins in terms of conductivity, biocompatibility, and fabrication scalability.

1. Clarifying the Novelty of Our Work

To summarize, our CMC-Cu(II) approach constitutes a unique membrane-forming strategy, and the novelty of our work includes (**Figure 18**): (i) developing an ultra-fast (≈2 s), highly convenient in-situ membrane formation strategy, whose mechanism has been systematically investigated and has guided us to expand the method to other systems; (ii) the achieved membrane exhibits various advantageous features, including ultrathin thickness (~3.4 μm), conformality, patternability, a transfer-free process (no additional post-transfer step), conductivity, low impedance, biocompatibility, and biodegradability, which fit perfectly for epidermal electrodes for physiological monitoring. Detailed explanations are as follows.

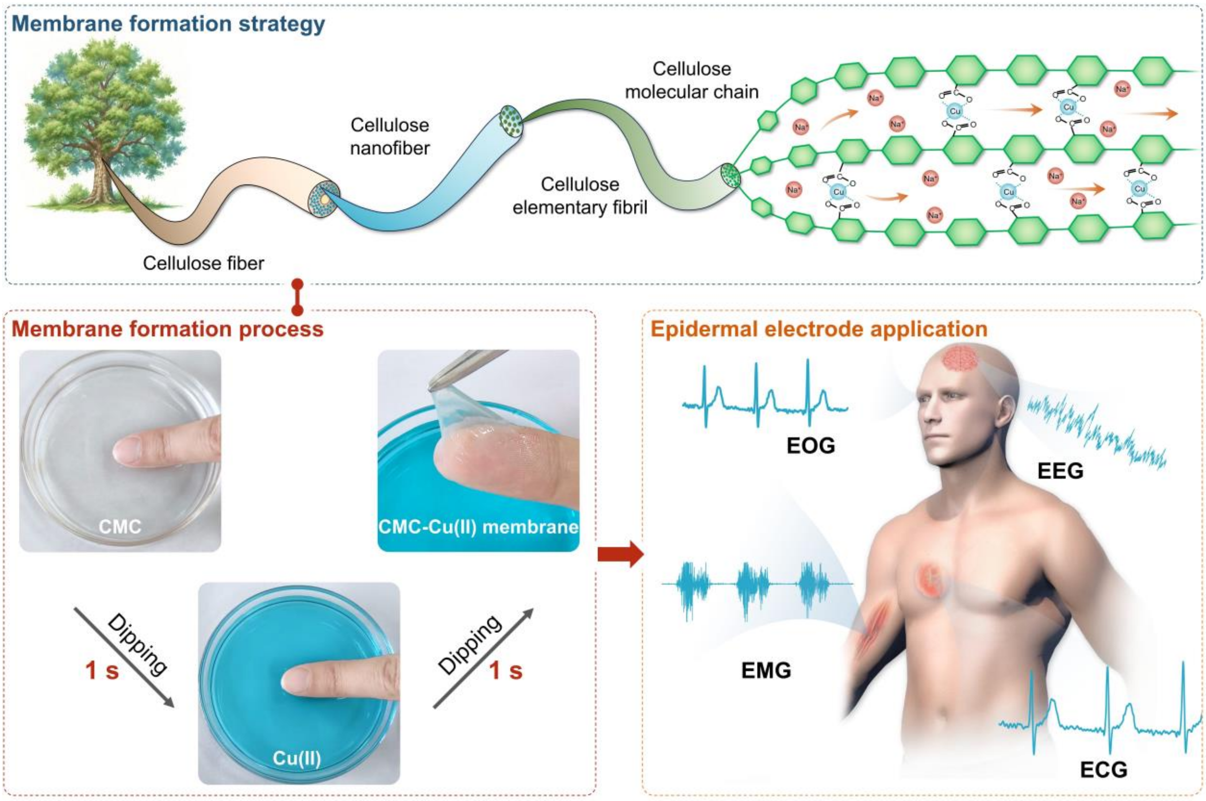

Figure R18. The unique membrane-forming strategy constituted by the CMC–Cu(II) approach for epidermal electrode.

- **Unique membrane formation technology.** The key novelty of this study is a facile "dipping–dipping" molecular-assembly method using the CMC–Cu(II) system that enables an ultra-fast liquid-to-solid transformation. Unlike traditional routes of casting → drying/curing → transfer, which are complicated, our approach directly forms the bio-membrane in situ by sequentially dipping the target surface into CMC solution and then Cu(II), which is highly convenient and time-saving. The rapid solidification is driven by strong coordination and accelerated interfacial assembly, elucidated by experiments (UV–vis, XANES, EXAFS) and simulations (MDS—captures nanosecond interfacial assembly kinetics; DFT—quantifies coordination structures and binding energies consistent with XANES/EXAFS). We further generalize the strategy to other metal ions (Ca(II), Fe(II), Ag(I)) and other biomacromolecules (MC, CMCH), establishing design rules that relate coordination energetics and interfacial assembly dynamics to membrane-forming efficacy, thereby delivering a transfer-free, substrate-agnostic, seconds-scale route to ultrathin, conformal membranes with tunable performance for bio-integrated electronics (**Figure R3**).

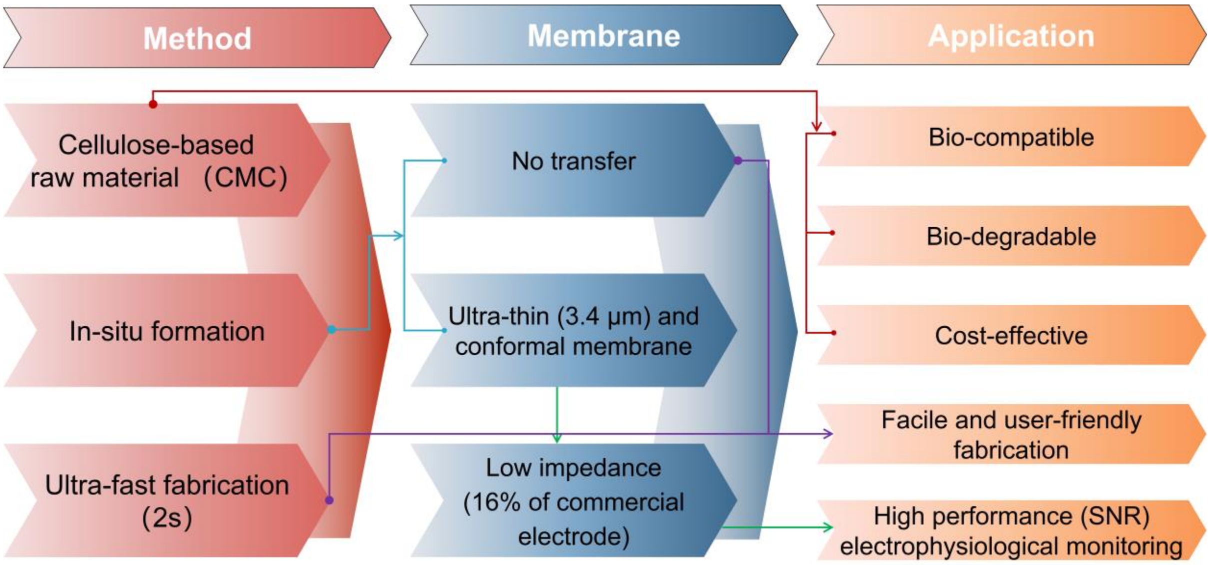

Figure R3. Illustration of the CMC-Cu(II) advantages for epidermal electronics from method to membrane and epidermal electrode applications.

- **Multiple advantageous features that perfectly suit epidermal electrodes.** The method—and the resulting membrane—offer a suite of advantages that meet the core requirements of epidermal electrodes (**Figure R3 and R4**). From the materials perspective, the membrane is ultrathin and conformal, enabling low interfacial impedance and a high signal-to-noise ratio for high-performance bioelectrical measurements—its most critical attribute. In addition, being derived from a cellulose derivative, the membrane is biocompatible and biodegradable, making it cost-effective, skin-benign, and environmentally friendly. From the manufacturing perspective, the process is ultra-fast and convenient; the in-situ formation strategy eliminates transfer steps that could damage fragile membranes, and its inherent patternability broadens application scenarios. Collectively, these features make the membrane an excellent fit for high-fidelity acquisition of physiological signals (ECG, EOG, EMG, EEG).

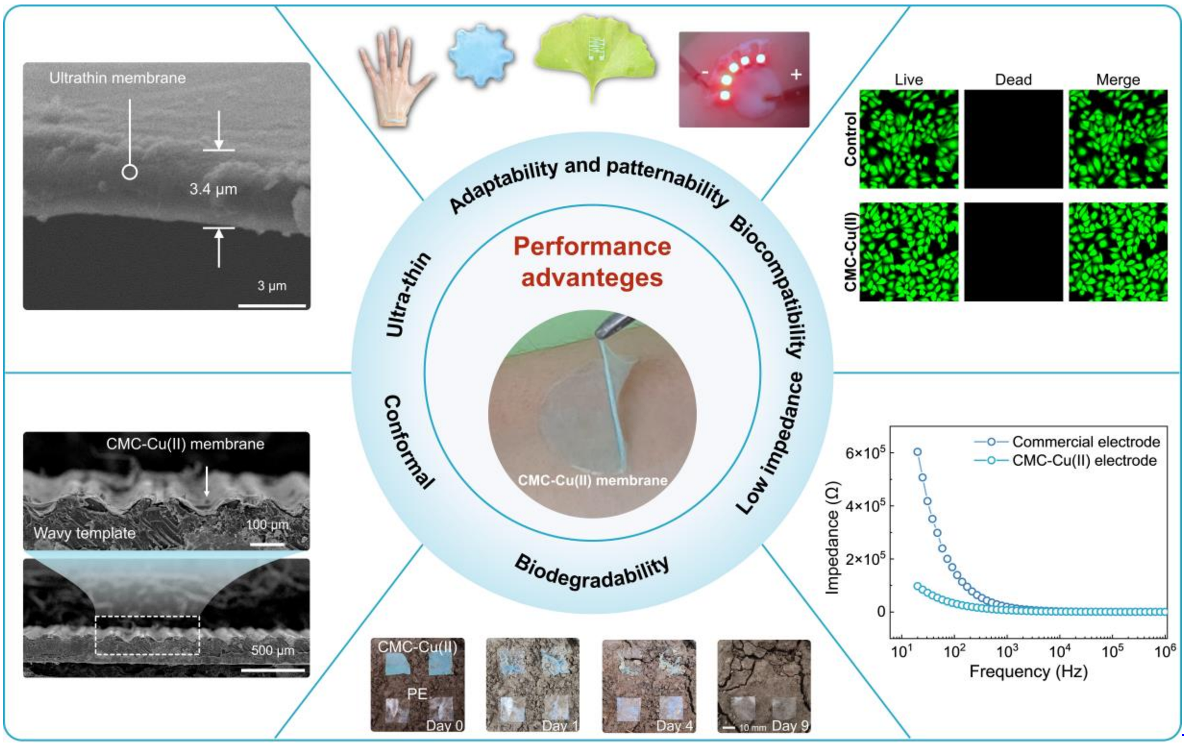

Figure R4. The combination of attributes about functional performance of CMC-Cu(II) membrane involving ultrathin, conformal, adaptive, patternable, degradable, biocompatible and low impedance characteristics.

In conclusion, this work presents a novel nature-inspired "dipping-dipping" assembly method and systematically validates its scientific foundation and practical significance through systemic evaluations of performance, underlying mechanisms, and application potential. Furthermore, the approach demonstrates broad universality and scalability. We believe this work possesses advancement in the field of "controlled preparation methods for flexible electronic materials," offering clear innovation and translational potential.

2. The quantitative comparison between the CMC-Cu(II) biological membrane and the current state-of-the-art electronic skins

(i) Conductivity: For epidermal electrodes, impedance is the key electrical parameter indicating their ability to capture high-quality bioelectric signals from the skin. Low electrode-skin impedance reflects better signal detection and conduction. The electrode-skin interface is typically modeled as a parallel LCR circuit of leakage resistance and capacitance (**Figure R19a**), illustrating the impedance measurement setup in dual-electrode configurations. Impedance for each recording electrode can be calculated using the following equation:

$$Z(\omega) = R_s + \frac{R_d/jC_d\omega}{R_d+1/jC_d\omega} = R_s + \frac{R_d}{1+jC_dR_d\omega}, \quad \omega=2\pi f \qquad (1)$$

Where the resistor R_d represents the charge transfer impedance of the electrode-skin interface, while the capacitance C_d stands for the electric double layer between the electrode and skin. R_s

is related to the skin surface conditions and underlying tissues, and R_{tissue} is the resistance of the dermis, subcutaneous tissue layers, and deeper tissues. Thus, maintaining high interfacial capacitance and low resistance at the electrode-skin interface (Eq.1) is crucial for minimizing bioelectrical signal attenuation and enhancing the overall signal-to-noise ratio (SNR)[9].

Our "dipping-dipping" molecular assembly strategy allows for rapid in-situ membrane formation on biological surfaces of arbitrary geometry within 2 seconds, with the CMC-Cu(II) membrane exhibiting excellent conformability (thickness ~3.4 μm) (**Figure R19b and c**), stable adhesion (6.05 N/m) (**Figure R19d**), which significantly reduces interfacial impedance—84% lower than that of commercial electrodes at 20 Hz (**Figure R19e and f**). Furthermore, as shown in **Table R1**, the impedance of our membrane is comparable or superior to previously reported hydrogel- or polymer-based epidermal electrodes, thereby enabling high-fidelity recordings of ECG, EEG, EOG, and EEG signals.

While the electrical conductivity of the CMC-Cu(II) membrane is not designed to reach the highest reported levels, the novelty of our work lies in simultaneously achieving multiple critical requirements for epidermal electronics—low impedance, good biocompatibility, conformal adhesion, environmental degradability, and rapid, scalable fabrication—through a simple and cost-effective strategy. These aspects are often difficult to achieve concurrently in existing electronic skin platforms.

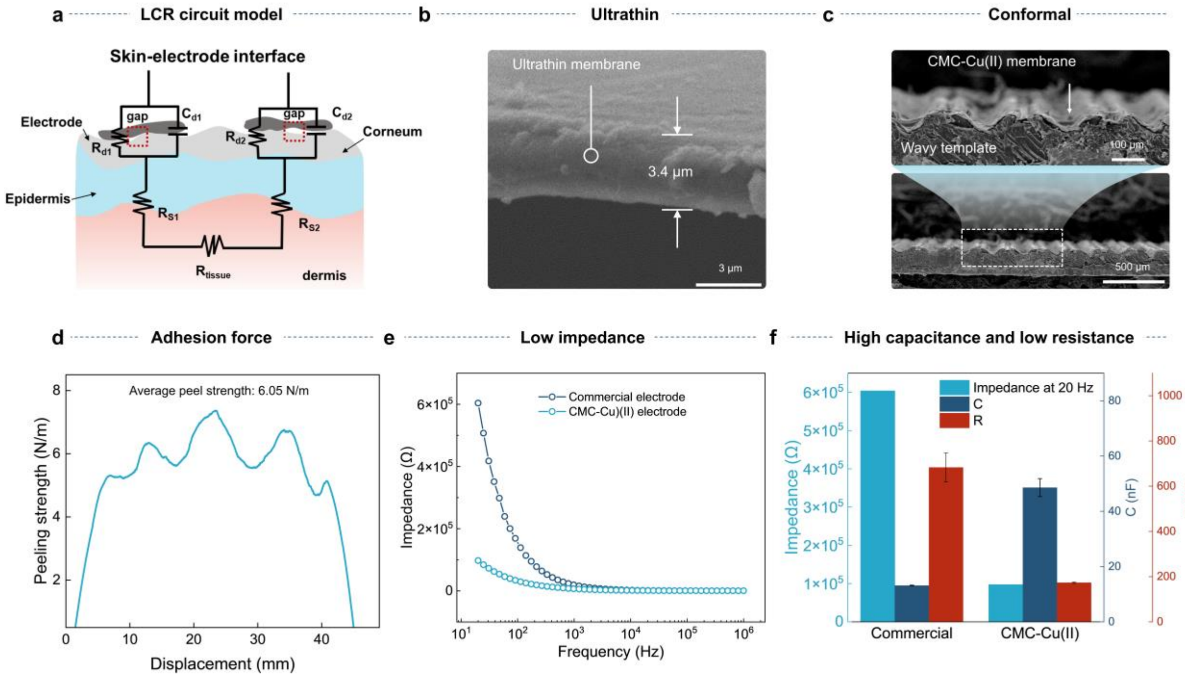

Figure R19. The low impedance of CMC-Cu(II) membranes, along with their associated contribution performances. a The simplified LCR circuit model of the electrode-skin interface. **b** Ultrathin feature of CMC-Cu(II) membrane shown by cross-sectional SEM image. **c** Highly conformal behavior of CMC-Cu(II) membrane attached on PVC wavy mold displayed by cross-sectional SEM images. **d** Peeling strength of the CMC-Cu(II) membrane detached from pigskin by 90° peeling test. **e** Skin-contact impedance values of CMC-Cu(II) and

commercial electrodes within 20 Hz to 1000 kHz. **f** Skin-contact impedance values at 20 Hz, capacitance, and resistance of CMC-Cu(II) and commercial electrodes.

Figure R19d and **Figure R19f** have been added as **Supplementary Fig. 8** and **Supplementary Fig. 22** in the revised Supplementary material, respectively.

(ii) Biocompatibility: Petroleum-derived epidermal electronics often suffer from limited biocompatibility and potential long-term irritation. In contrast to most previously reported epidermal electrodes, which are predominantly composed of non-renewable synthetic polymers (such as polyurethane and poly(ethylene-glycol) diacrylate), the proposed CMC-Cu(II) membrane is inherently biocompatible and biodegradable. In vitro cell viability assays confirmed that the CMC-Cu(II) membrane maintains 100% viability after 24 hours (**Supplementary Fig. 21**), affirming its suitability for prolonged skin contact and biological interfacing. For detailed data figures, please refer to the following Question 2.

(iii) Fabrication scalability: The manufacturing scalability of CMC-Cu(II) can be demonstrated through its cost-effectiveness and sustainability, the operational convenience, and its diverse application scenarios.

- **Cost-effectiveness and sustainability**. The membrane is fabricated from carboxymethyl cellulose (CMC), a low-cost (18.27 $\$\cdot kg^{-1}$), abundant, and biodegradable biopolymer. The entire device is assembled using minute amounts of Cu(II) solution without the need for specialized facilities or expensive equipment, highlighting its suitability for scalable and eco-friendly manufacturing.
- **Operational convenience**. The "dipping–dipping" strategy enables in-situ formation of the membrane within 2 seconds on target surfaces without requiring post-processing, transfer steps, or adhesive layer (**Figure R20a**), thereby offering significant advantages in terms of operational convenience and time efficiency compared to conventional surface electrode fabrication methods (**Table R1)**. This straightforward and rapid process presents no operational barriers, even for non-specialists, and supports large-scale production, thereby significantly enhancing its practical applicability in both clinical and consumer domains.
- **Diverse application scenarios.** Owing to the features of this method, including ultra-fast in-situ formation, no requirement for post-processing, no need for transfer, and no demand of additional adhesion layer, the resulting CMC-Cu(II) membrane can be effectively applied to a variety of target surfaces (**Figure R20b**). Moreover, arbitrary patterned structures can be fabricated using only a single mask. These advantages render the method highly competitive and broadly applicable across diverse complex scenarios.

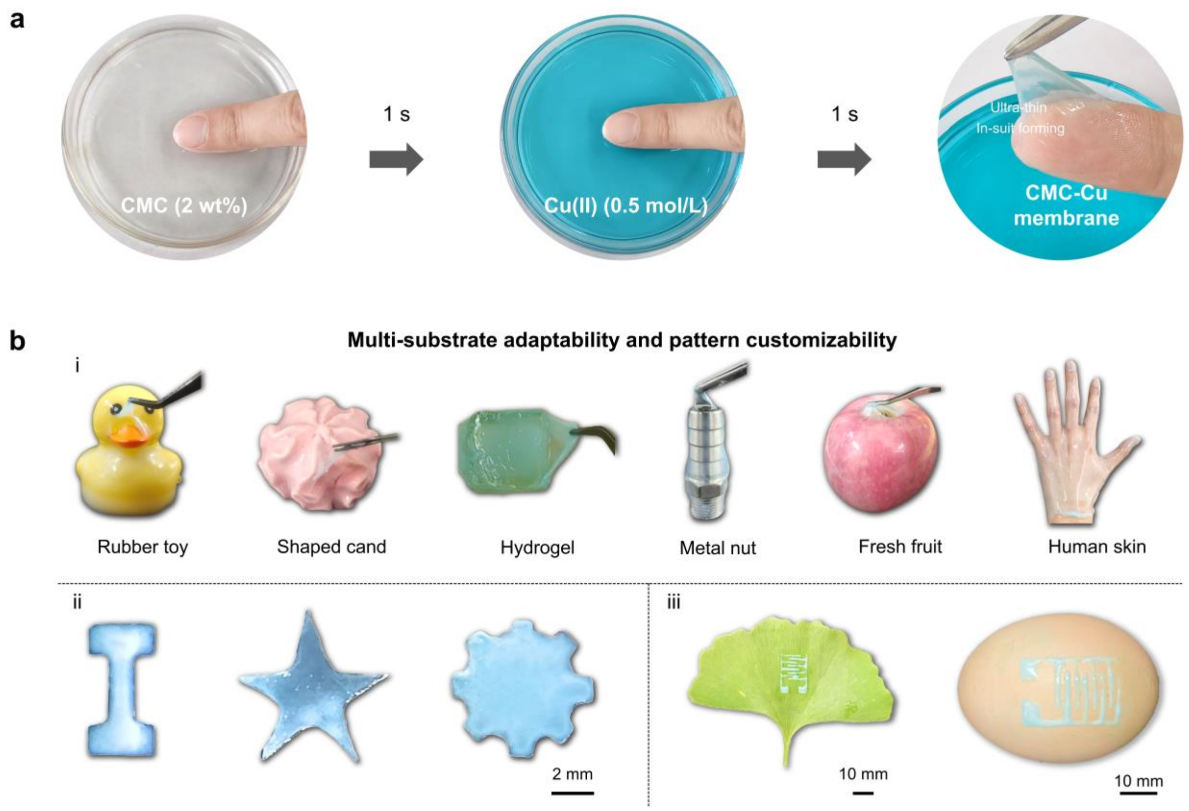

Figure R20. The operational convenience and diverse application scenarios of CMC-Cu(II). a Digital photographs of CMC-Cu(II) membranes fabricated by the "dipping-dipping" coordination assembly method from CMC and Cu(II) solutions. **b** The adaptable, highly conformal CMC-Cu(II) membrane assembled in-situ on irregular surfaces of multi-substrates (i). Digital photographs of CMC-Cu(II) patterns (ii) on diverse 2D and 3D surfaces (iii).

Together, these comparisons underscore that the CMC-Cu(II) bio-skin integrates low impedance, inherent biocompatibility from biobased materials, and unprecedented fabrication efficiency, thereby establishing clear advantages over current state-of-the-art epidermal electronic systems.

Table R1. Comparison of the CMC-Cu(II) membrane and other epidermal electrodes in conductivity, biocompatibility, and fabrication scalability.

**Comparison indicators**	**Ref.7**	**Ref.8**	**Ref.9**	**Ref.10**	**Our work**
**Impedance (kΩ) (Order of magnitudes at 20 Hz)**	10^2	10^2	10^2	10^3	**10^2**
**Thickness (μm)**	10	22	12.6	3.55	**3.4**
**Biocompatibility**	Primarily petroleum-based	Primarily petroleum-based	Primarily petroleum-based	Primarily petroleum-based	**Bio-based materials**

	materials	materials	materials	materials	
**Required fabrication time**	2 hours	3.5 hours	1 hours	10 minutes	**2 seconds**
**Fabrication methods**	Electrospinning, one-pot	Blending, drop-casting, drying	UV photo-polymerization	UV photo-polymerization	**Simple dipping-dipping**

References:
7. Zhang Z*, et al.* A 10-micrometer-thick nanomesh-reinforced gas-permeable hydrogel skin sensor for long-term electrophysiological monitoring. *Sci. Adv.* **10**, eadj5389.
8. Zhang L*, et al.* Fully organic compliant dry electrodes self-adhesive to skin for long-term motion-robust epidermal biopotential monitoring. *Nat. Commun.* **11**, 4683 (2020).
9. Wang B*, et al.* Antifreezing Ultrathin Bioionic Gel-Based Wearable System for Artificial Intelligence-Assisted Arrhythmia Diagnosis in Hypothermia. *ACS Nano* **19**, 8176-8188 (2025).
10. Zhang L*, et al.* A 3.55-μm Ultrathin, Skin-Like Mechanoresponsive, Compliant, and Seamless Ionic Conductive Electrode for Epidermal Electrophysiological Signal Acquisition and Human-Machine Interaction. *Exploration*, 20240232 (2025), DOI: https://doi.org/10.1002/EXP.20240232.

2. Given the use of Cu(II) ions, what is the environmental fate of the degraded CMC-Cu(II) membrane, particularly regarding potential Cu(II) ion release into soil or water? Additionally, were authors able to conduct cytotoxicity or long-term skin contact studies to ensure biocompatibility, especially considering the potential toxicity of copper ions?

Author reply: Thank you very much for raising these two significant questions concerning the practical application value and safety of our work. The Cu(II) content data from the CMC-Cu(II) membrane, together with the results of vitro cytotoxicity test, demonstrate that the material poses no risk to the environment or human skin. Detailed data and analyses are provided as follows.

(i) Regarding the environmental fate of the CMC-Cu(II) membrane and the potential release of copper ions.

- **The biodegradability of the material matrix:** The primary component of our membrane is CMC, a natural polysaccharide derived from cellulose that exhibits excellent biodegradability. In natural environments, CMC can be efficiently degraded by microorganisms into carbon dioxide and water, thereby avoiding persistent plastic pollution.

- **The behavior and environmental fate of Cu(II) ions:** In the CMC-Cu(II) membrane,

Cu(II) ions are strongly coordinated with the carboxyl groups of CMC, significantly limiting their leaching during degradation. Our experimental results show that when the CMC-Cu(II) membrane was placed in an aqueous environment, it only exhibited area contraction over a 30-day period, with no detectable free Cu(II) ions released into the surrounding water. Specifically, digital images and normalized membrane area measurements revealed a gradual contraction of the membrane, with the membrane area decreasing to 45.19% of its initial value after 30 days (**Figure R21a**). Since the structural formation of the CMC-Cu(II) membrane relies on the strong coordination interaction between CMC and Cu(II), to further verify its structural integrity. The UV spectroscopy was employed to analyze both aqueous Cu(II) solutions and the soaking water collected after immersing the CMC-Cu(II) membrane. The results reveal a distinct absorption peak at 194 nm in the UV spectrum of Cu(II), whereas no such absorption peak is observed in the soaking water even after 30 days of immersion (**Figure R21b**).

During soil degradation, any trace amounts of Cu(II) released are naturally present in the environment and, at low concentrations, serve as essential micronutrients for plants and microorganisms—copper is commonly included in agricultural fertilizers. The total Cu(II) content in our membrane is minimal (approximately 0.244 mg per membrane, according to the results of Inductively Coupled Plasma-Atomic Emission Spectrometry (ICP-AES)). From the standpoint of "potential toxicity" to "controlled functionality," the gradual release of trace copper ions from the degrading CMC-Cu(II) membrane may even yield beneficial effects in agricultural or environmental applications, such as serving as a micronutrient supplement for soils or acting as a mild antifungal agent.

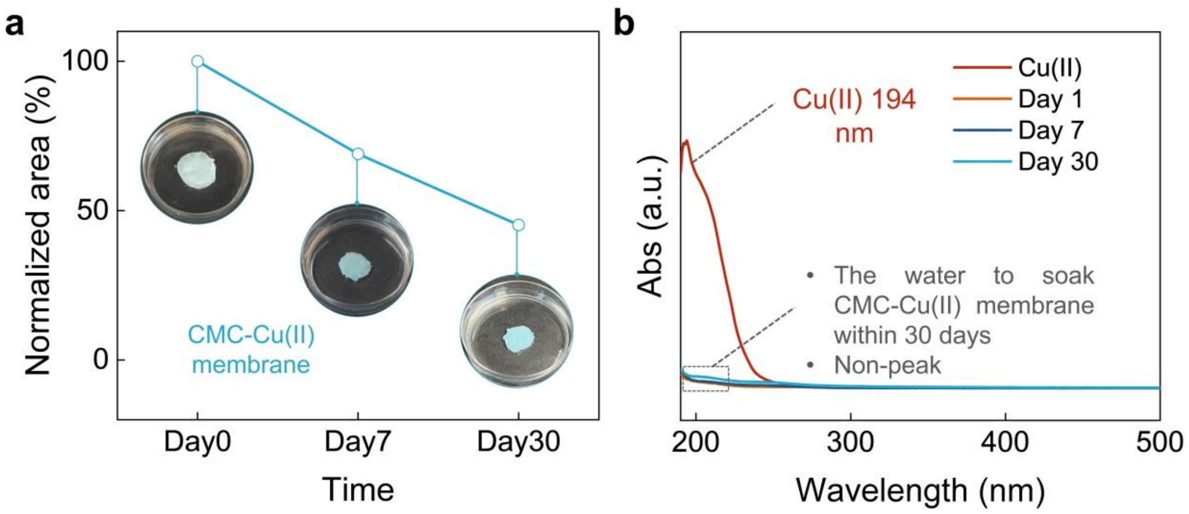

Figure R21. The influences of Cu(II) release and the biocompatibility evaluation of CMC-Cu(II) membrane. a The apparent morphology and normalized membrane area of CMC-Cu(II) membrane in aqueous environment within 30 days. **b** UV absorption spectra of Cu(II) and soaking water collected after immersing the CMC-Cu(II) membrane at day 1, day 7 and day 30.

(ii) Regarding the biocompatibility and cytotoxicity of the CMC-Cu(II) membrane.

In vitro cytotoxicity test showing biocompatibility of CMC-Cu(II)

To provide a more reliable assessment, we have performed in vitro cell culture experiments. In four parallel experiments, human umbilical vein endothelial cells (HUVECs) were co-cultured with the CMC-Cu(II) membrane for 24 hours. The results of fluorescence staining images from live/dead cell assays (**Supplementary Fig. 21**) demonstrate that the CMC-Cu membranes exhibit negligible cytotoxicity, thereby illustrating the good biocompatibility of the CMC-Cu(II) electrode. Detailed experimental processes and results are described in the Methods and Results sections of the revised manuscript. This experiment was carried out by the Key Laboratory of Biomedical Information Engineering of Ministry of Education, School of Life Science and Technology, Xi'an Jiaotong University.

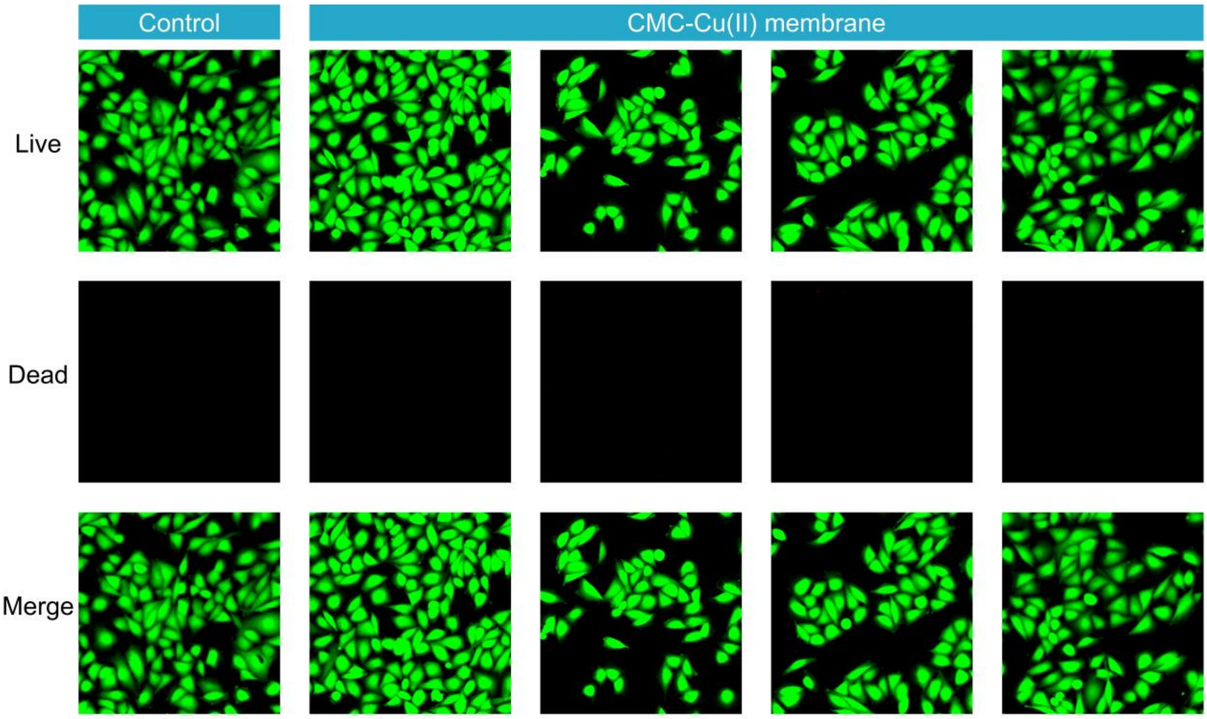

Supplementary Fig. 21. Biocompatibility evaluation of CMC-Cu(II) membrane. Representative fluorescence staining images showing live (green) and dead (red) HUVECs cells cultured without (Control) and with CMC-Cu(II) membrane (four parallel experiments).

Updates to the revised manuscript: According to Reviewer #2's comments, we added the detailed experimental procedures about vitro cell culture experiment of CMC-Cu membrane in the revised manuscript on Page 28.

> *"**Biocompatibility test***
>
> *Human umbilical vein endothelial cells (HUVECs) were seeded in Dulbecco's modified Eagle's medium supplemented with 10% fetal bovine serum, 100 units/mL penicillin, and 100 μg/mL streptomycin.The cells were maintained under either blank control conditions or in the presence of a CMC-Cu(II) membrane (5 mm × 5 mm) in a humidified incubator at 37°C with 95% humidity and 5% CO_2. After 24 h, the cells were gently washed twice with phosphate-buffered saline and then stained with Calcein AM and propidium iodide for 30 min at 37 °C. Following staining, samples were immediately visualized using a confocal laser scanning microscope[37]."*

Updates to the revised manuscript: We added the above results about the biocompatibility test of CMC-Cu membrane in the revised manuscript on Page 16.

> *"Therefore, biocompatibility tests of the CMC-Cu(II) membrane were conducted. After culturing HUVECs for 24 hours, both the blank control group and the CMC-Cu(II) membrane exhibited excellent cellular compatibility (**Supplementary Fig. 21**). These findings suggest that the CMC-Cu(II) electrode employed in this study poses negligible cytotoxic risk, supporting its safe application in wearable bioelectronics requiring prolonged skin contact."*

3. The manuscript claims the CMC-Cu(II) membrane remains stable in aqueous environments like sweat for 30 days. Can authors show experimental evidence that support this claim? How does the membrane's performance (e.g., conductivity, adhesion) hold up under dynamic conditions such as mechanical stretching, sweating, or prolonged wear?

Author reply: We appreciate the reviewer's valuable question regarding the stability and performance of the CMC-Cu(II) membrane under dynamic conditions. The supplementary experimental results demonstrate that the CMC-Cu(II) membrane maintains stable performance over a 30-day period. Although prolonged wear (24 hours) led to reduced electrical conductivity and adhesion due to water loss, the membrane remained functional and continued to meet the requirements for routine physiological signal monitoring. Under other tested conditions, both electrical properties and adhesion remain unaffected, even exposure to sweat was found to enhance the membrane's electrical performance. Detailed results and analyses are as follows.

(i) 30-day stability in aqueous environments.

We conducted a comprehensive stability test over a period of 30 days in water environments. The experimental results show that when the CMC-Cu(II) membrane was placed in an aqueous environment, it only exhibited area contraction over a 30-day period, with no detectable free Cu(II) ions released into the surrounding water. Specifically, digital images and morphological observations indicated a gradual size reduction over 30 days of immersion; however, the membrane retained its overall structure and shape without signs of disintegration or deformation (**Figure R21a**). Given that membrane formation is based on strong coordination between CMC and Cu(II), we further evaluated the potential ion release by analyzing the soaking solution via UV spectroscopy. While a distinct absorption peak at 194 nm was observed in the Cu(II) reference solution, no corresponding peak appeared in the soaking water, even after 30 days (**Figure R21b**). These results indicate the good stability and low environmental impact of the CMC-Cu(II) membrane.

(ii) Electrical performance under dynamic conditions.

In the revised supplementary materials, we have included additional electrical data for CMC-Cu(II) obtained under dynamic conditions (**Supplementary Fig. 23**). The results demonstrate that none of the tested conditions compromise the functionality of the CMC-Cu(II) epidermal electrode, and sweating even enhances its electrical performance. Specifically, the membrane's

electrical properties were evaluated under mechanical stretching-compression, sweating and prolonged wear conditions. After stretching and compression (**Supplementary Fig. 23a inset i-iv**), the impedance shows negligible changes, indicating that the membrane retained its electrical conductivity after mechanical operation (**Supplementary Fig. 23a and b**). Upon exposure to sweat, an increase in capacitance and a decrease in resistance are observed at 20 Hz, indicating a reduction in impedance (**Supplementary Fig. 23c and d**). This behavior is likely attributed to the conductive nature of sweat and the presence of trace ions, such as Na(I), which enhance ionic mobility within the system. After 24 hours of wear, a slight increase in impedance is observed. Specifically, under the test conditions, a series of electrical property measurements—impedance, capacitance, and resistance—were systematically conducted on the CMC-Cu(II) electrode every 3 hours. The impedance values at 20 Hz for the samples at 0 h, 3 h, 6 h, 9 h, 12 h, and 24 h are recorded as 9.75×104 Ω, 9.98×104 Ω, 9.99×104 Ω, 1.38×105 Ω, 1.36×105 Ω, and 2.21×105 Ω, respectively (**Supplementary Fig. 23e and f**). Both capacitance and resistance exhibited consistent and expected trends in accordance with Eq 1 (**Supplementary Fig. 23f**). The impedance of the CMC-Cu(II) electrode exhibits a slight increase within the first 6 hours, followed by a more substantial rise after 12 hours, and a more pronounced rise by 24 hours; however, it remains lower than that of the commercial electrode. This indicates that the physiological signal monitoring performance of the CMC-Cu(II) electrode experiences a very slight decrease within 6 hours, followed by a partial attenuation within 12 hours, and remains fully adequate for reliable, conventional physiological monitoring. These are expected due to the loss of water and environmental conditions.

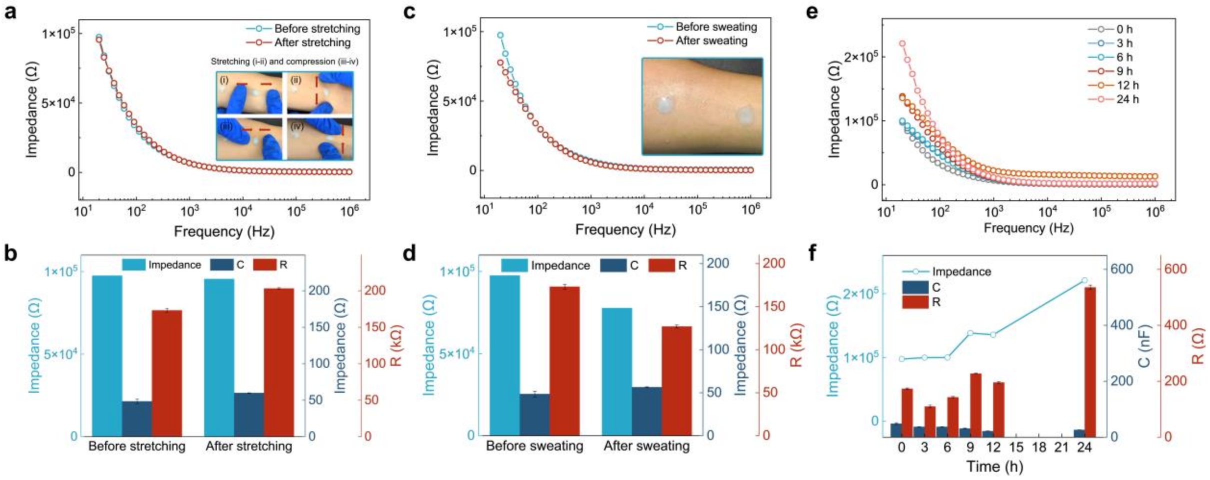

Supplementary Fig. 23. The electrical performances of CMC-Cu(II) membrane under dynamic conditions. a Skin-contact impedance values within 20 Hz to 1000 kHz of CMC-Cu(II) electrode before and after mechanical operation (stretching-compression). **b** Skin-contact impedance value at 20 Hz, capacitance, and resistance of CMC-Cu(II) electrodes before and after mechanical operation (stretching-compression). **c** Skin-contact impedance values within 20 Hz to 1000 kHz of CMC-Cu(II) electrode before and after sweating. **d** Skin-contact impedance value at 20 Hz, capacitance, and resistance of CMC-Cu(II) electrodes before and after sweating. **e** Skin-contact impedance values within 20 Hz to 1000 kHz of CMC-Cu(II) electrode within 24 h. **f** Skin-contact impedance value at 20 Hz, capacitance, and resistance of CMC-Cu(II) electrodes within 24 h.

Updates to the revised manuscript: According to Reviewer #2's comments, we added above results on pages 17.

> *"Furthermore, practical applications of physiological signal monitoring frequently involve dynamic and changing environments, such as mechanical stretching, compression, and exposure to sweat. To evaluate the functional stability of CMC-Cu(II) under such dynamic conditions, its electrical performance (impedance) was measured following repeated mechanical deformation, sweat exposure, and prolonged wear (24 h) testing (**Supplementary Fig. 23**). After stretching and compression (**Supplementary Fig. 23a inset i-iv**), the impedance shows negligible changes, indicating that the membrane retained its electrical conductivity after mechanical operation (**Supplementary Fig. 23a and b**). Upon exposure to sweat, an increase in capacitance and a decrease in resistance (**Supplementary Fig. 23d**) are observed at 20 Hz, indicating a reduction in impedance (**Supplementary Fig. 23c**). This behavior is likely attributed to the conductive nature of sweat and the presence of trace ions, such as Na(I), which enhance ionic mobility within the system. After 24 hours of wear, a slight increase in impedance is observed. Specifically, under the test conditions, a series of electrical property measurements, impedance, capacitance, and resistance, were systematically conducted on the CMC-Cu(II) electrode every 3 hours. The impedance values at 20 Hz for the samples at 0 h, 3 h, 6 h, 9 h, 12 h, and 24 h are recorded as 9.75×104 Ω, 9.98×104 Ω, 9.99×104 Ω, 1.38×105 Ω, 1.36×105 Ω, and 2.21×105 Ω, respectively ((**Supplementary Fig. 23e and f**)). The impedance of the CMC-Cu(II) electrode exhibits a slight increase within the first 6 hours, followed by a more substantial rise after 12 hours, and a more pronounced rise by 24 hours; however, it remains lower than that of the commercial electrode. This indicates that the physiological signal monitoring performance of the CMC-Cu(II) electrode experiences a very slight decrease within 6 hours, followed by a partial attenuation within 12 hours, and remains fully adequate for reliable, conventional physiological monitoring. These are expected due to the loss of water and environmental conditions."*

(iii) Adhesion performance under dynamic conditions.

The adhesion performance was assessed before and after mechanical deformations (i.e., stretching and compression) as well as following 24 hours of wear. No significant deterioration in adhesion (6.03 N/m) is observed after mechanical deformation compared with original adhesion force (6.05 N/m), indicating robust mechanical stability (**Figure R22a)**. However, a slight reduction in adhesion (4.03 N/m) is noted after 24 hours of continuous wear, which can be attributed to membrane drying resulting from moisture loss (**Figure R22b)**. Given the impracticality of performing a direct 90° peeling test on human skin, changes in adhesion and conformability under sweating conditions were inferred indirectly through impedance measurements. The absence of a significant drop in impedance suggests that sweat did not substantially compromise the membrane's adhesion or its conformal behavior to the skin surface.

These tests collectively demonstrate that the CMC-Cu(II) membrane maintains both structural integrity and electrical stability under prolonged exposure to sweat, mechanical stress, and extended wear, confirming its potential for use in epidermal electronic applications.

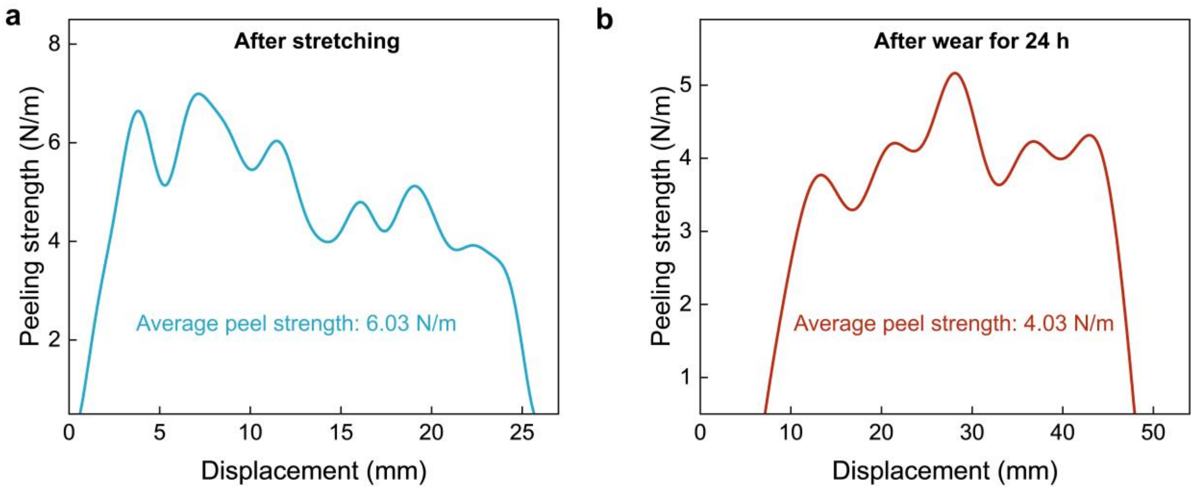

Figure R22. The adhesion performances of CMC-Cu(II) membrane under dynamic conditions. a Peeling strength of the CMC-Cu(II) membrane by 90° peeling test after mechanical operation (stretching). **b** Peeling strength of the CMC-Cu(II) membrane by 90° peeling test before and after 24-h wearing.

4. While the "dipping-dipping" process is rapid and simple, how feasible is it for large-scale manufacturing or clinical applications? What challenges are there in achieving uniform membrane thickness or patterning complex circuits over large or irregular surfaces? How might these affect practical deployment?

Author reply: The "dipping-dipping" process is indeed attractive for its rapidity, simplicity, and low-cost nature, which makes it conceptually scalable for large-area fabrication.

(i) Scalability and uniformity.

In our experiments, the membrane thickness was controlled by adjusting the solution concentration (**Figure R23a and b**). While achieving perfect uniformity over large areas remains challenging, due to the requirement for simultaneous contact between Cu(II) and all regions of the CMC under laboratory conditions, this limitation can be mitigated by leveraging established coating and printing techniques, such as inkjet deposition, which are fully compatible with our aqueous chemical system.

(ii) Complex patterns and irregular surfaces.

For patterned or circuit-like architectures, the "dipping-dipping" method can be integrated with masking, microcontact printing, or inkjet deposition to enable selective growth of conductive regions. The CMC-Cu(II) chemistry allows conformal coating on irregular or flexible substrates (**Figure R23c**), which is advantageous compared with rigid lithographic methods.

(iii) Practical implications.

These challenges are primarily engineering-related rather than fundamental. While precise control of uniformity and circuit resolution will require optimization, the simplicity, rapidity, and biocompatibility of the "dipping-dipping" approach provide a strong foundation for translation toward large-scale or clinical applications.

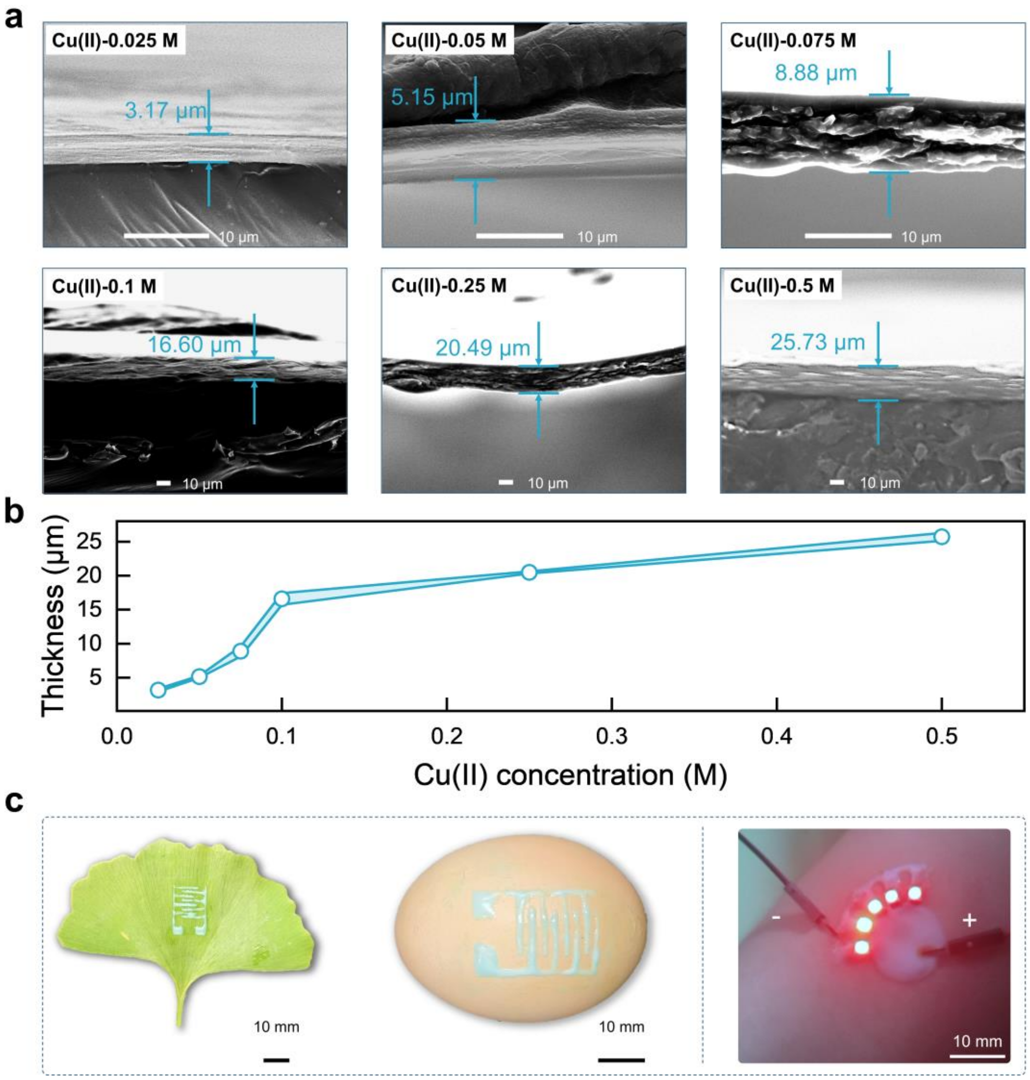

Figure R23. The Control over the thickness and patterning behavior of CMC-Cu(II) membranes. **a** Cross-sectional SEM images of CMC-Cu(II) membranes fabricated using varying concentrations of Cu(II). **b** A positive correlation between the thickness of the CMC-Cu(II) membrane and the concentration of Cu(II). **c** Digital photographs of CMC-Cu(II) patterns on diverse 2D and 3D surfaces, and digital image of CMC-Cu(II) patterned microelectronic circuit on human skin and its ability to illuminate LED lights.

Updates to the revised manuscript: According to Reviewer #2's comments, we added above results about controlling the thickness of CMC-Cu membrane on page 6.

> *"The cross-sectional scanning electron microscopy (SEM) image reveals a 3.4-μm-thick CMC-Cu (II) membrane (**Fig. 1d**) fabricated from a 1 wt% CMC solution and 0.5 M Cu(II) solution. The "thin" feature of the CMC-Cu(II) membrane further enhances its ability to conform to and match various interfaces[27, 28]. It is noteworthy that the thickness of the CMC-Cu(II) membrane can be tuned by varying the Cu(II) concentration. As clearly illustrated in the SEM cross-sectional images presented in **Supplementary Fig. 3**, the membrane thickness exhibits a positive correlation with rising Cu(II) concentration."*

5.Authors note that Ag(I), despite its +1 oxidation state, forms stable membranes comparable to Cu(II). What is the chemical or structural basis for this unexpected performance? Is it due to differences in coordination geometry, binding energy, or other factors? How does this impact the generalizability of the metal coordination strategy?

Author replay: Thank you for raising this insightful scientific question. The reviewer pointed out that Ag(I) can still form a membrane as stable as that of Cu(II) in the +1 oxidation state. This phenomenon exhibits a certain degree of uniqueness. We believe that the chemical and structural basis of this phenomenon can be explained from three aspects: the coordination geometric characteristics of the metal ions, the electronic structure, and the oxidation properties.

(i) According to our DFT simulation results, the CMC-Cu(II) complex presents a bidentate coordination tetrahedron structure, where the carboxyl oxygen atom forms a strong coordination bond with Cu(II) (Please reference **Figure R5d** on Page 8). This geometric configuration offers a higher crystal field stabilization energy and structural rigidity, leading to a dense and stable membrane structure. In contrast, despite Ag(I) having a +1 valence and its d^{10} electronic configuration resulting in a lower crystal field stabilization energy, Ag(I) possesses strong polarizability and oxidizing ability (Standard electrode potentials of Ag/Ag(I) and Cu/Cu(II) are +0.799 V and +0.340 V respectively). This can effectively induce the rearrangement of the electron cloud of the carboxylate group, facilitating interfacial electron transfer and ionic cross-linking[11-13].

(ii) From the perspective of coordination geometry, Ag(I) tends to form linear or approximately planar tetra-coordination structures and establishes flexible bidentate or bridging coordination with the carboxyl oxygen atoms on CMC. Although the coordination energy of this coordination mode is slightly lower than that of Cu(II), its softer binding and easier rearrangement at the interface enable it to rapidly construct continuous metal-polymer network layers[11-13]. Therefore, Ag(I) can also achieve interface gelation and solid membrane formation upon contact.

In summary, the similarity in the membrane-forming behavior of Cu(II) and Ag(I) lies in the fact that they can both form stable bidentate or bridging coordination structures with the carboxyl groups of CMC, but their driving forces differ: the membrane formation of Cu(II) is mainly governed by the strength of coordination bonds and geometric stability; the membrane formation of Ag(I) is mainly driven by oxidizing power and polarization effects.

Therefore, the "metal ion-induced coordination to membrane formation" mechanism we proposed is not solely reliant on the coordination ability itself but is also intricately associated with the oxidizing power, polarizing property of the ions, and the interface reaction kinetics. This understanding further suggests that this strategy possesses a certain degree of universality: as long as the metal ions can induce electron rearrangement at the interface and establish effective cross-links with the polymer carboxyl groups, a rapid and stable film formation process can be realized.

References:

11. Yoshinari N, Konno T. Multitopic metal – organic carboxylates available as supramolecular building units. *Coordination Chemistry Reviews* **474**, 214850 (2023).
12. Dudev T, Lim C. Effect of Carboxylate-Binding Mode on Metal Binding/Selectivity and Function in Proteins. *Accounts of Chemical Research* **40**, 85-93 (2007).
13. Kim M, Lee Y, Moon HR. Carboxylate-Based Metal – Organic Framework and Coordination Polymer Glasses: Progress and Perspectives. *Accounts of Chemical Research* **57**, 2347-2357 (2024).

Reviewer #3 (Remarks to the Author):

This paper proposes metal-coordination bond assisted rapid-curable, in-situ forming ultrathin hydrogel for bio-electronic skin, showing its applications of monitoring various biopotential signals. Metal coordination bond-assisted rapid-curing of hydrogel allows in-situ formation of ultrathin conductive hydrogel on curved surface with any shape desired. Cu(II), Ag(I) metal ion coordination bond provides superior curing speed of hydrogel compared with other metal ions, and its mechanism is verified by experiments and density functional theory simulation. Various biopotential signals (EOG, EEG, ECG, EMG) were monitored with conductive bioelectrode and showed superior contact impedance and SNR compared to commercial Ag/AgCl bioelectrode, owing to its high conformability. In fact, curing of cellulose-based material by Cu(II) ion is already reported in previous literature (Nature 598, 590–596 (2021), Nat. Nanotechnol. 18, 168–176 (2023), Nat. Nanotechnol. 17, 629–636 (2022))), which were cited as references in the manuscript. So, the approach of utilizing metal coordination bond with Cu(II) ion and cellulose is not novel by itself. The novelty of this manuscript lies in rapid curing approach. However, the manuscript does not provide sufficient data to support this result. The density functional theory simulation and X-ray absorption spectroscopy analysis is not sufficient to explain "speed" of curing by metal ion coordination bond. They only show that CMC-Cu(II) and CMC-Ag(I) will be cured to form hydrogel by metal-ion coordination bond, which is already shown in previous literature. More analysis and discussion must be done to provide a mechanism to explain faster of hydrogel curing by Cu(II) and Ag(I) ions. In addition, there are several missing points in this manuscript, which are crucial for bioelectrode applications. (No electrical and mechanical properties characterization of hydrogel; Adhesion characteristics of hydrogel; no details of the method for in-situ fabrication of hydrogel on skin like face and neck; no EOG/EEG performance comparison with Ag/AgCl electrodes) Therefore, based on the amount of novelty and lack of supporting data, this manuscript is not suitable to be published in Nature Communications.

Author reply: We sincerely thank the reviewer for the careful evaluation of our manuscript and for the constructive comments, which significantly help improve the quality and clarity of our work. The reviewer has raised insightful points in three key areas:
(i) concerns regarding the novelty of our work, particularly related to previously reported studies on cellulose–copper coordination;
(ii) the importance of elucidating the mechanism underlying the rapid membrane formation process;
(iii) suggestions for additional experimental validation.
Below, we address these issues point-by-point, starting with a clarification of our work's novelty and how it differs from the cited literature.

1. Clarifying the Novelty of Our Work in Comparison to Prior Studies

In our introduction, we cited three relevant papers — *Nature* 598, 590–596 (2021), *Nat. Nanotechnol.* 18, 168–176 (2023), and *Nat. Nanotechnol.* 17, 629–636 (2022). You raised the concern that these might undermine the significance of our work's novelty. We therefore elaborate from two perspectives: (i) the differences between our work and the cited literature;

and (ii) the novelty of our work.

iii. The differences between our work and the cited literature

The three cited studies and our work all exploit coordination interactions between biomass-derived materials and Cu(II) for specific functions. However, our approach differs fundamentally. First, the cited studies use biomass-based membranes to interact with Cu(II) and thereby impart functionality, whereas our work uses a biomass-based solution that interacts with Cu(II) to form a conformal membrane first, which is then used as an epidermal electrode (**Figure R24**). Second, even when placing the biomass materials from the cited studies into a solution/suspension state analogous to our CMC solution and allowing them to interact with Cu(II), those materials do not produce conformal membranes as in our work (**Figure R25**). We elaborate these points as follows.

- **Conceptual and Methodological Differences: Prior literature follows a membrane → functional membrane route; our approach is solution → functional membrane.**

  The cited studies all begin with a pre-formed biomass membrane, which then interacts with Cu(II) to confer specific properties, whereas our work begins with a biomass solution that forms an in-situ ultrathin, conformal membrane upon contact with Cu(II), which we then use as an epidermal electrode (**Figure R24**).

  Specifically:

  (1) *Nature* (2021)[14]: CNF (cellulose nanofiber) paper is first prepared by vacuum filtration of a CNF suspension, then immersed in a Cu(II)-saturated alkaline solution (produced by immersing excess copper wire in NaOH). The interaction builds molecular channels within cellulose, enabling rapid Li^+ transport for high-performance solid polymer ion conductors.

  (2) *Nat. Nanotechnol.* (2023)[15]: A cotton textile is immersed in a blue Cu(II)-saturated aqueous NaOH solution; the interaction imparts antiviral/antibacterial performance, excellent air/water retention, and superior mechanical stability.

  (3) *Nat. Nanotechnol.* (2022)[16]: A chitosan membrane is prepared by casting (chitosan dissolved in acetic acid) and then immersed in saturated $Na_2Cu(OH)_4$ to form a chitosan–Cu membrane. Coordination on one side produces hexagonal nanochannels for water diffusion and fast ion transport; on the other side enhances mechanical strength, reduces permeability, and improves alkaline stability for a high-performance hydroxide exchange membrane.

  From these descriptions, it is clear that the cited works all follow a pre-formed membrane (paper/textile/membrane) → Cu(II) treatment route to improve properties. By contrast, our work emphasizes that a biomass solution (CMC) can directly produce an in-situ ultrathin, conformal membrane on the target substrate upon contact with Cu(II), without first fabricating a CMC membrane. These features are not only unique, but also align well with the requirements for epidermal electrodes, as detailed later in the section "The novelty of our work." Moreover, compared with CNF papers, textiles, or cast chitosan membranes (which require additional preparation steps), a CMC solution is prepared simply and quickly by dissolving CMC in water.

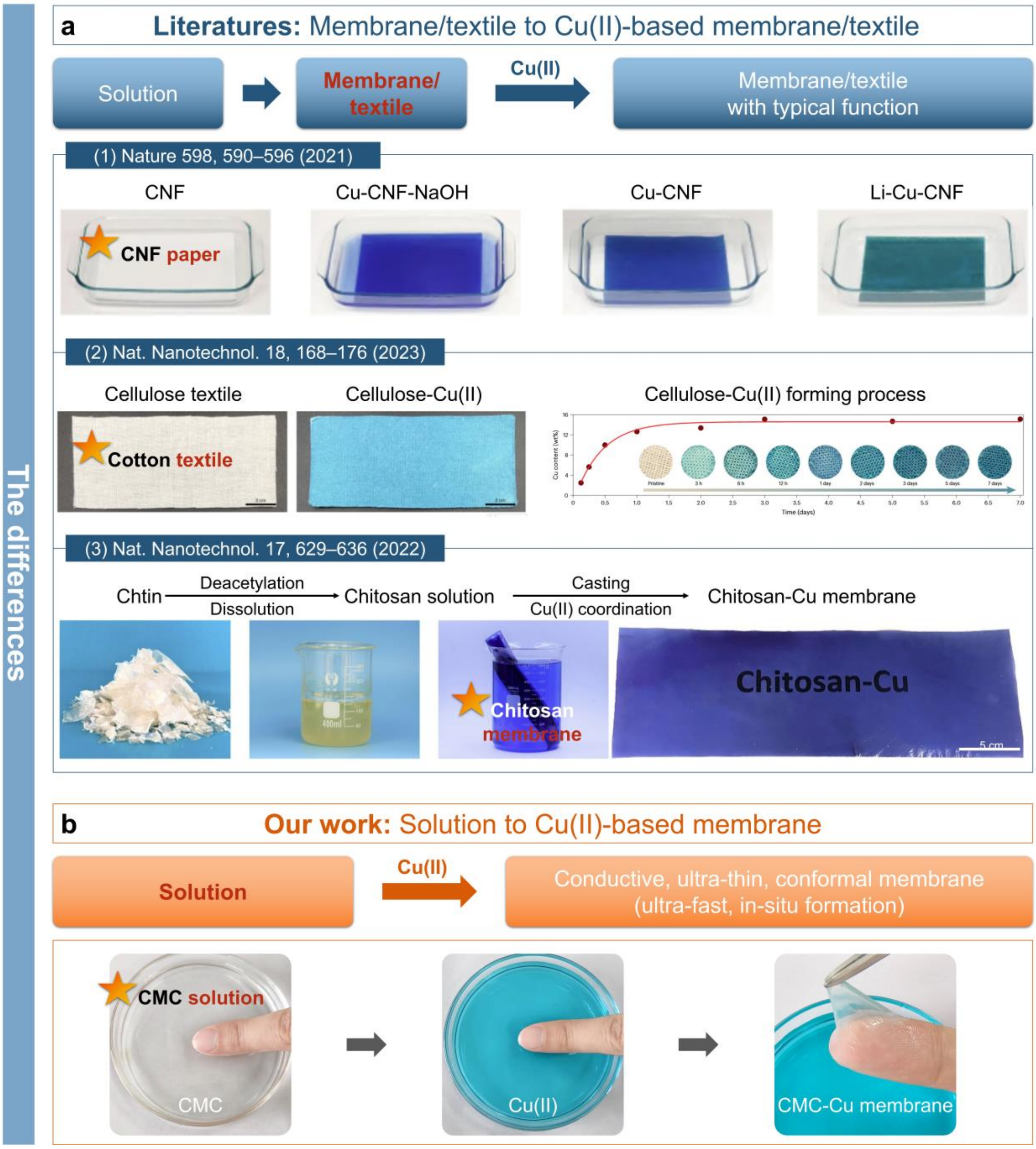

Figure R24. The differences between our work and the cited literature a The cited literatures use biomass-based membranes to interact with Cu(II) and thereby impart functionality. **b** Our work uses a biomass-based solution that interacts with Cu(II) to form a conformal membrane.

- **Comparison Under Identical Solution/Suspension-State Conditions: Even after converting prior materials to solution/suspensions, they do not form conformal membranes upon Cu(II) interaction, whereas CMC-Cu(II) does.**

  To further investigate the advantages and novelty of our approach relative to previous studies, we prepared CNF, dissolved cellulose, and chitosan as solutions/suspensions and exposed them to Cu(II) using the same "dipping–dipping" method. Unlike the CMC-Cu(II) system, which rapidly forms an ultrathin, conformal membrane within ~2 s and enables

seamless in-situ fabrication on various biological substrates, these other systems (CNF–Cu(II), dissolved cellulose-Cu(II), chitosan-Cu(II)) produced high-viscosity gel-like substances over the same period (**Figure R25**), lacking the structural integrity required for membrane formation or epidermal integration. Therefore, the membrane-forming strategy of the CMC-Cu(II) system is intrinsically and highly novel.

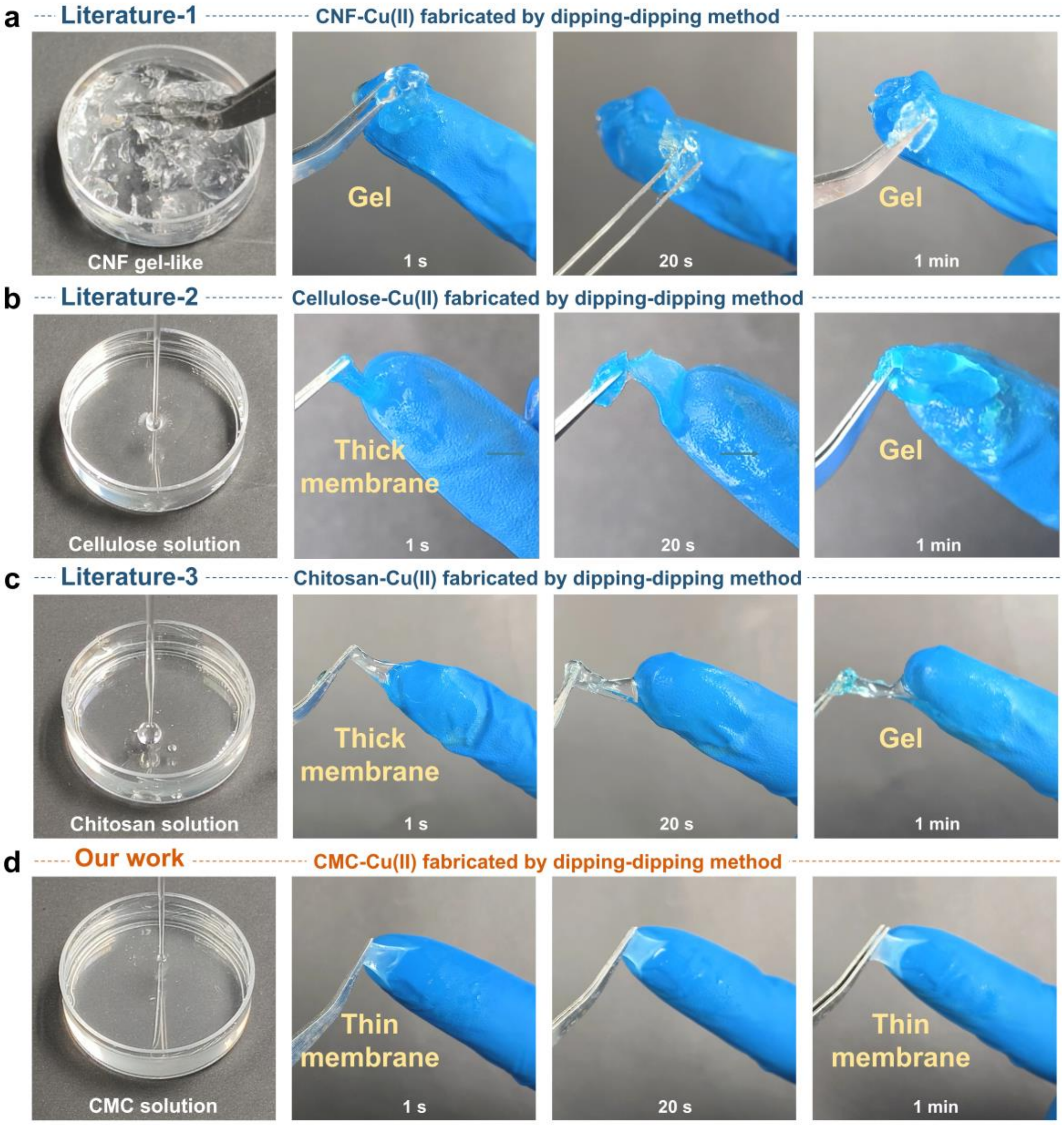

Figure R25. The products obtained by converting various biomass materials in the cited studies into a solution/suspension state and allowing them to interact with copper(II) ions are different from our CMC-Cu(II) ultrathin membranes. a The gel-like CNF suspension interacts with Cu(II) to form CNF-Cu(II) gel. **b** The cellulose solution interacts with Cu(II) to form thick Cellulose-Cu(II) membrane. **c** The chitosan solution interacts with Cu(II) to form thick Chitosan-Cu(II) membrane. **d** CMC solution in our work interacts with Cu(II) to form ultrathin conformal membrane.

Apart from the above-mentioned differences, our work specifically targets use as an epidermal electrode for physiological monitoring, leveraging the unique attributes of the

dipping-dipping method and the advantages of the resulting ultrathin, conformal membrane. A detailed comparison between prior literature and our approach is provided in **Table R2**, and a full explanation of the novelty and the mechanism underlying this unique phenomenon is presented in the following sections of this response letter and in the revised manuscript.

Table R2. A detailed comparison between prior literature and our approach

	Materials A interacts with Cu(II)	State of Materials A	Chemical structure of Material A	Function of the coordination of Material A-Cu(II)	Application of Material A-Cu(II)	Can Material A in solution/suspension state form a thin membrane when it contacts Cu(II)
**(1) Nature (2021)**	CNF **paper**	Solid	-OH	Form molecular channels for ion transport	For solid-state batteries	No
**(2) Nat. Nanotechnol. (2023)**	Cotton **textile**	Solid	-OH	For antiviral and antibacterial functions	As antiviral and antibacterial textiles for use in household products	No
**(3) Nat. Nanotechnol. (2022)**	Chitosan **membrane**	Solid	-OH, $-NH_2$	Form molecular channels for ion transport	As ion exchange membranes	No
**Our work**	CMC **solution**	**Liquid**	**-COOH**	**Assemble molecules into the conductive membrane through solid-liquid transformation**	As **epidermal electronics**	**Yes**

iv. The novelty of our work

Based on the above comparison with the previous literature, it can be concluded that our CMC-Cu(II) approach constitutes a unique membrane-forming strategy, and the novelty of our work includes (**Figure R18 and R3**): (i) developing an ultra-fast (≈2 s), highly convenient in-situ membrane formation strategy, whose mechanism has been systematically investigated and has guided us to expand the method to other systems; (ii) the achieved membrane exhibits various

advantageous features, including ultrathin thickness (~3.4 μm), conformality, patternability, a transfer-free process (no additional post-transfer step), conductivity, low impedance, biocompatibility, and biodegradability, which fit perfectly for epidermal electrodes for physiological monitoring. Detailed explanations are as follows.

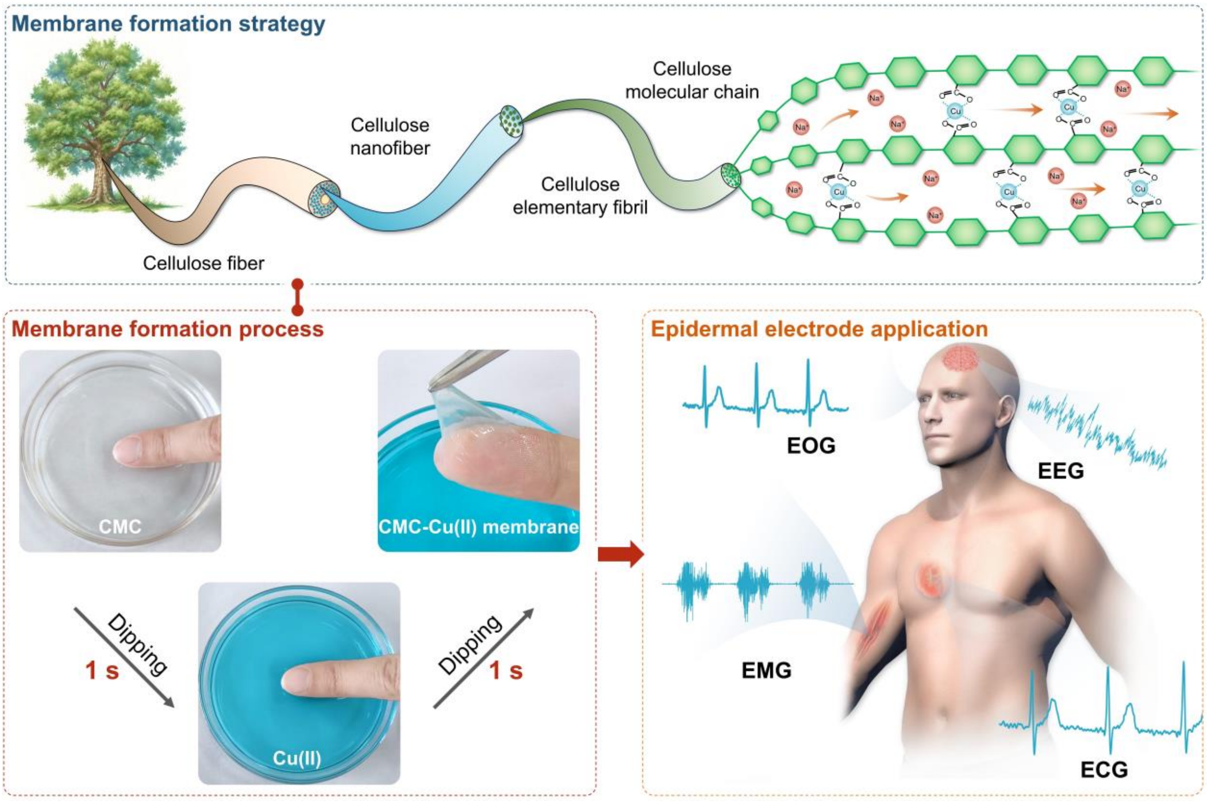

Figure R18. The unique membrane-forming strategy constituted by the CMC–Cu(II) approach for epidermal electrode.

- **<u>Unique membrane formation technology.</u>** The key novelty of this study is a facile "dipping-dipping" molecular-assembly method using the CMC-Cu(II) system that enables an ultra-fast liquid-to-solid transformation. Unlike traditional routes of casting → drying/curing → transfer, which are complicated, our approach directly forms the bio-membrane in situ by sequentially dipping the target surface into CMC solution and then Cu(II), which is highly convenient and time-saving. The rapid solidification is driven by strong coordination and accelerated interfacial assembly, elucidated by experiments (UV–vis, XANES, EXAFS) and simulations (MDS—captures nanosecond interfacial assembly kinetics; DFT—quantifies coordination structures and binding energies consistent with XANES/EXAFS). We further generalize the strategy to other metal ions (Ca(II), Fe(II), Ag(I)) and other biomacromolecules (MC, CMCH), establishing design rules that relate coordination energetics and interfacial assembly dynamics to membrane-forming efficacy, thereby delivering a transfer-free, substrate-agnostic, seconds-scale route to ultrathin, conformal membranes with tunable performance for bio-integrated electronics (**Figure R3**).

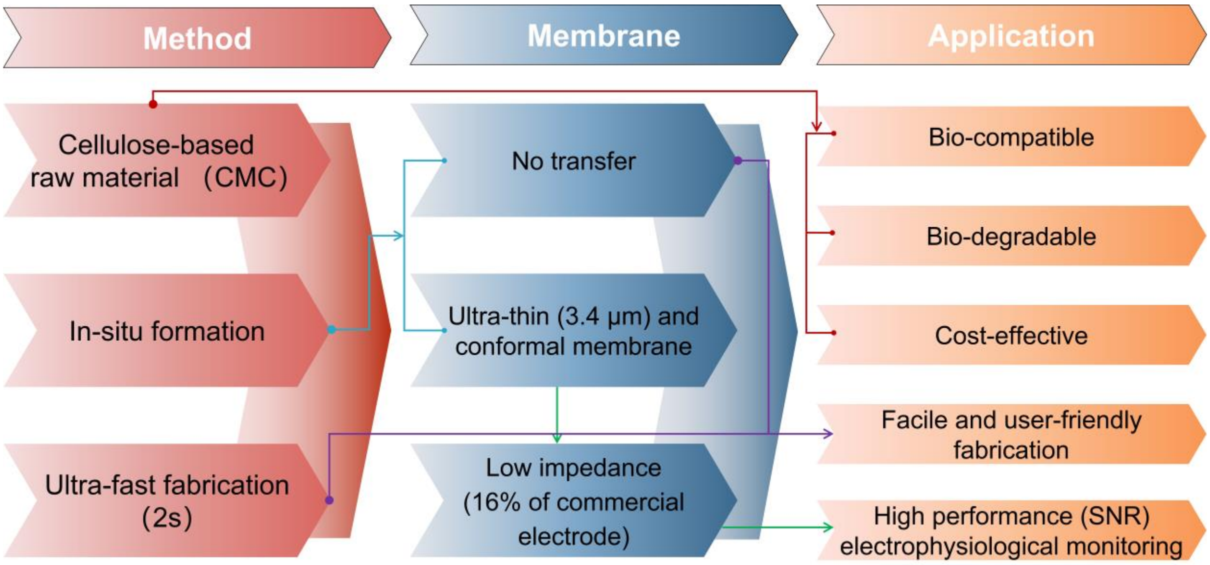

Figure R3. Illustration of the CMC-Cu(II) advantages for epidermal electronics from method to membrane and epidermal electrode applications.

- **Multiple advantageous features that perfectly suit epidermal electrodes.** The method—and the resulting membrane—offer a suite of advantages that meet the core requirements of epidermal electrodes (**Figure R3 and R4**). From the materials perspective, the membrane is ultrathin and conformal, enabling low interfacial impedance and a high signal-to-noise ratio for high-performance bioelectrical measurements—its most critical attribute. In addition, being derived from a cellulose derivative, the membrane is biocompatible and biodegradable, making it cost-effective, skin-benign, and environmentally friendly. From the manufacturing perspective, the process is ultra-fast and convenient; the in-situ formation strategy eliminates transfer steps that could damage fragile membranes, and its inherent patternability broadens application scenarios. Collectively, these features make the membrane an excellent fit for high-fidelity acquisition of physiological signals (ECG, EOG, EMG, EEG).

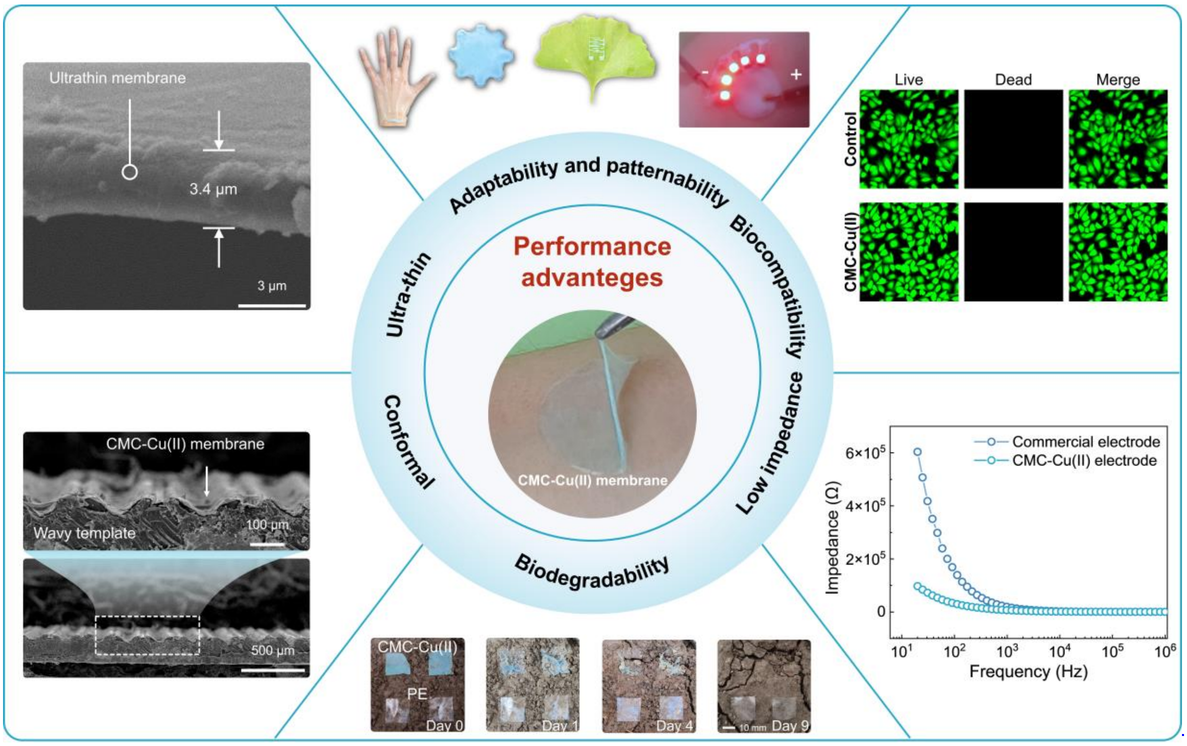

Figure R4. The combination of attributes about functional performance of CMC-Cu(II) membrane involving ultrathin, conformal, adaptive, patternable, degradable, biocompatible and low impedance characteristics.

In conclusion, this work presents a novel nature-inspired "dipping-dipping" assembly method and systematically validates its scientific foundation and practical significance through systemic evaluations of performance, underlying mechanisms, and application potential. Furthermore, the approach demonstrates broad universality and scalability. We believe this work possesses advancement in the field of "controlled preparation methods for flexible electronic materials," offering clear innovation and translational potential.

References:

14. Yang C, *et al.* Copper-coordinated cellulose ion conductors for solid-state batteries. *Nature* **598**, 590-596 (2021).
15. Qian J, *et al.* Highly stable, antiviral, antibacterial cotton textiles via molecular engineering. *Nat. Nanotechnol.* **18**, 168-176 (2023).
16. Wu M, *et al.* A high-performance hydroxide exchange membrane enabled by Cu2+-crosslinked chitosan. *Nat. Nanotechnol.* **17**, 629-636 (2022).

2. The mechanism investigation of the fast CMC-Cu(II) coordination

Thank you for your valuable suggestions. The issue you raised about the "speed" mechanism of the CMC-Cu(II) coordination bond formation into membranes, which the current data cannot fully account for, is of utmost importance. We fully agreed. "Fast curing" facilitates the formation of uniform and well-structured “thin” membrane and improves the processing convenience and applicability of the membrane in epidermal electronic application scenarios. Therefore, to further clarify this dynamic mechanism, in the revised version:

(1) We supplemented molecular dynamics simulations (MDS) to directly demonstrate the ultra-fast molecular assembly speed (40 ns) of CMC-Cu(II).
(2) The MDS-derived data and existing synchrotron X-ray absorption spectroscopy (XAS) have disclosed the detailed actual coordination information within CMC-Cu(II).
(3) The UV spectra results indicate that the characteristic peaks of CMC-Cu(II) remain unchanged over time, which corroborates the ultra-fast coordination between Cu(II) and CMC.
(4) We re-conducted systematic density functional theory (DFT) simulation and calculation of binding energies, which reveals that the higher binding energy of CMC-Cu(II) is the primary underlying mechanism for its extremely rapid membrane-formation speed, from a thermodynamic perspective.
By cross-validating the simulation and calculation results with the UV spectra and XAS analysis results, we systematically revealed the mechanism of CMC-Cu(II) coordination interaction, especially its ultrafast “speed” of interface liquid-solid transformation.
Specifically:

- **MDS:** As shown in **Figure R5a**, the simulation snapshot results reveal that, the CMC molecules initially dispersed in the aqueous environment and rapidly aggregated within 40 ns under the strong coordination forces of Cu(II), thereby completing molecular assembly and forming a solid-state thin membrane. This result directly illustrates the ultra-fast assembly “speed” of CMC under the coordination of Cu(II).
- **MDS and XAFS:** As shown in **Figure R5b**, Quantitative analysis of the interaction between carboxyl (-COO) and hydroxyl (-OH) groups in the CMC chain and copper (II) ions was performed using radial distribution function data from MDS. The results showed a stronger coordination tendency between Cu(II) and carboxyl groups, indicated by higher peak intensity in the distribution function, suggesting that CMC assembly is primarily driven by Cu(II)-carboxyl coordination rather than Cu(II)-hydroxyl coordination.
  Copper K-edge XANES results show that the electronic state of Cu in CMC-Cu(II) closely resembles that of CuO. The presence of a characteristic Cu(II) absorption peak confirms copper is in the +2 oxidation state without further chemical changes. EXAFS fitting analyses reveal that Cu(II) forms two oxygen-coordinated types with CMC, with coordination numbers of 2.2 ± 0.6 and 1.8 ± 0.7 (**Supplementary Table 2**), summing to approximately 4.
  The results of MDS and XAFS suggest that Cu(II) forms a local coordination structure closed to the square planar with carboxyl groups of CMC by bidentate coordination mode.
- **UV-spectra:** As shown in **Figure R5c**, Firstly, when compared with the UV characteristic peaks of CMC and Cu(II), a new UV absorption peak emerged in CMC -Cu(II), which demonstrated the formation of a "new substance", specifically the CMC-Cu(II) complex. Furthermore, above the mixtures of the three metal ions (Cu(II), Fe(II), and Ca(II)) with CMC, the UV absorption peak of CMC-Cu(II) remains completely unchanged over time, indicating Cu(II) and CMC forming a complete coordination bond immediately upon contact. The peak shape and position of CMC-Fe(II) have changed significantly over time, demonstrating Fe(II) consistently reacts with CMC, yet not at a rapid speed. In CMC-Ca(II), since its

coordination effect is too weak and no typical UV absorption peak is observed, it shows a different behavior. This result further corroborates the ultra-fast coordination speed between Cu(II) and CMC.

- **DFT:** As shown in **Figure R5d**, the structures of CMC-Cu(II), CMC-Fe(II), and CMC-Ca(II) were optimized, and their binding energies were calculated. The results indicate that the binding energies of these three compounds are -5.82 eV, -2.16 eV, and -1.15 eV, respectively. A negative binding energy suggests that the coordination reactions of the three systems occur spontaneously. The larger absolute value of the binding energy from CMC - Cu(II) further demonstrates that Cu(II) possesses a stronger coordination ability, which is the underlying reason for the rapidity of the reaction.

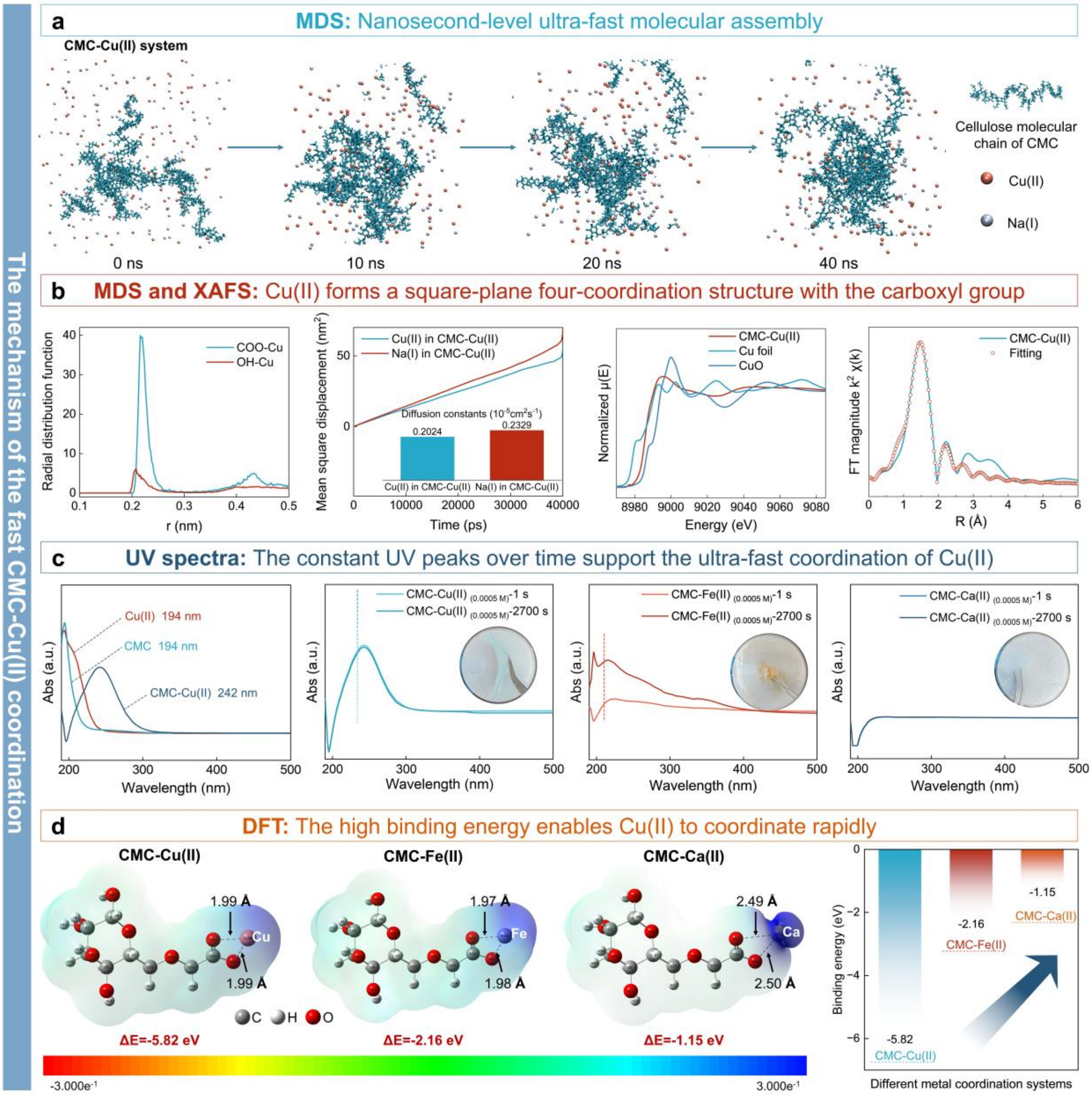

Figure R5. The mechanism characterization and simulation of the fast CMC-Cu(II) coordination. **a** The snapshot of the Cu(II)-induced CMC molecular assembly within 40 ns obtained by the MDS, clearly demonstrates the ultra-fast coordination of CMC-Cu(II). **b** The radial distribution

function of Cu (II), and mean square displacement of Cu(II) and Na(I) around CMC-Cu(II) system from MDS, combined with EXAFS and XANES data from XAFS, show that Cu(II) coordinates with two carboxyl oxygen atoms in CMC to form a nearly square four-coordinate structure. **c** The UV spectra of the three metal ions (Cu(II), Fe(II), and Ca(II)) after mixing with CMC demonstrate that only CMC-Cu(II) displays a characteristic peak that remains unchanged over time, which indicates the rapid coordination of Cu(II) with CMC. **d** The static electron density maps of the coordination structures of CMC-Cu(II), CMC- Fe(II), and CMC-Ca(II), calculated by DFT, along with their corresponding binding energies, show that the absolute value of the binding energy of CMC - Cu(II) is the highest.

Supplementary Table 2 | EXAFS coordination environment analysis of CMC-Cu(II).

Sample	Scattering Pair	CN	R (Å)	σ^2	ΔE_0 (eV)
CMC-Cu(II)	Cu-O	2.2±0.6	2.14±0.01	2.4±0.3	9.0±0.1
	Cu-O	1.8±0.7	2.00±0.01		

CN: coordination number, R: bond distance, σ^2: Debye-Waller factors, and ΔE_0: energy shift.

In the revised manuscript, **Figure R5a** has been substituted with **Fig. 3a**; the two left- most figures in **Figure R5b** (The Radial distribution function and the Average orientation shift) have been supplemented as **Fig. 3b and Fig. 3c**; and **Figure R5d** (The re-run DFT calculation) has been revised to **Fig. 3f**.

Updates to the revised manuscript: According to Reviewer #3's comments, we added the above results in the revised manuscript on Pages 12-14, 29.

"Mechanism characterization and simulation of molecular assembly

*To investigate the influence of Cu(II)'s strong coordination on molecular chain dynamics and ion transport pathways at the molecular level, molecular dynamics simulations were conducted to analyze the structural evolution and ionic transport behavior of the molecular chains in the CMC-Cu system (**Supplementary Table 1**). As shown in **Fig. 3a**, the simulation snapshot results reveal that, the CMC molecules initially dispersed in the aqueous environment and rapidly aggregated within 40 ns under the strong coordination forces of Cu(II), thereby completing molecular assembly and forming a solid-state thin membrane. Furthermore, the interactions between carboxyl (-COO) and hydroxyl (-OH) groups in the CMC molecular chain and Cu(II) are quantitatively analyzed using radial distribution functions. The results indicate a stronger preference of Cu(II) for coordination with -COO groups compared to -OH groups, as evidenced by the higher peak intensity in the distribution function (**Fig. 3b**). This suggests that CMC molecule assembly is primarily driven by the coordination between Cu(II) and -COO groups along the polymer chain. Moreover, the migration behavior of Na(I) and Cu(II) along the CMC chain direction was evaluated by calculating their diffusion coefficients. The calculated diffusion coefficients are 0.20 × 10^{-5} $cm^2 \cdot s^{-1}$ for Cu(II) and 0.23 × 10^{-5} $cm^2 \cdot s^{-1}$ for Na(I) (**Fig. 3c inset**), confirming faster diffusion of Na(I). Additionally, the simulated mean square displacements of Cu(II) and Na(I) in the CMC-Cu(II) system are*

*55.40 nm² and 68.36 nm², respectively (**Fig. 3c**), indicating that Na(I) exhibits a higher displacement rate. This supports the effectiveness of the ionic conduction mechanism in the CMC-Cu(II) system. Collectively, the MDS validate the feasibility of the proposed strategy that utilizing strong Cu(II) coordination to induce ultra-fast molecular assembly for the formation of ultrathin conductive bio-membranes, which offers significant practical advantages for on-demand applications in fields such as biomedicine and wearable electronics.*

*To further reveal and elucidate the detailed structure characteristics and transient assembly mechanism of metal ion coordination membranes, X-ray absorption spectroscopy (XAS) is employed to analyze the Cu valence and coordination state in formed CMC-Cu(II) membrane complex. Furthermore, density functional theory (DFT) calculations are conducted to determine the most stable coordination structures. The coordinated state of copper ions in the CMC-Cu(II) membrane is verified by XAS. The results of the Cu K-edge X-ray absorption near-edge spectrum (XANES; **Fig. 3d**) indicate that the electronic state of the Cu ions in CMC-Cu(II) is closer to the CuO, showing characteristic absorption peaks of Cu(II), which supports that the Cu in CMC-Cu(II) is in a +2 state without other chemical changes. Fitting of the XANES and extended X-ray absorption fine structure (EXAFS; **Fig. 3e and Supplementary Fig. 19**) spectra reveals two Cu-O distances of 2.14 ± 0.01 Å and 2.00 ± 0.01 Å (**Supplementary Table 2**). These distances can be attributed to the coordination of Cu(II) with the single and double bonds of oxygen atoms in the carboxyl groups of CMC. Additionally, we observe that the coordination numbers of copper in CMC-Cu(II) are 2.2 ± 0.6 and 1.8 ± 0.7, respectively (**Supplementary Table 2**), indicating a total coordination number of approximately 4. This suggests that the carboxyl groups of CMC form a coordination structure closed to the square planar with Cu(II) by bidentate coordination mode.*

*To support and display the coordination structures and corresponding binding energy, CMC-Cu(II), CMC-Fe(II), and CMC-Ca(II), which exhibit different membrane-forming behaviors, are selected as representative examples. One glucose unit substituted with a carboxymethyl group is employed as model to simulate their theoretical optimal structures using DFT theory, with consideration of the solvent effect. **Fig. 3f** illustrates the coordination structure of the complexes. The Cu(II) ion forms a bidentate coordination with the two oxygen atoms of a single carboxyl group on the CMC chain (based on the result of **Fig. 3b**). The calculated coordination bond lengths of C=O and C-O are both 1.99 Å, which is in good agreement with the experimental fitting results (~2.14 ± 0.01 Å and ~2.00 ± 0.01 Å). It is speculated that in the actual reaction, the Cu(II) ion will form a bidentate coordination with the carboxyl group on another molecular chain, ultimately forming a typical tetra-coordinated configuration that approximates a regular tetrahedron. This is highly consistent with the structure inferred from EXAFS analysis. In the simulated system containing a single glucose unit, Fe(II) and Ca(II) display coordination configurations similar to that of Cu(II), yet the lengths of the coordination bonds are distinct. The calculated bond lengths of Fe=O and Fe-O are 1.97 Å and 1.98 Å respectively, whereas the bond lengths of Ca=O and Ca-O are 2.49 Å and 2.50 Å respectively. All these values are in line with the conventional theoretical values.*

The binding energy calculation further quantifies the strength of the coordination

*interaction at the thermodynamic level (**Fig. 3g**, **Supplementary Table 3**). The calculated binding energies of the three systems, CMC-Cu(II), CMC-Fe(II), and CMC-Ca(II), are -5.82 eV, -2.16 eV, and -1.15 eV respectively. All these values are negative, indicating that all three reactions are spontaneous. Moreover, the absolute values of the binding energies of the three systems followed the order: CMC-Cu(II) > CMC-Fe(II) > CMC-Ca(II). When the binding energy is negative, a larger absolute value indicates a more stable combination of substances. The binding energy results indicate that among the three systems, CMC-Cu(II) forms the most stable coordination complex, followed by CMC-Fe(II), and CMC-Ca(II) forms the least stable one. This is consistent with the previous experimental phenomena and characterization results"*

"Characterization

Molecular dynamics simulation

A molecular dynamics simulation was conducted to investigate the ultra-fast molecular assembly behavior driven by Cu(II) coordination force of CMC-Cu system at aqueous environment. Specifically, quantum chemistry calculations were first performed to optimize molecular geometries of CMC using the Gaussian 16 package at B3LYP/6-311+G(d) level of theory. The atomistic force field parameters for all ions and molecules are described by the OPLS-AA and water was taken from the SPCE model. In the CMC-Cu(II) simulation system, 10 CMC, 100 $CuCl_2$, 130 Na(I), and 11112 water molecules were randomly inserted into a simulation box with x = 7.2 nm, y = 7.2 nm, and z =7.2 nm.

Atomistic simulations were performed using the GROMACS package with cubic periodic boundary conditions. The equations for the motion of all atoms were integrated using a classic Verlet leapfrog integration algorithm with a time step of 2.0 fs. A cutoff radius of 1.4 nm was set for short-range van der Waals interactions and real-space electrostatic interactions. The particle-mesh Ewald (PME) summation method with an interpolation order of 4 and a Fourier grid spacing of 0.12 nm was employed to handle long range electrostatic interactions in reciprocal space. In all the three directions, periodic boundary conditions were imposed. Leapfrog algorithm was used to integrate the Newtonian equation of motion. The MD simulation was processed in an NPT ensemble and the simulation time is 40 ns. In NPT simulations, the pressure was maintained at 1 bar by the Berendsen barostat in an isotropic manner, and the temperature was maintained by the V-rescale thermostat at 298.15 K."

3. The suggested additional experiments

Reviewer comment: No electrical and mechanical properties characterization of hydrogel; Adhesion characteristics of hydrogel; no details of the method for in-situ fabrication of hydrogel on skin like face and neck; no EOG/EEG performance comparison with Ag/AgCl electrodes.

(i) Electrical property.

For epidermal electrodes, impedance is the primary electrical parameter that reflects their ability to acquire high-quality bioelectric signals from the skin surface. A low electrode-skin impedance indicates superior physiological signal detection and conduction capabilities. Typically, the electrode-skin interface is modeled as a parallel circuit (LCR circuit) consisting

of leakage resistance and capacitance (**Figure R26a**), which illustrates the measurement setup for the electrode-skin impedance in dual-electrode recording configurations. For each recording electrode, the electrode-skin impedance can be derived according to the following equation:

$$Z(\omega) = R_s + \frac{R_d/jC_d\omega}{R_d+1/jC_d\omega} = R_s + \frac{R_d}{1+jC_dR_d\omega},\quad \omega=2\pi f \qquad (1)$$

Where the resistor R_d represents the charge transfer impedance of the electrode-skin interface, while the capacitance C_d stands for the electric double layer between the electrode and skin. R_s is related to the skin surface conditions and underlying tissues, and R_{tissue} is the resistance of the dermis, subcutaneous tissue layers, and deeper tissues. Thus, maintaining high interfacial capacitance and low resistance at the electrode-skin interface (Eq.1) is crucial for minimizing bioelectrical signal attenuation and enhancing the overall signal-to-noise ratio (SNR)[9].

Therefore, to evaluate the electrical property of CMC-Cu(II), we conducted a systematic re-evaluation. In addition to the existing impedance data, we supplemented measurements of the membrane's capacitance and resistance and compared these parameters with those of a commercial electrode. The CMC-Cu(II) electrode has the same surface area as the conductive hydrogel electrode employed in the commercial product. The experimental results show that our CMC-Cu(II) membrane exhibits significantly reduced interfacial impedance—84% lower than that of commercial electrodes at 20 Hz. Additionally, none of the dynamic conditions compromise the functionality of the CMC-Cu(II) epidermal electrode, and sweating even enhances its electrical performance. The detailed discussions are as follows.

As shown in **Figure R26b**, the impedance of CMC-Cu(II) was significantly lower than that of the commercial Ag/AgCl electrode. At 20 Hz, the impedance of CMC-Cu(II) (9.75×10^4 Ω) was approximately one order of magnitude lower than that of the commercial electrode (6.04×10^5 Ω). Furthermore, the capacitance of CMC-Cu(II) (48.59 nF) was substantially higher than that of the commercial electrode (13.17 nF), while its resistance (173.23 Ω) was considerably lower than that of the commercial counterpart (683 Ω) (**Figure R26c**). These results collectively demonstrate that CMC-Cu(II) outperforms the commercial electrode as an epidermal electrode in terms of electrical performance, that is, the capabilities of physiological signal monitoring and acquisition.

In the revised supplementary materials, we have included additional electrical data for CMC-Cu(II) obtained under dynamic conditions. The membrane's electrical properties were evaluated under mechanical stretching-compression, sweating, and prolonged wear conditions. After stretching and compression (**Figure R26d inset i-iv**), the impedance shows negligible changes, indicating that the membrane retained its electrical conductivity after mechanical operation (**Figure R26d and e**). Upon exposure to sweat, an increase in capacitance and a decrease in resistance are observed at 20 Hz, indicating a reduction in impedance (**Figure R26f and g**). This behavior is likely attributed to the conductive nature of sweat and the presence of trace ions, such as Na(I), which enhance ionic mobility within the system. After 24 hours of

wear, a slight increase in impedance is observed. Specifically, under the test conditions, a series of electrical property measurements—impedance, capacitance, and resistance—were systematically conducted on the CMC-Cu(II) electrode every 3 hours. The impedance values at 20 Hz for the samples at 0 h, 3 h, 6 h, 9 h, 12 h, and 24 h are recorded as 9.75×104 Ω, 9.98×104 Ω, 9.99×104 Ω, 1.38×105 Ω, 1.36×105 Ω, and 2.21×105 Ω, respectively (**Figure R26h and i**). Both capacitance and resistance exhibited consistent and expected trends in accordance with Eq 1 (**Figure R26i**). The impedance of the CMC-Cu(II) electrode exhibits a slight increase within the first 6 hours, followed by a more substantial rise after 12 hours, and a more pronounced rise by 24 hours; however, it remains lower than that of the commercial electrode. This indicates that the physiological signal monitoring performance of the CMC-Cu(II) electrode experiences a very slight decrease within 6 hours, followed by a partial attenuation within 12 hours, and remains fully adequate for reliable, conventional physiological monitoring. These are expected due to the loss of water and environmental conditions.

Figure R26b has been updated and incorporated into the main text as **Fig. 4b**; **Figure R26c** has been added as **Supplementary Fig. 22**, and **Figure R26d-i** have been included as **Supplementary Fig. 23**. The corresponding results and discussions have also been added in the revised manuscript.

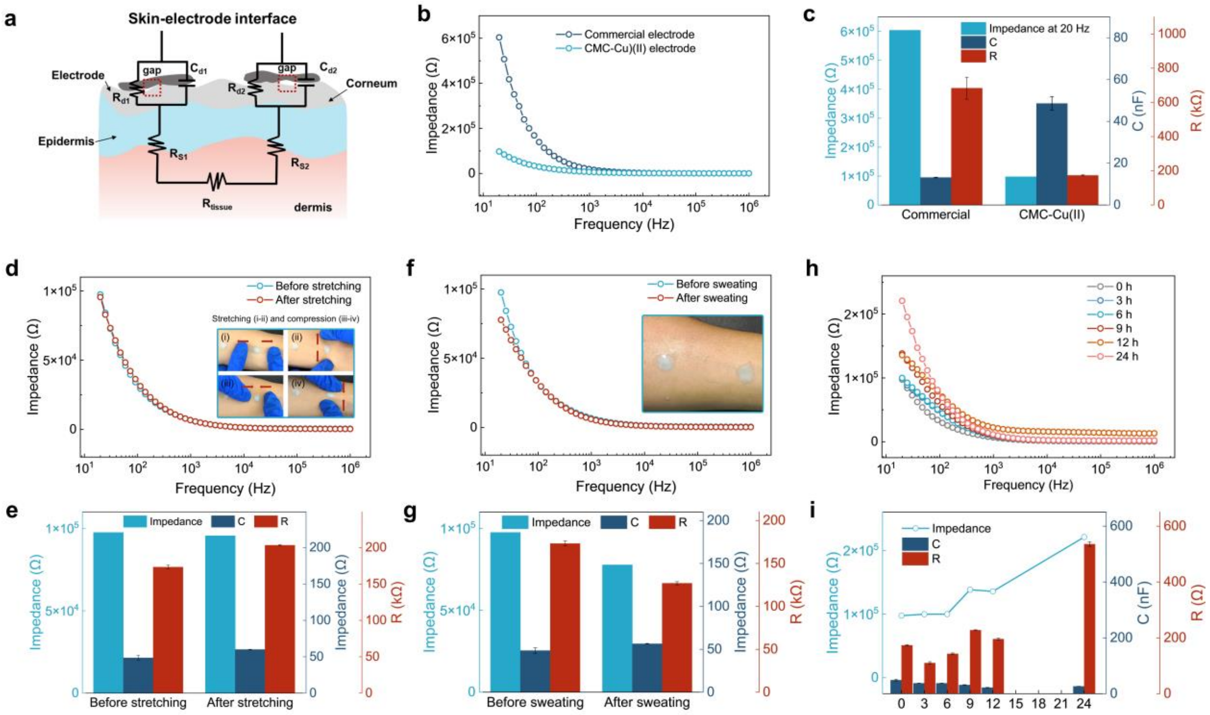

Figure R26. Electrical property of CMC-Cu(II) membrane as epidermal electrode. a Simplified LCR circuit model of the electrode-skin interface. **b** Skin-contact impedance values of CMC-Cu(II) and commercial electrodes within 20 Hz to 1000 kHz. **c** Skin-contact impedance values at 20 Hz, capacitance, and resistance of CMC-Cu(II) and commercial electrodes. **d** Skin-contact impedance values within 20 Hz to 1000 kHz of CMC-Cu(II) electrode before and after mechanical operation (stretching-compression). **e** Skin-contact impedance value at 20 Hz, capacitance, and resistance of CMC-Cu(II) electrodes before and after mechanical operation (stretching-compression). **f** Skin-contact impedance values within

20 Hz to 1000 kHz of CMC-Cu(II) electrode before and after sweating. **g** Skin-contact impedance value at 20 Hz, capacitance, and resistance of CMC-Cu(II) electrodes before and after sweating. **h** Skin-contact impedance values within 20 Hz to 1000 kHz of CMC-Cu(II) electrode within 24 h. **i** Skin-contact impedance value at 20 Hz, capacitance, and resistance of CMC-Cu(II) electrodes within 24 h.

Updates to the revised manuscript: According to Reviewer #3's comments, we added the above results in the revised manuscript on Pages 16-17.

> *"One crucial criterion for acquiring high-quality physiological signals is establishing a stable, low-impedance interface between electrodes and skin[32]. The CMC-Cu(II) electrodes demonstrate lower impedance than commercial Ag/AgCl electrodes (**Fig. 4b and Supplementary Fig. 22**). Specifically, compared with the corresponding commercial electrodes (6.04×10^{5} Ω), the impedance of the CMC-Cu(II) electrodes (9.75×10^{4} Ω) exhibit a notable reduction by approximately 84% at 20 Hz. Furthermore, the capacitance of CMC-Cu(II) (48.59 nF) is substantially higher than that of the commercial electrode (13.17 nF), while its resistance (173.23 Ω) is considerably lower than that of the commercial counterpart (683 Ω). Maintaining high interfacial capacitance and low resistance at the electrode-skin interface is crucial for minimizing bioelectrical signal attenuation and enhancing the overall signal-to-noise ratio (SNR)[9]. The superior electrical property of CMC-Cu(II) relative to commercial electrodes is attributed to its high ionic conductivity and conformal contact with the skin. Furthermore, practical applications of physiological signal monitoring frequently involve dynamic and changing environments, such as mechanical stretching, compression, and exposure to sweat. To evaluate the functional stability of CMC-Cu(II) under such dynamic conditions, its electrical performance (impedance) was measured following repeated mechanical deformation, sweat exposure, and prolonged wear (24 h) testing (**Supplementary Fig. 23**). After stretching and compression (**Supplementary Fig. 23a inset i-iv**), the impedance shows negligible changes, indicating that the membrane retained its electrical conductivity after mechanical operation (**Supplementary Fig. 23a and b**). Upon exposure to sweat, an increase in capacitance and a decrease in resistance (**Supplementary Fig. 23d**) are observed at 20 Hz, indicating a reduction in impedance (**Supplementary Fig. 23c**). This behavior is likely attributed to the conductive nature of sweat and the presence of trace ions, such as Na(I), which enhance ionic mobility within the system. After 24 hours of wear, a slight increase in impedance is observed. Specifically, under the test conditions, a series of electrical property measurements, impedance, capacitance, and resistance, were systematically conducted on the CMC-Cu(II) electrode every 3 hours. The impedance values at 20 Hz for the samples at 0 h, 3 h, 6 h, 9 h, 12 h, and 24 h are recorded as 9.75×104 Ω, 9.98×104 Ω, 9.99×104 Ω, 1.38×105 Ω, 1.36×105 Ω, and 2.21×105 Ω, respectively ((**Supplementary Fig. 23e and f**)). The impedance of the CMC-Cu(II) electrode exhibits a slight increase within the first 6 hours, followed by a more substantial rise after 12 hours, and a more pronounced rise by 24 hours; however, it remains lower than that of the commercial electrode. This indicates that the physiological signal monitoring performance of the CMC-Cu(II) electrode experiences a very slight decrease within 6 hours, followed by a partial attenuation within 12 hours, and remains*

fully adequate for reliable, conventional physiological monitoring. These are expected due to the loss of water and environmental conditions."

(ii) Mechanical and adhesion properties, as well as the method details for in-situ fabrication of hydrogel on skin.

To investigate the mechanical property of the CMC-Cu(II) membrane, we measured its tensile stress-strain curve. The results show an elongation at break of 98.66% and a fracture energy of 2.4×10^6 J·m^{-3}, which reflects the material's toughness. These findings demonstrate that the membrane exhibits sufficient flexibility and extensibility, making it suitable for skin-contact electronic devices under dynamic strain (**Figure R27a**). This result has been added to **Supplementary Fig. 20**.

In response to the reviewer's inquiry concerning adhesion, we performed a 90° peeling test on the CMC-Cu(II) membrane using pigskin (modulus comparable to human skin). The average adhesion strength was 6.05 N/m (**Figure R27b**). This value ensures a conformal skin-attached electronic device for electrophysiological signal acquisition. Based on both the measured adhesion strength and visual observations, the CMC-Cu(II) membrane exhibits no tendency to slip during operation. To provide a more comprehensive characterization of CMC-Cu(II), the adhesion strength data (**Figure R27b**) have been added to **Supplementary Fig. 8** in the supplementary materials, along with corresponding descriptions and test method incorporated into the revised main manuscript.

For the reviewer's suggestion to add details on the in-situ hydrogel fabrication, we have supplemented the procedure in the revised Supplementary Materials. As illustrated in **Figure R27c,** a laser-cut mask is first attached to the skin, CMC solution is dispensed into the circular aperture and spread uniformly, the mask is then removed, and Cu(II) solution is added to trigger rapid in-situ membrane formation. For clarity, **Figure R27c** has been incorporated as **Supplementary Fig. 7**, and the corresponding description has been added to the revised main manuscript.

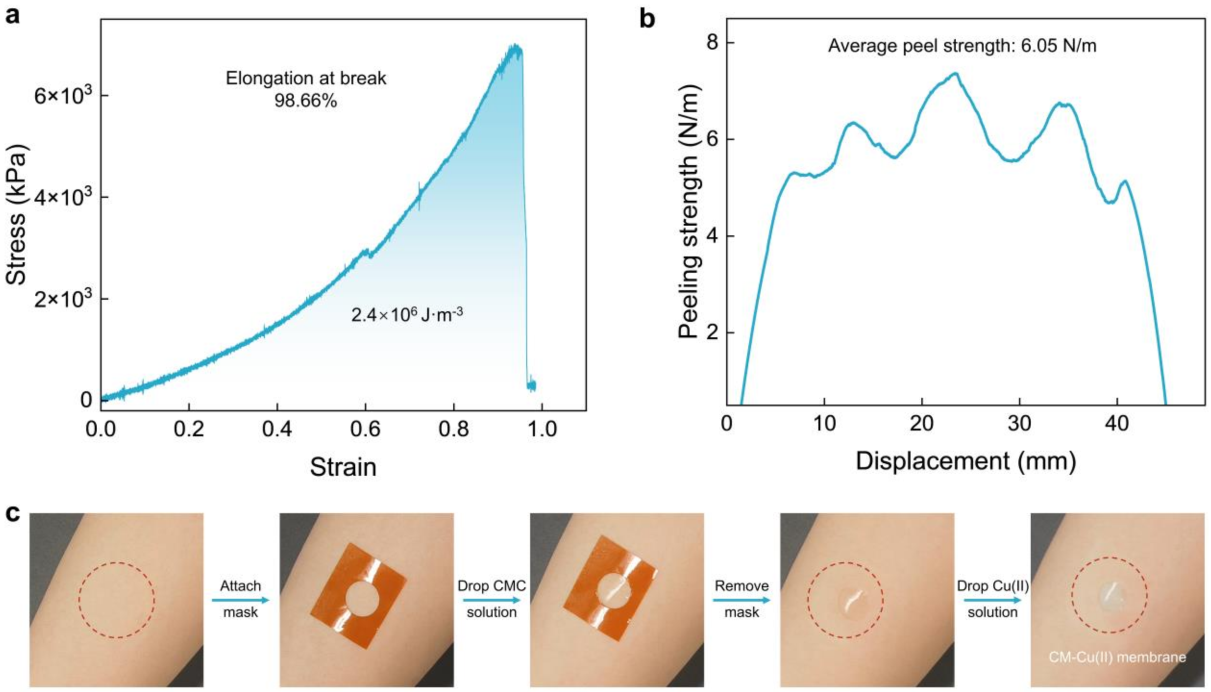

Figure R27. Mechanical and adhesion properties, as well as the method details for in-situ fabrication of hydrogel on skin. a Stress-strain curve displaying the mechanical strength of CMC-Cu(II) membrane. **b** 90° peeling test on pigskin showing the peeling strength of the CMC-Cu(II) membrane. **c** The formation of the CMC-Cu(II) membrane on the skin using the mask-forming method.

Updates to the revised manuscript: According to Reviewer #3's comments, we added the above information in the revised manuscript on Pages 16, 27, and 7.

About mechanical property, Pages 16 and 27:

> *"For accurate monitoring, sensors must maintain conformal contact with the skin, deforming synchronously during use. Based on the stress-strain curve, the CMC–Cu(II) membrane shows a fracture elongation of 98.66% and a fracture energy (toughness) of 2.4 × 10⁶ J·m⁻³ (**Supplementary Fig. 20**), indicating sufficient flexibility and ductility for use as a skin-contact electrode under dynamic strain environments during physiological signal monitoring."*
>
> *"Characterization*
> *The thickness of CMC-Cu(II) membranes was characterized using Hitachi SU-1510 SEM to obtain cross-sectional characterization data. To assess the conformal properties, a CMC-Cu(II) membrane was attached onto a wavy PVC mold and subjected to embrittle in liquid nitrogen. 90° peeling test and stress-strain curves were measured on the electronic universal testing machine (Instron Corporation -3369)."*

About adhesion property, Page 7:

> *"Furthermore, a 90° peeling test revealed an average adhesion strength of 6.05 N/m for the CMC-Cu(II) membrane (**Supplementary Fig. 8**), which enables a conformal contact*

and prevents slippage during skin deformation, thereby ensuring reliable mechanical coupling with the skin. These findings lays a solid foundation for the application of the CMC-Cu(II) membrane as an epidermal electronic device in electrophysiological signal monitoring."

About the method details for in-situ fabrication of hydrogel on skin, Page 7:

"Additionally, the preparation of CMC-Cu(II) membrane with defined size and shape on the skin via the mask-forming method is illustrated in ***Supplementary Fig.*** *7."*

(iii) EOG/EEG performance comparison between CMC-Cu(II) and commercial (Ag/AgCl) electrodes.

About EOG. In the revised manuscript, we have incorporated additional EOG data acquired using commercial electrodes (Ag/AgCl). The results demonstrate that both the CMC-Cu(II) and commercial electrodes, when placed at the outer canthus of the eye, are capable of capturing periodic EOG signals corresponding to blinking and ocular motility. During blinking events, both electrode types reliably detect the characteristic voltage patterns associated with each blink, with local magnification revealing precise signal morphologies (**Figure R28a and e**). The time-frequency spectrum, derived from Fourier transform analysis of the EOG signals, provides detailed insight into signal composition, further validating the stability and accuracy of the recordings (**Figure R28b and f**). In ocular motility trials, both the CMC-Cu(II) and commercial electrodes consistently record continuous EOG patterns that are clearly distinguishable from the transient, discrete signals generated during blinking (**Figure R28c and g**). Local magnification and time-frequency spectral analysis confirm these distinct signal features (**Figure R28d and h**). Notably, the peak patterns observed during blinking and ocular motility using both electrode types align closely with the actual physiological behaviors of the subjects. Furthermore, the amplitude of the signals recorded by the CMC-Cu(II) electrode is slightly higher than that of the commercial electrode, suggesting its enhanced capability in acquiring high-fidelity ocular signals.

The EOG data recorded by commercial electrode (**Figure R28e-h**) have been added as **Supplementary Fig. 24** in the revised supplementary materials.

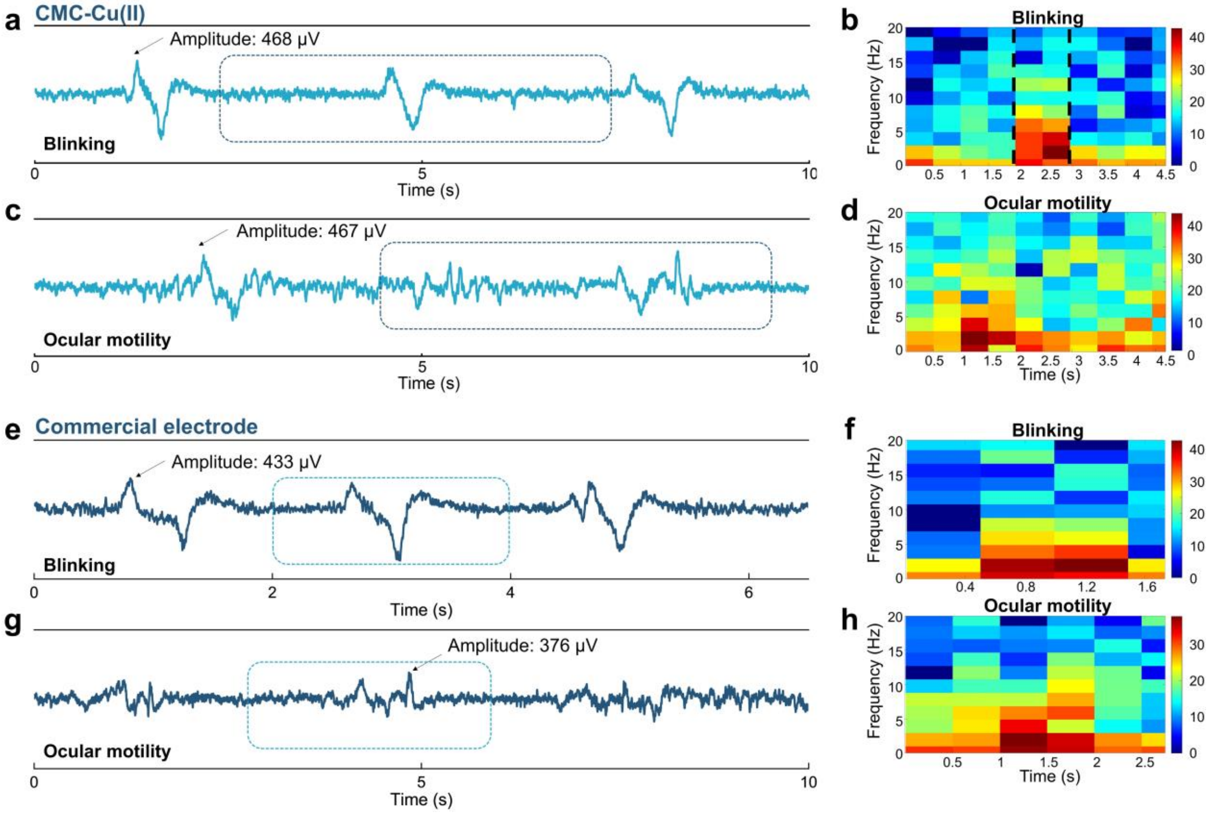

Figure R28. EOG performance comparison between CMC-Cu(II) and commercial (Ag/AgCl) electrodes. a-d Periodic EOG signals captured by the CMC-Cu(II) electrodes displaying movements involving blinking (**a**) and ocular motility (**c**) of the subject and corresponding time-frequency spectrum of a single cycle of EOG signal (**b and d**). **e-h** Periodic EOG signals captured by the commercial electrodes displaying movements involving blinking (**e**) and ocular motility (**g**) of the subject and corresponding time-frequency spectrum of a single cycle of EOG signal (**f and h**).

Updates to the revised manuscript: According to Reviewer #3's comments, we added the above information in the revised manuscript on Page 19.

> *"The application of the CMC-Cu (II) electrode in EOG monitoring as horizontal unipolar leads is demonstrated in* ***Fig. 4e****. Single CMC-Cu(II) electrodes at the outer corner of the eye capture periodic EOG signals, reflecting blinking and ocular motility[34]. For blinking, the CMC-Cu(II) electrode captures distinct voltage patterns for each blink event, with localized amplification illustrating precise signal characteristics (****Fig. 4f top****). The time-frequency spectrum derived from the Fourier transform of the EOG signal reveals detailed signal characteristics, thereby further substantiating its stability and accuracy (****Fig. 4g top****). Similarly, during ocular motility tests, the CMC-Cu(II) records persistent pattern of EOG signals corresponding to eye movements that displays distinct difference from the intermittent independent signal of blinking, with localized amplification and time-frequency spectrum analysis revealing detailed signal patterns (****Fig. 4f bottom and Fig. 4g bottom****). The above results align with the peak patterns observed in the subjects' blinking and ocular motility behaviors, which are recorded using commercial electrodes (****Supplementary Fig. 24****). Based on the amplitude of the acquired signals, the signal peak*

monitored by the CMC-Cu(II) electrode is slightly higher than that of the commercial electrode, demonstrating its capability to capture high-fidelity ocular signals."

About EEG. We have supplemented additional EEG data recorded while subjects listened to music using commercial Ag/AgCl electrodes. The signal analysis method remains consistent with that used for the CMC-Cu(II) electrodes: time-frequency spectra were derived via Fourier transformation. Both electrode types successfully captured spatial maps reflecting real-time brain activity on the human forehead (**Figure R29a and d**), and the corresponding time-frequency spectra provided fine-grained temporal resolution of neural dynamics (**Figure R29b and e**). Specifically, at any given moment, the energy distribution across distinct brainwave bands—delta, theta, alpha, and beta—reflects the intensity of underlying neural processes. The band exhibiting peak energy (Crimson) indicates the dominant brain activity at that time point. Furthermore, both electrode systems exhibited a similar trend in average concentration and relaxation coefficients (CMC-Cu(II): 50.60 and 63.29; commercial electrodes: 54.69 and 58.96) shown in **Figure R29c and f**, with relaxation values consistently exceeding concentration values—consistent with the widely recognized cognitive state during music listening.

It is important to note that EEG signals exhibit considerable inter- and intra-individual variability: even when exposed to the same auditory stimulus, different individuals, and the same individual under varying conditions, can display distinct patterns of brain activity. As a result, a direct quantitative comparison between the two electrode types may lack statistical significance. However, both electrode types demonstrate consistent overall trends and comparable time-frequency characteristics, suggesting that our CMC-Cu(II) electrode can capture EEG signals with accuracy comparable to that of commercial Ag/AgCl electrodes. In the revised manuscript, we have expanded the discussion on EEG data, including enhanced interpretation and analysis, which we believe provides readers with a more comprehensive and clearer understanding of the recorded neural activity.

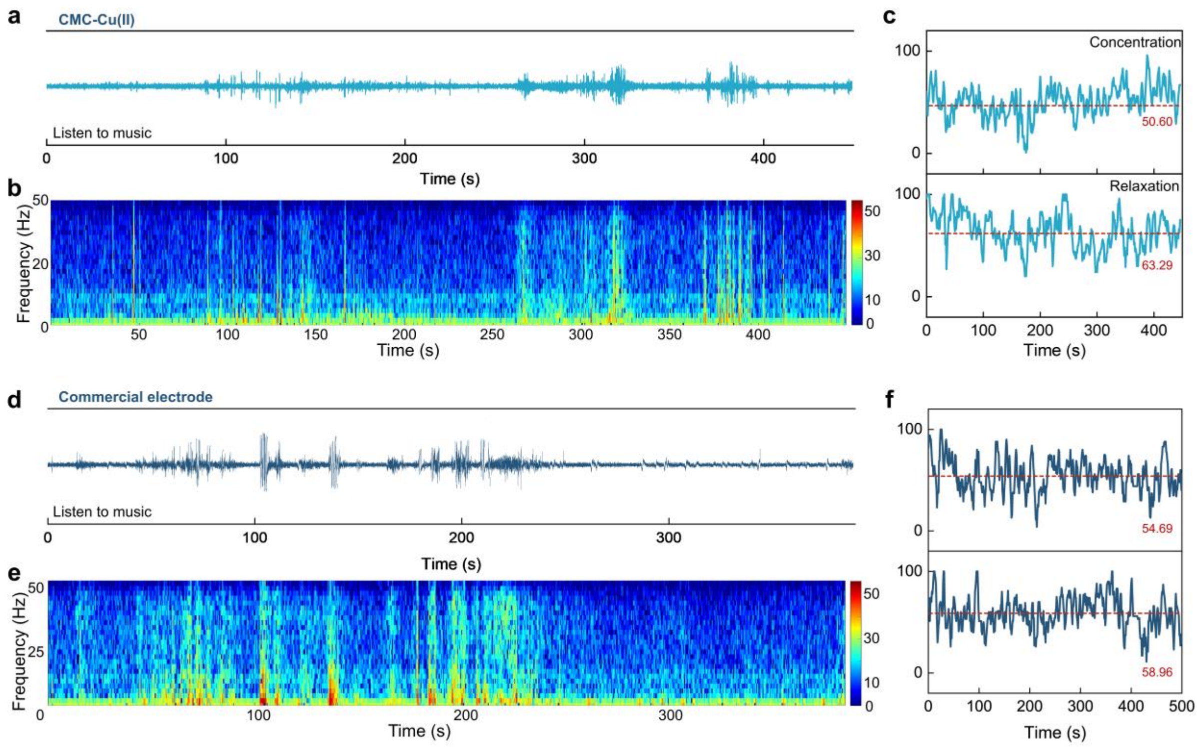

Figure R29. EEG performance comparison between CMC-Cu(II) and commercial (Ag/AgCl) electrodes. a-c EEG signals (**a**), corresponding time-frequency spectrum (**b**), and coefficients of concentration and relaxation (**c**) recorded by CMC-Cu(II) electrodes during the subject's cognitive engagement in music listening. **d-f** EEG signals (**d**), corresponding time-frequency spectrum (**e**), and coefficients of concentration and relaxation (**f**) recorded by commercial electrodes during the subject's cognitive engagement in music listening.

Updates to the revised manuscript: We revised the above content about enhancing the clarity and interpretability of EEG in the revised manuscript on Pages 19-21.

> *"Unipolar lead EEG collection offers a simple and efficient approach for analyzing electrical activity of specific brain regions. EEG signals can be categorized into distinct frequency bands based on their specific ranges, with each band corresponding to particular physiological and cognitive states. The δ wave (0.5–4 Hz) is predominantly observed during deep sleep and reflects the brain's restorative and recovery processes. The θ wave (4–8 Hz) is associated with light sleep, relaxation, or meditative states and is often enhanced during certain cognitive processing and memory-related tasks. The α wave (8–13 Hz) is most prominent during a relaxed, awake state with eyes closed, indicating cortical inhibition and relaxation; it diminishes significantly upon eye opening or when attention is actively engaged. The β wave (13–30 Hz) is linked to active thinking, concentration, anxiety, and arousal, reflecting the brain's engagement in information processing and task execution. The γ wave (>30 Hz) is closely associated with higher-order cognitive functions, including learning, perception, and multisensory integration, and becomes particularly evident in tasks requiring rapid coordination across distributed brain regions. Analyzing these frequency bands enables the characterization of dynamic brain activity across various cognitive states and behavioral tasks, offering critical*

*insights for neuroscience research and practical applications such as brain-computer interfaces. A CMC-Cu(II) electrode attached to a volunteer's forehead, where it intimately associates with decision-making, attention, executive function, and other cognitive processes (**Fig. 4h**), enables detection of prefrontal cortex activity by measuring potential differences between the recording and reference electrodes.*

*When the subjects listened to music, brain activities elicited by real-time stimuli from musical elements—such as sound, lyrics, and melody—are converted into electrical signals and captured by the CMC-Cu(II) electrode (**Fig. 4i**). These acquired signals are subsequently transformed into time-frequency spectra via Fourier transformation (**Fig. 4j**). The resulting energy density distribution (represented by color intensity) across frequency bands offers a more detailed representation of the subjects' cognitive processes during each time interval. In contrast, EEG signals recorded by CMC-Cu(II) during the participants' mathematical calculation tasks (**Supplementary Fig. 25a**) and their corresponding time-frequency spectra (**Supplementary Fig. 25b**) exhibited distinct patterns of frequency band energy distribution compared to those observed during music listening. This difference confirms that the CMC-Cu(II) electrode effectively captures the brain's real-time neural responses to varying external stimuli, thereby reflecting the dynamic interplay between the brain and its environment. Additionally, the EEG signal collection and analysis system simultaneously provides concentration and relaxation metrics across different tasks, which also show significant variation. Music listening activity shows average coefficients of concentration and relaxation at 50.60 and 63.29, respectively (total range 0-100) (**Fig. 4k**), while mathematical calculation displays 57.60 for concentration and 44.87 for relaxation (**Supplementary Fig. 25c**), which aligned with the general trend observed and the prevailing understanding. A localized results over 20 seconds further illustrates the stability and precision of CMC-Cu(II) recordings (**Supplementary Fig. 26a and 25d**), with the time-frequency spectrum displaying well-defined frequency bands (**Supplementary Fig. 26b and 25e**)[35, 36]. These results demonstrate the accuracy and practical application potential of CMC-Cu(II) as an electrophysiological sensor."*

Itemized list of response to the reviewers' remarks

(Black: Reviewers' remarks; Blue type: Our response)

Reviewer #2 (Remarks to the Author):

There are several grammatical errors and awkward phrases which should be corrected for better readability.

Author reply: We thank Reviewer #2 for this valuable feedback. We have carefully reviewed and revised the entire manuscript to correct grammatical errors and improve readability. Below we provide several representative examples of the revisions. Additional corrections can be found in the revised manuscript, where all changes are clearly marked in red font.

- **Complex sentence structures & spelling**

  The original version:

  *A notable example of thermally induced molecular self-assembly is the formation of milk skin (**Fig. 1ai-ii**), where heat unfolds the tertiary structure of coiled proteins, leading to the self-assembly of protein melecular s into an ordered milk lamellar membrane, driven by the synergistic effect of hydrophobic interactions, hydrogen bonding, and fat globules (**Fig. 1aii**)[19].*

  The modified versions in the revised manuscript on Pages 2, lines 43-46:

  *A notable example of thermally induced molecular self-assembly is the formation of milk skin (**Fig. 1ai-ii**). Heat unfolds the tertiary structure of coiled proteins, leading to the self-assembly of protein molecules into an ordered milk lamellar membrane. This process is driven by the synergistic effects of hydrophobic interactions, hydrogen bonding, and fat globules (**Fig. 1aii**)[19].*

- **Addition of missing information**

  The original version:

  In typical CMC structure, hydrogen ions at the carboxymethyl group on the C6 position are partially substituted by Na(I) ions.

  The modified versions in the revised manuscript on Pages 6, lines 111-114:

  In the typical commercially available CMC structure, the hydrogen atom on the hydroxyl group (–OH) at the C6 position of certain cellulose molecules are substituted with carboxymethyl groups (–CH_2COOH or –CH_2COONa). Most of these substituents terminate as sodium carboxylates (–COONa), in which the sodium ions (Na(I)) are ionically bonded to the carboxylate anions (–COO^-).

- **Incorrect verb tenses**

  The original version:

  Owing to the inherent biodegradability of CMC, the biodegradability of CMC-Cu(II) membrane in natural environments is investigated. Both CMC-Cu(II) membranes and polyethylene (PE) films are placed on the soil surface.

  The modified versions in the revised manuscript on Pages 7, lines 133-135:

Owing to the inherent biodegradability of CMC, the biodegradation of CMC-Cu(II) membrane in natural environments was investigated. Both CMC-Cu(II) membranes and polyethylene (PE) films were placed on the soil surface.

- **Redundant or wordy expressions**

The original version:

Specifically, under the test conditions, a series of electrical property measurements, impedance, capacitance, and resistance, were systematically conducted on the CMC-Cu(II) electrode every 3 hours.

The modified versions in the revised manuscript on Pages 6, lines 111-114:

Specifically, impedance, capacitance, and resistance of the CMC-Cu(II) electrode were systematically measured every 3 hours under the test condition.

Reviewer #3 (Remarks to the Author):

Authors revised the manuscript according to the reviewer's comments. Therefore, the reviewer thinks that the paper is good for publication in Nat. Comm.

Author reply: We thank Reviewer #3 for the positive evaluation and recommendation for publication in ***Nature Communications***. We greatly appreciate the reviewer's time and insightful comments, which have helped us improve the quality of our manuscript.

Reviewer #4 (Remarks to the Author):

[Editor's Note: This reviewer was invited to evaluate the response to Reviewer #1's evaluation] Overall, the revision is clearly improved. The authors have substantially strengthened the manuscript in quantification, added mechanistic support, and clarified several technical points raised by Reviewer #1. In particular, the response is materially stronger on thickness control, mechanical/electrical characterization, and mechanistic explanation of the Cu(II)-CMC assembly process.

However, Reviewer #1's concerns are not entirely resolved.

The concerns regarding novelty have only been partially addressed. The authors have now clarified that the claimed novelty lies not in CMC-metal coordination itself, but in the ultrafast, in situ, transfer-free formation of a conformal membrane directly from solution on the target substrate. This distinction is clearer than before. However, the response mainly differentiates the work from a relatively narrow subset of prior CMC-based studies, rather than fully establishing sufficient novelty against the broader literature on rapid or in situ membrane/hydrogel formation. On this point, I consider the response improved but not fully convincing.

The concern regarding limited quantification and vague mechanistic explanation has been addressed substantially better. The authors have added UV spectroscopy, XANES/EXAFS, molecular dynamics simulations, and DFT analysis, and the revised results provide a more quantitative explanation for the rapid coordination of Cu(II) with CMC and film formation. I consider this part of the problem has been largely resolved.

The concern regarding suitability for epidermal applications is only partially resolved. The authors have repositioned the system as an epidermal electronic membrane and provide additional performance arguments for electrode use. However, the skin-compatibility issue remains weaker than the current claim language. The added 24 h HUVEC live/dead assay supports low short-term cytotoxicity, but it does not directly resolve Reviewer #1's original concern about skin rash and true epidermal-use safety; the authors also explicitly state that no additional skin-irritation data were provided. Thus, this issue is improved, but not fully resolved.

In summary, the authors have made a serious effort and have resolved many of Reviewer #1's technical concerns. However, the two core issues upon which Reviewer #1's negative evaluation was based—novelty and skin compatibility/epidermal suitability—remain partially unresolved. Therefore, my overall assessment is that the response letter is substantial and represents a significant improvement to the manuscript, but it does not fully address all the issues initially raised by Reviewer #1.

Author reply: We sincerely thank Reviewer #4 for the constructive feedback. The reviewer still has concerns in two areas:

(i) concern regarding the novelty against the broader literature on rapid or in situ membrane/hydrogel formation;

(ii) concern about skin compatibility/epidermal suitability.

Below, we address these issues point-by-point.

1. Clarifying the Novelty of Our Work in Comparison to Prior Studies on Rapid or In Situ Membrane/Hydrogel Formation

To directly address the reviewer's concern, we have compiled a detailed comparison between our work and representative studies from prior literature (**Table R1)**. The comparison reveals several key distinctions that collectively define the novelty of our approach:

Our work uniquely combines (i) in situ formation directly on the target substrate without transfer; (ii) ultrafast fabrication within 2 seconds; (iii) a facile process under ambient conditions (no UV, heat, pressure, or crosslinkers); (iv) ultrathin (~3.4 μm) conformal features; and (v) direct use as an epidermal electrode without additional conductive components. This specific combination of features appears to be less common in previous reports on rapid or in situ membrane/hydrogel formation. As shown in **Table R1**, while some studies achieve in situ formation (Refs. 1–3) or rapid formation (Refs. 4–6), none combine all of the above features. Most reported works require more time, external energy, or additional crosslinkers, obtaining much thicker structures (>12 μm to >10,000 μm), and often need extra conductive components for electrode use.

Table R1. A detailed comparison between prior literatures and our work

	Fabrication mold	Fabrication time	Fabrication conditions	Thickness (μm)	Biocompatibility	Is it suitable for direct use as an epidermal electrode?
**Ref.1 (Wang B, et al., 2025)**	In situ	1 hour	Pressure & UV photo-polymerization	12.6	Primarily petroleum-based materials	Yes
**Ref.2 (Zhang M, et al., 2025)**	In situ	5 min	Temperature trigger	1000	Petroleum/ bio-based hybrid	No, require additional conductive component
**Ref.3 (Liu, G W, et al., 2024)**	In situ	10-20 min	Double-network crosslinking	>10000	Petroleum/ bio-based hybrid	No, for gastrointestinal drug delivery
**Ref.4 (Zhang L, et al., 2026)**	Rapid	5-30 s	Liquid-triggered crosslinking	>3000	Bio-based materials	No, for burn wound healing
**Ref.5 (Li L, et al., 2024)**	Rapid	15 s	Temperature trigger	≈2000	Bio-based materials	No, require additional conductive component
**Ref.6**	Rapid	15-60 min	Saltwater	Fibrous/bulk	Petroleum-	No,

**(Zhang Y, et al., 2024)**			trigger	hydrogel without defined thickness	based materials	as biomedical multifunction al materials
**Our work**	**In situ and rapid**	**2 s**	**Simple dipping-dipping**	**3.4**	**Bio-based materials**	**Direct use**

References:

1. Wang B, *et al.* Antifreezing Ultrathin Bioionic Gel-Based Wearable System for Artificial Intelligence-Assisted Arrhythmia Diagnosis in Hypothermia. *ACS Nano* **19**, 8176-8188 (2025).
2. Zhang M, et al. Flexible electromyography sensor with in situ gelation hydrogel for early diagnosis of lumbar spine diseases. *InfoMat* **7**(12), 70066 (2025).
3. Liu, G W, et al. Drinkable in situ-forming tough hydrogels for gastrointestinal therapeutics. *Nat. Mater.* **23**, 1292–1299 (2024).
4. Zhang L, et al. An Ultrafast Self-Gelling Versatile Hydrogel for Rapid Infected Burn Wound Repair in Military Medicine. *Adv. Funct. Mater.* **36**(29), 25541 (2026).
5. Li L, et al. Paintable, Fast Gelation, Highly Adhesive Hydrogels for High-fidelity Electrophysiological Monitoring Wirelessly. *Small* **21**, 2407996 (2025).
6. Zhang Y, et al. Saltwater-Induced Rapid Gelation of Photoredox-Responsive Mucomimetic Hydrogels. *Adv. Mater.* **36**, 2307356 (2024).

2. Clarifying the Skin Compatibility of Our Work

We agree that the 24 h HUVEC assay alone does not fully establish in vivo skin safety. As the Editor advised, performing skin irritation tests would turn this study into a clinical trial, which is beyond the scope of the present work. Therefore, we have taken the following actions:

- Qualified the language throughout the manuscript. For example, on Page 16, line 290, *"excellent biocompatibility"* has been changed to *"favorable biocompatibility"*
- Added a clear statement in the "Discussion" section on Page 25-26, lines 479-483):
  "It should be noted that, while the CMC-Cu(II) membrane shows favorable in vitro biocompatibility and conformal properties for epidermal applications, the 24 h cytotoxicity results only confirm safety within conventional monitoring durations. Future work including skin irritation and sensitization assessments (e.g., in accordance with ISO 10993 standards) are needed to evaluate long-term safety. Hence, this work provides a preliminary validation, with full regulatory assessment beyond its scope."

All changes are highlighted in red in the revised manuscript.
